# Supplementary material for: Stereospecific syn-dihalogenations and regiodivergent syn-interhalogenation of alkenes via vicinal double electrophilic activation strategy
Source: Nat Commun. 2024 May 2;15:3710. doi: 10.1038/s41467-024-47942-w (PMC11066093; doi:10.1038/s41467-024-47942-w)
Supplement: Supplementary file 1 — Supplementary Information [file 41467_2024_47942_MOESM1_ESM.pdf]

Stereospecific *syn*-dihalogenations and regiodivergent *syn*-interhalogenation of alkenes via vicinal double electrophilic activation strategy

Hyeon Moon‡, Jungi Jung‡, Jun-Ho Choi* & Won-jin Chung*

Department of Chemistry, Gwangju Institute of Science and Technology, Gwangju 61005, Republic of Korea.

e-mail: junhochoi@gist.ac.kr; wjchung@gist.ac.kr

Supplementary Information

1. Supplementary Notes ········ *S2*

2. Supplementary Methods ········ *S3*

2.1. Literature Preparations ········ *S3*

2.2. Preparation of Substrates ········ *S3*

2.3. Preparation of Thianthrene-*S*-Oxide (**19**) ········ *S13*

2.4. General Procedure I: *syn*-Dihalogenation of Alkenes ········ *S14*

2.5. General Procedure II: *syn*-Bromochlorination of Alkenes ········ *S20*

2.6. Solvent Survey for *syn*-Dichlorination of **14a** ········ *S26*

2.7. Reaction Condition Optimisation for *syn*-Bromochlorination of **14a** ········ *S26*

2.8. Reaction Condition Optimisation for *syn*-Bromochlorination of **14p** ········ *S27*

2.9. Mechanistic Validation of Reactive Species ········ *S28*

3. Supplementary Figures ········ *S28*

3.1. X-Ray Crystallographic Data ········ *S28*

3.2. Computational Study – NCI Analysis ········ *S31*

3.3. NMR Spectra ········ *S32*

4. Supplementary References ········ *S114*

1. Supplementary Notes

All reactions were performed in oven-dried (140 °C) or flame-dried glassware under an atmosphere of dry argon unless otherwise noted. Tetrahydrofuran (THF), diethyl ether (Et_2O), dichloromethane (CH_2Cl_2), and acetonitrile (MeCN) were dried by percolation through a column packed with neutral alumina, and a column packed with Q5 reactant, a supported copper catalyst for scavenging oxygen, under a positive pressure of argon. Dimethyl sulfoxide (DMSO) and dimethylformamide (DMF) were dried and stored over 3 Å molecular sieves prior to use. Toluene was dried with CaH_2, distilled under reduced pressure, and stored over 3 Å molecular sieves prior to use. 2,3-Dihydrofuran was distilled under argon prior to use. The following reagents were distilled from the indicated drying agents under argon prior to use: acetic acid (Ac_2O), triethylamine (CaH_2), and triflic anhydride (P_2O_5). The following reagents were distilled under reduced pressure prior to use: (*Z*)-4-hexen-1-ol, (*E*)-4-hexen-1-ol, (*Z*)-3-hexen-1-ol, isovaleraldehyde, hydrocinnamaldehyde, benzyl bromide, *tert*-butyl(chloro)diphenylsilane (TBDPSCl), and benzoyl chloride (BzCl). The following reagents were recrystallised from the indicated solvents prior to use: benzoic acid (hexanes), thianthrene (acetone), triphenylphosphine (hexanes), phthalimide (CH_2Cl_2/hexanes), imidazole (CH_2Cl_2/hexanes), 4-(dimethylamino)pyridine (CH_2Cl_2/hexanes), methyl (triphenylphosphoranylidene)acetate (CH_2Cl_2/hexanes), and ethyl (triphenylphosphoranylidene)acetate (CH_2Cl_2/hexanes), phenol (hexanes/petroleum ether). $Fe(NO_3)_3$•$9H_2O$ (Alfa, >98%), sodium bromide (Aldrich, 98%), tetra-*n*-butylammonium chloride (TBACl, Aldrich, 97%), tetra-*n*-butylammonium bromide (TBABr, Alfa, 98%), sodium hydride (Alfa, 60% in mineral oil), potassium *tert*-butoxide (ACROS, 98%), dichlorobis(triphenylphosphine)nickel(II) (ACROS, 98%), methyl magnesium chloride (MeMgCl, Aldrich, 3 M in THF), oxalyl chloride (Alfa, 98%), 2-thenoyl chloride (TCI, 99%), piperonylic acid (Aldrich, 99%), 4-bromobenzoyl chloride (Alfa, >98%), 4-nitrobenzoyl chloride (Alfa, 98%), and diisopropyl azodicarboxylate (DIAD, Alfa, 94%), 9-decyn-1-ol (TCI, >94%), $Ni(OAc)_2$•$4H_2O$ (Alfa, >99%), sodium borohydride (Alfa, 98%), sodium tetrafluoroborate (TCI, >98%), and ethylene diamine (Daejung, 98%) were used without further purification. Solvents and reagents for workup and chromatography were hexanes (Duksan, Extra Pure), ethyl acetate (EtOAc, Duksan, Extra Pure), CH_2Cl_2 (Duksan, Extra Pure), Et_2O (Daejung, Extra Pure), pentane (Daejung, Extra Pure), and isopropanol (Duksan, 99.5%). Quenching solutions and drying reagents for workup were magnesium sulphate (Duksan, Extra pure), sodium bicarbonate (Daejung, Extra Pure), potassium carbonate (Duksan, Extra pure), and sodium chloride (Daejung, Extra pure).

^{1}H and ^{13}C NMR spectra were recorded on a JEOL JCX-400 spectrometer (400 MHz, ^{1}H; 100 MHz, ^{13}C; 376 MHz, ^{19}F). Spectra were referenced to residual chloroform (7.26 ppm, ^{1}H; 77.16 ppm, ^{13}C) and hexafluorobenzene (–164.9 ppm, ^{19}F). Chemical shifts were reported in ppm, and multiplicities are indicated by s (singlet), d (doublet), t (triplet), q (quartet), pent (pentet), and m (multiplet). Coupling constants, *J*, are reported in Hertz. Kugelrohr distillation was carried out using a Büchi B585 glass oven with Büchi bulb-to-bulb distillation apparatus, and air bath temperatures (ABT) are reported. Filtration and column chromatography were performed using Merck 230-400 mesh silica gel. Analytical thin layer chromatography (TLC) was conducted on Merck silica gel 60 F_{254} TLC plates. Visualisation was accomplished with UV (254 nm) as well as potassium permanganate ($KMnO_4$) and ceric ammonium molybdate (CAM) staining solutions. ESI-HRMS was performed on a Bruker Impact II quadrupole-time-of-flight (Q-TOF) spectrometer at GIST Central Research Facilities (GCRF). EI-HRMS was performed on a JEOL JMS-700 MStation mass spectrometer at Korea Basic Science Institute (KBSI), Daegu Center. Data are reported in the form of *m/z*. X-ray crystallographic data for **17n** and **18n** were collected using a Rigaku Oxford Diffraction XtaLAB Synergy-S diffractometer using $Cu_{K\alpha}$ radiation (λ = 1.54184 Å) at GIST Central Research Facilities (GCRF). Melting point was determined using a Büchi M-560 melting point apparatus.

2. Supplementary Methods

2.1. Literature Preparations

The following compounds were prepared according to literature procedures: (*Z*)-[(Hex-4-en-1-yloxy)methyl]benzene (**14i**)[1] and 8-decyn-1-ol[2].

2.2. Preparation of Substrates

(*Z*)-hex-4-en-1-yl benzoate (**14a**)

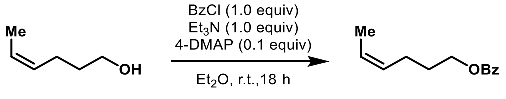

14a

To a stirred solution of 4-DMAP (294 mg, 2.41 mmol, 0.1 equiv), (*Z*)-4-hexen-1-ol (2.8 mL, 24 mmol, 1.0 equiv, *Z*/*E* = 98/2), and Et_3N (3.4 mL, 24 mmol, 1.0 equiv) in Et_2O (24 mL) was added benzoyl chloride (2.8 mL, 24 mmol, 1.0 equiv) at room temperature. After 18 hours (unoptimised), EtOAc (20 mL) was added, and the crude solution was washed with sat. aq. $NaHCO_3$ (50 mL), H_2O (50 mL × 2), dried over $MgSO_4$ (3 g), filtered through a glass frit, and concentrated in vacuo. The crude material was purified by flash column chromatography (SiO_2, ϕ = 3.0 cm, l = 9.0 cm) eluting with EtOAc/hexanes = 1/30 (R_f = 0.32 [254 nm/CAM (blue)]) to afford **14a** as a colourless oil (4.59 g, 93%). Further purification by Kugelrohr distillation (P = 0.15 mmHg, ABT = 140–155 °C) provided a colourless oil (4.34 g, 88%, *Z*/*E* = 99/1, contaminated with 1% of terminal alkene).

Data for **14a**:[3] HM-06-041

^{1}H NMR: (400 MHz, $CDCl_3$)

δ 8.07–8.04 (m, 2H), 7.58–7.53 (m, 1H), 7.47–7.42 (m, 2H), 5.56–5.48 (m, 1H), 5.45–5.38 (m, 1H), 4.33 (t, *J* = 6.6, 2H), 2.22 (appr. q, *J* = 7.3, 2H), 1.84 (appr. pent, *J* = 6.9, 2H), 1.63–1.61 (m, 3H).

(*Z*)-pent-3-en-1-yl benzoate (**14b**)[4]

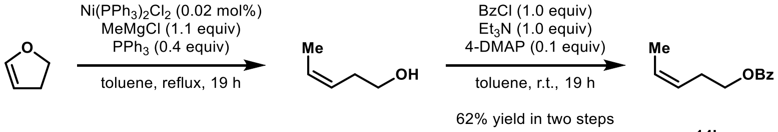

14b

To a stirred solution of triphenylphosphine (5.55 g, 21.2 mmol, 0.4 equiv) and $Ni(PPh_3)_2Cl_2$ (7 mg, 0.01 mmol, 0.02 mol%) in toluene (55 mL) was added MeMgCl (20 mL, 3 M in THF, 60 mmol, 1.1 equiv) at room temperature. The mixture was concentrated under reduced pressure (P = 0.15 mmHg) to evaporate the solvent (ca. 15 mL) without exposure to the ambient atmosphere. 2,3-Dihydrofuran (4.00 mL, 52.9 mmol, 1.0 equiv) was added, and the reaction mixture was refluxed. After 19 hours (unoptimised), sat. aq. NH_4Cl (150 mL) was added, and the organic layer was separated. The

aqueous layer was extracted with toluene (10 mL × 3), and the combined organic extracts were washed with brine (15 mL), dried over $MgSO_4$ (4 g), and filtered through a glass frit. Then, 4-DMAP (646 mg, 5.29 mmol, 0.10 equiv) was added to the crude solution, and the atmosphere was charged by argon. To the stirred crude solution were added benzoyl chloride (6.2 mL, 53 mmol, 1.0 equiv) and Et_3N (7.4 mL, 53 mmol, 1.0 equiv) at room temperature. After 19 hours, H_2O (100 mL) was added, and the organic layer was separated. The aqueous layer was extracted with EtOAc (20 mL × 3), and the combined organic extracts were dried over $MgSO_4$ (2 g), filtered through a glass frit, and concentrated in vacuo. The crude material was purified by flash column chromatography (SiO_2, ϕ = 5.0 cm, l = 11 cm) eluting with EtOAc/hexanes = 2/98 (R_f = 0.25 [254 nm/CAM (blue)]) to afford **14b** as a colourless oil (7.49 g, 74%). Further purification by Kugelrohr distillation (P = 0.15 mmHg, ABT = 155 °C) provided a colourless oil (6.27 g, 62%, *Z*/*E* = >99/1 contaminated with 1% of terminal alkene).

Data for **14b**: HM-07-087

^{1}H NMR: (400 MHz, $CDCl_3$)
δ 8.05–8.03 (m, 2H), 7.57–7.50 (m, 1H), 7.46–7.42 (m, 2H), 5.66–5.58 (m, 1H), 5.50–5.44 (m, 1H), 4.33 (t, *J* = 6.9, 2H), 2.53 (appr. q, *J* = 7.0, 2H), 1.67 (d, *J* = 6.7, 3H).

^{13}C NMR: (100 MHz, $CDCl_3$)
δ 166.8, 133.0, 130.5, 129.7, 128.5, 127.1, 125.4, 64.5, 26.7, 13.1.

HRMS (ESI): $[M+H]^+$ calcd for $C_{12}H_{15}O_2$: 191.1067; found: 191.1066.

(*Z*)-hex-3-en-1-yl benzoate (**14c**)

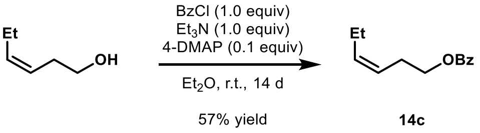

To a stirred solution of 4-DMAP (62 mg, 0.51 mmol, 0.1 equiv), (*Z*)-3-hexen-1-ol (0.60 mL, 5.1 mmol, 1.0 equiv), and Et_3N (0.71 mL, 5.1 mmol, 1.0 equiv) in Et_2O (10 mL) was added benzoyl chloride (0.59 mL, 5.1 mmol, 1.0 equiv) at room temperature. After 14 d (unoptimised), EtOAc (10 mL) and H_2O (5 mL) were added, and the organic layer was separated. The aqueous layer was extracted with EtOAc (10 mL × 3), and the combined organic extracts were dried over $MgSO_4$ (1 g), filtered through a glass frit, and concentrated in vacuo. Then, the crude material was purified by flash column chromatography (SiO_2, ϕ = 3.5 cm, l = 10 cm) eluting with CH_2Cl_2/hexanes = 1/3 (R_f = 0.33 [254 nm/CAM (blue)]) to afford **14c** as a colourless oil (602 mg, 58%). Further purification by Kugelrohr distillation (P = 0.15 mmHg, ABT = 160 °C) provided a colourless oil (595 mg, 57%, *Z*/*E* = >99/1).

Data for **14c**:[5] HM-07-013

^{1}H NMR: (400 MHz, $CDCl_3$)
δ 8.05–8.03 (m, 2H), 7.57–7.53 (m, 1H), 7.46–7.42 (m, 2H), 5.57–5.51 (m, 1H), 5.44–5.38 (m, 1H), 4.32 (t, *J* = 6.7, 2H), 2.52 (appr. q, *J* = 6.8, 2H), 2.10 (appr. pent, *J* = 7.2, 2H), 0.98 (t, *J* = 7.5, 3H).

(Z)-9-methyldec-6-en-1-yl benzoate (**14d**)

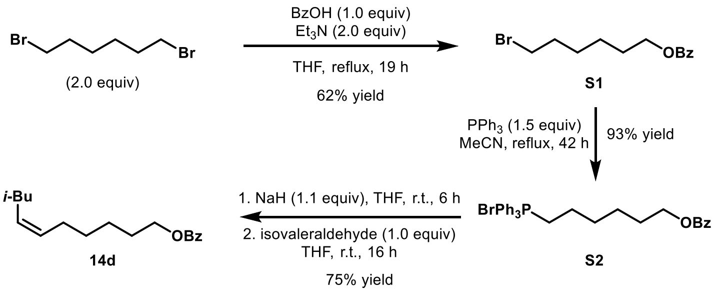

To a stirred solution of benzoic acid (1.83 g, 15.0 mmol, 1.0 equiv) in THF (60 mL) was added 1,6-dibromohexane (4.60 mL, 30.0 mmol, 2.0 equiv) dropwise at room temperature. Then, triethylamine (4.20 mL, 30.0 mmol, 2.0 equiv) was added, and the reaction mixture was refluxed. After 19 hours (unoptimised), H_2O (50 mL) was added, and the organic layer was separated. The aqueous layer was extracted with EtOAc (30 mL × 3), and the combined organic extracts were dried over $MgSO_4$ (5.0 g), filtered through a glass frit, and concentrated in vacuo. The crude material was purified by flash column chromatography (SiO_2, ϕ = 5.0 cm, l = 12 cm) eluting with EtOAc/hexanes = 1/20 to afford **S1** as a colourless oil (3.64 g, 85%). Further purification by Kugelrohr distillation (P = 0.20 mmHg, ABT = 210–240 °C) provided a colourless oil (2.64 g, 62%).

A stirred solution of 6-bromohexyl benzoate (**S1**, 2.64 g, 9.30 mmol, 1.0 equiv) and triphenylphosphine (3.65 g, 13.9 mmol, 1.5 equiv) in MeCN (10 mL) was refluxed. After 42 hours (unoptimised), the reaction mixture was cooled to room temperature and poured into Et_2O (50 mL). The mixture was vigorously stirred for 2 hours, and the precipitated solid was filtered, and dried in vacuo to afford **S2** as a white solid (4.73 g, 93%).

A mixture of phosphonium bromide **S2** (2.20 g, 4.01 mmol, 1.1 equiv) and NaH (160 mg, 4.01 mmol, 1.1 equiv, 60% in mineral oil) in THF (24 mL) was stirred at room temperature. After 16 hours (unoptimised), isovaleraldehyde (0.4 mL, 3.7 mmol, 1.0 equiv) was added dropwise. After 13 hours (unoptimised), CH_2Cl_2 (30 mL) and brine (30 mL) were added, and the organic layer was separated. The aqueous layer was extracted with CH_2Cl_2 (15 mL × 3), and the combined organic extracts were dried over $MgSO_4$ (2 g), filtered through a glass frit, and concentrated in vacuo. The crude material was purified by flash column chromatography (SiO_2, ϕ = 5.0 cm, l = 10 cm) eluting with CH_2Cl_2/hexanes = 1/5 (R_f = 0.19 [254 nm/CAM (blue)]) to afford **14d** as a colourless oil (763 mg, 76%). Further purification by Kugelrohr distillation (P = 0.15 mmHg, ABT = 230 °C) provided a colourless oil (756 mg, 75%, Z/E = 94/6).

Data for **S1**:[6] JGJ-07-097

^{1}H NMR: (400 MHz, $CDCl_3$)

δ 8.04 (dd, J = 8.4, 1.3, 2H), 7.56 (tt, J = 7.4, 1.2, 1H), 7.44 (t, J = 7.7, 2H), 4.33 (t, J = 6.6, 2H), 3.42 (t, J = 6.7, 2H), 1.95–1.84 (m, 2H), 1.84–1.74 (m, 2H), 1.56–1.43 (m, 4H).

Data for **14d**: HM-06-098

^{1}H NMR: (400 MHz, $CDCl_3$)

δ 8.06–8.04 (m, 2H), 7.57–7.54 (m, 1H), 7.46–7.42 (m, 2H), 5.44–5.33 (m, 2H), 4.32 (t, J = 6.7, 2H), 2.08–2.03 (m, 2H), 1.93–1.90 (m, 2H), 1.81–1.74 (m, 2H), 1.60 (appr. nonet, J = 6.7, 1H), 1.49–1.38 (m, 4H), 0.89 (d, J = 6.7, 6H).

^{13}C NMR: (100 MHz, $CDCl_3$)
δ 166.8, 132.9, 130.7, 130.2, 129.7, 129.1, 128.5, 65.2, 36.5, 29.8, 29.5, 28.8, 27.3, 25.9, 22.6.
HRMS (ESI): $[M+H]^+$ calcd for $C_{18}H_{27}O_2$: 275.2006; found: 275.2027.

(*Z*)-1,6-diphenyl 3-hexene (**14e**)[7]

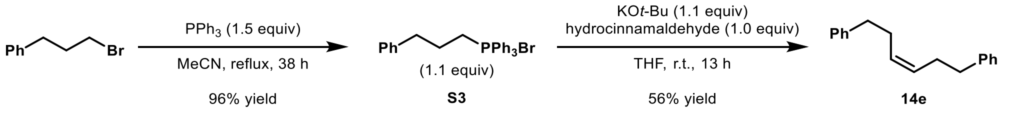

A stirred solution of hydrocinnamyl bromide (3.0 mL, 20 mmol, 1.0 equiv) and triphenylphosphine (7.76 g, 29.6 mmol, 1.5 equiv) in MeCN (20 mL) was refluxed. After 38 hours (unoptimised), the reaction mixture was cooled to room temperature, and the precipitated solid was filtered and washed with Et_2O (20 mL × 3) to give **S3** as a white powder (8.77 g, 96%).

A solution of potassium *tert*-butoxide (224 mg, 2.00 mmol, 1.1 equiv) and phosphonium bromide **S3** (924 mg, 2.00 mmol, 1.1 equiv) in THF (12 mL) was stirred for 4 hours at room temperature, and hydrocinnamaldehyde (0.24 mL, 1.8 mmol, 1.0 equiv) was added. After 13 hours (unoptimised), CH_2Cl_2 (15 mL) and H_2O (20 mL) were added, and the organic layer was separated. The aqueous layer was extracted with CH_2Cl_2 (10 mL × 3), and the combined organic extracts were dried over $MgSO_4$ (1.5 g), filtered through a glass frit, and concentrated in vacuo. The crude material was purified by flash column chromatography (SiO_2, ϕ = 3.5 cm, l = 12 cm) eluting with CH_2Cl_2/hexanes = 1/40 (R_f = 0.33 [254 nm/CAM (blue)]) to afford **14e** as a colourless oil (240 mg, 56%, *Z*/*E* = 92/8). Further purification by Kugelrohr distillation (P = 0.15 mmHg, ABT = 250 °C) provided a white solid (240 mg, 56%, *Z*/*E* = 92/8).

Data for **14e**:[8] HM-07-009
^{1}H NMR: (400 MHz, $CDCl_3$)
δ 7.29–7.26 (m, 4H), 7.20–7.15 (m, 5H), 5.46–5.39 (m, 2H), 2.58 (t, J = 7.8, 4H), 2.33–2.28 (m, 4H).

(*Z*)-hex-4-en-1-yl thiophene-2-carboxylate (**14f**)

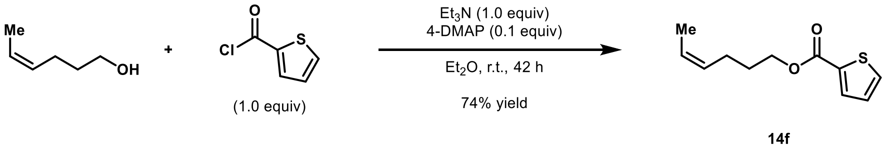

To a stirred solution of 4-DMAP (62 mg, 0.51 mmol, 0.1 equiv), (*Z*)-4-hexen-1-ol (0.58 mL, 5.0 mmol, 1.0 equiv, *Z*/*E* = 98/2), and Et_3N (0.70 mL, 5.0 mmol, 1.0 equiv) in Et_2O (10 mL) was added 2-thenoyl chloride (0.54 mL, 5.0 mmol, 1.0 equiv) at room temperature. After 42 hours (unoptimised), H_2O (10 mL) was added, and the organic layer was separated. The aqueous layer was extracted with EtOAc (10 mL × 3), and the combined organic extracts were dried over $MgSO_4$ (5 g), filtered through a glass frit, and concentrated in vacuo. The crude material was purified by flash column chromatography

(SiO_2, ϕ = 4.0 cm, l = 12 cm) eluting with CH_2Cl_2/hexanes = 1/2 (R_f = 0.29 [254 nm]) to afford **14f** as a yellow oil (773 mg, 74%).

Data for **14f**: JGJ-08-097

^{1}H NMR: (400 MHz, $CDCl_3$)

δ 7.80 (dd, J = 3.7, 1.2, 1H), 7.55 (dd, J = 4.9, 1.2, 1H), 7.10 (dd, J = 5.2, 4.0, 1H), 5.56–5.48 (m, 1H), 5.44–5.37 (m, 1H), 4.30 (t, J = 6.6, 2H), 2.20 (appr. q, J = 7.3, 2H), 1.82 (appr. pent, J = 6.9, 2H), 1.63–1.60 (m, 3H).

^{13}C NMR: (100 MHz, $CDCl_3$)

δ 162.4, 134.2, 133.4, 132.3, 129.1, 127.8, 125.2, 64.7, 28.6, 23.3, 12.9.

HRMS (ESI): $[M+H]^+$ calcd for $C_{11}H_{15}O_2S$: 211.0787; found: 211.0793.

(Z)-hex-4-en-1-yl benzo[d][1,3]dioxole-5-carboxylate (**14g**)

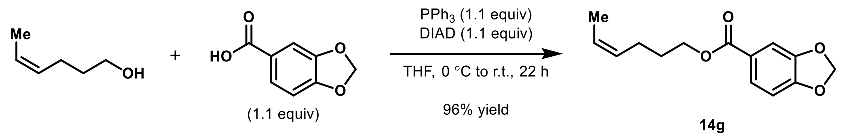

To a stirred solution of piperonylic acid (1.10 g, 6.60 mmol, 1.1 equiv), triphenylphosphine (1.73 g, 6.60 mmol, 1.1 equiv), (Z)-4-hexen-1-ol (0.7 mL, 6.0 mmol, 1.0 equiv, Z/E = 98/2) in THF (30 mL) was added DIAD (1.3 mL, 6.6 mmol, 1.1 equiv) at 0 °C. Then, the reaction mixture was warmed up to room temperature. After 2 hours, Et_2O (30 mL) and H_2O (30 mL) were added, and the organic layer was separated. The aqueous layer was extracted with CH_2Cl_2 (10 mL × 3), and the combined organic extracts were dried over $MgSO_4$ (2 g), filtered through a glass frit, and concentrated in vacuo. The crude material was purified by flash column chromatography (SiO_2, ϕ = 3.5 cm, l = 12 cm) eluting with CH_2Cl_2/hexanes = 1/4 (R_f = 0.29 [254 nm/$KMnO_4$]) to afford **14g** as a yellow oil (1.49 g, 100%). Further purification by Kugelrohr distillation (P = 0.15 mmHg, ABT = 230 °C) provided a yellow oil (1.43 g, 96%, Z/E = 98/2).

Data for **14g**: HM-08-033

^{1}H NMR: (400 MHz, $CDCl_3$)

δ 7.66 (dd, J = 8.2, 1.8, 1H), 7.47 (d, J = 1.8, 1H), 6.84 (d, J = 8.2, 1H), 6.04 (s, 2H), 5.55–5.46 (m, 1H), 5.45–5.37 (m, 1H), 4.28 (t, J = 6.6, 2H), 2.20 (appr. q, J = 7.3, 2H), 1.81 (appr. pent, J = 6.9, 2H), 1.63–1.60 (m, 3H).

^{13}C NMR: (100 MHz, $CDCl_3$)

δ 166.1, 151.7, 147.8, 129.3, 125.4, 125.2, 124.7, 109.6, 108.1, 101.9, 64.5, 28.7, 23.4, 12.9.

HRMS (ESI): $[M+H]^+$ calcd for $C_{14}H_{17}O_4$: 249.1121; found: 249.1117.

(*Z*)-2-(hex-4-en-1-yl)isoindoline-1,3-dione (**14h**)

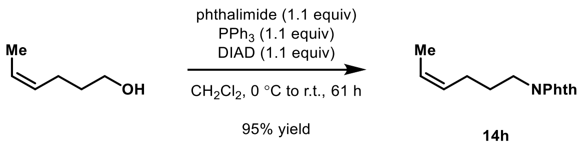

To a stirred solution of phthalimide (890 mg, 6.05 mmol, 1.1 equiv), triphenylphosphine (1.59 g, 6.05 mmol, 1.1 equiv), and (*Z*)-4-hexen-1-ol (0.64 mL, 5.5 mmol, 1.0 equiv, *Z*/*E* = 98/2) in CH_2Cl_2 (28 mL) was added DIAD (1.2 mL, 6.1 mmol, 1.1 equiv) at 0 °C. Then, the reaction mixture was warmed up to room temperature. After 61 hours (unoptimised), H_2O (30 mL) was added, and the organic layer was separated. The aqueous layer was extracted with CH_2Cl_2 (20 mL × 3), and the combined organic extracts were dried over $MgSO_4$ (2 g), filtered through a glass frit, and concentrated in vacuo. The crude material was purified by flash column chromatography (SiO_2, ϕ = 3.5 cm, l = 12 cm) eluting with EtOAc/hexanes = 1/10 (R_f = 0.37 [254 nm/$KMnO_4$]) to afford **14h** as a colourless oil (1.20 g, 95%). Further purification by Kugelrohr distillation (P = 0.15 mmHg, ABT = 240 °C) provided a colourless oil (1.19 g, 95%, *Z*/*E* = 98/2).

Data for **14h**:[9] HM-06-074

^{1}H NMR: (400 MHz, $CDCl_3$)

δ 7.86–7.82 (m, 2H), 7.73–7.68 (m, 2H), 5.51–5.54 (m, 1H), 5.42–5.35 (m, 1H), 3.69 (t, *J* = 7.3, 2H), 2.11 (appr. q, *J* = 7.4, 2H), 1.75 (appr. pent, *J* = 7.5, 2H) 1.61–1.59 (m, 3H).

(*Z*)-*tert*-butyl(hex-4-en-1-yloxy)diphenylsilane (**14j**)

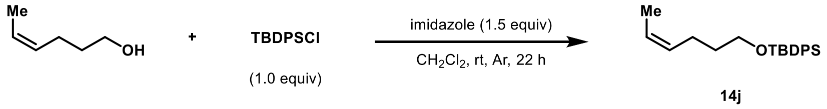

To a stirred solution (*Z*)-4-hexen-1-ol (0.47 mL, 4.0 mmol, 1.0 equiv) and imidazole (410 mg, 6.0 mmol, 1.5 equiv) in CH_2Cl_2 (8 mL) was added TBDPSCl (1.05 mL, 4.0 mmol, 1.0 equiv) at room temperature. After 22 hours (unoptimised), H_2O (40 mL) was added, and the organic layer was separated. The aqueous layer was extracted with CH_2Cl_2 (40 mL × 3), and the combined organic extracts were dried over $MgSO_4$ (5 g), filtered through a glass frit, and concentrated in vacuo. The crude material was purified by flash column chromatography (SiO_2, ϕ = 4.0 cm, l = 10 cm) eluting with EtOAc/hexanes = 1/30 (R_f = 0.60 [254 nm]) to afford **14j** as a colourless oil (1.31 g, 97%). Further purification by Kugelrohr distillation (P = 0.15 mmHg, ABT = 270 °C) provided a colourless oil (1.27 g, 94%).

Data for **14j**:[1] JGJ-07-087

^{1}H NMR: (400 MHz, $CDCl_3$)

δ 7.68–7.66 (m, 4H), 7.44–7.35 (m, 6H), 5.48–5.32 (m, 2H), 3.67 (t, *J* = 6.4, 2H), 2.14 (q, *J* = 7.3, 2H), 1.65–1.58 (m, 5H), 1.06 (s, 9H).

Ethyl (2*E*,6*Z*)-octa-2,6-dienoate (**14k**)[1]

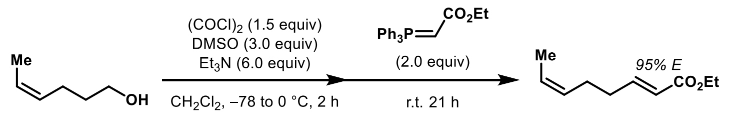

14k

To a stirred solution of oxalyl chloride (0.51 mL, 6.0 mmol, 1.5 equiv) in CH_2Cl_2 (17 mL) was added DMSO (0.85 mL, 12 mmol, 3.0 equiv) at –78 °C. Then, a solution of (*Z*)-4-hexen-1-ol (0.47 mL, 4.0 mmol, 1.0 equiv, *Z*/*E* = 98/2) in CH_2Cl_2 (1 mL) and Et_3N (3.4 mL, 24 mmol, 6.0 equiv) were added dropwise. After 2 hours, the reaction mixture was warmed up to 0 °C. Then, a solution of ethyl (triphenylphosphoranylidene)acetate (2.79 g, 8.00 mmol, 2.0 equiv) in CH_2Cl_2 (6 mL) was added. After 21 hours (unoptimised), H_2O (30 mL) was added, and the organic layer was separated. The aqueous layer was extracted with Et_2O (30 mL × 3), and the combined organic extracts were dried over $MgSO_4$ (3 g), filtered through a glass frit, and concentrated in vacuo. The crude material was purified by flash column chromatography (SiO_2, ϕ = 5.0 cm, *l* = 14 cm) eluting with EtOAc/hexanes = 1/99 (R_f = 0.16 [254 nm/$KMnO_4$]) to afford **14k** as a colourless oil (568 mg, 84%). Further purification by Kugelrohr distillation (P = 0.15 mmHg, ABT = 160 °C) provided a colourless oil (560 mg, 83%, 6*Z*/6*E* = >99:1, 2*E*/2*Z* = 95/5).

Data for **14k**: HM-06-052

^{1}H NMR: (400 MHz, $CDCl_3$)
δ 7.00–6.92 (m, 1H), 5.86–5.81 (m, 1H), 5.54–5.46 (m, 1H), 5.40–5.33 (m, 1H), 4.21–4.15 (m, 2H), 2.29–2.16 (m, 4H), 1.62–1.59 (m, 3H), 1.30–1.24 (m, 3H).

^{13}C NMR: (100 MHz, $CDCl_3$)
δ 166.8, 148.7, 128.9, 125.2, 121.7, 60.3, 32.2, 25.5, 14.4, 12.9.

HRMS (EI): $[M]^+$ calcd for $C_{10}H_{16}O_2$: 168.1150; found: 168.1152.

(*E*)-hex-4-en-1-yl benzoate (**14l**)

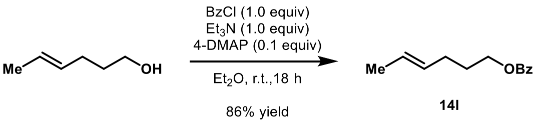

14l

To a stirred solution of 4-DMAP (116 mg, 0.950 mmol, 0.10 equiv), (*E*)-4-hexen-1-ol (1.11 mL, 9.47 mmol, 1.0 equiv, *E* only), and Et_3N (1.32 mL, 9.47 mmol, 1.0 equiv) in Et_2O (10 mL) was added benzoyl chloride (1.1 mL, 9.5 mmol, 1.0 equiv) at room temperature. After 18 hours (unoptimised), H_2O (15 mL) was added, and the organic layer was separated. The aqueous layer was extracted with EtOAc (15 mL × 3), and the combined organic extracts were washed with brine (20 mL), dried over $MgSO_4$ (1 g), filtered through a glass frit, and concentrated in vacuo. The crude material was purified by flash column chromatography (SiO_2, ϕ = 4.0 cm, *l* = 13 cm) eluting with EtOAc/hexanes = 1/49 (R_f = 0.34 [254 nm/CAM (blue)]) to afford **14l** as a colourless oil (1.73 g, 89%). Further purification by Kugelrohr distillation (P = 0.15 mmHg, ABT = 160 °C) provided a colourless oil (1.66 g, 86%).

Data for **14l**:[3] HM-06-059

^{1}H NMR: (400 MHz, $CDCl_3$)

δ 8.06–8.03 (m, 2H), 7.57–7.55 (m, 1H), 7.46–7.42 (m, 2H), 5.53–5.41 (m, 2H), 4.32 (t, *J* = 6.6, 2H), 2.17–2.12 (m, 2H), 1.83 (appr. pent, *J* = 7.0, 2H), 1.66–1.65 (m, 3H).

Methyl (2*E*,6*Z*)-octa-2,6-dienoate (**14m**)[1]

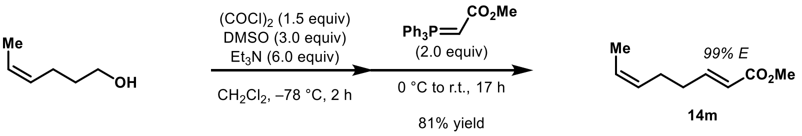

To a stirred solution of oxalyl chloride (0.26 mL, 3.0 mmol, 1.5 equiv) in CH_2Cl_2 (8.4 mL) was added DMSO (0.43 mL, 6.0 mmol, 3.0 equiv) at –78 °C. Then, a solution of (*Z*)-4-hexen-1-ol (0.23 mL, 2.0 mmol, 1.0 equiv, *Z*/*E* = 98/2) in CH_2Cl_2 (1 mL) and Et_3N (1.7 mL, 12 mmol, 6.0 equiv) were added dropwise. After 2 hours, the reaction mixture was warmed up to 0 °C. Then, a solution of methyl (triphenylphosphoranylidene)acetate (1.34 g, 4.00 mmol, 2.0 equiv) in CH_2Cl_2 (4 mL) was added. After 17 hours (unoptimised), H_2O (30 mL) was added, and the organic layer was separated. The aqueous layer was extracted with Et_2O (20 mL × 3), and the combined organic extracts were dried over $MgSO_4$ (5 g), filtered, and concentrated in vacuo. The crude material was purified by flash column chromatography (SiO_2, ϕ = 4.0 cm, l = 12 cm) eluting with EtOAc/hexanes = 1/100 (R_f = 0.15 [254 mm, $KMnO_4$]) to afford **14m** as a colourless oil (251 mg, 81%, 6*Z*/6*E* = >99:1, 2*E*/2*Z* = 99:1).

Data for **14m**: JGJ-08-095

^{1}H NMR: (400 MHz, $CDCl_3$)

δ 6.97 (dt, *J* = 13.1, 6.6, 1H), 5.89–5.81 (m, 1H), 5.54–5.46 (m, 1H), 5.39–5.29 (m, 1H), 3.72 (s, 3H), 2.30–2.17 (m, 4H), 1.60 (dd, *J* = 6.7, 0.9, 3H).

^{13}C NMR: (100 MHz, $CDCl_3$)

δ 167.2, 149.1, 128.8, 125.3, 121.3, 51.6, 32.2, 25.5, 12.9.

HRMS (ESI): $[M+H]^+$ calcd for $C_9H_{15}O_2$: 155.1067; found: 155.1065.

(*Z*)-2-(pent-3-en-1-yl)isoindoline-1,3-dione (**14n**)[4]

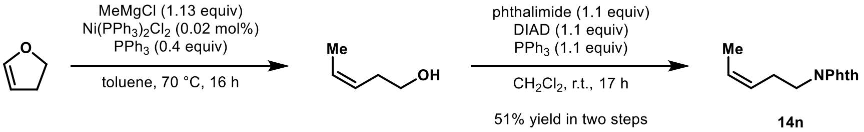

To a stirred solution of triphenylphosphine (4.20 g, 16.0 mmol, 0.4 equiv) and $Ni(PPh_3)_2Cl_2$ (5 mg, 0.008 mmol, 0.02 mol%) in toluene (50 mL) was added MeMgCl (15.0 mL, 3 M in Et_2O, 45.2 mmol, 1.13 equiv) at room temperature. The mixture was concentrated under reduced pressure (P = 0.15 mmHg) to evaporate the solvent (ca. 12 mL) without exposure to the ambient atmosphere. 2,3-Dihydrofuran (3.0 mL, 40 mmol, 1.0 equiv) was added, and the reaction mixture

was refluxed. After 16 hours (unoptimised), sat. aq. NH_4Cl (200 mL) was added, and the organic layer was separated. The aqueous layer was extracted with Et_2O (50 mL × 2), and the combined organic extracts were dried over $MgSO_4$ (5 g), filtered through a glass frit, and concentrated in vacuo. The crude material was filtered through a short pad of silica gel with *n*-pentane and concentrated in vacuo.

To a stirred solution of phthalimide (6.47 g, 44.0 mmol, 1.1 equiv) and triphenylphosphine (11.5 g, 44.0 mmol, 1.1 equiv) in CH_2Cl_2 (100 mL) was added the prepared pentenol at room temperature. The reaction mixture was cooled to 0 °C, and DIAD (7.0 mL, 44 mmol, 1.1 equiv) was added. The reaction mixture was warmed up to room temperature. After 17 hours (unoptimised), H_2O (100 mL) was added, and the organic layer was separated. The aqueous layer was extracted with CH_2Cl_2 (50 mL × 3), and the combined organic extracts were dried over $MgSO_4$ (10 g), filtered, and concentrated in vacuo. The crude material was purified by flash column chromatography through SiO_2 (ϕ = 4.0 cm, l = 10 cm) eluting with EtOAc/hexanes = 1/10 (R_f = 0.32 [254 nm]) to afford **14n** as a white solid (4.40 g, 51%).

Data for **14n**: JGJ-08-060

^{1}H NMR: (400 MHz, $CDCl_3$)
δ 7.84 (dd, *J* = 5.5, 3.1, 2H), 7.71 (dd, *J* = 5.5, 3.1, 2H), 5.59–5.50 (m, 1H), 5.43–5.35 (m, 1H), 3.73 (t, *J* = 7.2, 2H), 2.45 (appr. q, *J* = 7.2, 2H), 1.58–1.53 (m, 3H).

^{13}C NMR: (100 MHz, $CDCl_3$)
δ 168.6, 134.0, 132.3, 127.3, 125.9, 123.3, 37.6, 26.2, 12.9.

HRMS (ESI): $[M+H]^+$ calcd for $C_{13}H_{14}NO_2$: 216.1019; found: 216.1024.

(*Z*)-hex-4-en-1-yl 4-bromobenzoate (**14o**)

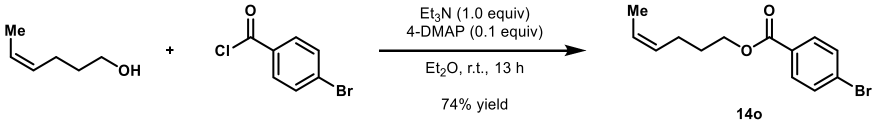

To a stirred solution of 4-DMAP (122 mg, 1.00 mmol, 0.10 equiv), (*Z*)-4-hexen-1-ol (1.2 mL, 10 mmol, 1.0 equiv, *Z*/*E* = 98/2), and Et_3N (1.4 mL, 10 mmol, 1.0 equiv) in Et_2O (10 mL) was added 4-bromobenzoyl chloride (2.20 g, 10.0 mmol, 1.0 equiv) at 0 °C. Then, the reaction mixture was warmed up to room temperature. After 13 hours (unoptimised), H_2O (15 mL) was added, and the organic layer was separated. The aqueous layer was extracted with Et_2O (50 mL × 2), and the combined organic extracts were dried over $MgSO_4$ (5 g), filtered, and concentrated in vacuo. The crude material was purified by flash column chromatography (SiO_2, ϕ = 4.0 cm, l = 10.0 cm) eluting with CH_2Cl_2/hexanes = 1/3 (R_f = 0.24 [254 nm]) to afford **14o** as a colourless oil (2.13 g, 75%). Further purification by Kugelrohr distillation (P = 0.15 mmHg, ABT = 140–170 °C) provided a colourless oil (2.09 g, 74%).

Data for **14o**: JGJ-08-061

^{1}H NMR: (400 MHz, $CDCl_3$)
δ 7.92–7.88 (m, 2H), 7.60–7.56 (m, 2H), 5.59–5.50 (m, 1H), 5.43–5.35 (m, 1H), 4.32 (t, *J* = 6.6, 2H), 2.20 (appr. q, *J* = 7.3, 2H), 1.83 (appr. pent, *J* = 6.9, 2H), 1.63–1.59 (m, 3H).

^{13}C NMR: (100 MHz, $CDCl_3$)
δ 166.0, 131.8, 131.2, 129.5, 129.1, 128.1, 125.2, 64.9, 28.6, 23.4, 12.9.

HRMS (ESI): $[M+H]^+$ calcd for $C_{13}H_{16}{}^{79}BrO_2$, $C_{13}H_{16}{}^{81}BrO_2$: 283.0328 (100.0%), 285.0309 (98.6%); found: 283.0342 (100.0%), 285.0321 (93.4%).

(*Z*)-hex-4-en-1-yl 4-nitrobenzoate (**14p**)

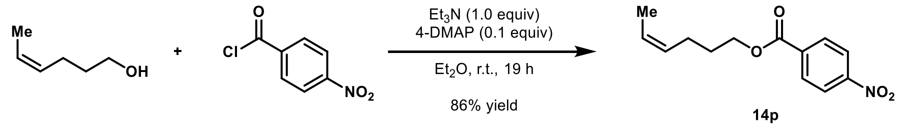

To a stirred solution of 4-DMAP (244 mg, 2.00 mmol, 0.10 equiv), (*Z*)-4-hexen-1-ol (2.3 mL, 20.0 mmol, 1.0 equiv, *Z*/*E* = 98/2), and Et_3N (2.8 mL, 20 mmol, 1.0 equiv) in Et_2O (30 mL) was added 4-nitrobenzoyl chloride (3.70 g, 20.0 mmol, 1.0 equiv) at 0 °C. Then, the reaction mixture was warmed up to room temperature. After 19 hours (unoptimised), H_2O (30 mL) was added, and the organic layer was separated. The aqueous layer was extracted with EtOAc (15 mL × 3), and the combined organic extracts were dried over $MgSO_4$ (5 g), filtered, and concentrated in vacuo. The crude material was purified by flash column chromatography (SiO_2, ϕ = 4.0 cm, l = 13 cm) eluting with EtOAc/hexanes = 1/15 (R_f = 0.32 [254 nm]) to afford **14p** as a pale yellow oil (4.46 g, 89%). Further purification by Kugelrohr distillation (P = 0.15 mmHg, ABT = 250 °C) provided a pale yellow oil (4.27 g, 86%).

Data for **14p**: JGJ-08-042

^{1}H NMR: (400 MHz, $CDCl_3$)
δ 8.31–8.26 (m, 2H), 8.23–8.17 (m, 2H), 5.58–5.48 (m, 1H), 5.45–5.36 (m, 1H), 4.38 (t, J = 6.6, 2H), 2.22 (appr. q, J = 7.2, 2H), 1.87 (appr. pent, J = 6.9, 2H), 1.63–1.57 (m, 3H).

^{13}C NMR: (100 MHz, $CDCl_3$)
δ 164.9, 150.6, 135.9, 130.8, 128.9, 125.4, 123.7, 65.6, 28.5, 23.3, 12.9.

HRMS (ESI): $[M+H]^+$ calcd for $C_{13}H_{15}NO_4$: 250.1074; found: 205.1067.

(*Z*)-dec-8-en-1-yl benzoate (**14q**)[10]

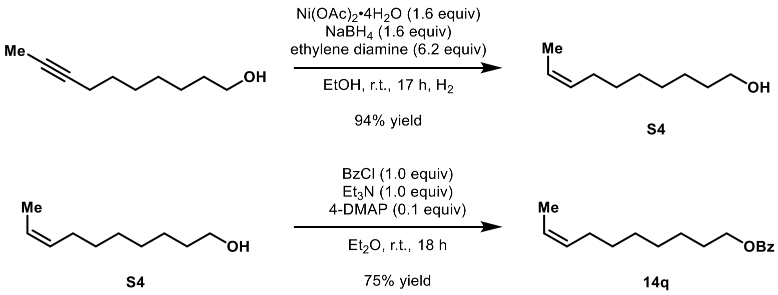

To a stirred solution of $Ni(OAc)_2•4H_2O$ (3.16 g, 12.7 mmol, 1.6 equiv) in EtOH (13 mL) was added a solution of $NaBH_4$ (480 mg, 12.7 mmol, 1.6 equiv) in EtOH (13 mL) at room temperature, and the flask was purged with H_2. Then,

ethylene diamine (3.4 mL, 51 mmol, 6.2 equiv) and a solution of 8-decyn-1-ol (1.26 g, 8.19 mmol, 1.0 equiv) in EtOH (13 mL) were added sequentially. After 17 hours (unoptimised), the reaction mixture was filtered through a short celite pad and concentrated in vacuo to afford **S4** as a pale yellow oil (1.20 g, 94%).

To a stirred solution of 4-DMAP (24 mg, 0.20 mmol, 0.1 equiv), **S4** (313 mg, 2.00 mmol, 1.0 equiv), and Et_3N (0.28 mL, 2.0 mmol, 1.0 equiv) in Et_2O (5 mL) was added benzoyl chloride (0.23 mL, 2.0 mmol, 1.0 equiv) at room temperature. After 18 hours (unoptimised), H_2O (5 mL) was added, and the organic layer was separated. The aqueous layer was extracted with EtOAc (10 mL × 3), and the combined organic extracts were dried over $MgSO_4$ (5 g), filtered through a glass frit, and concentrated in vacuo. The crude material was purified by flash column chromatography (SiO_2, ϕ = 3.5 cm, l = 9.0 cm) eluting with EtOAc/hexanes = 1/15 (R_f = 0.39 [254 nm/$KMnO_4$]) to afford **14q** as a pale yellow oil (455 mg, 87%). Further purification by Kugelrohr distillation (P = 0.30 mmHg, ABT = 280 °C) provided a colourless oil (390 mg, 75%).

Data for **14q**: JGJ-09-042

^{1}H NMR: (400 MHz, $CDCl_3$)

δ 8.05–8.03 (m, 2H), 7.57–7.54 (m, 1H), 7.46–7.42 (m, 2H), 5.48–5.34 (m, 2H), 4.32 (t, J = 6.7, 2H), 2.05–2.00 (m, 2H), 1.80–1.73 (m, 2H), 1.61–1.57 (m, 4H), 1.46–1.40 (m, 2H), 1.37–1.30 (m, 5H).

^{13}C NMR: (100 MHz, $CDCl_3$)

δ 166.9, 132.9, 130.9, 130.7, 129.7, 128.5, 123.9, 65.3, 29.6, 29.33, 29.30, 28.9, 26.9, 26.2, 12.9.

HRMS (ESI): $[M+Na]^+$ calcd for $C_{17}H_{24}NaO_2$: 283.1669; found: 283.1667.

2.3. Preparation of Thianthrene-*S*-Oxide (**19**)

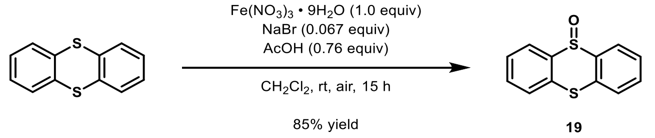

To a stirred solution of thianthrene (4.92 g, 22.7 mmol, 1.0 equiv), $Fe(NO_3)_3 \cdot 9H_2O$ (9.18 g, 22.7 mmol, 1.0 equiv), and NaBr (0.157 g, 1.52 mmol, 0.067 equiv) in CH_2Cl_2 (50 mL) was added acetic acid (0.99 mL, 17 mmol, 0.76 equiv) under air. After 15 hours, sat. aq. $NaHCO_3$ (50 mL) was added, and the organic layer was separated. The aqueous layer was extracted with CH_2Cl_2 (50 mL × 3), and the combined organic extracts were dried over $MgSO_4$ (5 g), filtered through a glass frit, and concentrated in vacuo. The crude material was purified by Kugelrohr distillation (P = 0.15 mmHg, ABT = 220–240 °C) to give **19** as a white solid (5.11 g, 97%). Further purification by recrystallisation from EtOAc provided colourless needle-type crystals (4.45 g, 85%).

Data for **19**:[11] HM-06-048

^{1}H NMR: (400 MHz, $CDCl_3$)

δ 7.94 (d, J = 7.6, 2H), 7.64 (d, J = 7.6, 2H), 7.56 (t, J = 7.6, 2H), 7.44 (t, J = 7.6, 2H).

2.4. General Procedure I: *syn*-Dihalogenation of Alkenes

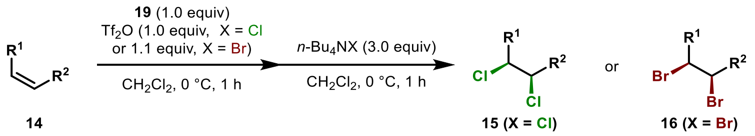

To a stirred solution of alkene (**14**, 1.00 mmol, 1.0 equiv) and thianthrene-*S*-oxide (**19**, 232 mg, 1.00 mmol, 1.0 equiv) in CH_2Cl_2 (4 mL) was added Tf_2O (168 μL, 1.00 mmol, 1.0 equiv for X = Cl or 185 μL, 1.10 mmol, 1.1 equiv for X = Br) at 0 °C. After 1 hour, a solution of *n*-Bu_4NCl (834 mg, 3.00 mmol, 3.0 equiv) or *n*-Bu_4NBr (967 mg, 3.00 mmol, 3.0 equiv) in CH_2Cl_2 (8 mL) was added. After 1 hour, H_2O (10 mL) was added, and the organic layer was separated. The aqueous layer was extracted with CH_2Cl_2 (10 mL × 3), and the combined organic extracts were dried over $MgSO_4$ (2 g), filtered through a glass frit, and concentrated in vacuo. The residue was diluted with Et_2O (5 mL), filtered through a pad of SiO_2 (ϕ = 2.0 cm, *l* = 4.0 cm, Et_2O, 150 mL), and concentrated in vacuo. The crude material was purified by flash column chromatography on SiO_2 to afford **15** or **16**. **15** was further purified by Kugelrohr distillation.

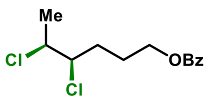

Flash column chromatography (SiO_2, ϕ = 5.5 cm, *l* = 15 cm, EtOAc/hexanes = 1/30, R_f = 0.30 [254 nm/CAM (blue)], dry loaded, SiO_2 3.6 g) and Kugelrohr distillation (P = 0.15 mmHg, ABT = 220 °C) afforded **15a** (169 mg, 61%, >99:1 dr) as a colourless oil.

Data for (4*R**,5*S**)-4,5-dichlorohexyl benzoate (**15a**):[2] HM-06-023

^{1}H NMR: (400 MHz, $CDCl_3$)

δ 8.06–8.03 (m, 2H), 7.59–7.55 (m, 1H), 7.47–7.43 (m, 2H), 4.42–4.33 (m, 2H), 4.12 (appr. pent, *J* = 6.6, 1H), 4.00 (ddd, *J* = 9.2, 6.7, 2.8, 1H), 2.26–2.07 (m, 2H), 1.98–1.87 (m, 2H), 1.65 (d, *J* = 6.4, 3H).

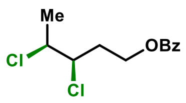

Addition of *n*-Bu_4NCl at –40 °C. Flash column chromatography (SiO_2, ϕ = 5.0 cm, *l* = 11 cm, CH_2Cl_2/hexanes = 1/5, R_f = 0.23 [254 nm/$KMnO_4$]) and Kugelrohr distillation (P = 0.15 mmHg, ABT = 210 °C) afforded **15b** (164 mg, 63%, 94:6 dr) as a colourless oil.

Data for (3*R**,4*S**)-3,4-dichloropentyl benzoate (**15b**): HM-08-085

^{1}H NMR: (400 MHz, $CDCl_3$)

δ 8.06–8.03 (m, 2H), 7.60–7.56 (m, 1H), 7.48–7.46 (m, 2H), 4.61–4.57 (m, 1H), 4.53–4.47 (m, 1H), 4.21–4.12 (m, 2H), 2.57 (dddd, *J* = 15.0, 8.5, 5.8, 2.4, 1H), 2.19–2.10 (dddd, *J* = 14.7, 9.8, 4.9, 4.6, 1H), 1.67 (d, *J* = 6.1, 3H).

1.63 (d, *J* = 6.7, 0.20H for the minor diastereomer).

^{13}C NMR: (100 MHz, $CDCl_3$)

δ 166.5, 132.3, 130.1, 129.7, 128.6, 63.5, 61.8, 60.3, 34.3, 22.3.

HRMS (ESI): $[M+Na]^+$ calcd for $C_{12}H_{14}{}^{35}Cl^{35}ClNaO_2$, $C_{12}H_{14}{}^{37}Cl^{35}ClNaO_2$, $C_{12}H_{14}{}^{37}Cl^{37}ClNaO_2$: 283.0263 (100.0%), 285.0235 (65.2%), 287.0210 (11.0%); found: 283.0266 (100.0%), 285.0237 (62.2%), 287.0208 (9.7%).

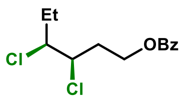

Flash column chromatography (SiO_2, ϕ = 4.0 cm, l = 15 cm, EtOAc/hexanes = 1/40, R_f = 0.38 [254 nm/CAM (blue)], dry loaded, SiO_2 2.5 g) and Kugelrohr distillation (P = 0.15 mmHg, ABT = 240 °C) afforded **15c** (154 mg, 56%, >99:1 dr) as a colourless oil.

Data for (3R^*,4S^*)-3,4-dichlorohexyl benzoate (**15c**): HM-07-020

^{1}H NMR: (400 MHz, $CDCl_3$)

δ 8.05–8.03 (m, 2H), 7.59–7.55 (m, 1H), 7.47–7.43 (m, 2H), 4.63–4.57 (m, 1H), 4.54–4.48 (m, 1H), 4.20 (ddd, J = 9.5, 6.7, 2.4, 1H), 4.01 (ddd, J = 9.9, 6.9, 3.2, 1H), 2.59 (dddd, J = 15.0, 8.9, 6.1, 2.4, 1H), 2.22–2.13 (ddt, J = 15.0, 9.8, 5.0, 1H), 2.07 (appr. dpent, J = 7.2, 3.3, 1H), 1.91–1.80 (m, 1H), 1.09 (t, J = 7.3, 3H).

^{13}C NMR: (100 MHz, $CDCl_3$)

δ 166.5, 133.3, 130.1, 129.7, 128.6, 67.6, 61.8, 61.7, 34.0, 28.4, 10.7.

HRMS (ESI): $[M+H]^+$ calcd for $C_{13}H_{17}{}^{35}Cl^{35}ClO_2$, $C_{13}H_{17}{}^{37}Cl^{35}ClO_2$, $C_{13}H_{17}{}^{37}Cl^{37}ClO_2$: 275.0600 (100.0%), 277.0572 (65.4%), 279.0548 (11.1%); found: 275.0601 (100.0%), 277.0572 (62.1%), 279.0545 (8.6%).

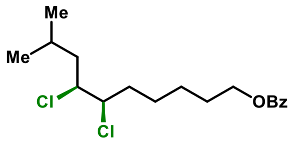

Flash column chromatography twice (SiO_2, ϕ = 4.0 cm, l = 12 cm, CH_2Cl_2/hexanes = 1/3, R_f = 0.19 [254 nm/CAM (blue)]), then ϕ = 3.0 cm, l = 7.0 cm, CH_2Cl_2/hexanes = 1/3) and Kugelrohr distillation (P = 0.15 mmHg, ABT = 240 °C) afforded **15d** as a colourless oil (43 mg, 12%, >99:1 dr).

Data for (6R^*,7S^*)-6,7-dichloro-9-methyldecyl benzoate (**15d**): HM-07-006

^{1}H NMR: (400 MHz, $CDCl_3$)

δ 8.06–8.04 (m, 2H), 7.58–7.54 (m, 1H), 7.46–7.43 (m, 2H), 4.34 (t, J = 6.6, 2H), 4.06–3.96 (m, 2H), 2.02–1.90 (m, 2H), 1.89–1.69 (m, 6H), 1.57–1.43 (m, 3H), 0.97 (d, J = 6.7, 3H), 0.91 (d, J = 6.4, 3H).

^{13}C NMR: (100 MHz, $CDCl_3$)

δ 166.8, 133.0, 130.6, 129.7, 128.5, 66.7, 65.0, 64.5, 43.8, 34.7, 28.7, 26.1, 25.8, 25.3, 23.6, 20.9.

HRMS (ESI): $[M+Na]^+$ calcd for $C_{18}H_{26}{}^{35}Cl^{35}ClNaO_2$, $C_{18}H_{26}{}^{37}Cl^{35}ClNaO_2$, $C_{18}H_{26}{}^{37}Cl^{37}ClNaO_2$: 367.1202 (100.0%), 369.1176 (66.3%), 371.1155 (11.7%); found: 367.1201 (100.0%), 369.1172 (64.5%), 371.1146 (9.7%).

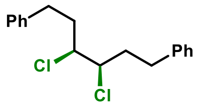

Flash column chromatography (SiO_2, ϕ = 4.0 cm, l = 14 cm, hexanes, R_f = 0.22 [254 nm/CAM (blue)], dry loaded, SiO_2 1.7 g) and Kugelrohr distillation (P = 0.15 mmHg, ABT = 300 °C) afforded **15e** as a white solid (135 mg, 44%, >99:1 dr).

Data for (3R,4S)-3,4-dichlorohexane-1,6-diyl)dibenzene (**15e**): HM-07-011

^{1}H NMR: (400 MHz, $CDCl_3$)

δ 7.33–7.29 (m, 4H), 7.24–7.16 (m, 6H), 3.99–3.94 (m, 2H), 2.93 (ddd, J = 13.7, 9.2, 4.6, 2H), 2.75–2.67 (m, 2H), 2.31–2.23 (m, 2H), 2.14–2.05 (m, 2H).

^{13}C NMR: (100 MHz, $CDCl_3$)

δ 140.7, 128.70, 128.68, 126.4, 65.1, 36.5, 32.4.

HRMS (ESI): $[M+H]^+$ calcd for $C_{18}H_{21}{}^{35}Cl^{35}Cl$, $C_{18}H_{21}{}^{37}Cl^{35}Cl$, $C_{18}H_{21}{}^{37}Cl^{37}Cl$: 307.1015 (100.0%), 309.0988 (65.8%), 311.0966 (11.4%); found: 307.1004 (100.0%), 309.0974 (70.4%), 311.0982 (10.0%).

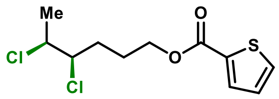

With 1.2 equiv of TTO and Tf_2O. Flash column chromatography (SiO_2, ϕ = 4.0 cm, l = 15 cm, CH_2Cl_2/hexanes = 1/2, R_f = 0.17 [254 nm/$KMnO_4$]) and Kugelrohr distillation (P = 0.15 mmHg, ABT = 240 °C) afforded **15f** as a colourless oil (147 mg, 52%, 97:3 dr).

Data for (4*R**,5*S**)-4,5-dichlorohexyl thiophene-2-carboxylate (**15f**): HM-08-071

^{1}H NMR: (400 MHz, $CDCl_3$)

δ 7.81 (dd, *J* = 3.7, 1.2, 1H), 7.56 (dd, *J* = 5.2, 1.2, 1H), 7.11 (dd, *J* = 5.2, 3.7, 1H), 4.39–4.30 (m, 2H), 4.11 (appr. pent, *J* = 6.6, 1H), 4.02–3.97 (m, 1H), 2.24–2.15 (m, 1H), 2.14–2.05 (m, 1H), 1.96–1.87 (m, 2H), 1.65 (d, *J* = 6.7, 3H).

1.60 (d, *J* = 6.7, 0.09H for the minor diastereomer).

^{13}C NMR: (100 MHz, $CDCl_3$)

δ 162.3, 133.8, 133.6, 132.6, 127.9, 66.7, 64.4, 60.2, 31.8, 25.6, 22.3.

HRMS (ESI): $[M+H]^+$ calcd for $C_{11}H_{15}{}^{35}Cl^{35}ClO_2S$ $C_{11}H_{15}{}^{37}Cl^{35}ClO_2S$ $C_{11}H_{15}{}^{37}Cl^{37}ClO_2S$: 281.0164 (100.0%), 283.0136 (69.6%), 285.0107 (13.8%); found: 281.0168 (100.0%), 283.0138 (68.5%), 285.0108 (10.0%).

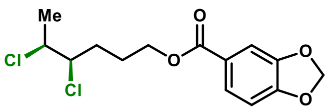

With 1.2 equiv of TTO and Tf_2O. Flash column chromatography (SiO_2, ϕ = 4.0 cm, l = 11 cm, CH_2Cl_2/hexanes = 1/2, R_f 0.19 [254 nm/$KMnO_4$]) and Kugelrohr distillation (P = 0.15 mmHg, ABT = 280 °C) afforded **15g** as a colourless oil (159 mg, 50%, 98:2 dr).

Data for (4*R**,5*S**)-4,5-dichlorohexyl benzo[d][1,3]dioxole-5-carboxylate (**15g**): HM-08-067

^{1}H NMR: (400 MHz, $CDCl_3$)

δ 7.65 (dd, *J* = 8.2, 1.5, 1H), 7.46 (d, *J* = 1.5, 1H), 6.84 (d, *J* = 8.2, 1H), 6.04 (s, 2H), 4.37–4.28 (m, 2H), 4.11 (appr. pent, *J* = 6.7, 1H), 4.01–3.96 (m, 1H), 2.24–2.15 (m, 1H), 2.14–2.04 (m, 1H), 1.95–1.86 (m, 2H), 1.64 (d, *J* = 6.4, 3H).

1.60 (d, *J* = 6.7, 0.06H for the minor diastereomer).

^{13}C NMR: (100 MHz, $CDCl_3$)

δ 166.0, 151.8, 147.9, 125.5, 124.3, 109.6, 108.1, 101.9, 66.7, 64.1, 60.2, 31.9, 25.7, 22.3.

HRMS (ESI): $[M+H]^+$ calcd for $C_{14}H_{17}{}^{35}Cl^{35}ClO_4$, $C_{14}H_{17}{}^{37}Cl^{35}ClO_4$, $C_{14}H_{17}{}^{37}Cl^{37}ClO_4$: 319.0498 (100.0%), 321.0471 (65.9%), 323.0449 (11.5%); found: 319.0510 (100.0%), 321.0481 (62.1%), 323.0455 (8.9%).

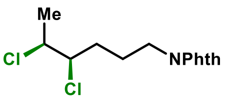

Flash column chromatography twice (SiO_2, ϕ = 4.0 cm, l = 20 cm, EtOAc/hexanes = 1/10, R_f = 0.24 [254 nm/CAM (blue)], dry loaded, SiO_2 2.0 g, then ϕ = 2.0 cm, l = 20 cm, EtOAc/hexanes = 1/10) and recrystallisation (CH_2Cl_2/pentane) afforded **15h** as a white solid (161 mg, 54%, >99:1 dr).

Data for 2-((4R^*,5S^*)-4,5-dichlorohexyl)isoindoline-1,3-dione (**15h**):[1] HM-06-081

^{1}H NMR: (400 MHz, $CDCl_3$)

δ 7.88–7.83 (m, 2H), 7.75–7.70 (m, 2H), 4.08 (appr. pent, *J* = 6.6, 1H), 4.01–3.97 (m, 1H), 3.78–3.72 (m, 2H), 2.05–1.99 (m, 2H), 1.85–1.80 (m, 2H), 1.61 (d, *J* = 6.4, 3H).

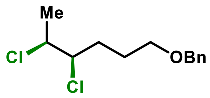

Thianthrenation for 1 minute. Flash column chromatography (SiO_2, ϕ = 4.0 cm, *l* = 15 cm, CH_2Cl_2/hexanes = 1/2, R_f = 0.20 [254 nm/$KMnO_4$]) and Kugelrohr distillation (P = 0.15 mmHg, ABT = 230 °C) afforded **15i** as a colourless oil (82 mg, 31%, 91:9 dr).

Data for ((((4R^*,5S^*)-4,5-dichlorohexyl)oxy)methyl)benzene (**15i**):[1] HM-08-073

^{1}H NMR: (400 MHz, $CDCl_3$)

δ 7.37–7.27 (m, 5H), 4.54–4.48 (m, 2H), 4.11 (appr. pent, *J* = 6.6, 1H), 4.00–3.95 (m, 1H), 3.96–3.47 (m, 2H), 2.19–2.12 (m, 1H), 1.98–1.89 (m, 1H), 1.86–1.70 (m, 2H), 1.62 (d, *J* = 6.4, 3H).

1.58 (d, *J* = 6.7, 0.30H for the minor diastereomer).

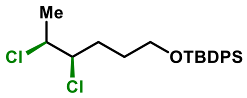

Flash column chromatography (SiO_2, ϕ = 4.0 cm, *l* = 15 cm, CH_2Cl_2/*n*-pentane = 1/10, R_f = 0.20 [254 nm/CAM(Blue)]) afforded **15j** as a colourless oil (170 mg, 42%, 94:6 dr).

Data for *tert*-butyl(((4R^*,5S^*)-4,5-dichlorohexyl)oxy)diphenylsilane (**15j**):[1] JGJ-09-074

^{1}H NMR: (400 MHz, $CDCl_3$)

δ 7.68–7.65 (m, 4H), 7.45–7.36 (m, 6H), 4.09 (appr. pent, *J* = 6.5, 1H), 3.98 (ddd, *J* = 9.5, 6.4, 3.1, 1H), 3.73–3.66 (m, 2H), 2.21–2.12 (m, 1H), 1.83–1.75 (m, 2H), 1.72–1.65 (m, 1H), 1.62 (d, *J* = 6.7, 3H), 1.05 (s, 9H).

1.57 (d, *J* = 6.4, 0.19H for the minor diastereomer).

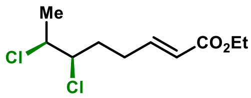

Flash column chromatography (SiO_2, ϕ = 5.0 cm, *l* = 14 cm, EtOAc/hexanes = 1/24, R_f = 0.14 [254 nm/CAM (blue)], dry loaded, SiO_2 2.1 g) and Kugelrohr distillation (P = 40 mmHg, ABT = 250 °C) afforded **15k** as a colourless oil (130 mg, 54%, 97:3 dr).

Data for ethyl (6R^*,7S^*,*E*)-6,7-dichlorooct-2-enoate (**15k**): HM-06-061

^{1}H NMR: (400 MHz, $CDCl_3$)

δ 6.93 (ddd, *J* = 15.6, 7.3, 6.7, 1H), 5.89 (d, *J* = 15.6, 1H), 4.19 (q, *J* = 7.1, 2H), 4.09 (appr. pent, 6.6, 1H), 3.90 (ddd, *J* = 9.9, 7.0, 2.8, 1H), 2.57–2.48 (m, 1H), 2.42–2.32 (m, 1H), 2.23–2.15 (dddd, *J* = 16.5, 9.2, 7.0, 2.4, 1H).

1.59 (d, *J* = 6.7, 0.11H for the minor diastereomer).

^{13}C NMR: (100 MHz, $CDCl_3$)

δ 166.5, 146.8, 122.8, 66.2, 60.5, 60.2, 33.4, 28.8, 22.4, 14.4.

HRMS (EI): $[M]^+$ calcd for $C_{10}H_{16}{}^{35}Cl^{35}ClO_2$, $C_{10}H_{16}{}^{37}Cl^{35}ClO_2$, $C_{10}H_{16}{}^{37}Cl^{37}ClO_2$: 238.0531 (100.0%), 240.0507 (58.5%), 242.0487 (11.5%); found: 238.0527 (100.0%), 240.0499 (64.9%), 242.0473 (10.8%).

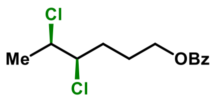

Chlorination for 5 hours. Flash column chromatography (SiO_2, ϕ = 4.5 cm, l = 13 cm, EtOAc/hexanes = 1/30, R_f = 0.34 [254 nm/CAM (blue)], dry loaded, SiO_2 2.2 g) and Kugelrohr distillation (P = 0.15 mmHg, ABT = 220 °C) afforded **15l** as a colourless oil (121 mg, 44%, >99:1 dr).

Data for (4*R**,5*R**)-4,5-dichlorohexyl benzoate (**15l**):[2] HM-06-066

^{1}H NMR: (400 MHz, $CDCl_3$)

δ 8.06–8.04 (m, 2H), 7.59–7.55 (m, 1H), 7.47–7.43 (m, 2H), 4.43–4.33 (m, 2H), 4.28 (qd, *J* = 6.7, 3.1, 1H), 4.09 (dt, *J* = 9.8, 2.8, 1H), 2.18–2.07 (m, 2H), 1.97–1.85 (m, 2H), 1.60 (d, *J* = 6.9, 3H).

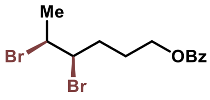

Flash column chromatography twice (SiO_2, ϕ = 4.0 cm, l = 12 cm, CH_2Cl_2/hexanes = 1/2, R_f = 0.31 [254 nm], then ϕ = 4.0 cm, l = 12 cm, CH_2Cl_2/*n*-pentane = 1/2) afforded **16a** as a colourless oil (257 mg, 71%, 83:17 dr).

Data for (4*R**,5*S**)-4,5-dibromohexyl benzoate (**16a**): JGJ-08-081

^{1}H NMR: (400 MHz, $CDCl_3$)

δ 8.07–8.02 (m, 2H), 7.59–7.54 (m, 1H), 7.47–7.41 (m, 2H), 4.38 (appr. t, *J* = 6.2, 2H), 4.29–4.14 (m, 2H), 2.41–2.27 (m, 1H), 2.18–2.02 (m, 2H), 2.00–1.92 (m, 1H), 1.89 (d, *J* = 6.4, 3H).

1.79 (d, *J* = 6.7, 0.63H for the minor diastereomer (i.e., **16l**)).

^{13}C NMR: (100 MHz, $CDCl_3$)

δ 166.7, 133.1, 130.3, 129.7, 128.5, 64.1, 60.6, 52.2, 34.1, 26.6, 25.5.

HRMS (ESI): $[M+Na]^+$ calcd for $C_{13}H_{16}{}^{79}Br^{79}BrNaO_2$, $C_{13}H_{16}{}^{81}Br^{79}BrNaO_2$, $C_{13}H_{16}{}^{81}Br^{81}BrNaO_2$: 384.9409 (51.0%), 386.9389 (100.0%), 388.9371 (49.7%); found: 384.9398 (49.4%), 386.9378 (100.0%), 388.9359 (46.7%).

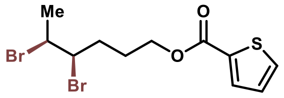

Flash column chromatography twice (SiO_2, ϕ = 4.0 cm, l = 12 cm, CH_2Cl_2/*n*-pentane = 1/2, R_f = 0.30 [254 nm]) afforded **16f** as a colourless oil (232 mg, 63%, 77:23 dr).

Data for (4*R**,5*S**)-4,5-dibromohexyl thiophene-2-carboxylate (**16f**): JGJ-08-098

^{1}H NMR: (400 MHz, $CDCl_3$)

δ 7.81 (dd, *J* = 3.7, 1.2, 1H), 7.56 (dd, *J* = 4.9, 1.5, 1H), 7.11 (dd, *J* = 5.2, 4.0, 1H), 4.39–4.31 (m, 2H), 4.29–4.13 (m, 2H), 2.39–2.25 (m, 1H), 2.15–1.99 (m, 2H), 1.95–1.90 (m, 1H), 1.89 (d, *J* = 6.4, 3H).

1.79 (d, *J* = 6.7, 0.91H for the minor diastereomer).

^{13}C NMR: (100 MHz, $CDCl_3$)

δ 162.3, 133.8, 133.6, 132.6, 127.9, 64.2, 60.5, 52.2, 34.0, 26.5, 25.4.

HRMS (ESI): $[M+Na]^+$ calcd for $C_{11}H_{14}{}^{79}Br^{79}BrNaO_2S$, $C_{11}H_{14}{}^{81}Br^{79}BrNaO_2S$, $C_{11}H_{14}{}^{81}Br^{81}BrNaO_2S$: 390.8973 (49.9%), 392.8953 (100.0%), 394.8932 (52.8%); found: 390.8962 (48.0%), 392.8942 (100.0%), 394.8921 (51.7%).

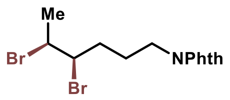

Flash column chromatography (SiO$_2$, ϕ = 4.0 cm, l = 14 cm, CH_2Cl_2/*n*-pentane = 1/2, R_f = 0.24 [254 nm/CAM (blue)]) afforded **16h** as a white solid (271 mg, 70%, 85:15 dr).

Data for 2-((4*R**,5*S**)-4,5-dibromohexyl)isoindoline-1,3-dione (**16h**): JGJ-08-094

^{1}H NMR: (400 MHz, $CDCl_3$)
δ 7.87–7.83 (m, 2H), 7.74–7.71 (m, 2H), 4.26–4.12 (m, 2H), 3.74 (appr. t, J = 6.9, 2H), 2.23–2.14 (m, 1H), 2.10–1.90 (m, 2H), 1.95–1.86 (m, 3H), 1.85 (d, J = 6.4, 3H).
1.77 (d, J = 6.7, 0.51H for the minor diastereomer).

^{13}C NMR: (100 MHz, $CDCl_3$)
δ 168.5, 134.2, 132.2, 123.4, 60.4, 52.2, 37.2, 34.4, 26.4, 25.3.

HRMS (ESI): [M+Na]$^+$ calcd for $C_{14}H_{15}{}^{79}Br^{79}BrNNaO_4$, $C_{14}H_{15}{}^{81}Br^{79}BrNNaO_4$, $C_{14}H_{15}{}^{81}Br^{81}BrNNaO_4$: 409.9362 (51.0%), 411.9342 (100.0%), 413.9323 (49.8%); found: 409.9358 (49.0%), 411.9339 (100.0%), 413.9319 (48.4%).

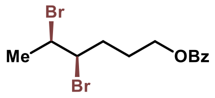

Flash column chromatography twice (SiO$_2$, ϕ = 4.0 cm, l = 15 cm, CH_2Cl_2/hexanes = 1/2 then ϕ = 4.0 cm, l = 15 cm, CH_2Cl_2/*n*-pentane = 1/2, R_f = 0.34 [254 nm]) afforded **16l** as a colourless oil (258 mg, 71%, 44:56 dr).

Data for (4*R**,5*R**)-4,5-dibromohexyl benzoate (**16l**): JGJ-08-087

^{1}H NMR: (400 MHz, $CDCl_3$)
δ 8.07–8.03 (m, 1H), 7.59–7.55 (m, 1H), 7.47–7.42 (m, 1H), 4.47 (qd, J = 6.7, 3.0, 1H), 4.30–4.13 (m, 2H), 4.20–4.14 (m, 1H), 2.41–2.27 (m, 1H), 2.18–1.91 (m, 3H), 1.79 (d, J = 6.7, 3H).
1.89 (d, J = 6.4, 3.9, 3.9H for the other diastereomer (i.e., **16a**)).

^{13}C NMR: (100 MHz, $CDCl_3$)
δ 166.7, 133.1, 130.3, 129.7, 128.5, 64.0, 59.2, 52.2, 31.0, 27.4, 21.6.

HRMS (ESI): [M+Na]$^+$ calcd for $C_{13}H_{16}{}^{79}Br^{79}BrNaO_2$, $C_{13}H_{16}{}^{81}Br^{79}BrNaO_2$, $C_{13}H_{16}{}^{81}Br^{81}BrNaO_2$,: 384.9409 (51.0%), 386.9389 (100.0%), 388.9371 (49.7%); found: 384.9408 (46.6%), 386.9389 (100.0%), 388.9373 (48.8%).

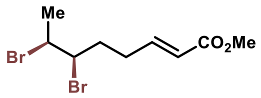

Flash column chromatography (SiO$_2$, ϕ = 3.5 cm, l = 12 cm, CH_2Cl_2/*n*-pentane = 1/2, R_f = 0.24 [254 nm/CAM (blue)]) afforded **16m** as a colourless oil (229 mg, 73%, 82:18 dr).

Data for methyl (6*R**,7*S**,*E*)-6,7-dibromooct-2-enoate (**16m**): JGJ-08-096

^{1}H NMR: (400 MHz, $CDCl_3$)
δ 6.93 (appr. dt, J = 15.6, 6.9, 1H), 5.91 (d, J = 15.6, 1H), 4.25–4.14 (m, 2H), 4.08–4.03 (m, 1H), 3.74 (s, 3H), 2.60–2.48 (m, 1H), 2.45–2.27 (m, 2H), 2.09–1.96 (m, 1H), 1.88 (d, J = 6.4, 3H).
1.78 (d, J = 6.7, 0.71H for the minor diastereomer).

^{13}C NMR: (100 MHz, $CDCl_3$)
δ 166.9, 147.0, 122.4, 60.0, 52.2, 51.7, 35.6, 29.9, 25.4.

HRMS (ESI): [M+Na]$^+$ calcd for $C_9H_{14}{}^{79}Br^{79}BrNaO_2$, $C_9H_{14}{}^{81}Br^{79}BrNaO_2$, $C_9H_{14}{}^{81}Br^{81}BrNaO_2$: 334.9253 (51.2%), 336.9233 (100.0%), 338.9213 (49.3%); found: 334.9255 (49.5%), 336.9235 (100.0%), 338.9215 (46.4%).

2.5. General Procedure II: *syn*-Bromochlorination of Alkenes

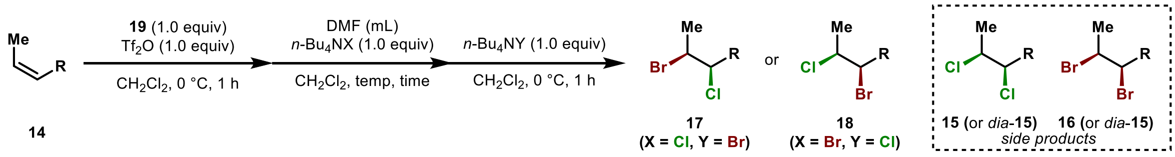

To a stirred solution of alkene (**14**, 1.00 mmol, 1.0 equiv) and thianthrene-*S*-oxide (**19**, 232 mg, 1.00 mmol, 1.0 equiv) in CH_2Cl_2 (4 mL) was added Tf_2O (168 µL, 1.00 mmol, 1.0 equiv) at 0 °C. After 1 hour, the reaction mixture was cooled to a lower temperature (indicated for each substrate), and DMF was added in some cases (indicated for each substrate). Then, a solution of *n*-Bu_4NX (1.00 mmol, 1.0 equiv) in CH_2Cl_2 (2.5 mL) was added. After a given period (indicated for each substrate), the reaction mixture was warmed up to 0 °C. Then, a solution of *n*-Bu_4NY (1.0 mmol, 1.0 equiv) in CH_2Cl_2 (2.5 mL) was added. After 1 hour, H_2O (10 mL) was added, and the organic layer was separated. The aqueous layer was extracted with CH_2Cl_2 (10 mL × 3), and the combined organic extracts were dried over $MgSO_4$ (2 g), filtered through a glass frit, and concentrated in vacuo. The residue was diluted with Et_2O (5 mL), filtered through a pad of SiO_2 (ϕ = 2.0 cm, l = 4.0 cm, Et_2O, 150 mL), and concentrated in vacuo. The crude material was purified by flash column chromatography through SiO_2 to afford **17** or **18**. When necessary, further purification by Kugelrohr distillation or recrystallisation was conducted. The products were obtained as a mixture with the diastereomer, the constitutional isomer, and the homodihalides. The calculated yields of **17** or **18** are given in the parenthesis.

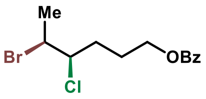

X = Cl, Y = Br, –30 °C, 24 hours. Flash column chromatography (SiO_2, ϕ = 4.0 cm, l = 11 cm, CH_2Cl_2/hexanes = 1/3, R_f = 0.30 [254 nm/CAM (blue)]) and Kugelrohr distillation (P = 0.15 mmHg, ABT = 230 °C) afforded **17a** (220 mg, 69% (59% **17a**), 99:1 dr, 90:10 rr) as a colourless oil.

Data for (4*R**,5*S**)-5-bromo-4-chlorohexyl benzoate (**17a**): HM-07-099

^{1}H NMR: (400 MHz, $CDCl_3$)
δ 8.06–8.03 (m, 2H), 7.59–7.52 (m, 1H), 7.47–7.43 (m, 2H), 4.39–4.36 (m, 2H), 4.23–4.16 (m, 1H), 4.08–4.04 (m, 1H), 2.32–2.23 (m, 1H), 2.14–2.05 (m, 1H), 2.00–1.88 (m, 2H), 1.84 (d, *J* = 6.7, 3H).
1.79 (d, *J* = 6.7, 0.057H, the dibromide *dia*-**16a** (**16l**)).
1.76 (d, *J* = 6.7, 0.030H, the minor diastereomer of **17a**).
1.70 (d, *J* = 6.4, 0.340H, the constitutional isomer **18a**).
1.60 (d, *J* = 6.7, 0.050H, the dichloride *dia*-**15a** (**15l**)).

^{13}C NMR: (100 MHz, $CDCl_3$)
δ 166.7, 133.1, 130.3, 129.7, 128.5, 66.9, 64.2, 52.1, 33.0, 25.7, 23.6.

HRMS (ESI): [M+H]+ calcd for $C_{13}H_{17}{}^{79}Br^{35}ClO_2$, [$C_{13}H_{17}{}^{81}Br^{35}ClO_2$ + $C_{13}H_{17}{}^{79}Br^{37}ClO_2$], $C_{13}H_{17}{}^{81}Br^{37}ClO_2$: 319.0095 (76.5%), 321.0073 (100.0%), 323.0050 (25.2%); found: 319.0108 (76.1%), 321.0086 (100.0%), 323.0059 (23.2%).

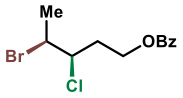

X = Cl, Y = Br, –40 °C, 5 hours, DMF (0.3 mL). Flash column chromatography (SiO_2, ϕ = 3.0 cm, l = 14 cm, CH_2Cl_2/hexanes = 1/3, R_f = 0.25 [254 nm/CAM (blue)]) and Kugelrohr distillation (P = 0.15 mmHg, ABT = 230 °C) afforded **17b** (201 mg, 66% (59% **17b**), >99:1 dr, 96:4 rr) as a colourless oil.

Data for (3*R**,4*S**)-4-bromo-3-chloropentyl benzoate (**17b**): HM-07-085

^{1}H NMR: (400 MHz, $CDCl_3$)

δ 8.06–8.04 (m, 2H), 7.60–7.56 (m, 1H), 7.48–7.44 (m, 2H), 4.62–4.57 (m, 1H), 4.54–4.47 (m, 1H), 4.25 (appr. pent, J = 6.6, 1H), 4.19 (ddd, J = 9.8, 7.0, 2.4, 1H), 2.64 (dddd, J = 15.0, 8.9, 6.1, 2.8, 1H), 2.21–2.12 (m, 1H), 1.86 (d, J = 6.4, 3H).

1.80 (d, J = 6.7, 0.0083H, the diastereomer of **17b**).

1.70 (d, J = 6.1, 0.12H, the constitutional isomer **18b**).

1.67 (d, J = 6.4, 0.20H, the dichloride **15b**).

^{13}C NMR: (100 MHz, $CDCl_3$)

δ 166.5, 133.3, 130.1, 129.7, 128.6, 63.8, 61.8, 52.1, 35.4, 23.6.

HRMS (ESI): [M+H]+ calcd for $C_{12}H_{15}{}^{79}Br^{35}ClO_2$, [$C_{12}H_{15}{}^{81}Br^{35}ClO_2$ + $C_{12}H_{15}{}^{79}Br^{37}ClO_2$], $C_{12}H_{15}{}^{81}Br^{37}ClO_2$: 304.9938 (76.6%), 306.9917 (100.0%), 308.9893 (25.1%); found: 304.9932 (74.8%), 306.9911 (100.0%), 308.9885 (21.5%).

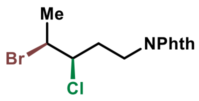

X = Cl, Y = Br, –40 °C, 24 hours, DMF (0.3 mL). Flash column chromatography twice (SiO_2, ϕ = 3.5 cm, l = 12 cm, CH_2Cl_2/hexanes = 1/1, R_f = 0.22 [254 nm/$KMnO_4$]), then ϕ = 4.0 cm, l = 13 cm, CH_2Cl_2/hexanes = 1/2) afforded **17n** (238 mg, 72% (66% **17n**), >99:1 dr, 96:4 dr) as a white solid.

Data for 2-((3*R**,4*S**)-4-bromo-3-chloropentyl)isoindoline-1,3-dione (**17n**): HM-08-024

^{1}H NMR: (400 MHz, $CDCl_3$)

δ 7.87–7.85 (m, 2H), 7.74–7.72 (m, 2H), 4.21 (appr. pent, J = 6.8, 1H), 4.05–3.85 (m, 3H), 2.57–2.50 (m, 1H), 2.19–2.10 (m, 1H), 1.81 (d, J = 6.7, 3H).

1.67 (d, J = 6.4, 0.13H, the constitutional isomer **18n**).

1.62 (d, J = 6.4, 0.15H, the dichloride **15n**).

^{13}C NMR: (100 MHz, $CDCl_3$)

δ 168.4, 134.2, 132.2, 123.5, 64.5, 51.7, 35.6, 35.1, 23.4.

HRMS (ESI): [M+H]+ calcd for $C_{13}H_{14}{}^{79}Br^{35}ClNO_2$, [$C_{13}H_{14}{}^{81}Br^{35}ClNO_2$ + $C_{13}H_{14}{}^{79}Br^{37}ClNO_2$], $C_{13}H_{14}{}^{81}Br^{37}ClNO_2$: 329.9891 (76.5%), 331.9869 (100.0%), 333.9846 (25.2%); found: 329.9888 (75.4%), 331.9866 (100.0%), 333.9839 (22.7%).

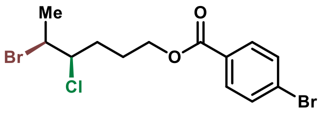

X = Cl, Y = Br, –50 °C, 24 hours, DMF (0.5 mL). Flash column chromatography (SiO_2, ϕ = 4.5 cm, l = 13 cm, CH_2Cl_2/hexanes = 1/2, R_f = 0.28 [254 nm/CAM (blue)]) afforded **17o** (268 mg, 67% (58% **17o**), 99:1 dr, 92:8 rr) as a white solid.

Data for (4R^*,5S^*)-5-bromo-4-chlorohexyl 4-bromobenzoate (**17o**): HM-08-016

^{1}H NMR: (400 MHz, $CDCl_3$)
δ 7.92–7.88 (m, 2H), 7.60–7.57 (m, 2H), 4.38–4.34 (m, 2H), 4.18 (appr. pent, J = 6.6, 1H), 4.06–4.02 (m, 1H), 2.30–2.22 (m, 1H), 2.15–2.04 (m, 1H), 1.97–1.88 (m, 2H), 1.84 (d, J = 6.7, 3H).
1.70 (d, J = 6.4, 0.26H, the constitutional isomer **18o**).
1.65 (d, J = 6.7, 0.17H, the dichloride **15o**).

^{13}C NMR: (100 MHz, $CDCl_3$)
δ 166.0, 131.9, 131.2, 129.2, 128.3, 66.8, 64.5, 52.0, 33.0, 25.6, 23.7.

HRMS (ESI): $[M+H]^+$ calcd for $C_{13}H_{16}{}^{79}Br^{79}Br^{35}ClO_2$, [$C_{13}H_{16}{}^{81}Br^{79}Br^{35}ClO_2$ + $C_{13}H_{16}{}^{79}Br^{79}Br^{37}ClO_2$], [$C_{13}H_{16}{}^{81}Br^{81}Br^{35}ClO_2$ + $C_{13}H_{16}{}^{81}Br^{79}Br^{37}ClO_2$], $C_{13}H_{16}{}^{81}Br^{81}Br^{37}ClO_2$: 396.9200 (43.9%), 398.9179 (100.0%), 400.9157 (70.2%), 402.9135 (14.2%); found: 396.9211 (38.0%), 398.9190 (100.0%), 400.9168 (65.3%), 402.9144 (10.1%).

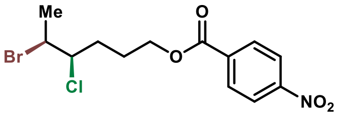

X = Cl, Y = Br, –50 °C, 24 hours, DMF (0.3 mL). Flash column chromatography (SiO_2, ϕ = 4.0 cm, l = 13 cm, CH_2Cl_2/hexanes = 1/2, R_f = 0.27 [254 nm/CAM (blue)]) afforded **17p** (221 mg, 61% (52% **17p**), >99:1 dr, 91:9 rr) as a colourless crystalline solid.

Data for (4R^*,5S^*)-5-bromo-4-chlorohexyl 4-nitrobenzoate (**17p**): HM-08-006

mp: 63.6–64.6 °C

^{1}H NMR: (400 MHz, $CDCl_3$)
δ 8.31–8.29 (m, 2H), 8.23–8.20 (m, 2H), 4.43 (t, J = 6.1, 2H), 4.22–4.14 (m, 1H), 4.07–4.02 (m, 1H), 2.33–2.26 (m, 1H), 2.18–2.09 (m, 1H), 2.01–1.94 (m, 2H), 1.85 (d, J = 6.4, 3H).
1.71 (d, J = 6.4, 0.28H, the constitutional isomer **18p**).
1.66 (d, J = 6.4, 0.20H, the dichloride **15p**).

^{13}C NMR: (100 MHz, $CDCl_3$)
δ 164.8, 150.7, 135.7, 130.9, 123.7, 2266.7, 65.2, 52.0, 33.0, 25.5, 23.9.

HRMS (ESI): $[M+H]^+$ calcd for $C_{13}H_{16}{}^{79}Br^{35}ClNO_4$, [$C_{13}H_{16}{}^{81}Br^{35}ClNO_4$ + $C_{13}H_{16}{}^{79}Br^{37}ClNO_4$], $C_{13}H_{16}{}^{81}Br^{37}ClNO_4$: 363.9946 (76.3%), 365.9924 (100.0%), 367.9902 (25.6%); found: 363.9900 (79.2%), 365.9918 (100.0%), 367.9884 (20.7%).

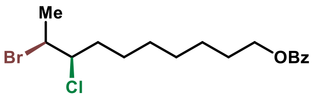

X = Cl, Y = Br, –30 °C, 24 hours. Flash column chromatography (SiO_2, ϕ = 5.0 cm, l = 12.0 cm, CH_2Cl_2/hexanes = 1/4, R_f = 0.19 [254 nm/CAM (blue)]) and Kugelrohr distillation (P = 0.15 mmHg, ABT = 300 °C) afforded **17q** (232 mg, (62% **17q)**, ca. 84:16 rr) as a pale yellow oil.

Data for (8*R**,9*S**)-9-bromo-8-chlorodecyl benzoate (**17q**): HM-09-036

^{1}H NMR: (400 MHz, $CDCl_3$)

δ 8.06–8.03 (m, 2H), 7.58–7.54 (m, 1H), 7.46–7.42 (m, 2H), 4.32 (t, *J* = 6.7, 2H), 4.18 (appr. pent, *J* = 6.7, 1H), 3.99 (ddd, *J* = 9.5, 7.0, 3.1, 1H), 2.07–1.98 (m, 1H), 1.81–1.74 (m, 6H), 1.63–1.58 (m, 1H), 1.48–1.32 (m, 7H).

1.86 (d, *J* = 6.4, 0.76H, the dibromide **16q**).

1.66 (d, *J* = 6.4, 0.58H, the constitutional isomer **18q**).

^{13}C NMR: (100 MHz, $CDCl_3$)

δ 166.8, 133.0, 130.6, 129.7, 128.5, 67.7, 65.2, 52.4, 36.2, 29.2, 29.0, 28.8, 26.14, 26.08, 23.4.

HRMS (ESI): $[M+Na]^+$ calcd for $C_{17}H_{24}{}^{79}Br^{35}ClNaO_2$, $[C_{17}H_{24}{}^{81}Br^{35}ClNaO_2 + C_{17}H_{24}{}^{79}Br^{37}ClNaO_2]$, $C_{17}H_{24}{}^{81}Br^{37}ClNaO_2$: 397.0540 (76.1%), 399.0519 (100.0%), 401.0498 (25.7%); found: 397.0549 (79.3%), 399.0524 (100.0%), 401.0503 (23.5%).

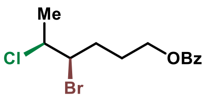

X = Br, Y = Cl, –30 °C, 22 hours. Flash column chromatography twice (SiO_2, ϕ = 4.0 cm, *l* = 12 cm, CH_2Cl_2/hexanes = 1/2 then ϕ = 3.0 cm, *l* = 14 cm, CH_2Cl_2/*n*-pentane = 1/2, R_f = 0.28 [CAM (blue)]) afforded **18a** (172 mg, 54% (38% **18a**), 99:1 dr, 90:10 rr) as a colourless oil.

Data for (4*R**,5*S**)-4-bromo-5-chlorohexyl benzoate (**18a**): JGJ-08-057

^{1}H NMR: (400 MHz, $CDCl_3$)

δ 8.06–8.03 (m, 2H), 7.59–7.54 (m, 1H), 7.47–7.42 (m, 2H), 4.41–4.33 (m, 2H), 4.20–4.06 (m, 2H), 2.34–2.24 (m, 1H), 2.18–2.10 (m, 1H), 2.09–1.91 (m, 2H), 1.70 (d, *J* = 6.1, 3H).

1.89 (d, *J* = 6.7, 0.33H, the dibromide **16a**).

1.84 (d, *J* = 6.7, 0.31H, the constitutional isomer **17a**).

1.79 (d, *J* = 6.7, 0.49H, the dibromide *dia*-**16a**).

1.65 (d, *J* = 6.4, 0.089H, the dichloride **15a**).

1.63 (d, *J* = 6.7, 0.038H, the minor diastereomer of **18a**).

^{13}C NMR: (100 MHz, $CDCl_3$)

δ 166.7, 133.2, 130.3, 129.7, 128.6, 64.1, 60.4, 60.2, 32.7, 26.7, 23.8.

HRMS (ESI): $[M+Na]^+$ calcd for $C_{13}H_{16}{}^{79}Br^{35}ClNaO_2$, $[C_{13}H_{16}{}^{81}Br^{35}ClNaO_2 + C_{13}H_{16}{}^{79}Br^{37}ClNaO_2]$, $C_{13}H_{16}{}^{81}Br^{37}ClNaO_2$: 340.9914 (76.6%), 342.9893 (100.0%), 344.9869 (25.2%); found: 340.9916 (73.8%), 342.9894 (100.0%), 344.9867 (23.5%).

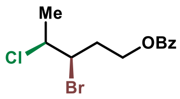

X = Br, Y = Cl, –30 °C, time = 17 hours. Flash column chromatography twice (SiO_2, ϕ = 5.0 cm, *l* = 10 cm, CH_2Cl_2/hexanes = 1/2 then ϕ = 3.5 cm, *l* = 12 cm, CH_2Cl_2/*n*-pentane = 1/2, R_f = 0.33 [254 nm/CAM (blue)]) afforded **18b** (197 mg, 64% (49% **18b**), >99:1 dr, 95:5 rr) as a colourless oil.

Data for (3*R**,4*S**)-3-bromo-4-chloropentyl benzoate (**18b**): JGJ-08-066

^{1}H NMR: (400 MHz, $CDCl_3$)

δ 8.09–8.01 (m, 2H), 7.60–7.55 (m, 1H), 7.49–7.41 (m, 2H), 4.64–4.57 (m, 1H), 4.53–4.46 (m, 1H), 4.26–4.19 (m, 2H), 2.64 (dddd, *J* = 15.1, 8.9, 6.1, 2.4, 1H), 2.29–2.19 (m, 1H), 1.71 (d, *J* = 6.4, 3H).

1.91 (d, *J* = 6.1, 0.12H, the dibromide **16b**).

1.86 (d, *J* = 6.7, 0.18H, the constitutional isomer **17b**).

1.82 (d, *J* = 6.7, 0.31H, the dibromide *dia*-**16b**).

1.67 (d, *J* = 6.4, 0.37H, the dichloride **15b**).

^{13}C NMR: (100 MHz, $CDCl_3$)

δ 166.5, 133.3, 130.1, 129.7, 128.6, 62.7, 60.4, 56.7, 35.0, 23.7.

HRMS (ESI): $[M+H]^+$ calcd for $C_{12}H_{14}{}^{79}Br^{35}ClNaO_2$, $[C_{12}H_{14}{}^{81}Br^{35}ClNaO_2 + C_{12}H_{14}{}^{79}Br^{37}ClNaO_2]$, $C_{12}H_{14}{}^{81}Br^{37}ClNaO_2$: 326.9758 (76.6%), 328.9736 (100.0%), 330.9712 (25.1%); found: 326.9753 (73.8%), 328.9731 (100.0%), 330.9706 (23.2%).

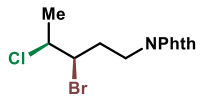

X = Br, Y = Cl, –30 °C, 23 hours. Flash column chromatography twice (SiO_2, ϕ = 4.0 cm, *l* = 15 cm, CH_2Cl_2/*n*-pentane = 1/1, R_f = 0.34 [254 nm/CAM (blue)]) afforded **18n** (175 mg, 53% (41% **18n**), >99:1 dr, 94:6 rr) as a white solid.

Data for 2-((3*R**,4*S**)-3-bromo-4-chloropentyl)isoindoline-1,3-dione (**18n**): JGJ-08-100

^{1}H NMR: (400 MHz, $CDCl_3$)

δ 7.88–7.84 (m, 2H), 7.75–7.71 (m, 2H), 4.17 (appr. pent, *J* = 6.6, 1H), 4.09–4.02 (m, 1H), 4.00–3.95 (m, 1H), 3.92–3.85 (m, 1H), 2.58–2.50 (m, 1H), 2.28–2.18 (m, 1H), 1.67 (d, *J* = 6.4, 3H).

1.86 (d, *J* = 6.7, 0.16H, the dibromide **16n**).

1.81 (d, *J* = 6.4, 0.21H, the constitutional isomer **17n**).

1.77 (d, *J* = 6.7, 0.24H, the dibromide *dia*-**16n**).

1.62 (d, *J* = 6.4, 0.25H, the dichloride **15n**).

^{13}C NMR: (100 MHz, $CDCl_3$)

δ 168.4, 134.2, 132.2, 123.5, 60.1, 57.2, 36.5, 34.7, 23.5.

HRMS (ESI): $[M+Na]^+$ calcd for $C_{13}H_{13}{}^{79}Br^{35}ClNNaO_2$, $[C_{13}H_{13}{}^{81}Br^{35}ClNNaO_2 + C_{13}H_{13}{}^{79}Br^{37}ClNNaO_2]$, $C_{13}H_{13}{}^{81}Br^{37}ClNNaO_2$: 351.9710 (76.5%), 353.9689 (100.0%), 355.9665 (25.2%); found: 351.9715 (77.8%), 353.9693 (100.0%), 355.9667 (22.6%).

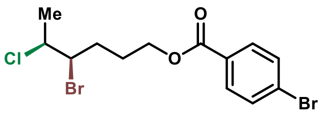

X = Br, Y = Cl, –30 °C, 16 hours. Flash column chromatography twice (SiO_2, ϕ = 4.0 cm, *l* = 10 cm, CH_2Cl_2/hexanes = 1/3, then ϕ = 4.0 cm, *l* = 12 cm, CH_2Cl_2/*n*-pentane = 1/3, R_f = 0.21 [254 nm]) afforded **18o** (151 mg, 38% (26% **18o**), 98:2 dr, 89:11 rr) as colourless oil.

Data for (4R^*,5S^*)-4-bromo-5-chlorohexyl 4-bromobenzoate (**18o**): JGJ-08-062

^{1}H NMR: (400 MHz, $CDCl_3$)

δ 7.93–7.86 (m, 2H), 7.62–7.55 (m, 2H), 4.37 (appr. t, J = 6.1, 2H), 4.15 (appr. pent, J = 6.6, 1H), 4.08 (appr. td, J = 8.8, 2.6, 1H), 2.31–2.23 (m, 1H), 2.17–2.09 (m, 1H), 2.06–1.90 (m, 2H), 1.84 (d, J = 6.4, 3H).

1.89 (d, J = 6.7, 0.49H, the dibromide **16o**).

1.84 (d, J = 6.4, 0.38H, the constitutional isomer **17o**).

1.79 (d, J = 6.7, 0.38H, the dibromide *dia*-**16o**).

1.65 (d, J = 6.7, 0.15H, the dichloride **15o**).

1.63 (d, J = 6.4, 0.043H, the constitutional isomer *dia*-**17o**).

^{13}C NMR: (100 MHz, $CDCl_3$)

δ 166.0, 131.9, 131.3, 129.2, 128.3, 64.4, 60.2, 32.6, 26.6, 23.9.

HRMS (ESI): $[M+H]^+$ calcd for $C_{13}H_{15}{}^{79}Br^{79}Br^{35}ClNaO_2$, $[C_{13}H_{15}{}^{81}Br^{79}Br^{35}ClNaO_2 + C_{13}H_{15}{}^{79}Br^{79}Br^{37}ClNaO_2]$, $[C_{13}H_{15}{}^{81}Br^{81}Br^{35}ClNaO_2 + C_{13}H_{15}{}^{81}Br^{79}Br^{37}ClNaO_2]$, $C_{13}H_{15}{}^{81}Br^{81}Br^{37}ClNaO_2$: 418.9020 (43.9%), 420.8998 (100.0%), 422.8977 (70.2%), 424.8955 (14.2%); found: 418.9002 (41.3%), 420.8980 (100.0%), 422.8960 (66.4%), 424.8935 (12.7%).

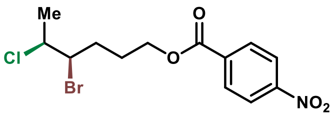

X = Br, Y = Cl, –30 °C, 25 hours. Flash column chromatography (SiO_2, ϕ = 4.0 cm, l = 13 cm, CH_2Cl_2/*n*-pentane = 1/2, R_f = 0.16 [254 nm]) afforded **18p** (192 mg, 52% (34% **18p**), 99:1 dr, 88:12 rr) as a yellow solid.

Data for (4R^*,5S^*)-4-bromo-5-chlorohexyl 4-nitrobenzoate (**18p**): JGJ-08-099

^{1}H NMR: (400 MHz, $CDCl_3$)

δ 8.33–8.28 (m, 2H), 8.23–8.19 (m, 2H), 4.43 (appr. t, J = 6.1, 2H), 4.20–4.12 (m, 1H), 4.11–4.04 (m, 2H), 2.33–2.26 (m, 1H), 2.20–2.11 (m, 1H), 2.09–1.95 (m, 2H), 1.71 (d, J = 6.4, 3H).

1.91 (d, J = 6.4, 0.45H, the dibromide **16p**).

1.85 (d, J = 6.4, 0.41H, the constitutional isomer **17p**).

1.79 (d, J = 6.7, 0.45H, the dibromide *dia*-**16p**).

1.77 (d, J = 7.0, 0.10H, the constitutional isomer *dia*-**17p**).

1.66 (d, J = 6.4, 0.21H, the dichloride **15p**).

1.61 (d, J = 6.7, 0.043H, the minor diastereomer of **18p**).

^{13}C NMR: (100 MHz, $CDCl_3$)

δ 164.8, 150.7, 135.7, 130.9, 123.8, 65.1, 60.2, 60.0, 32.6, 26.5, 24.0.

HRMS (ESI): $[M+H]^+$ calcd for $C_{13}H_{15}{}^{79}Br^{35}ClNNaO_4$, $[C_{13}H_{15}{}^{81}Br^{35}ClNNaO_4 + C_{13}H_{15}{}^{79}Br^{37}ClNNaO_4]$, $C_{13}H_{15}{}^{81}Br^{37}ClNNaO_4$: 385.9765 (76.2%), 387.9744 (100.0%), 389.9721 (25.6%); found: 385.9761 (73.1%), 387.9739 (100.0%), 389.9714 (22.6%).

2.6. Solvent Survey for *syn*-Dichlorination of **14a**[a]

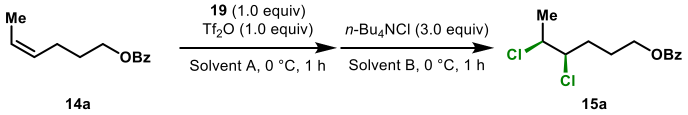

Entry	Solvent A	Solvent B	Yield (%)[b]	dr[c]
1	CH_2Cl_2	CH_2Cl_2	62	>99:1
2	$PhCF_3$	CH_2Cl_2	57	90:10
3	PhMe	CH_2Cl_2	19	89:11
4	Et_2O	CH_2Cl_2	57	95:5
5	1,2-DCE	1,2-DCE	50	>99:1
6	$CHCl_3$	$CHCl_3$	48	>99:1
7	MeCN	MeCN	46	94:6
8	HFIP	HFIP	2	>99:1

[a]All reactions were performed on 1.0 mmol scale at 0.25 M concentration. [b]Isolated yields after column chromatography. [c]Determined by ^1H NMR analysis of the purified materials.

2.7. Reaction Condition Optimisation for *syn*-Bromochlorination of **14a**[a]

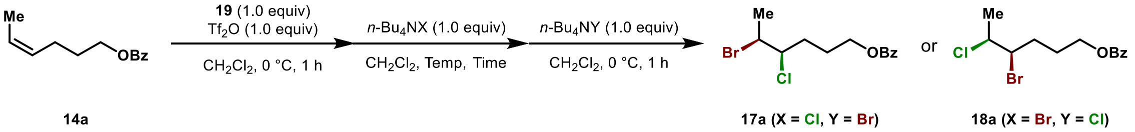

Entry	X	Y	Temp (°C)	Time (h)	Yield (%)[b]	rr[c]	dr[c]
1	Cl	Br	0	1	78	87:13	>99:1
2	Cl	Br	–30	1	84	90:10	>99:1
3	Cl	Br	–30	5	83	92:8	>99:1
4	Cl	Br	–40	41	79	91:9	95:5
5	Cl	Br	–50	24	81	89:11	97:3
6	Br	Cl	0	1	51	88:12	99:1
7	Br	Cl	–30	1	59	88:12	93:7
8	Br	Cl	–30	17	63	89:11	96:4
9[d]	Br	Cl	–50	18	63	93:7	97:3
10	Br	Cl	–50	72	55	92:8	97:3

[a]All reactions were performed on 1.0 mmol scale at 0.25 M concentration. [b]Isolated yields after column chromatography. [c]Determined by the ^1H NMR analysis of purified materials. [d]0.71 mmol scale.

2.8. Reaction Condition Optimisation for *syn*-Bromochlorination of **14p**[a]

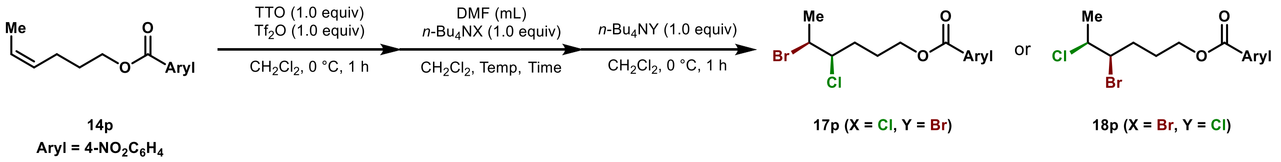

Entry	DMF (mL)	X	Y	Temp (°C)	Time (h)	Yield (%)[b]	rr[c]	dr[c]
1	–	Cl	Br	0	5	71	80:10	88:12
2	0.1	Cl	Br	0	5	69	87:13	96:4
3	1.0	Cl	Br	0	5	63	88:12	>99:1
4	0.1	Cl	Br	–50	68	62	85:15	95:5
5	0.3	Cl	Br	–50	22	73	91:9	>99:1
6	0.5	Cl	Br	–50	22	58	89:11	>99:1
7	1.0	Cl	Br	–50	5	68	90:10	>99:1
8	1.0	Cl	Br	–78	24	70	51:49	83:17
9	–	Br	Cl	0	1	73	88:12	86:14
10	–	Br	Cl	–30	19	69	90:10	96:4
11	–	Br	Cl	–50	48	50	79:21	79:21
12	0.1	Br	Cl	–50	45	80	69:31	63:37
13	1.0	Br	Cl	–50	13	35	62:38	96:4

[a]All reactions were performed on 1.0 mmol scale at 0.25 M concentration. [b]Isolated yields after column chromatography. [c]Determined by ^{1}H NMR analysis of the purified materials.

2.9. Mechanistic Validation of Reactive Species

Preparation of alkenyl TT^+

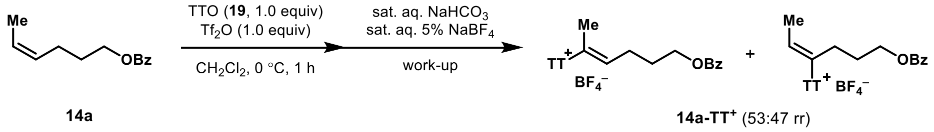

To a stirred solution of (*Z*)-hex-4-en-1-yl benzoate (**14a**, 409 mg, 2.00 mmol) and thianthrene-*S*-oxide (**19**, 465 mg, 2.00 mmol, 1.00 equiv) in CH_2Cl_2 (10 mL) was added Tf_2O (340 μL, 2.02 mmol, 1.01 equiv) at 0 °C. After 1 hour, sat. aq. $NaHCO_3$ (20 mL) was added, and the organic layer was separated. The aqueous layer was extracted with CH_2Cl_2 (10 mL × 2), and the combined organic extracts was washed with 5% aq. $NaBF_4$ (20 mL × 2), dried over $MgSO_4$ (2.7 g), filtered through a glass frit, and concentrated in vacuo. The crude material was purified by flash column chromatography (SiO_2, ϕ = 3.5 cm, l = 10 cm) eluting with *i*-PrOH/CH_2Cl_2 = 1/100 to 1/10 [254 nm/CAM (purple)] to afford **14a-TT**$^+$ as a yellow wax (740 mg, 74%, 53:47 rr).

Data for **14a-TT**$^+$: HM-09-094

^{1}H NMR: (400 MHz, $CDCl_3$)

δ 8.42–8.35 (m, 2H), 7.98–7.96 (m, 2H), 7.80–7.67 (m, 5H), 7.64–7.54 (m, 2H), 7.49–7.42 (m, 2H), 5.87–5.83 (m, 0.53H), 5.73 (q, *J* = 7.1, 0.47H), 4.23–4.20 (m, 2H), 2.43–2.31 (m, 2H), 1.88–1.67 (m, 5H).

^{13}C NMR: (100 MHz, $CDCl_3$)

δ 166.4, 166.2, 142.3, 138.5, 136.6, 136.1, 135.3, 135.0 (2C), 134.9, 133.3, 133.2, 130.2 (3C), 130.1, 129.9, 129.8, 129.5 (2C), 128.6, 128.5, 123.0, 121.7, 116.8, 116.5, 66.5, 63.2, 27.2, 26.9, 26.2, 24.7, 15.2, 13.8.

^{19}F NMR (376 MHz, $CDCl_3$)

δ –154.47, –154.51.

HRMS (ESI): $[M]^+$ calcd for $C_{25}H_{23}O_2S_2^+$: 419.1134; found: 419.1140.

Subjection of alkenyl TT^+ to the reaction of a different alkene

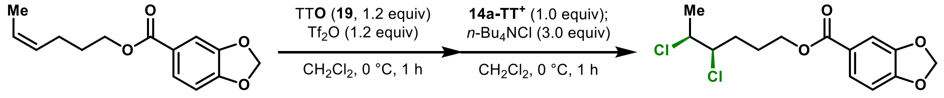

To a stirred solution of (*Z*)-hex-4-en-1-yl benzo[d][1,3]dioxole-5-carboxylate (**14g**, 248 mg, 1.00 mmol) and thianthrene-*S*-oxide (**19**, 279 mg, 1.20 mmol, 1.2 equiv) in CH_2Cl_2 (4 mL) was added Tf_2O (202 μL, 1.20 mmol, 1.2 equiv) at 0 °C. After 1 hour, a solution of **14a-TT**$^+$ (506 mg, 1.00 mmol, 1.0 equiv) in CH_2Cl_2 (2 mL) and a solution of *n*-Bu_4NCl (834 mg, 3.00 mmol, 3.0 equiv) in CH_2Cl_2 (8 mL) were added sequentially. After 1 hour, H_2O (10 mL) was added, and the organic layer was separated. The aqueous layer was extracted with CH_2Cl_2 (10 mL × 3), and the combined organic extracts were dried over $MgSO_4$ (2.7 g), filtered through a glass frit, and concentrated in vacuo. The residue was diluted with Et_2O (5 mL), filtered through a pad of SiO_2 (ϕ = 2.5 cm, *l* = 3.0 cm, Et_2O, 150 mL), and concentrated in vacuo. The crude material was purified by flash column chromatography (SiO_2, ϕ = 5.0 cm, *l* = 9.0 cm) eluting with CH_2Cl_2/hexanes = 1/2, (R_f = 0.19 [254 nm/$KMnO_4$]) to afford **15g** as a pale yellow oil (138 mg, 43%, 85:15 dr). **15a** was not detected.

3. Supplementary Figures

3.1. X-Ray Crystallographic Data

Crystals mounted on a diffractometer were analysed at 100 K (**17n**) or 294 K (**18n**). X-Ray Crystallographic Data Reflection data were collected using a Rigaku Oxford Diffraction XtaLAB Synergy-S diffractometer with $Cu_{K\alpha}$ radiation (λ = 1.54184 Å). The cell parameters were determined and refined using the CrysAlisPro program[12]. The compound structures were solved by direct methods and refined by full matrix least-squares using the Olex2[13] with anisotropic thermal parameters for all non-hydrogen atoms. The relevant data are summarised in below. CCDC 2262333 (**17n**) and 2262334 (**18n**) and contains the supplementary crystallographic data for this study. These data can be obtained free of charge from The Cambridge Crystallographic Data Centre via www.ccdc.cam.ac.uk/data_request/cif.

Crystallographic data and parameters for compounds **17n** and **18n**

	**17n**	**18n**
CCDC #	2262333	2262334
Empirical formula	$C_{13}H_{13}BrClNO_2$	$C_{13}H_{13}BrClNO_2$
Formula weight	330.61	330.61
Temperature/K	100.0	294.0
Crystal system	Triclinic	Triclinic
Space group	P–1	P–1
a/Å	5.73032(5)	5.8215(3)
b/Å	9.26726(8)	9.5907(4)
c/Å	12.76898(13)	12.7255(4)
α/°	84.2222(7)	84.423(3)
β/°	77.6359(8)	76.921(3)
γ/°	79.3387(7)	77.737(4)
Volume/$Å^3$	649.610(10)	675.37(5)
Z	2	2
$\rho_{calc}(g/cm^3)$	1.690	1.626
μ/mm^{-1}	6.157	5.922
F(000)	332.2	332.2
Crystal size/mm^3	0.17 × 0.14 × 0.13	0.29 × 0.06 × 0.05
Radiation	$Cu_{K\alpha}$ (λ = 1.54184 Å)	$Cu_{K\alpha}$ (λ = 1.54184 Å)
2Θ range for data collection/°	7.102 to 154.036	7.104 to 154.740
Index ranges	$-6 \le h \le 7, -11 \le k \le 11, -14 \le l \le 15$	$-6 \le h \le 7, -12 \le k \le 12, -16 \le l \le 15$
Reflections collected	16353	18745
Independent reflections	2557 [R_{int} = 0.0696, R_{sigma} = 0.0333]	2646 [R_{int} = 0.0442, R_{sigma} = 0.0227]
Data/restraints/parameters	2557/0/165	2646/0/164
Goodness-of-fit on F2	1.037	1.046
Final R indexes [I>=2σ (I)]	R_1 = 0.0263, wR_2 = 0.0668	R_1 = 0.0432, wR_2 = 0.1299
Final R indexes [all data]	R_1 = 0.0266, wR_2 = 0.0670	R_1 = 0.0460, wR_2 = 0.1326
Largest diff. peak/hole / e Å-3	0.48/–0.67	1.05/–0.55

$R_1 = \sum||Fo| - |Fc||/\sum|Fo|$. [b] $wR_2 = \{[\sum w(Fo^2 - Fc^2)^2]/[\sum w(Fo^2)^2]\}^{1/2}$.

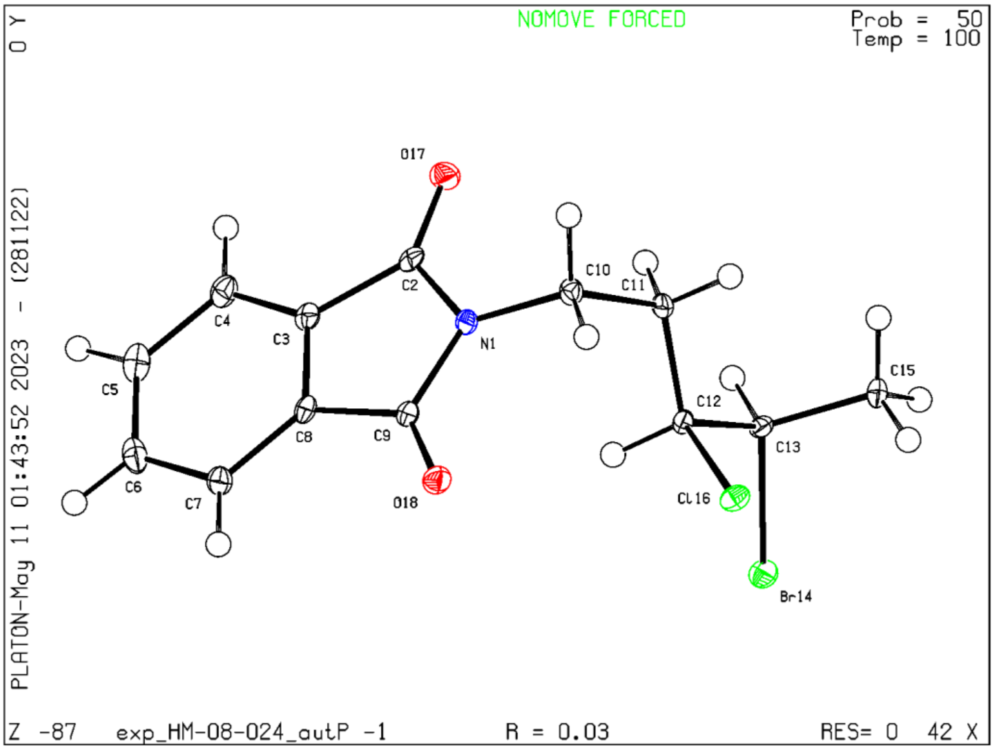

Supplementary Fig. 1. Thermal ellipsoid plot of **17n** at the 50% probability level, CCDC 2262333.

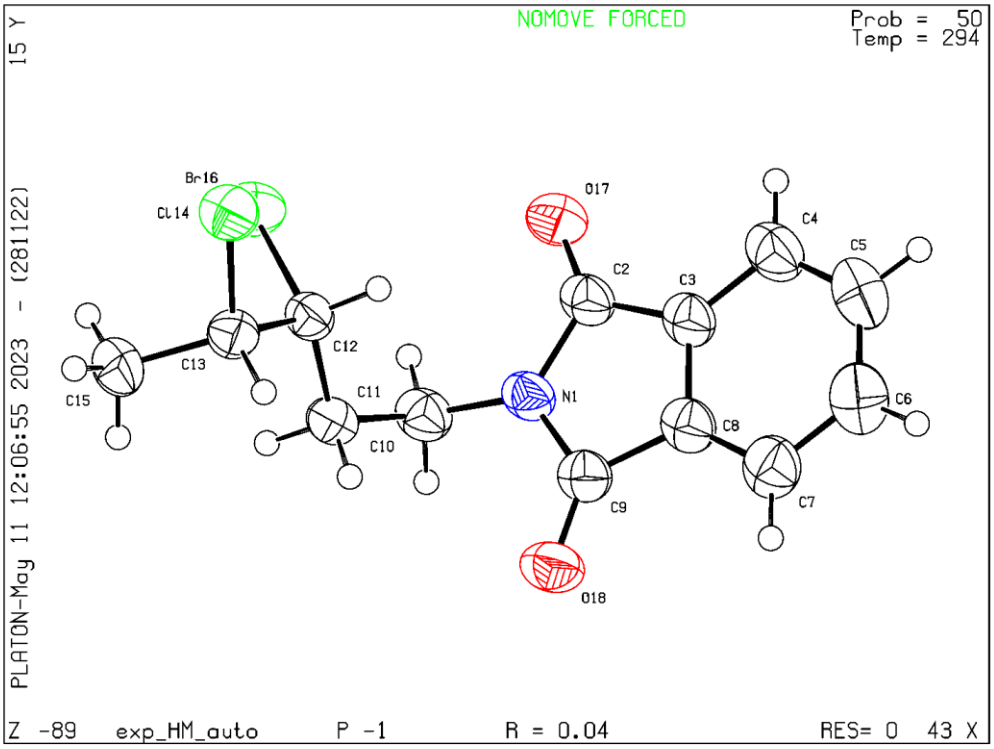

Supplementary Fig. 2. Thermal ellipsoid plot of **18n** at the 50% probability level, CCDC 2262334.

3.2. Computational Study – NCI Analysis

The NCI plot analysis[14,15] was performed using the Multiwfn software[16] and visualized by VMD[17]. In the (*Z*)-2-pentene-TT^{2+} adduct, reddish isosurfaces are shown between the ethyl group and the thianthrene backbone, indicating the presence of repulsive van der Waals interaction, which is proposed to be responsible for the site-selective opening on that side.

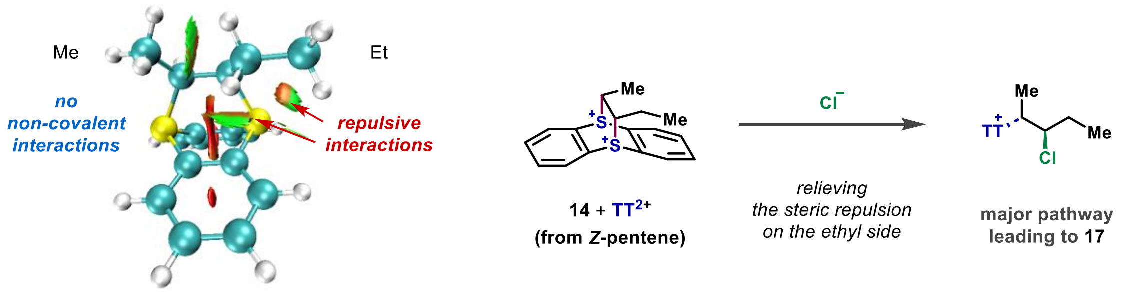

Supplementary Fig. 3. Rationalization of the site-selectivity via the NCI analysis of an alkene-TT^{2+} adduct.

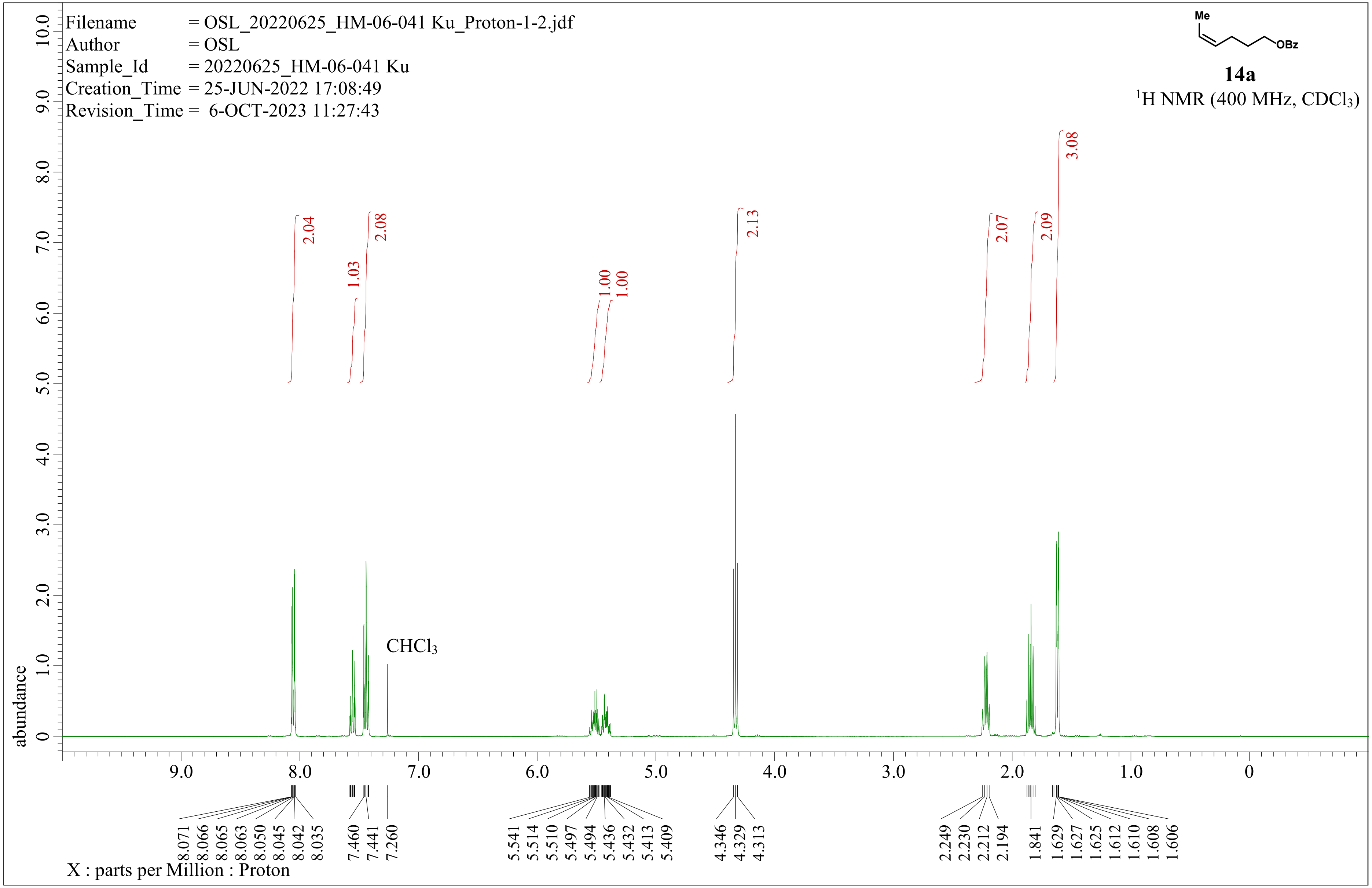

Supplementary Fig. 4. ^{1}H NMR spectrum of compound **14a**, recorded at 400 MHz and 298 K in $CDCl_3$.

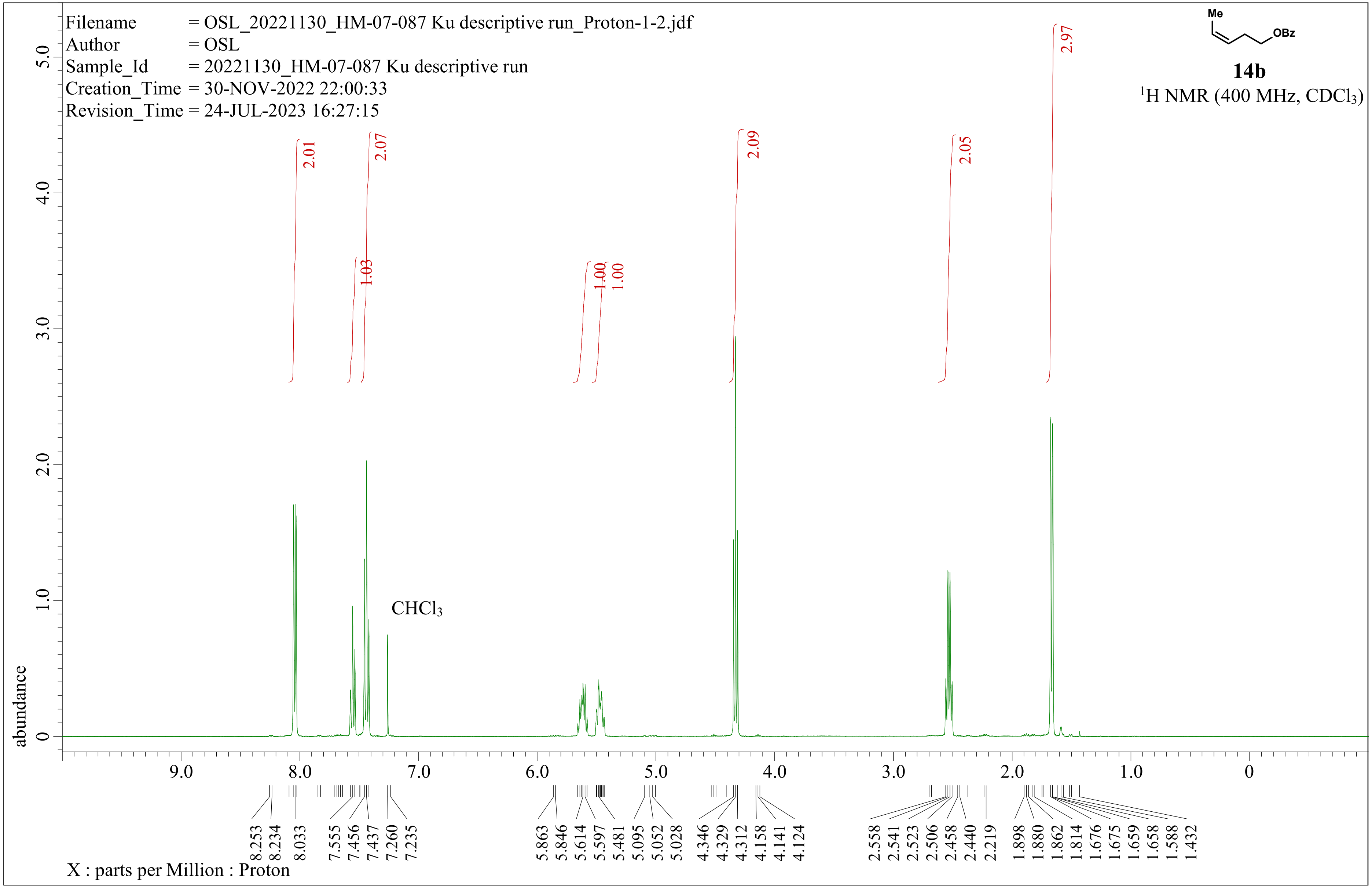

Supplementary Fig. 5. ^{1}H NMR spectrum of compound **14b**, recorded at 400 MHz and 298 K in $CDCl_3$.

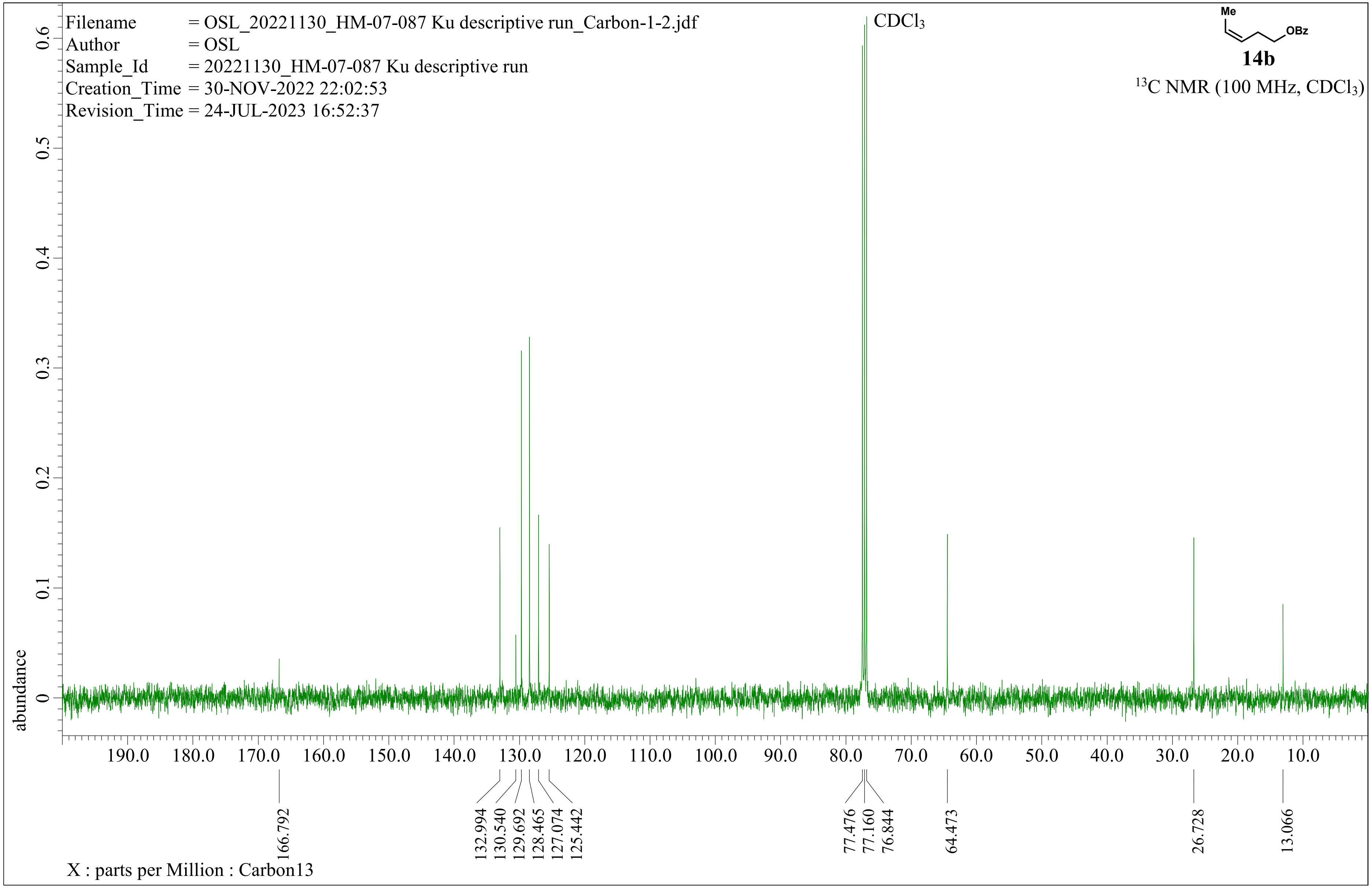

Supplementary Fig. 6. ^{13}C NMR spectrum of compound **14b**, recorded at 100 MHz and 298 K in $CDCl_3$.

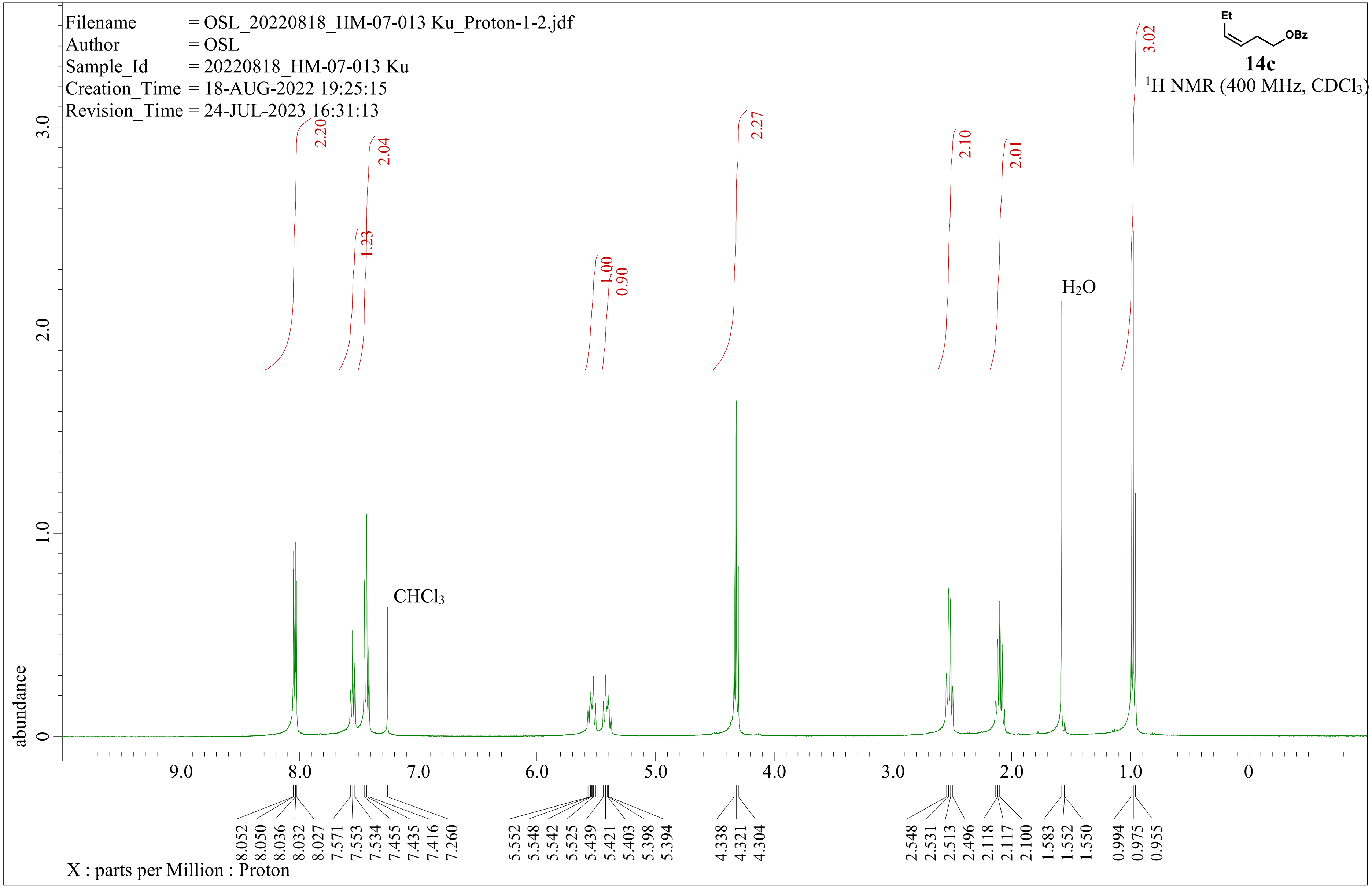

Supplementary Fig. 7. ^{1}H NMR spectrum of compound **14c**, recorded at 400 MHz and 298 K in $CDCl_3$.

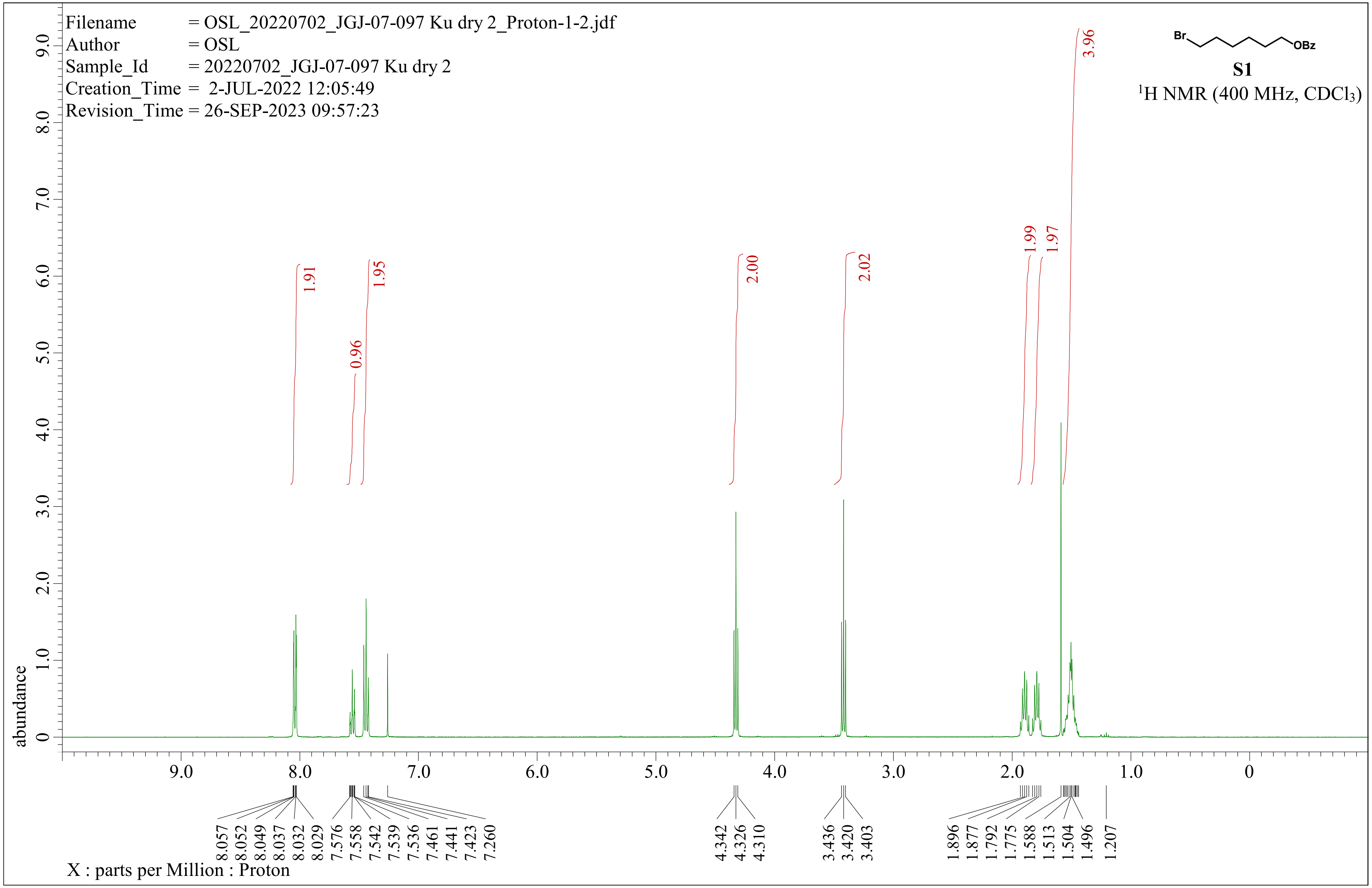

Supplementary Fig. 8. ^{1}H NMR spectrum of compound **S1**, recorded at 400 MHz and 298 K in $CDCl_3$.

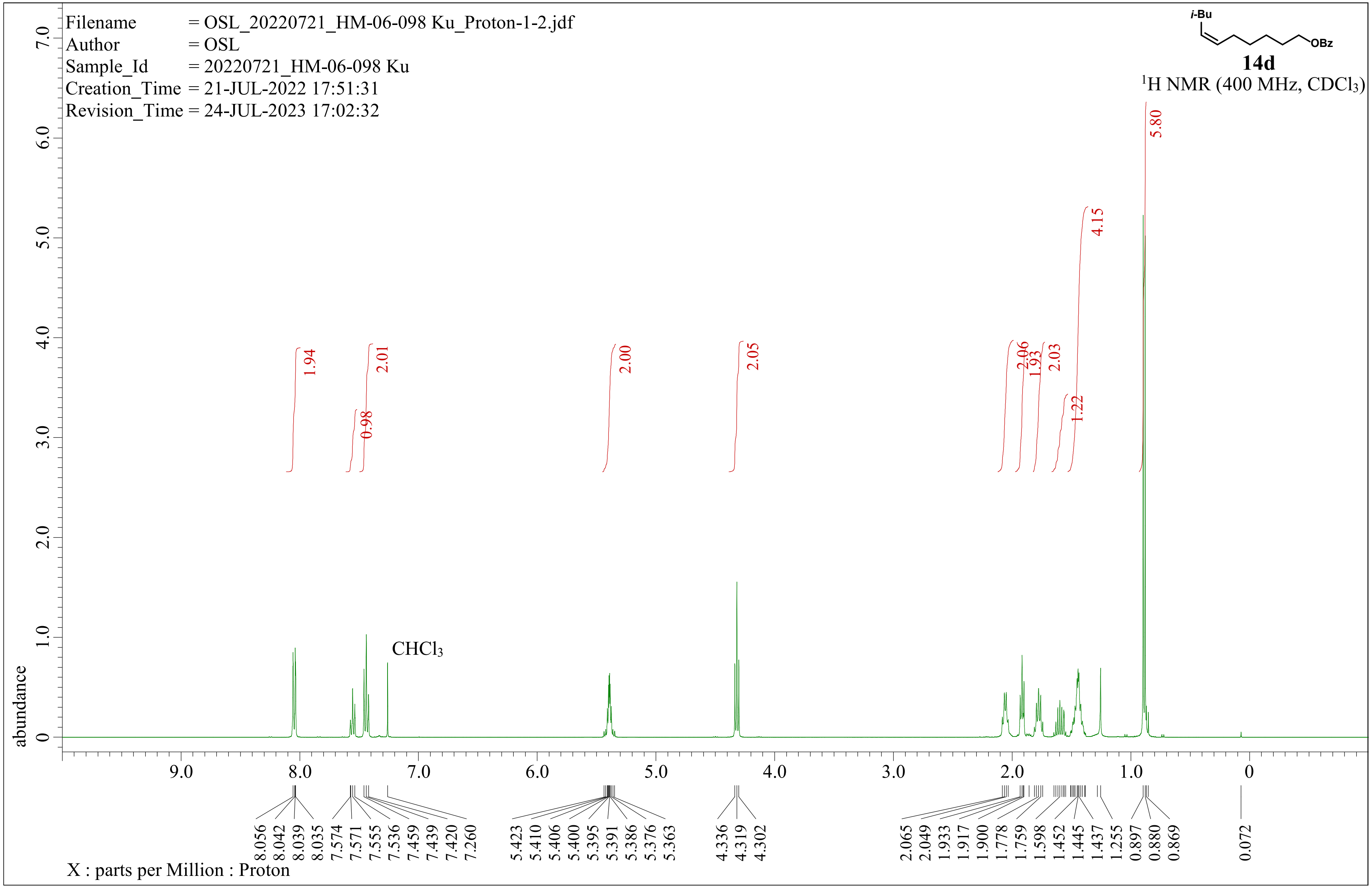

Supplementary Fig. 9. ^{1}H NMR spectrum of compound **14d**, recorded at 400 MHz and 298 K in $CDCl_3$.

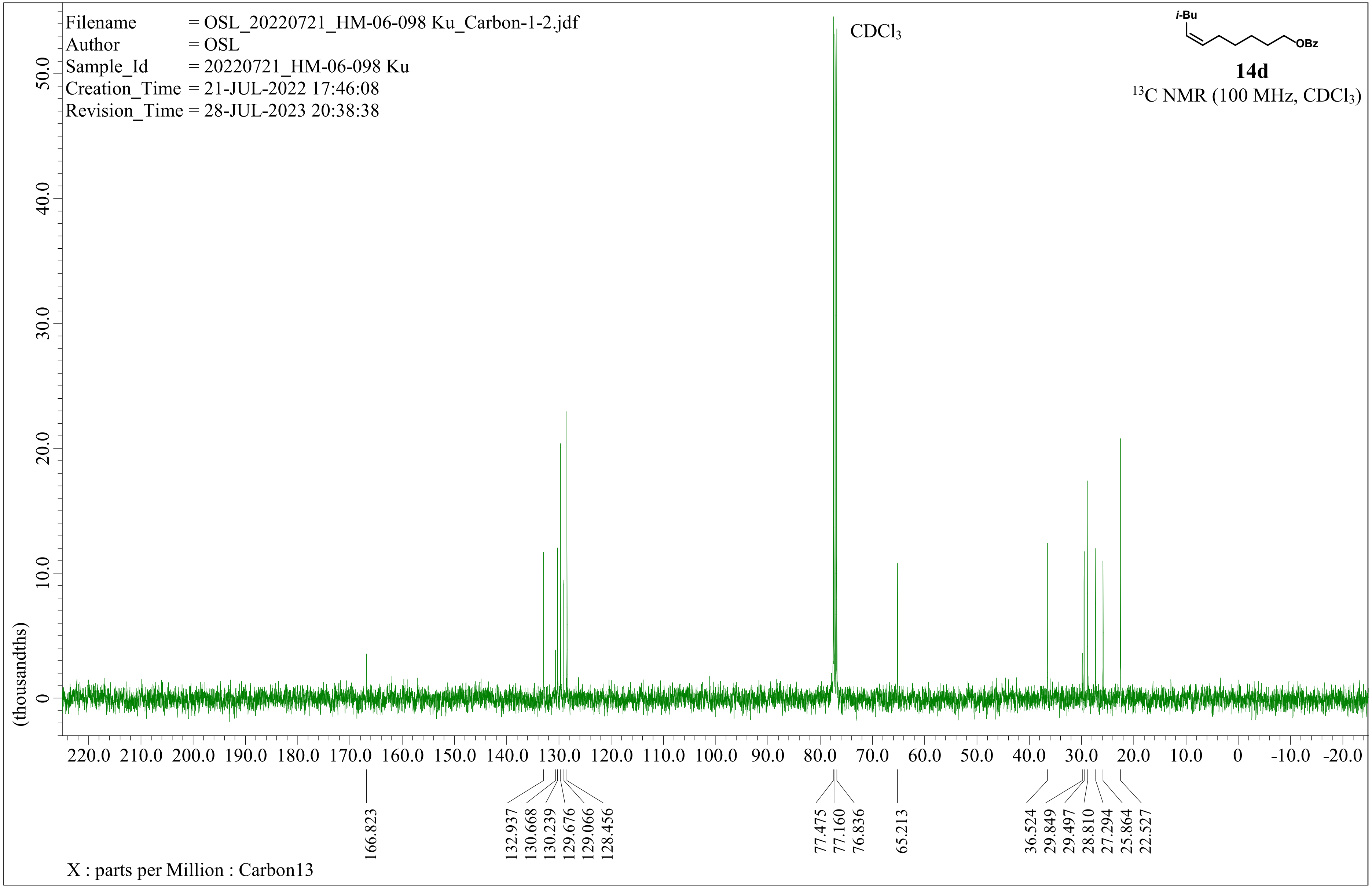

Supplementary Fig. 10. ^{13}C NMR spectrum of compound **14d**, recorded at 100 MHz and 298 K in $CDCl_3$.

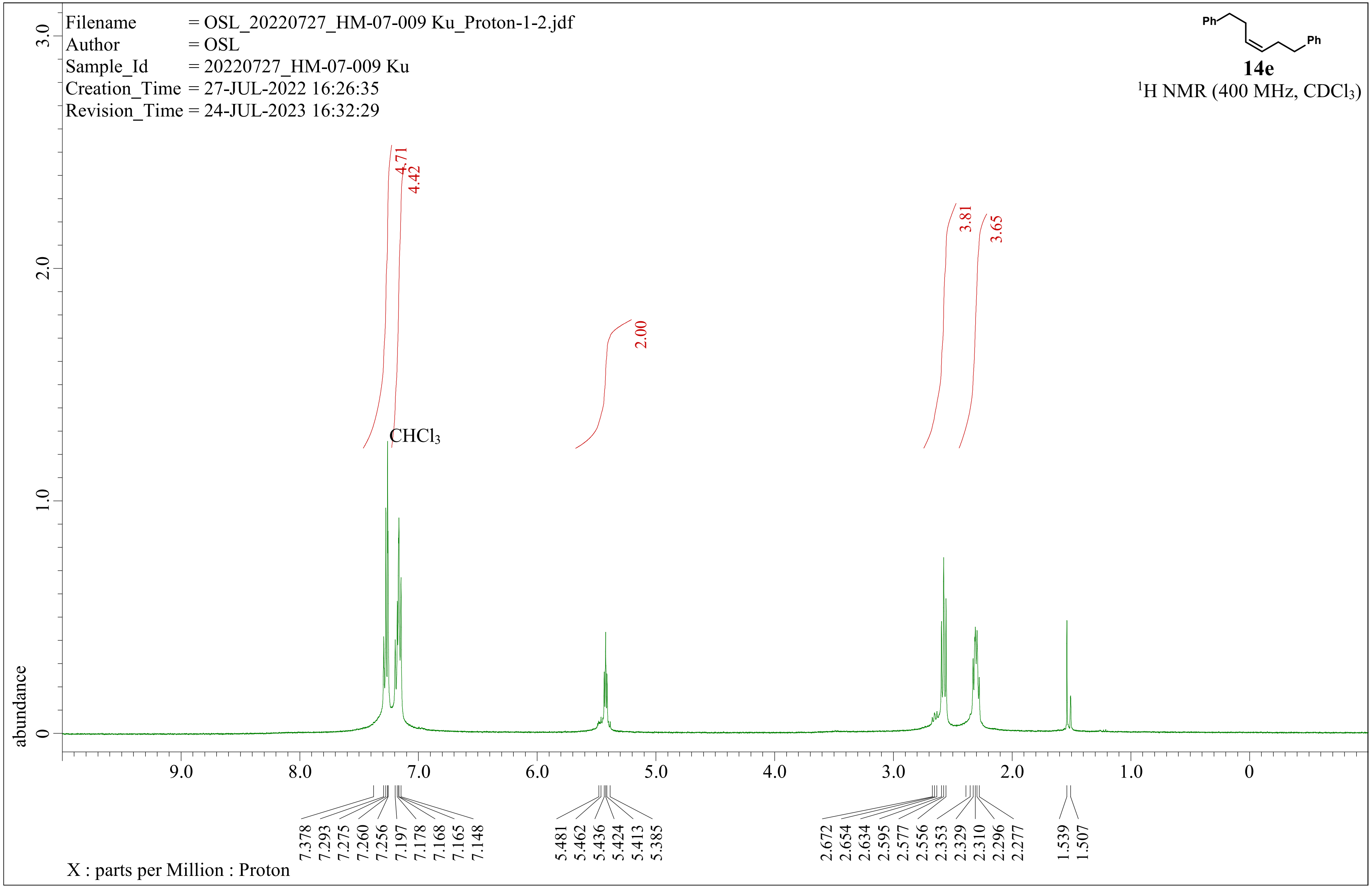

Supplementary Fig. 11. ^{1}H NMR spectrum of compound **14e**, recorded at 400 MHz and 298 K in $CDCl_3$.

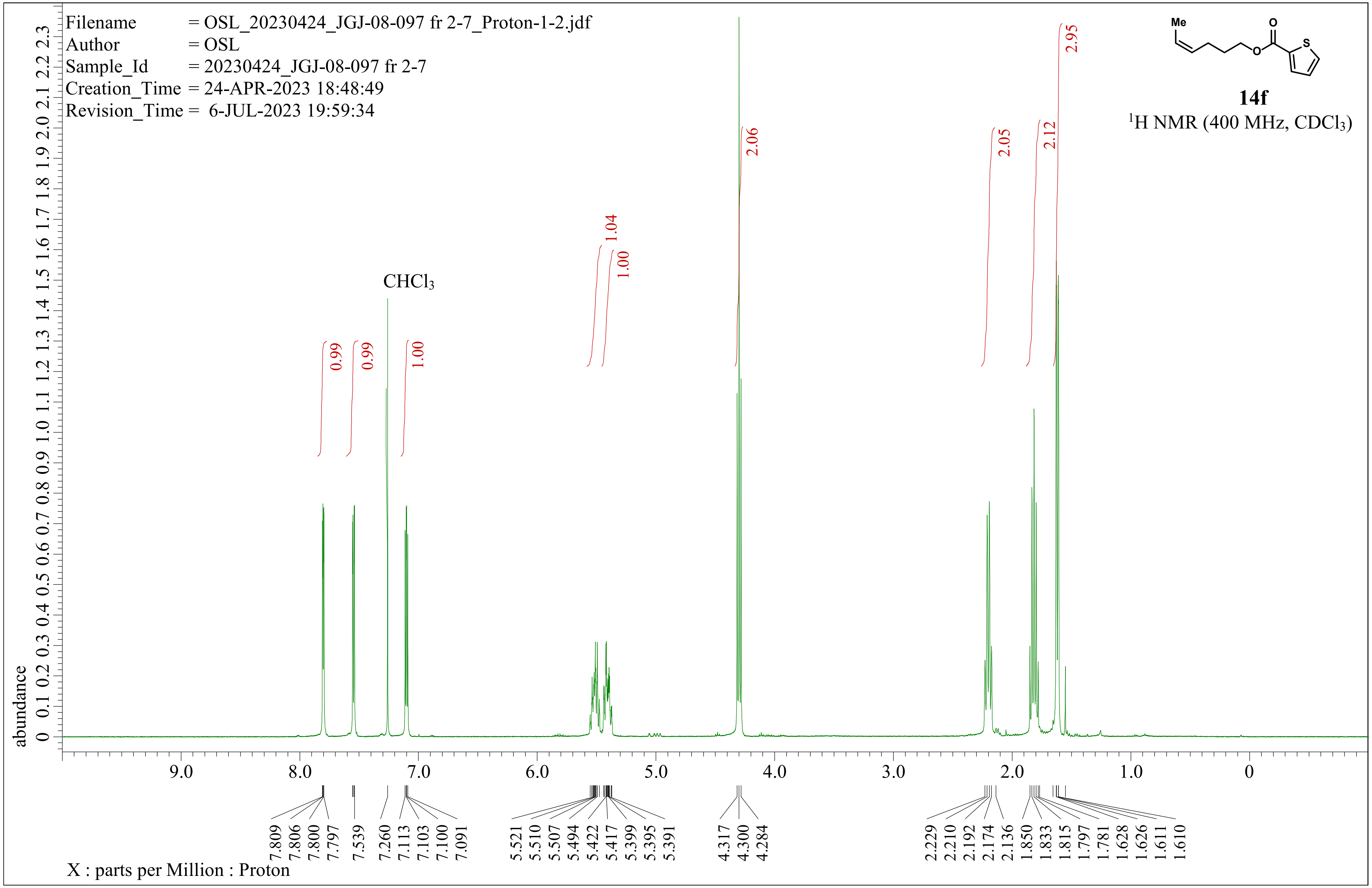

Supplementary Fig. 12. ^{1}H NMR spectrum of compound **14f**, recorded at 400 MHz and 298 K in $CDCl_3$.

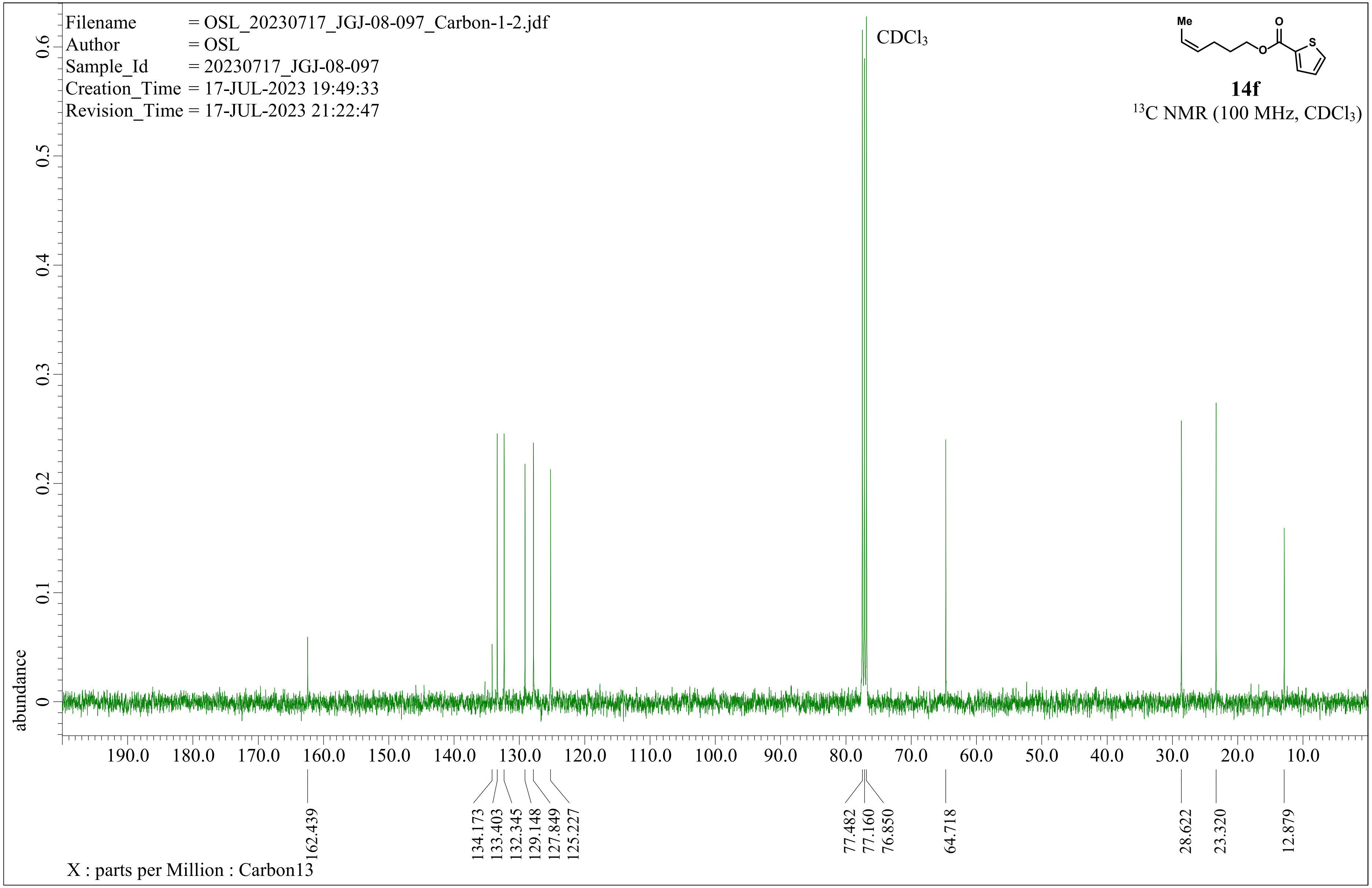

Supplementary Fig. 13. ${}^{13}C$ NMR spectrum of compound **14f**, recorded at 100 MHz and 298 K in $CDCl_3$.

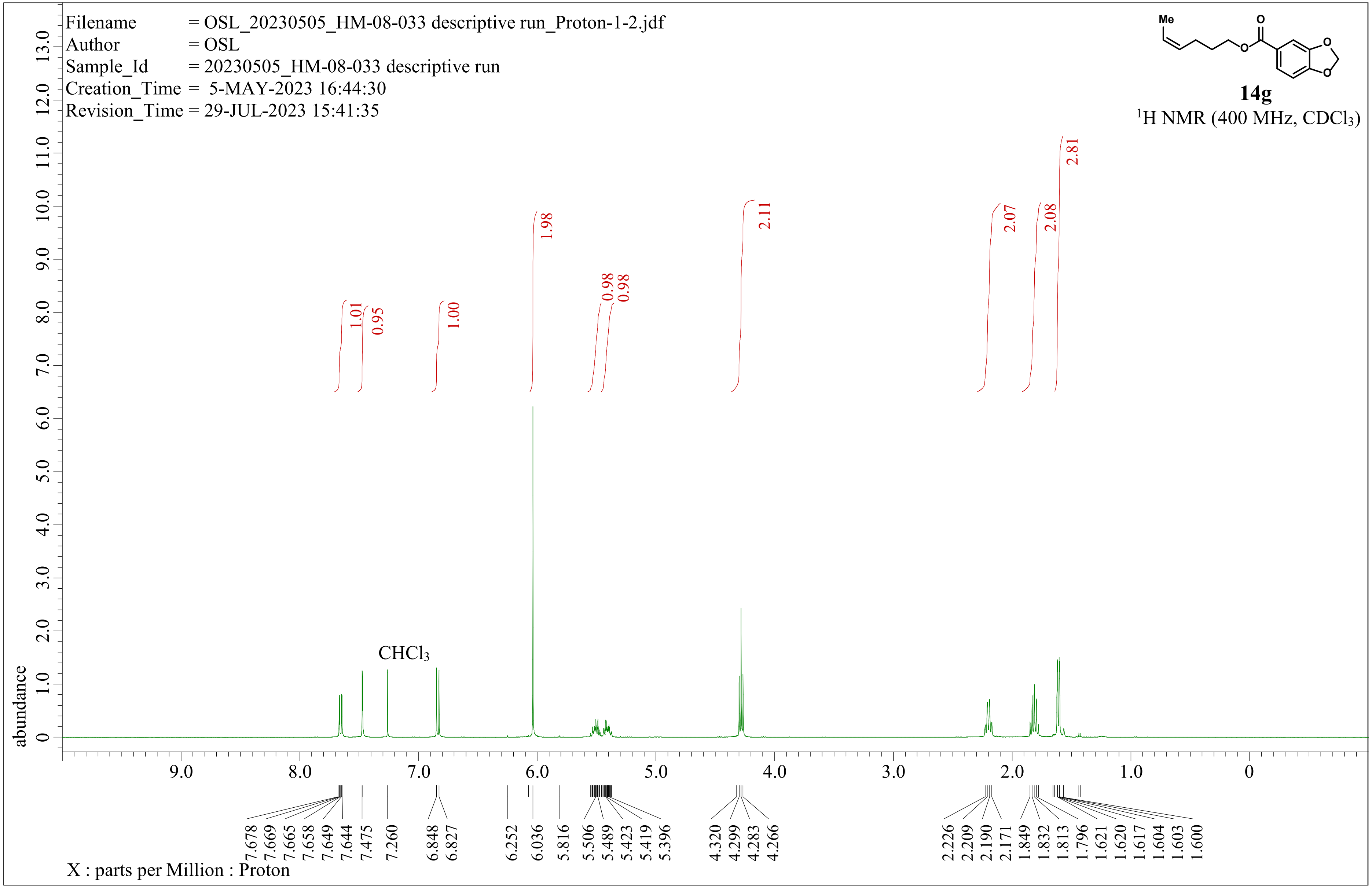

Supplementary Fig. 14. ^{1}H NMR spectrum of compound **14g**, recorded at 400 MHz and 298 K in $CDCl_3$.

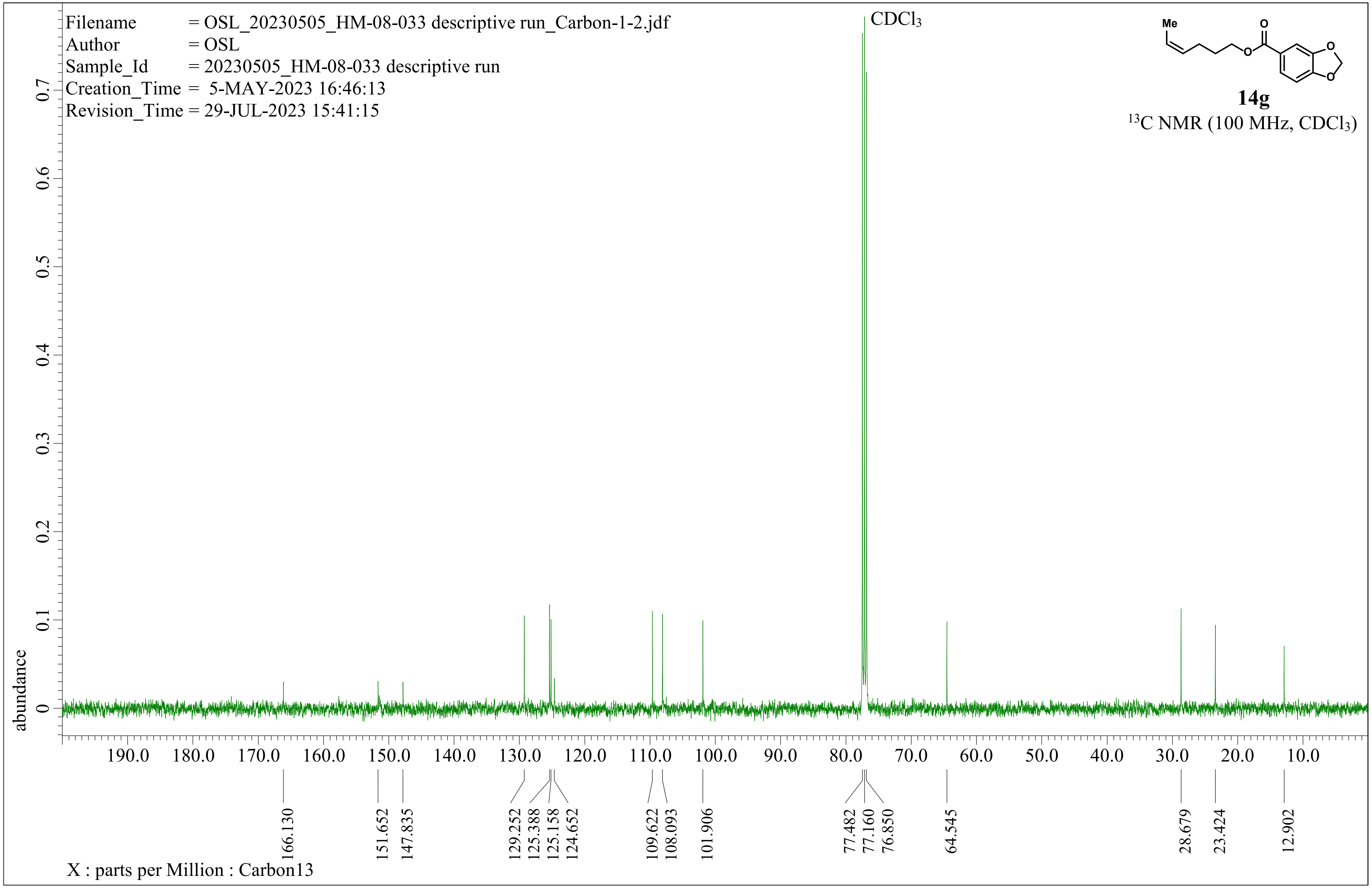

Supplementary Fig. 15. ^{13}C NMR spectrum of compound **14g**, recorded at 100 MHz and 298 K in $CDCl_3$.

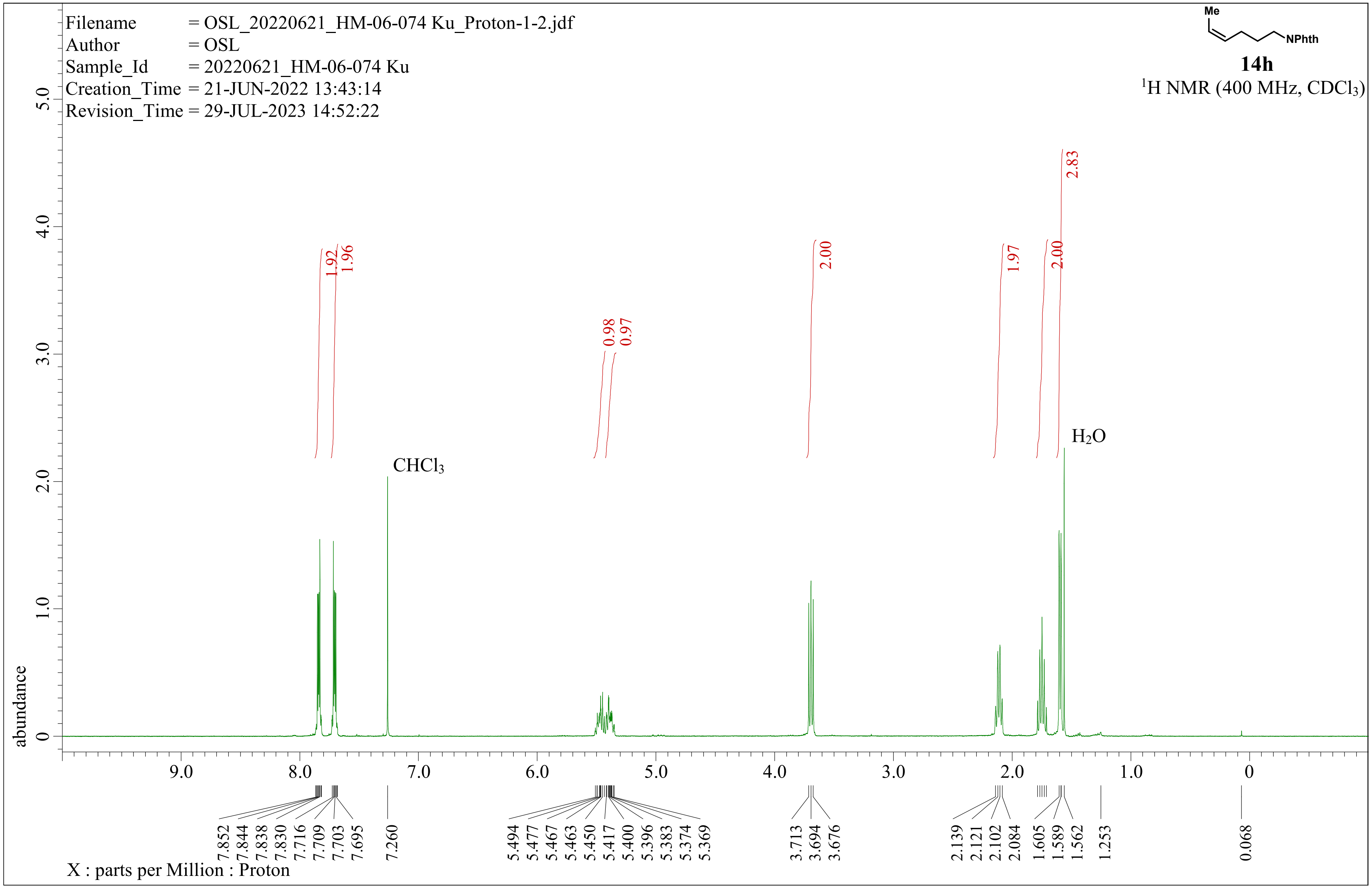

Supplementary Fig. 16. ^1H NMR spectrum of compound **14h**, recorded at 400 MHz and 298 K in $CDCl_3$.

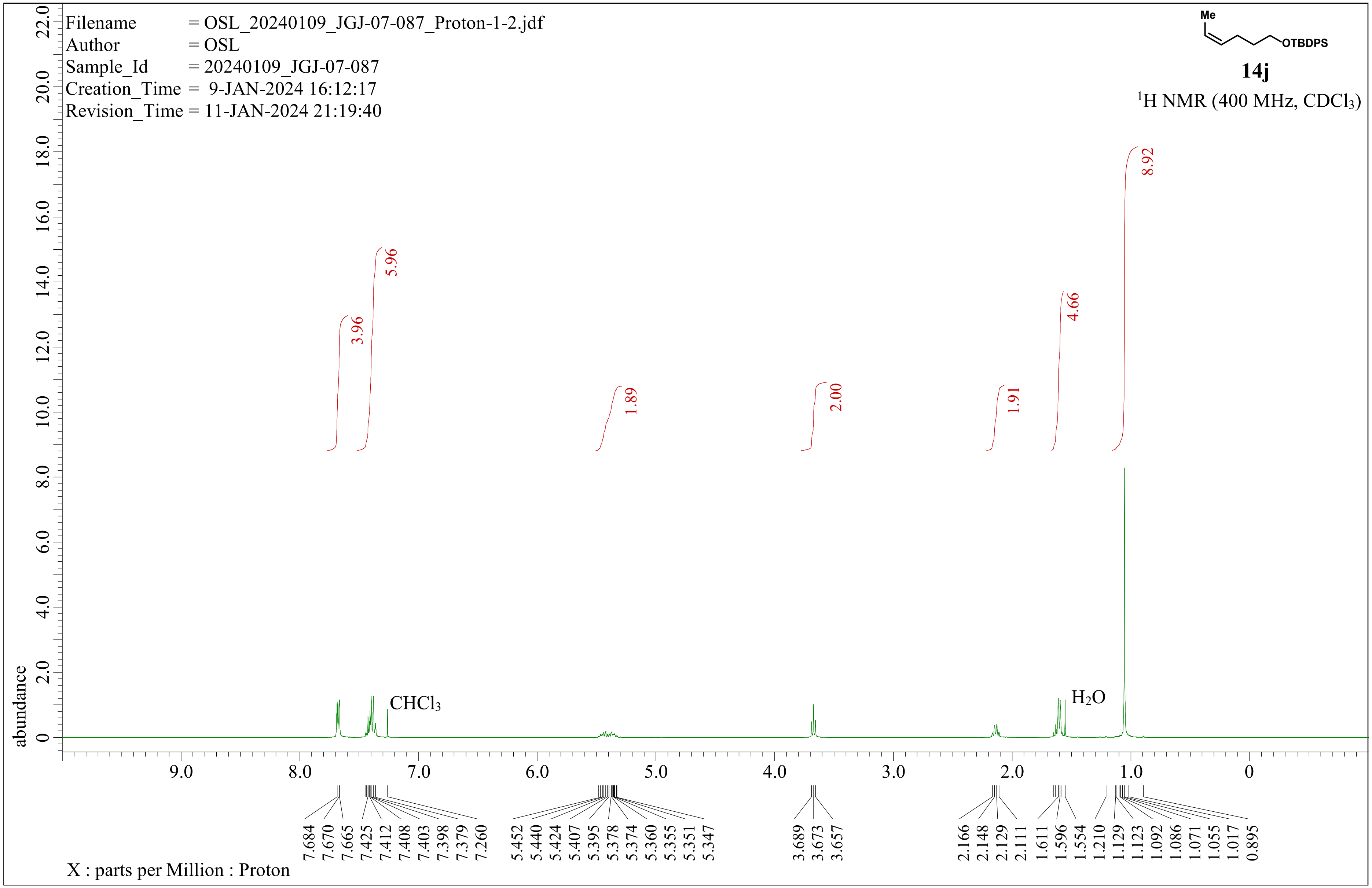

Supplementary Fig. 17. ^{1}H NMR spectrum of compound **14j**, recorded at 400 MHz and 298 K in $CDCl_3$.

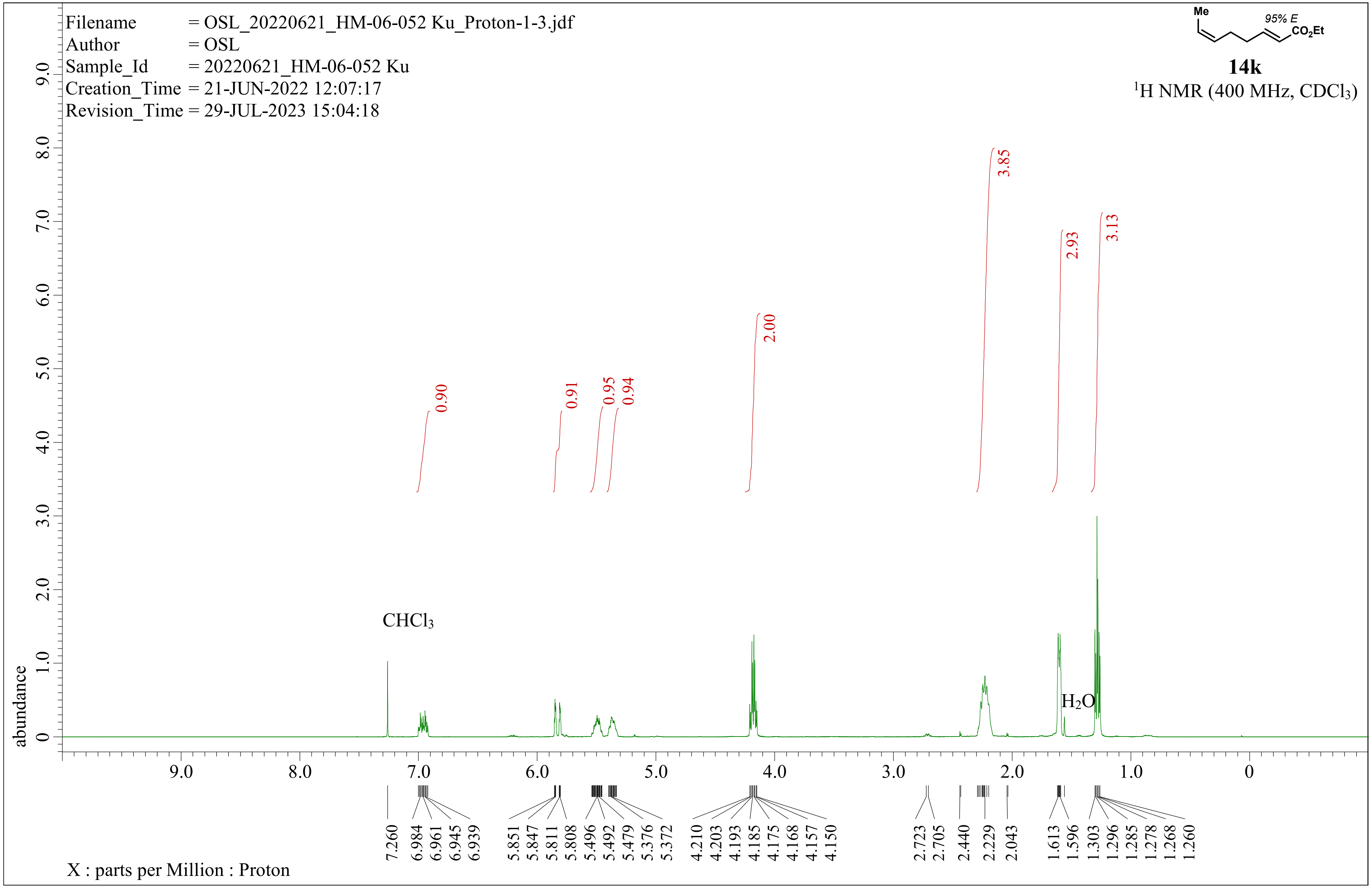

Supplementary Fig. 18. ^{1}H NMR spectrum of compound **14k**, recorded at 400 MHz and 298 K in $CDCl_3$.

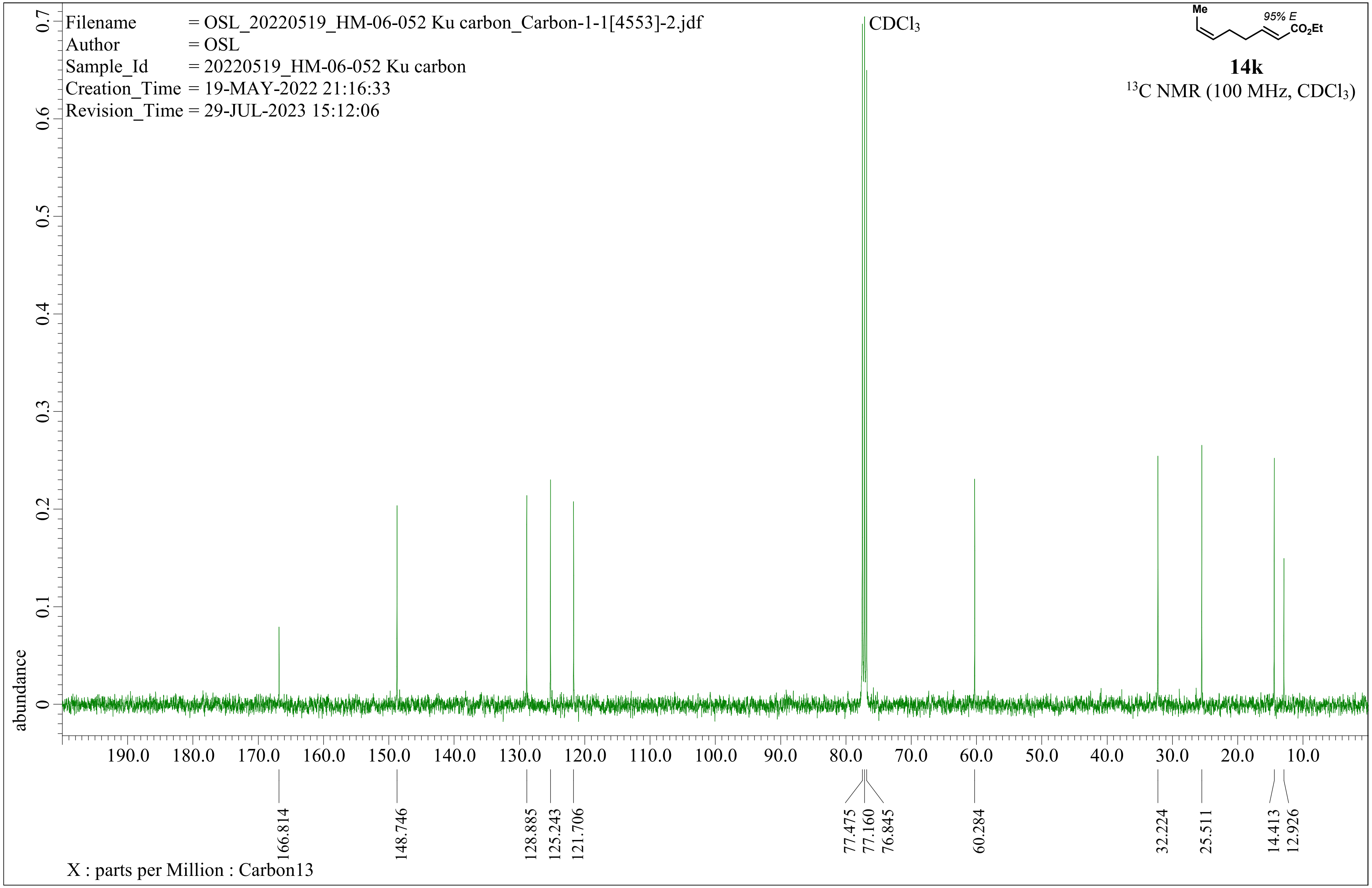

Supplementary Fig. 19. ^{13}C NMR spectrum of compound **14k**, recorded at 100 MHz and 298 K in $CDCl_3$.

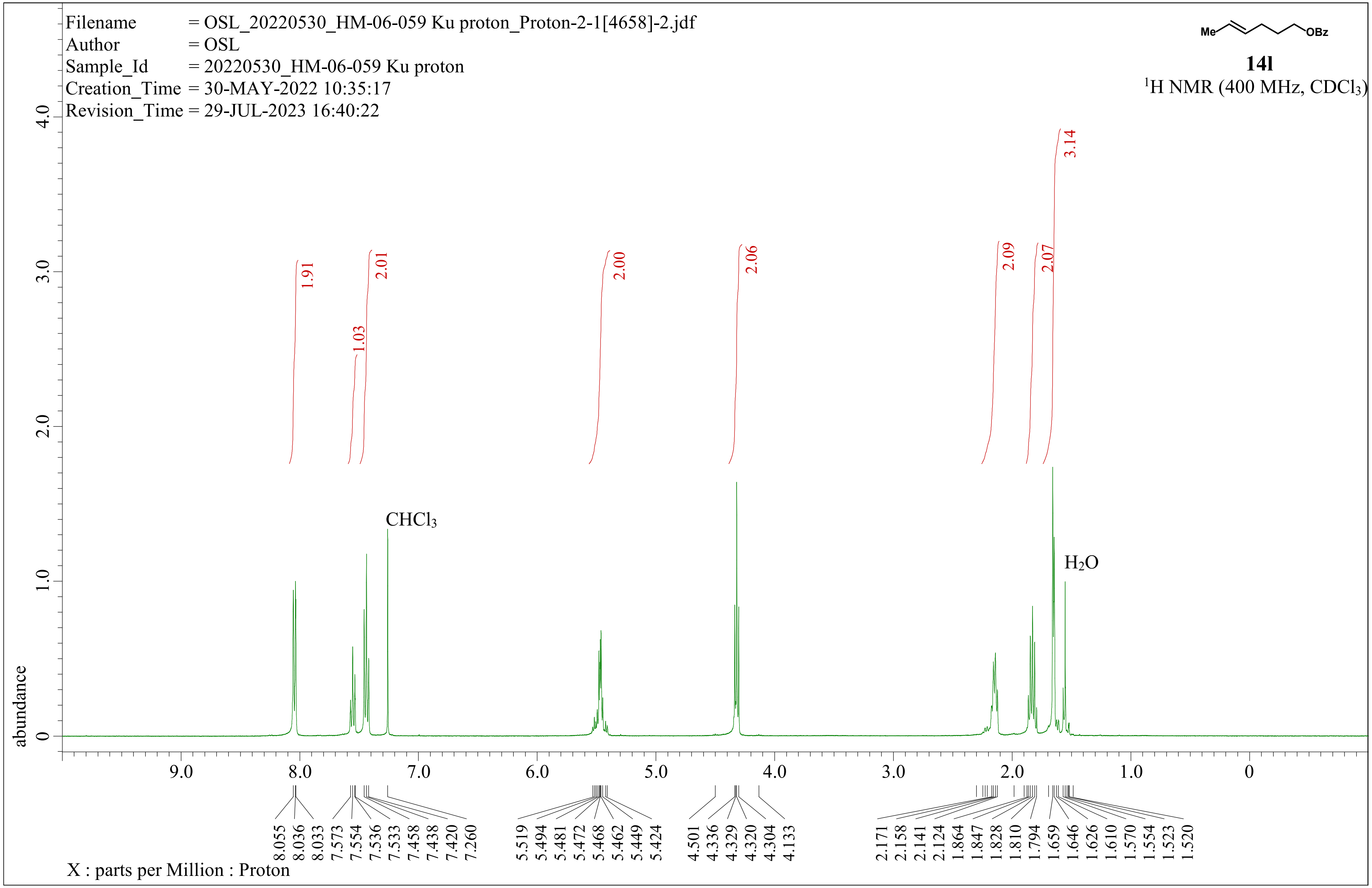

Supplementary Fig. 20. ^1H NMR spectrum of compound **14l**, recorded at 400 MHz and 298 K in $CDCl_3$.

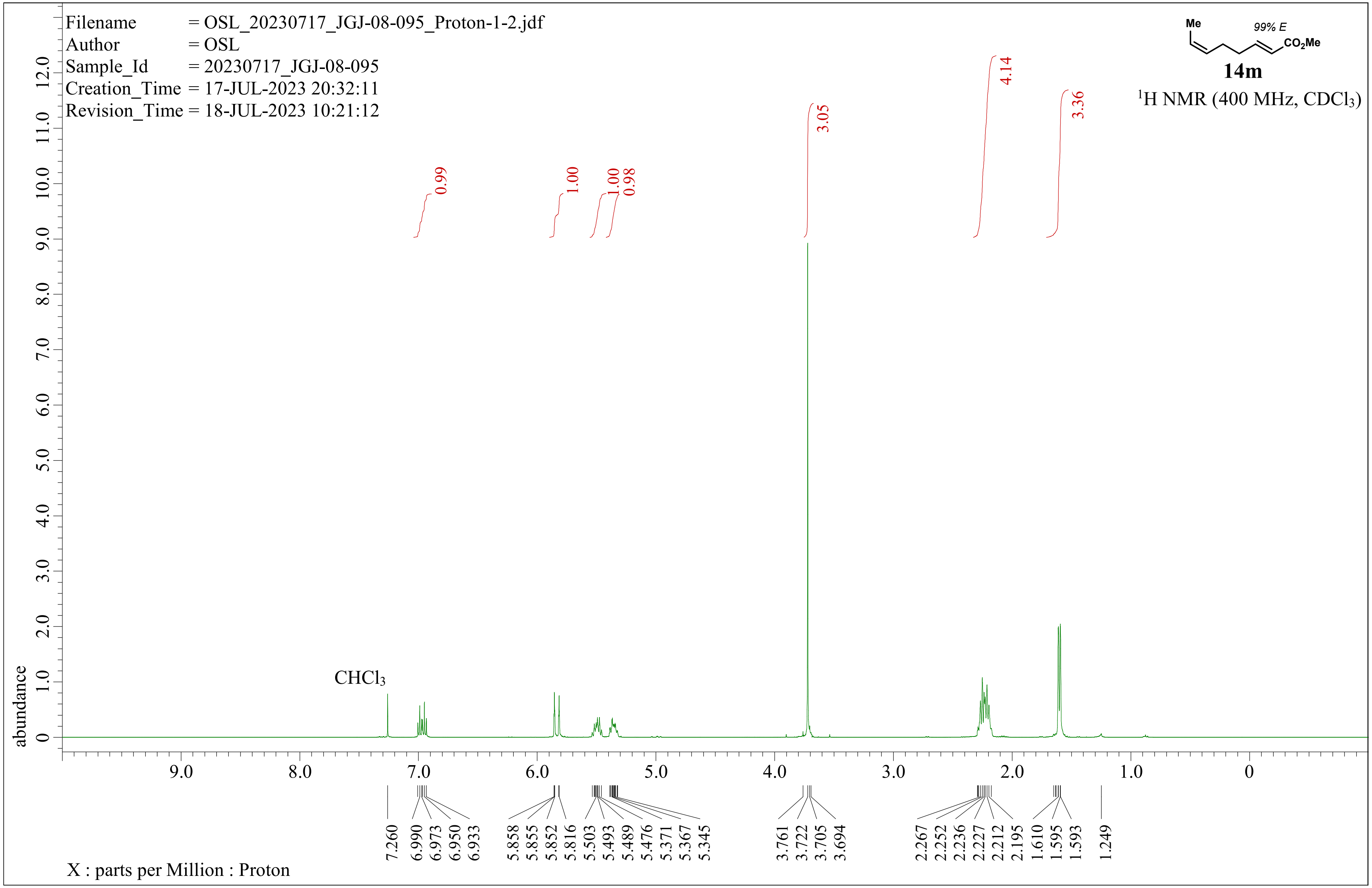

Supplementary Fig. 21. ^{1}H NMR spectrum of compound **14m**, recorded at 400 MHz and 298 K in $CDCl_3$.

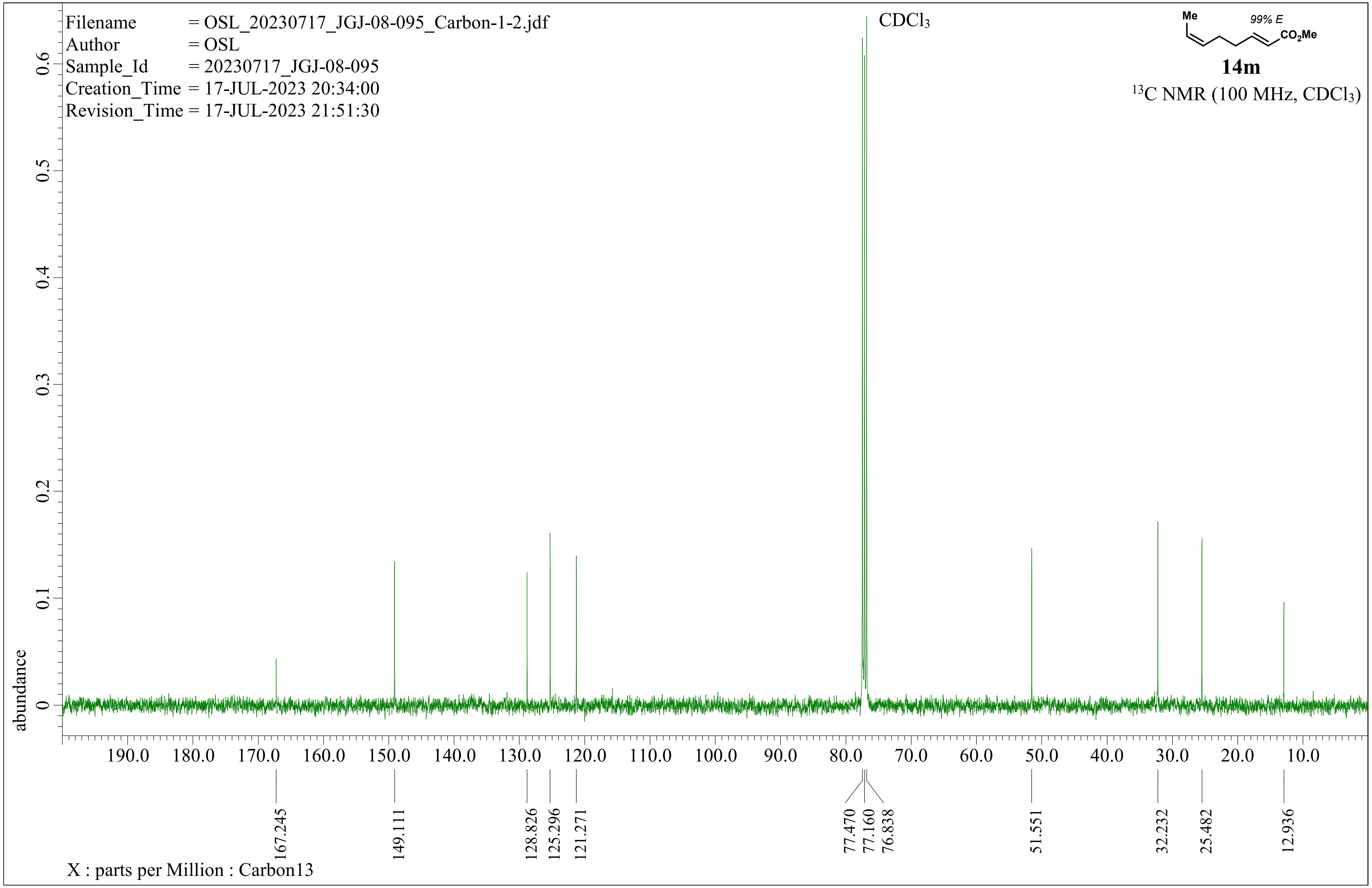

Supplementary Fig. 22. ^{13}C NMR spectrum of compound **14m**, recorded at 100 MHz and 298 K in $CDCl_3$.

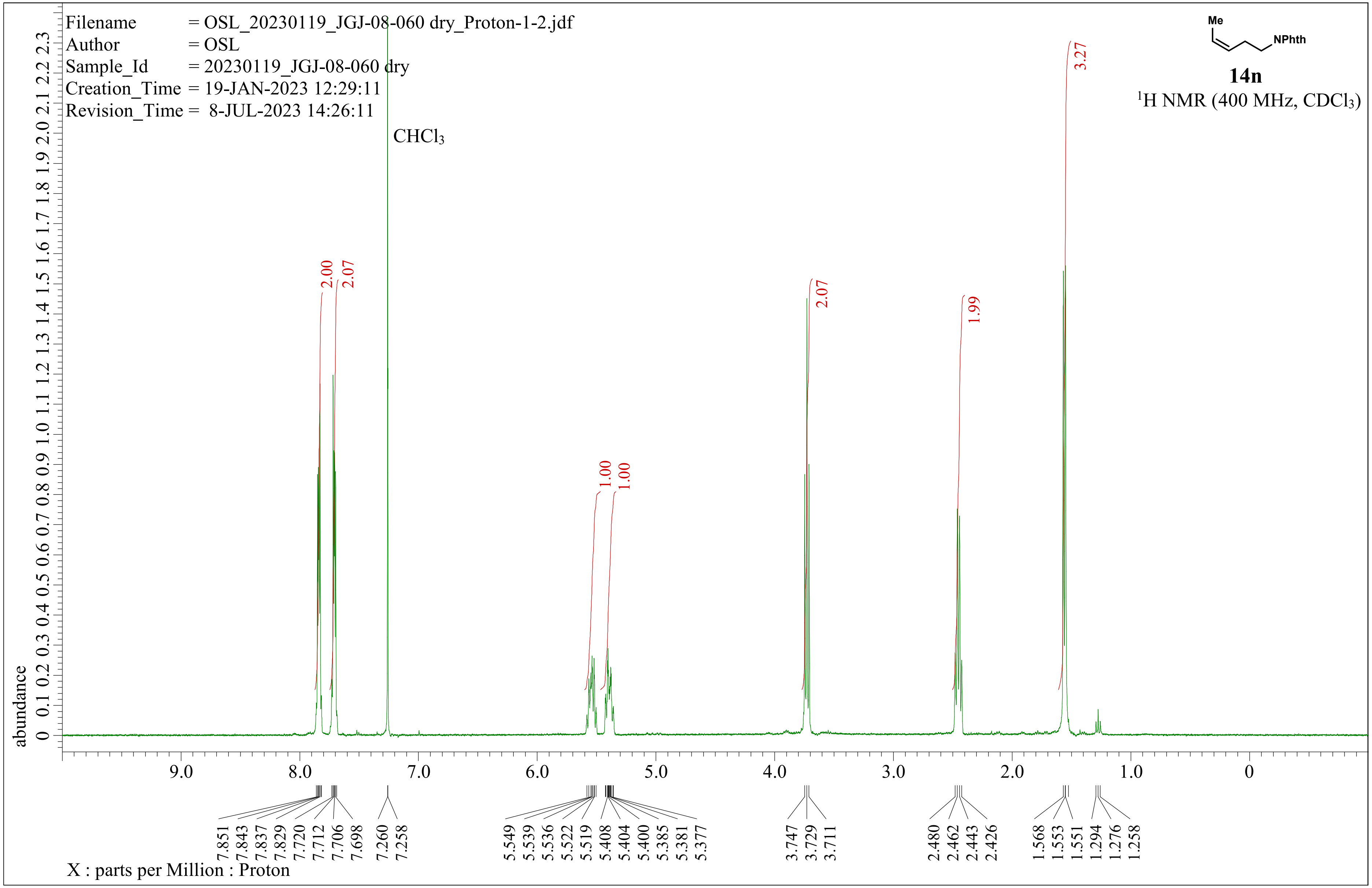

Supplementary Fig. 23. ^{1}H NMR spectrum of compound **14n**, recorded at 400 MHz and 298 K in $CDCl_3$.

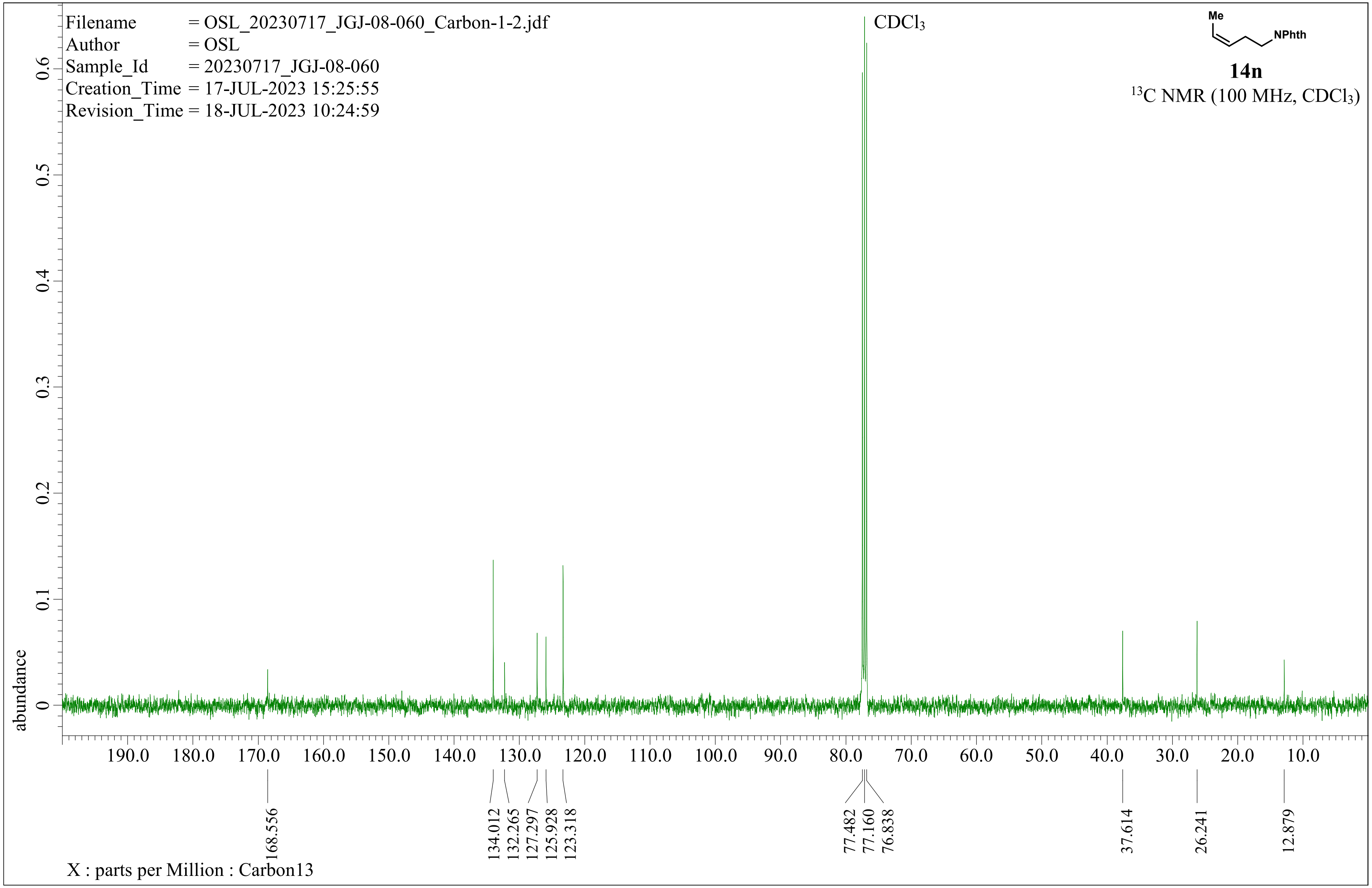

Supplementary Fig. 24. ^{13}C NMR spectrum of compound **14n**, recorded at 100 MHz and 298 K in $CDCl_3$.

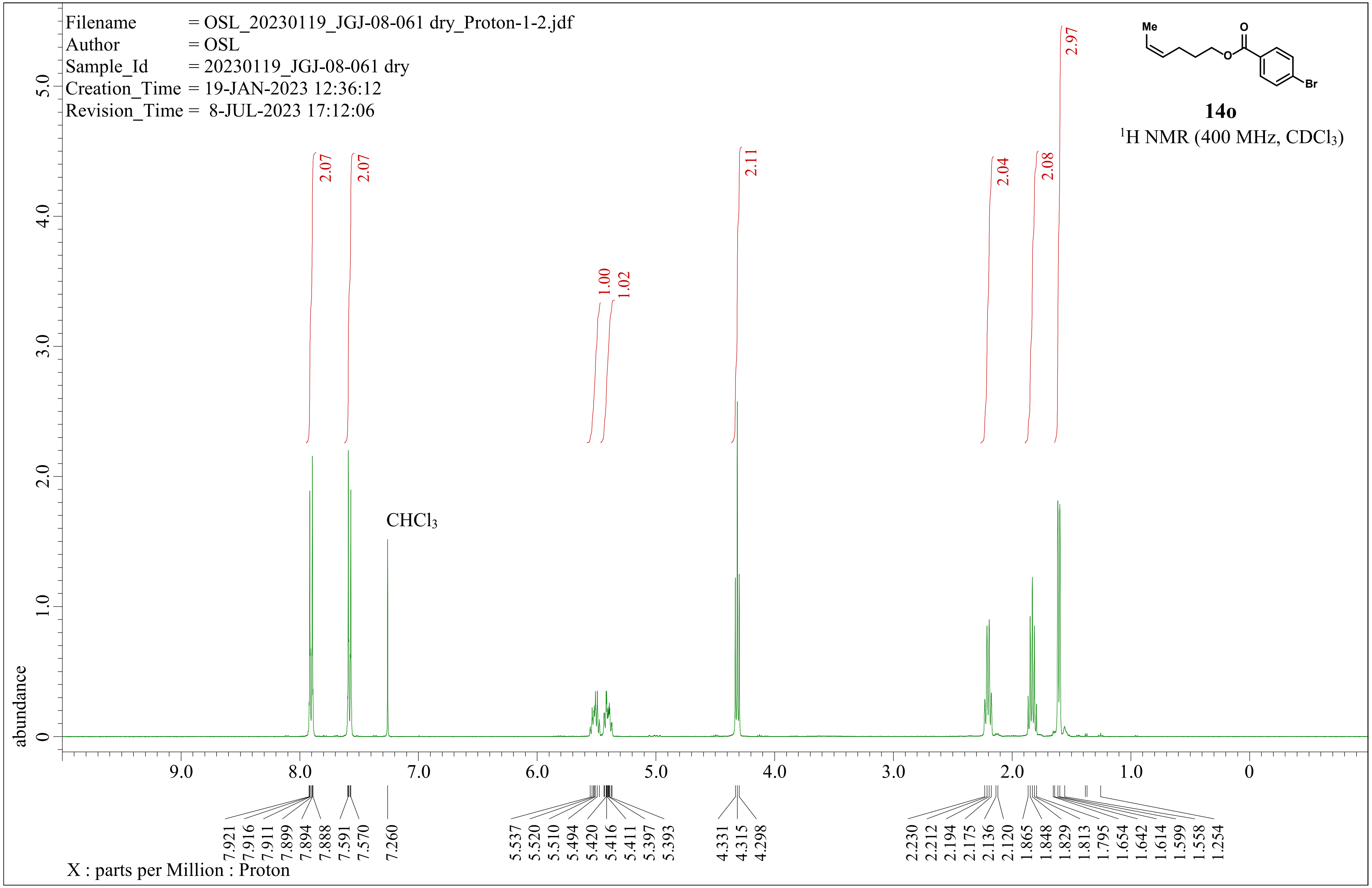

Supplementary Fig. 25. ^{1}H NMR spectrum of compound **14o**, recorded at 400 MHz and 298 K in $CDCl_3$.

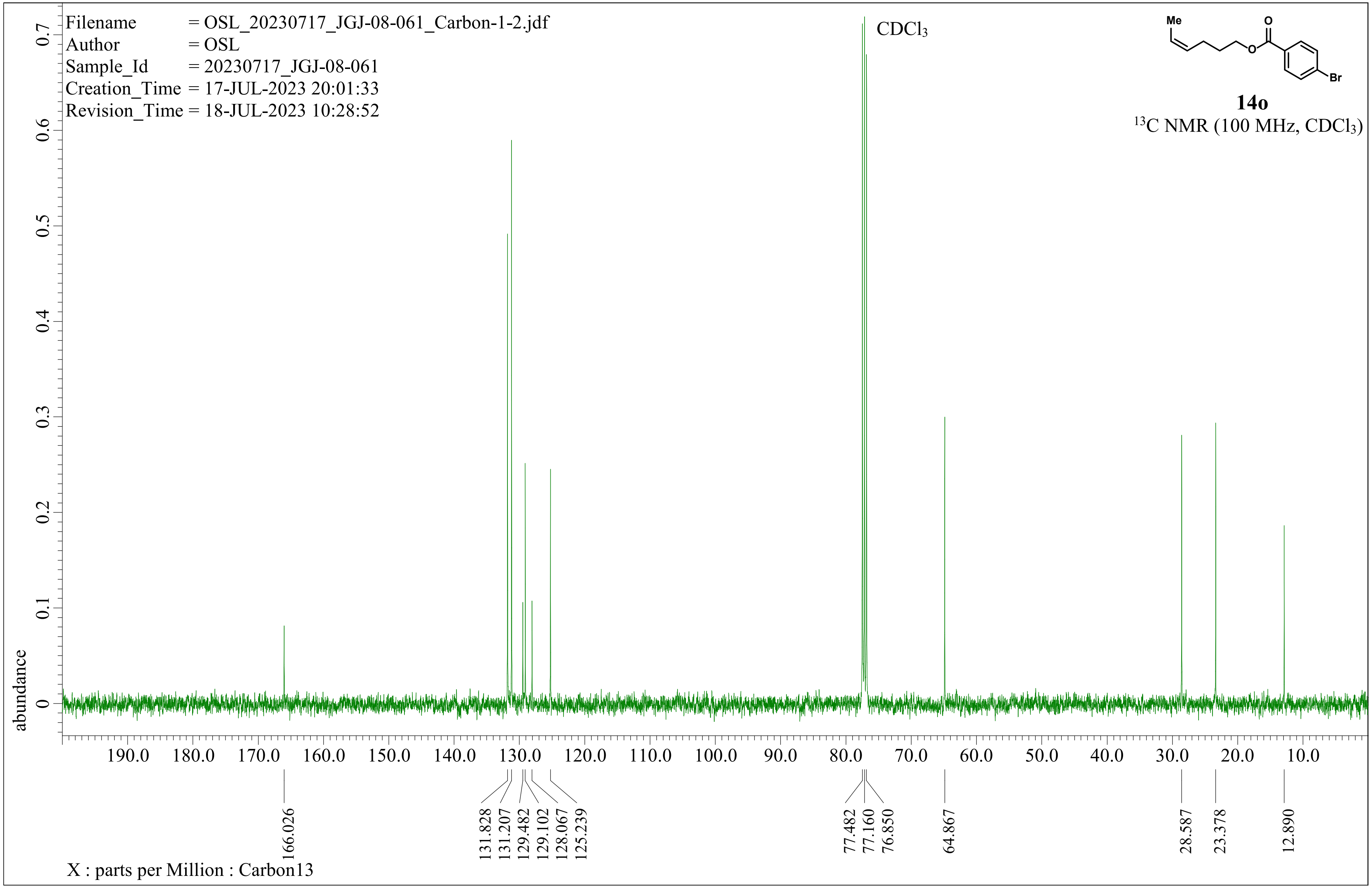

Supplementary Fig. 26. ^{13}C NMR spectrum of compound **14o**, recorded at 100 MHz and 298 K in $CDCl_3$.

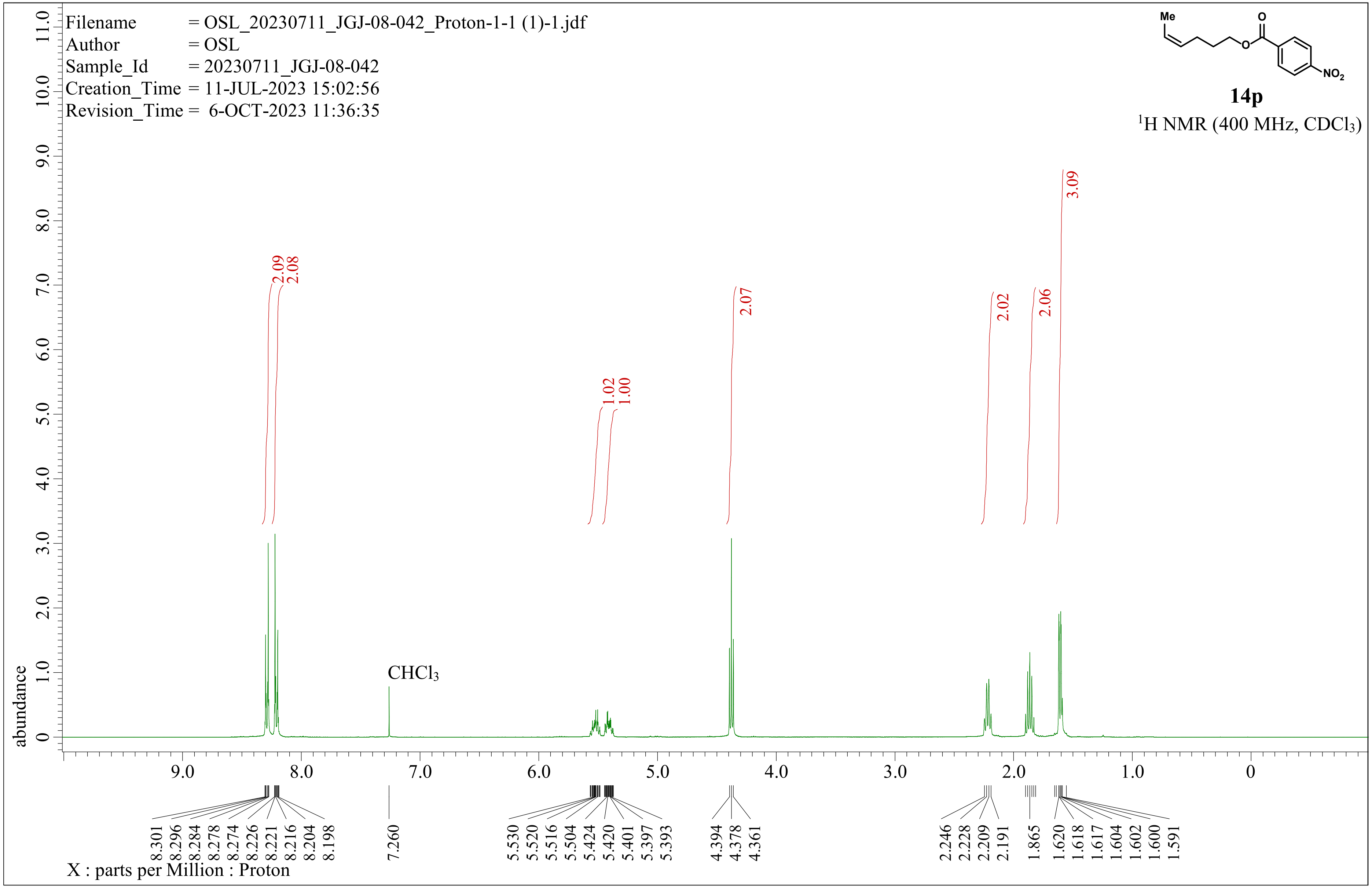

Supplementary Fig. 27. ^{1}H NMR spectrum of compound **14p**, recorded at 400 MHz and 298 K in $CDCl_3$.

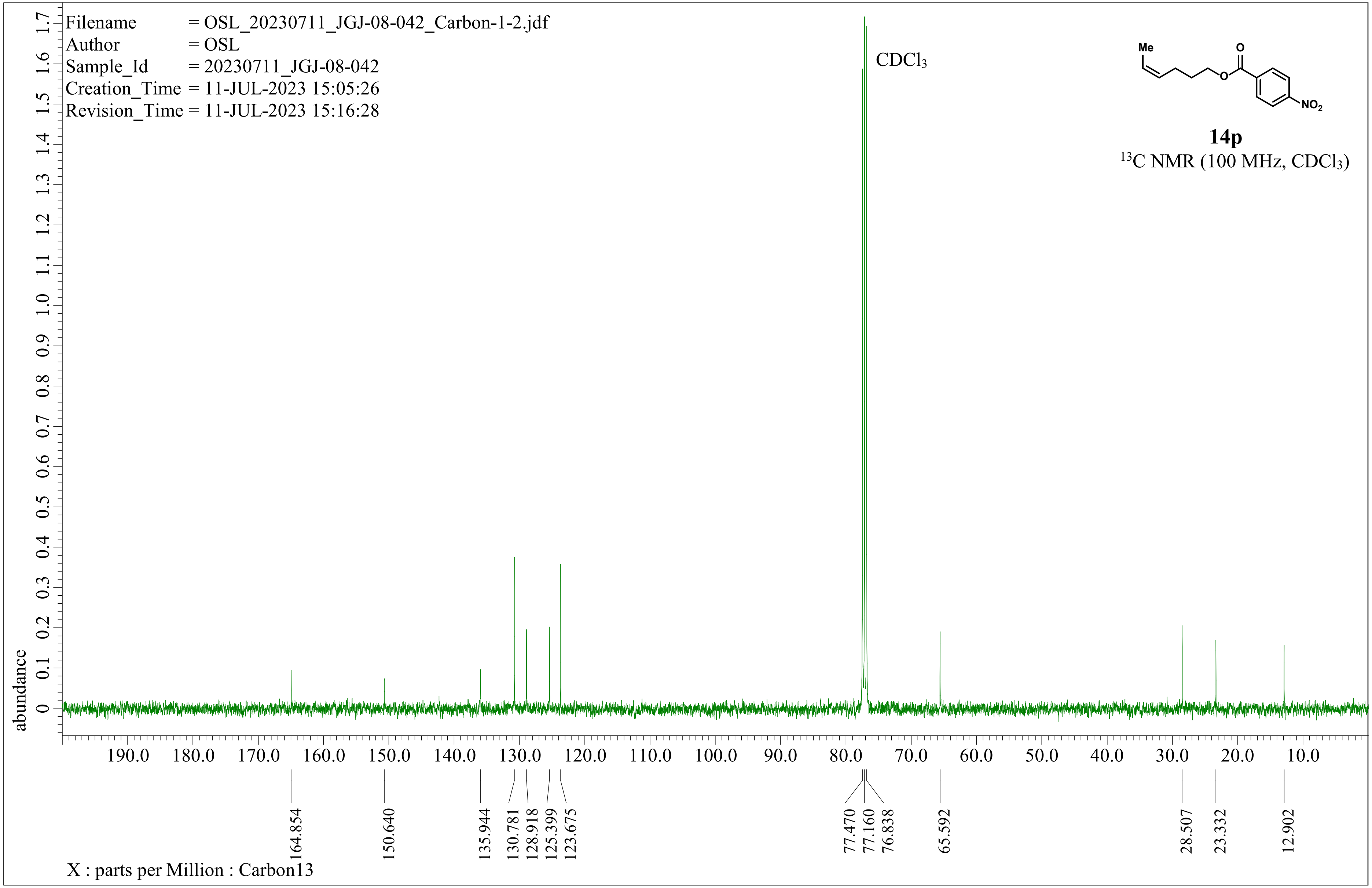

Supplementary Fig. 28. ^{13}C NMR spectrum of compound **14p**, recorded at 100 MHz and 298 K in $CDCl_3$.

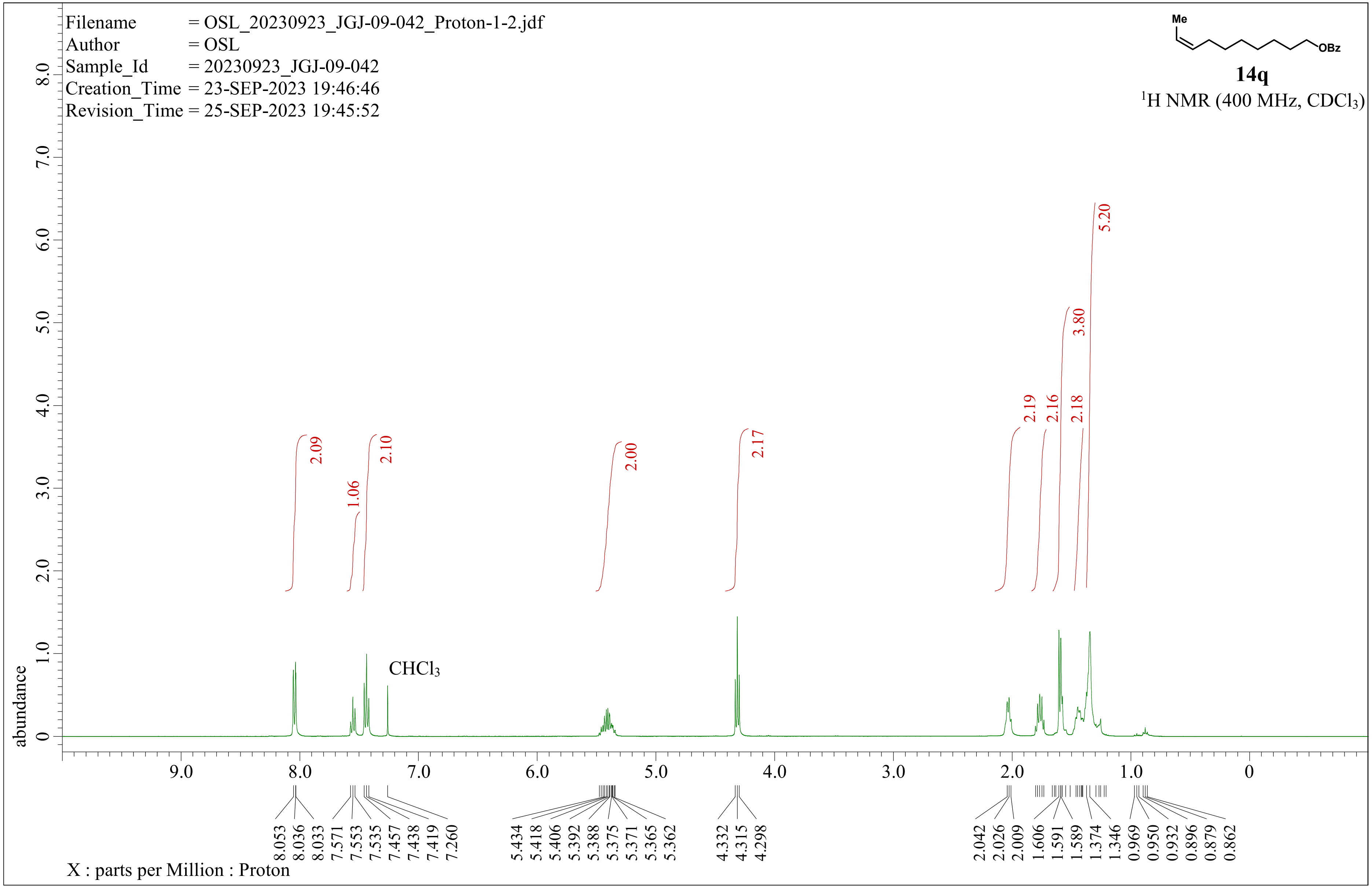

Supplementary Fig. 29. ^{1}H NMR spectrum of compound **14q**, recorded at 400 MHz and 298 K in $CDCl_3$.

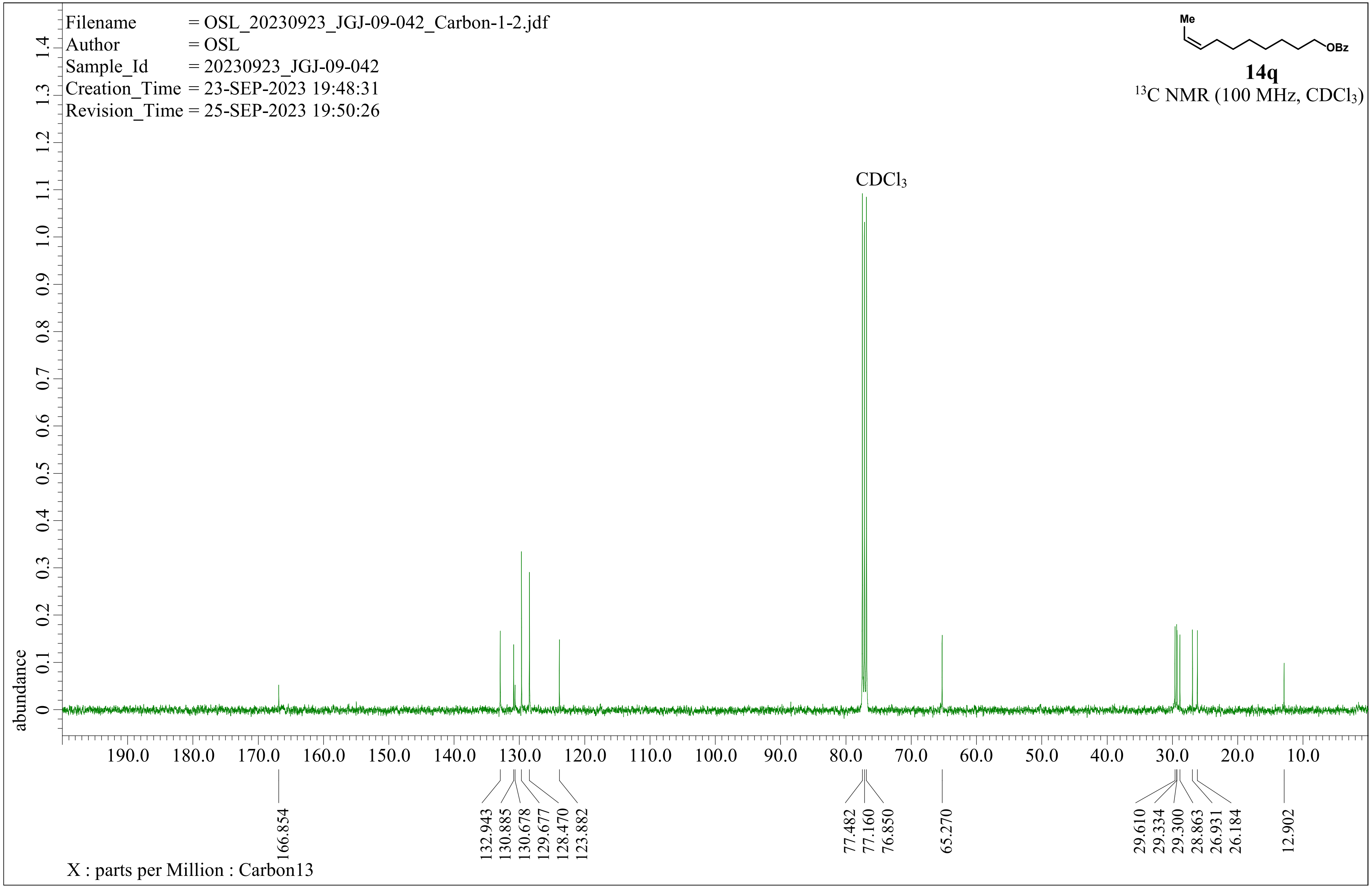

Supplementary Fig. 30. ^{13}C NMR spectrum of compound **14q**, recorded at 100 MHz and 298 K in $CDCl_3$.

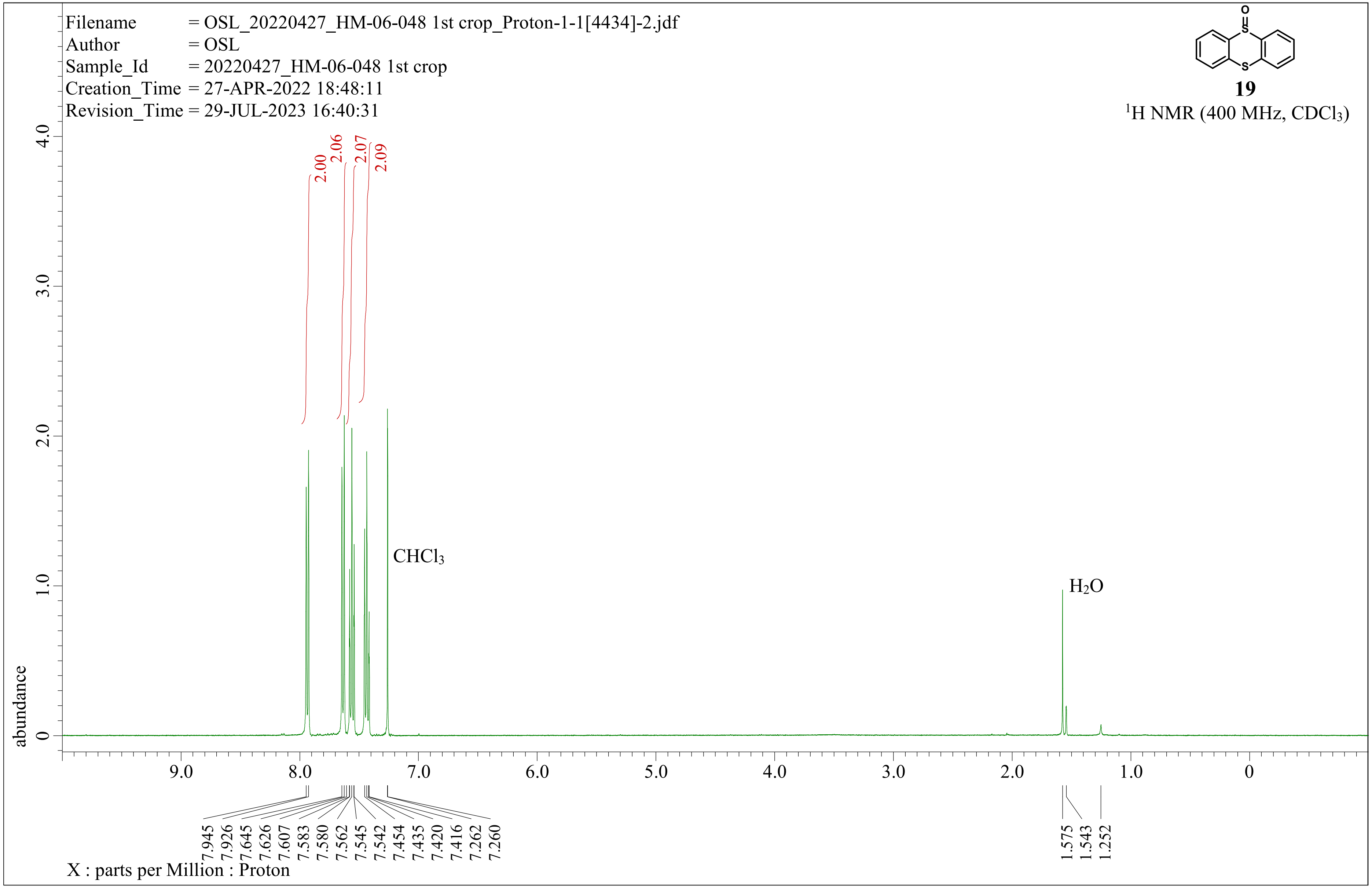

Supplementary Fig. 31. ^{1}H NMR spectrum of compound **19**, recorded at 400 MHz and 298 K in $CDCl_3$.

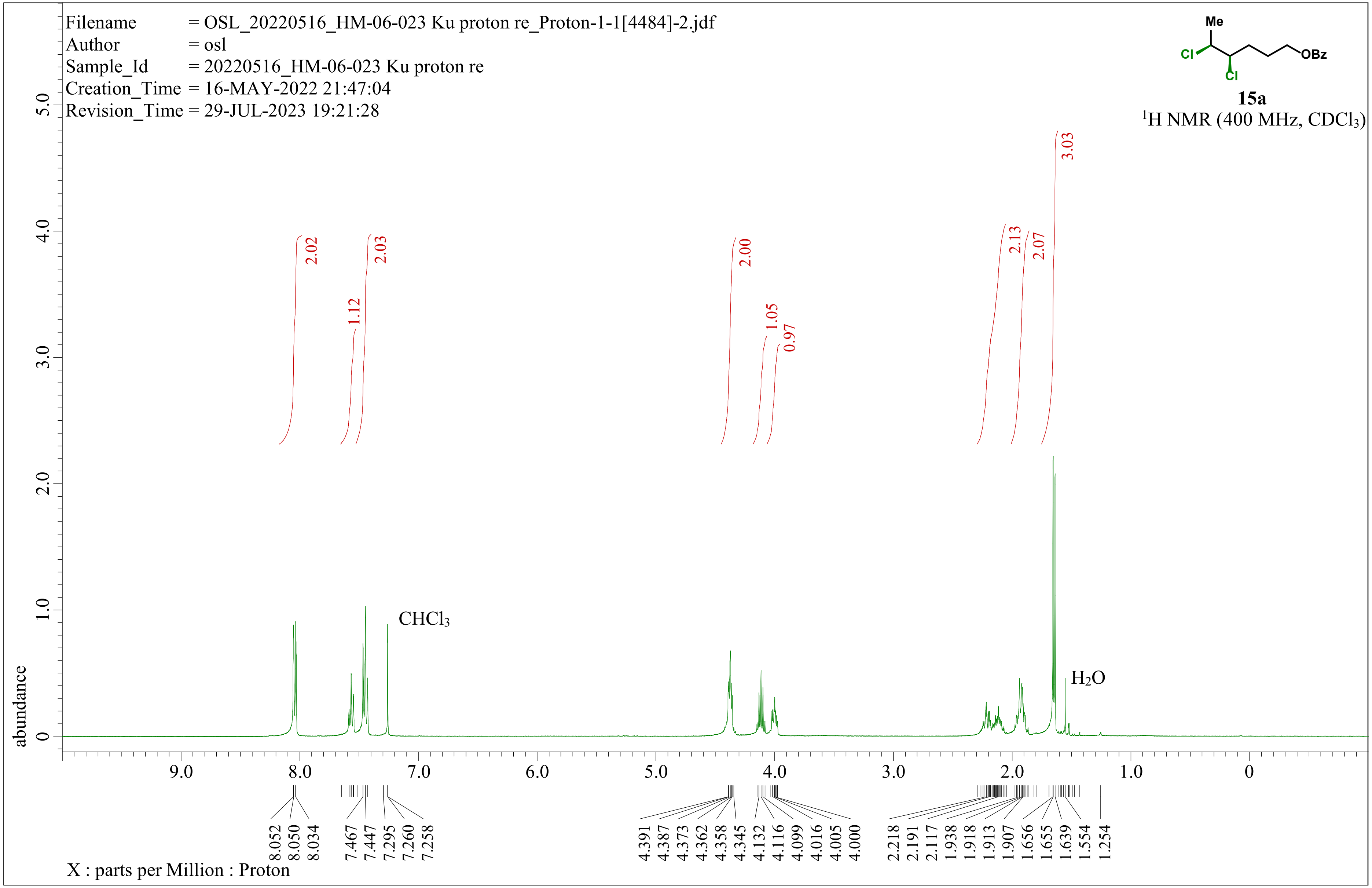

Supplementary Fig. 32. ^{1}H NMR spectrum of compound **15a**, recorded at 400 MHz and 298 K in $CDCl_3$.

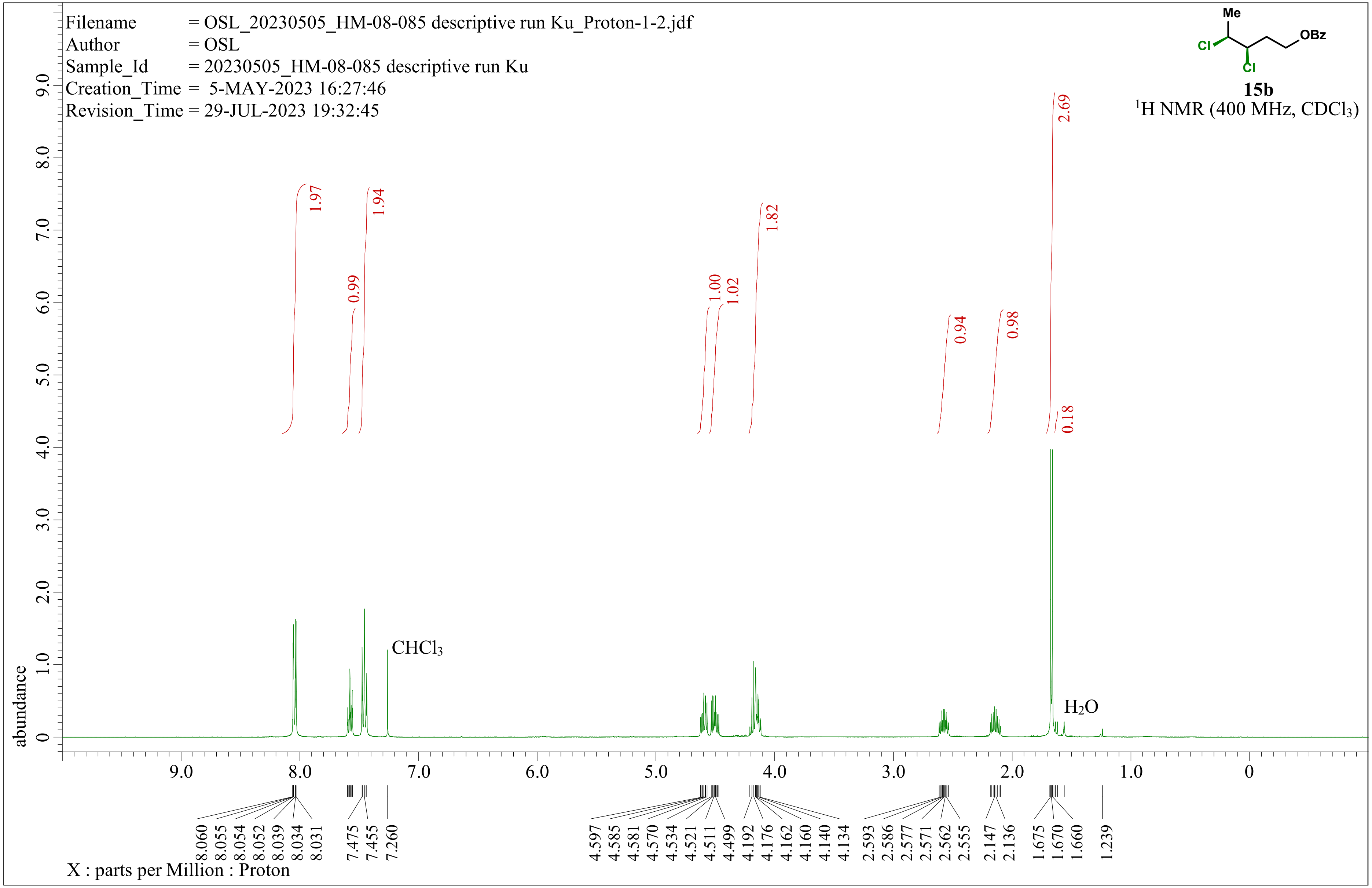

Supplementary Fig. 33. ^{1}H NMR spectrum of compound **15b**, recorded at 400 MHz and 298 K in $CDCl_3$.

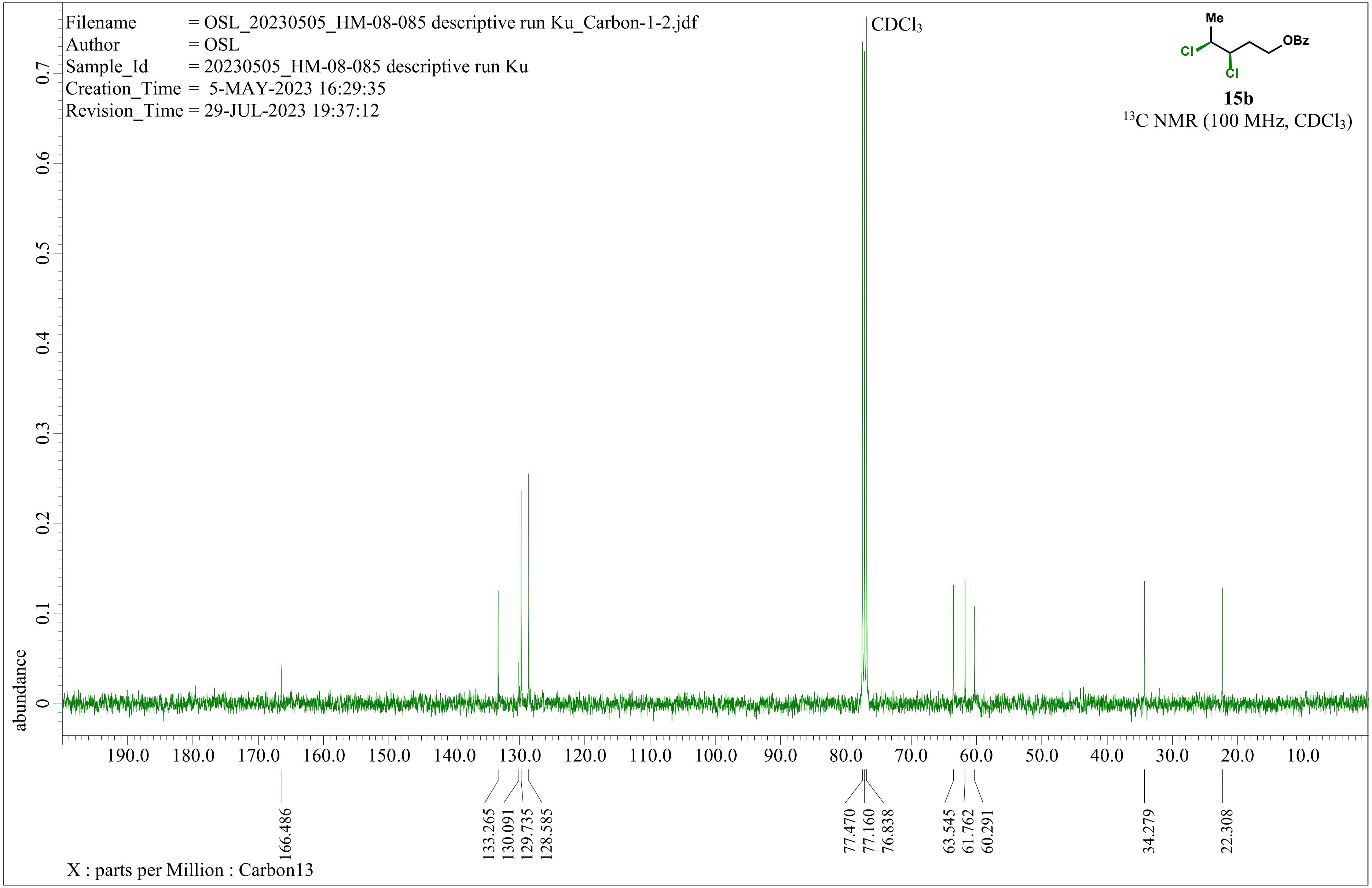

Supplementary Fig. 34. ^{13}C NMR spectrum of compound **15b**, recorded at 100 MHz and 298 K in $CDCl_3$.

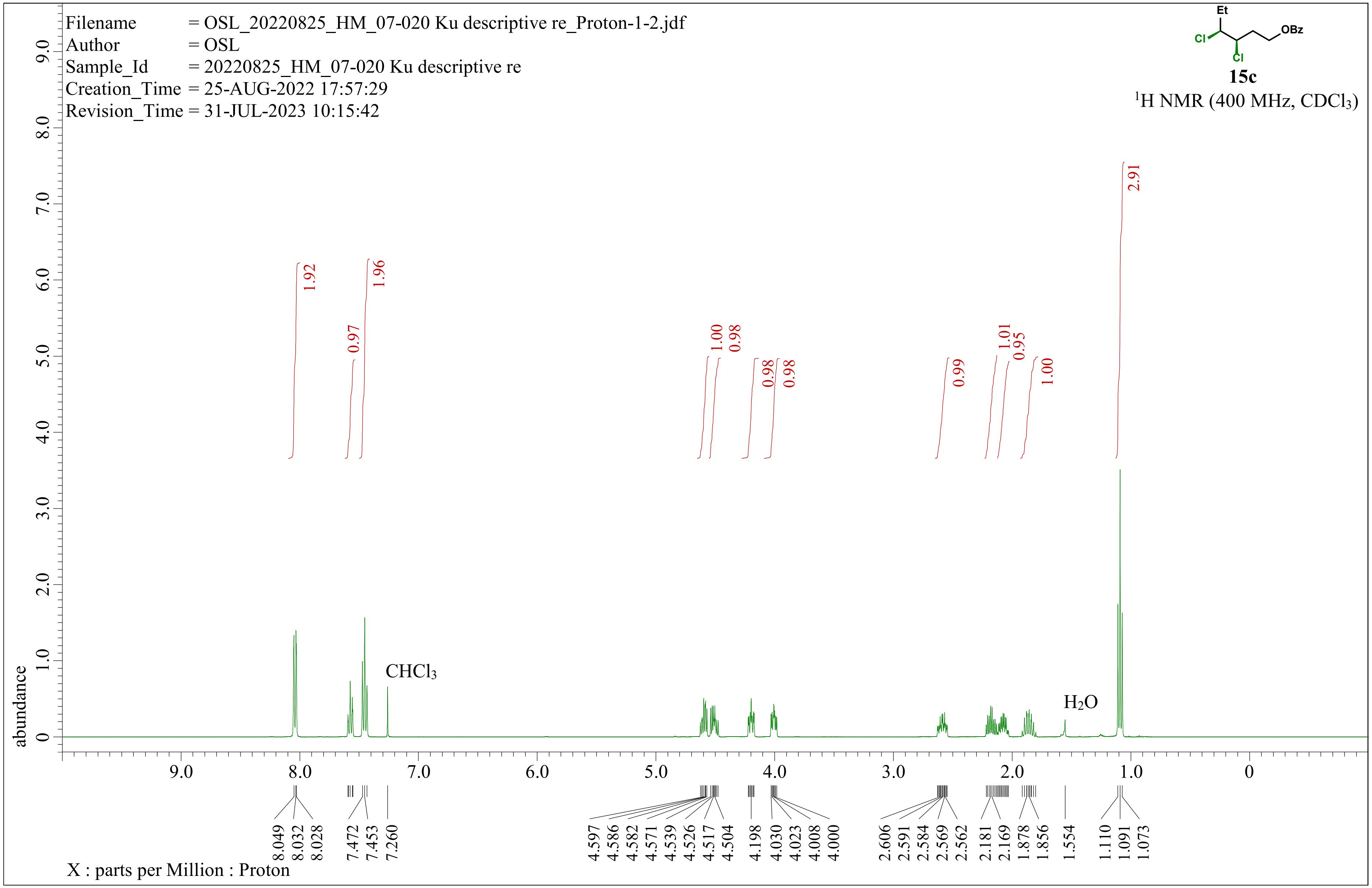

Supplementary Fig. 35. ^1H NMR spectrum of compound **15c**, recorded at 400 MHz and 298 K in $CDCl_3$.

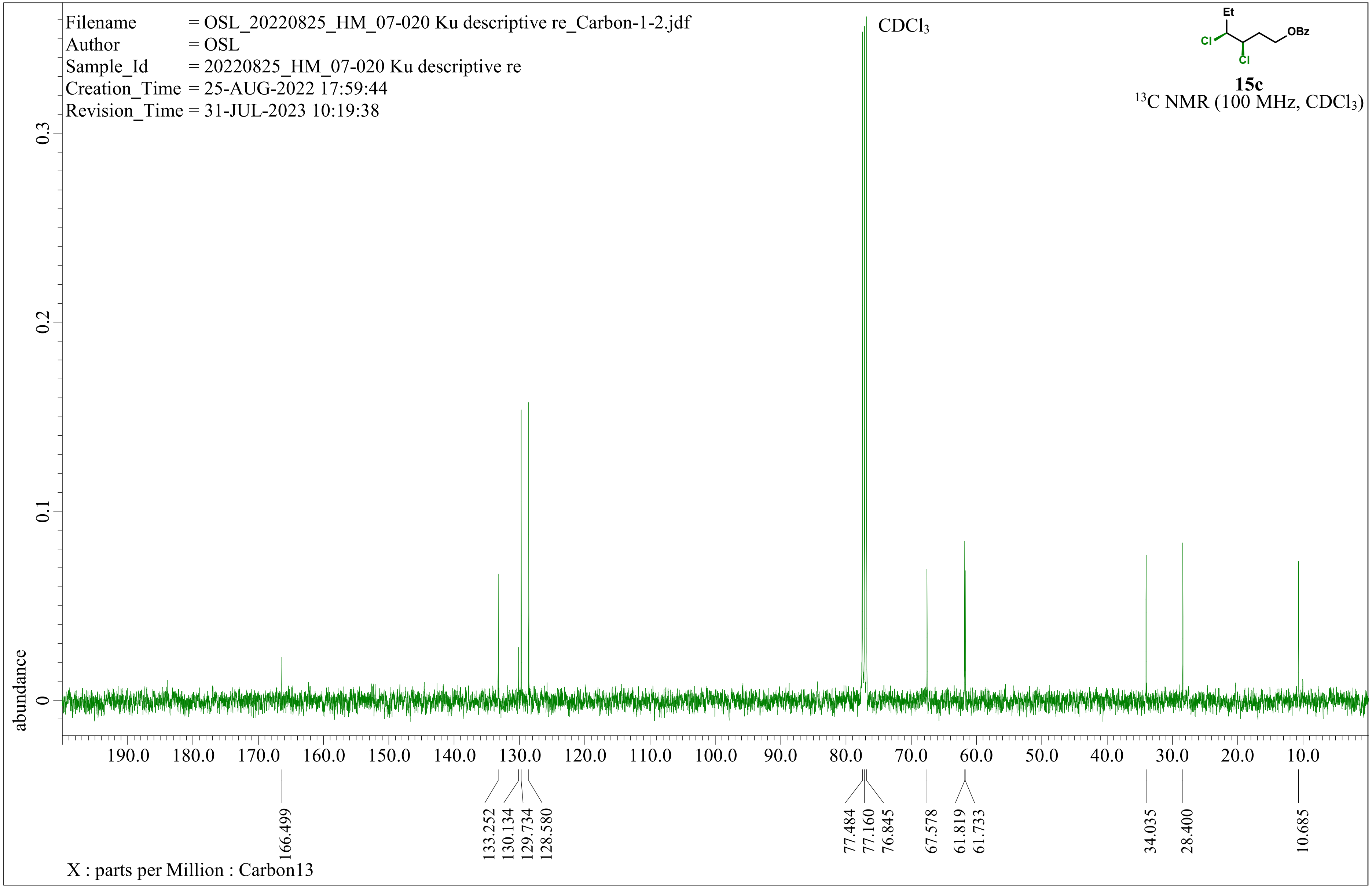

Supplementary Fig. 36. ^{13}C NMR spectrum of compound **15c**, recorded at 100 MHz and 298 K in $CDCl_3$.

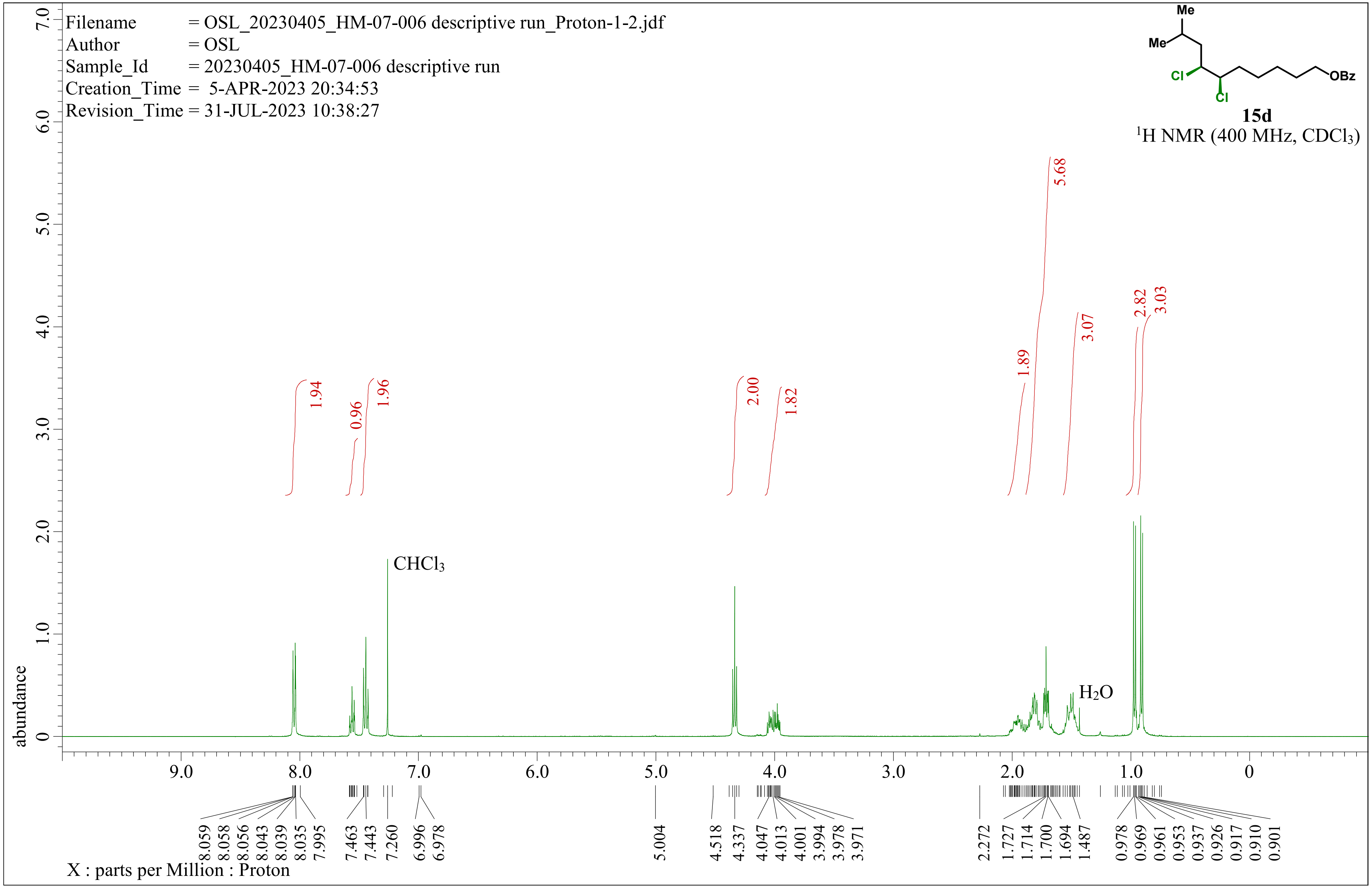

Supplementary Fig. 37. ^{1}H NMR spectrum of compound **15d**, recorded at 400 MHz and 298 K in $CDCl_3$.

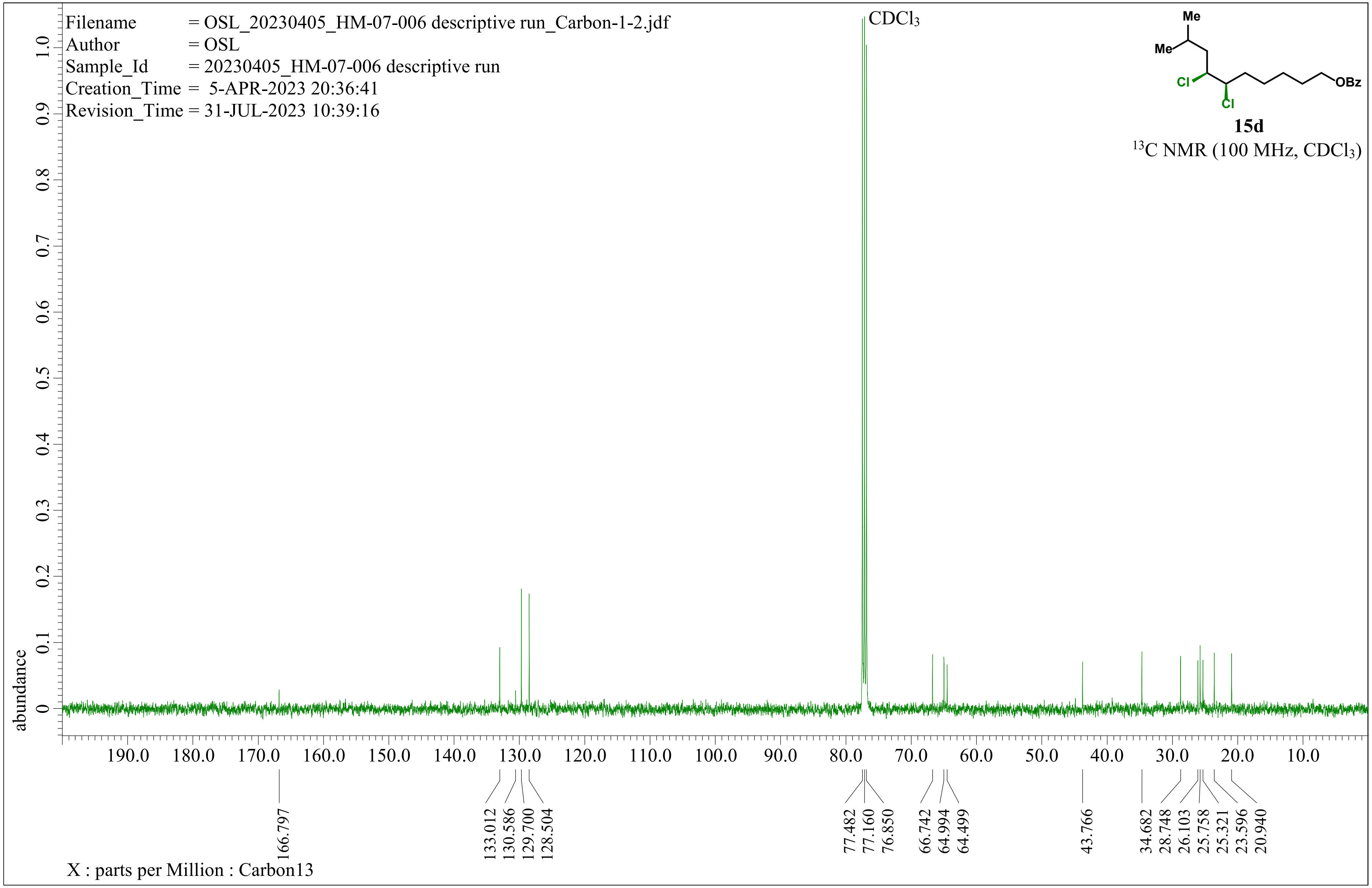

Supplementary Fig. 38. ^{13}C NMR spectrum of compound **15d**, recorded at 100 MHz and 298 K in $CDCl_3$.

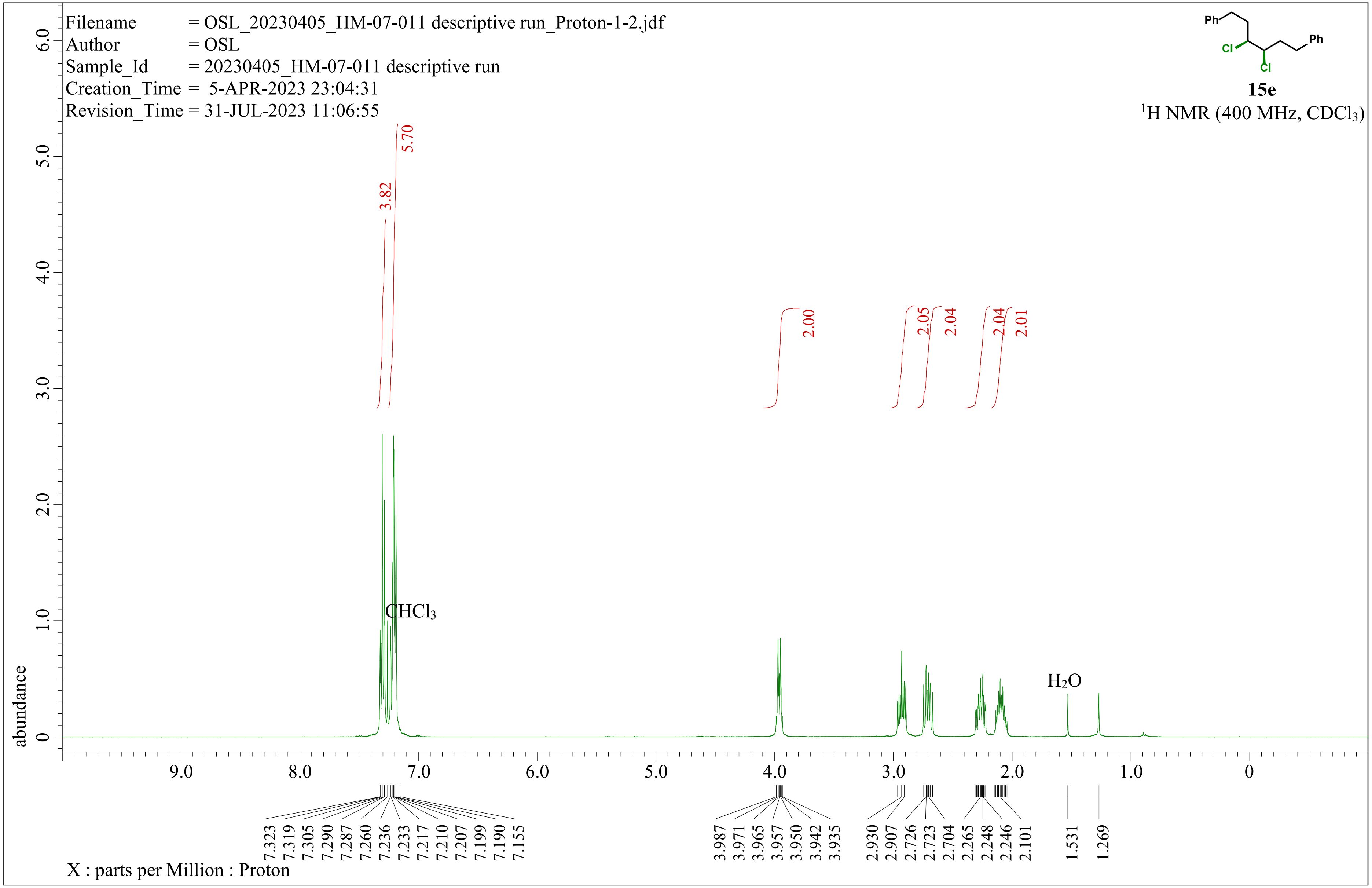

Supplementary Fig. 39. ^{1}H NMR spectrum of compound **15e**, recorded at 400 MHz and 298 K in $CDCl_3$.

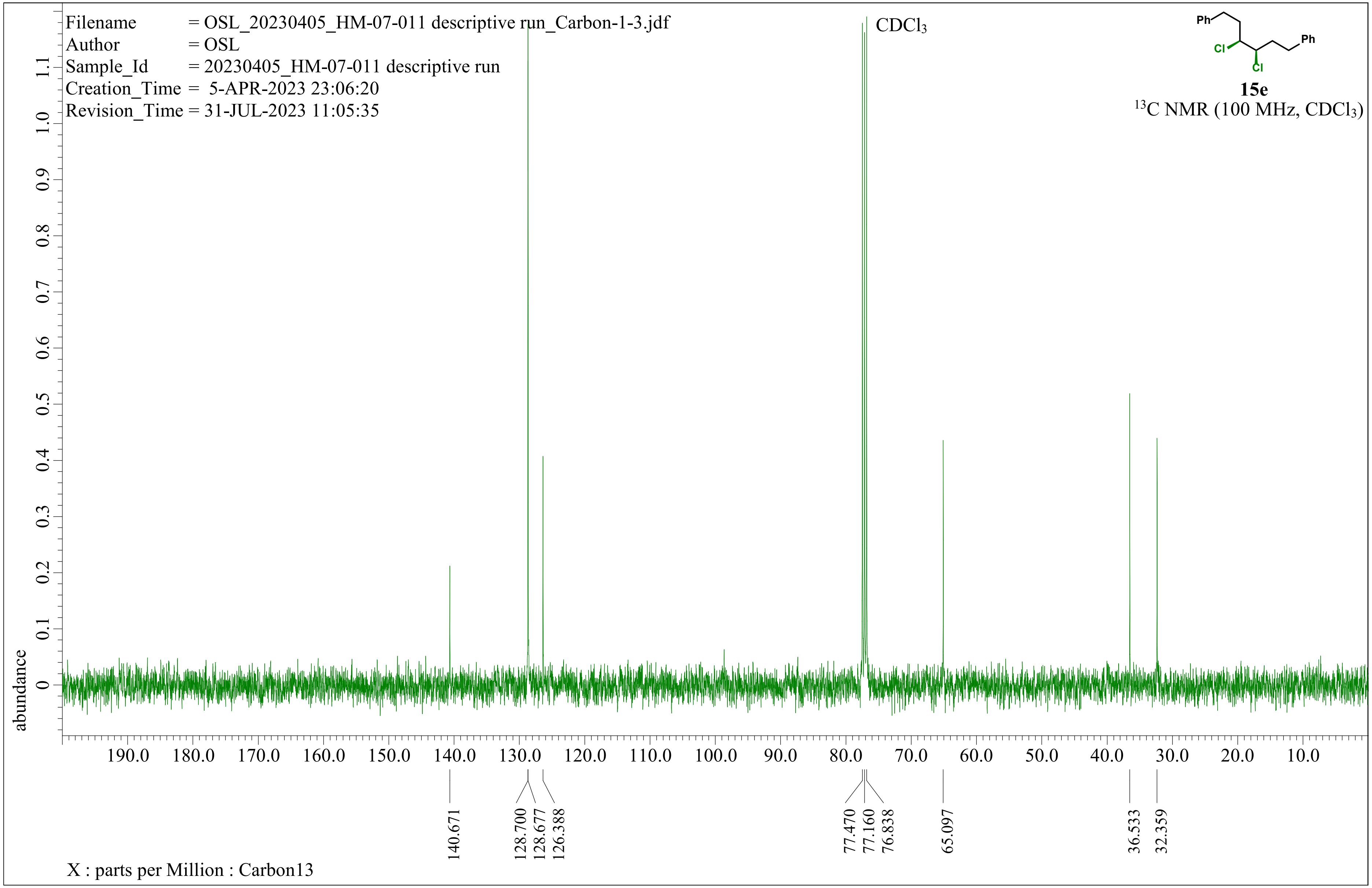

Supplementary Fig. 40. ^{13}C NMR spectrum of compound **15e**, recorded at 100 MHz and 298 K in $CDCl_3$.

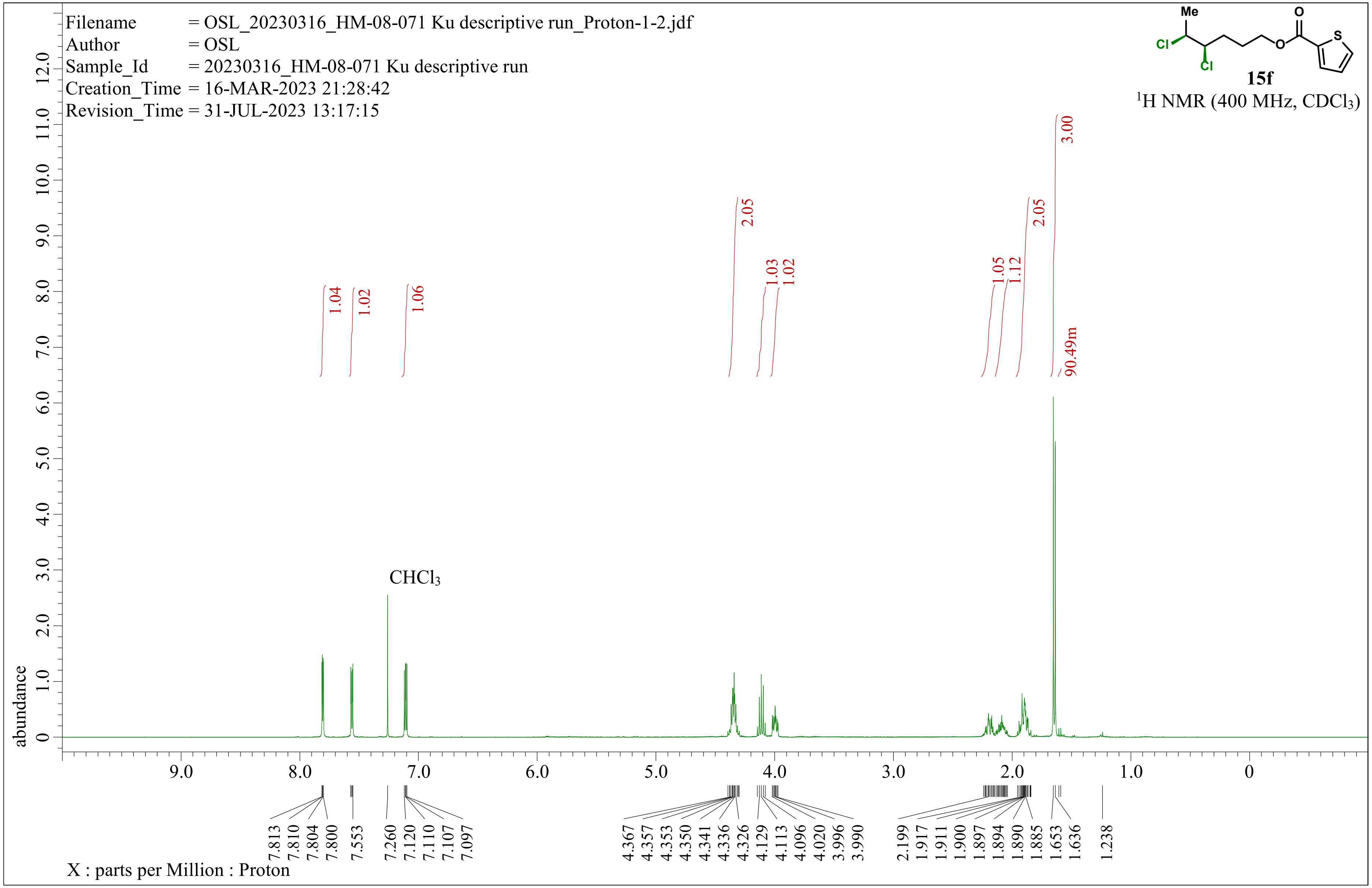

Supplementary Fig. 41. ^{1}H NMR spectrum of compound **15f**, recorded at 400 MHz and 298 K in $CDCl_3$.

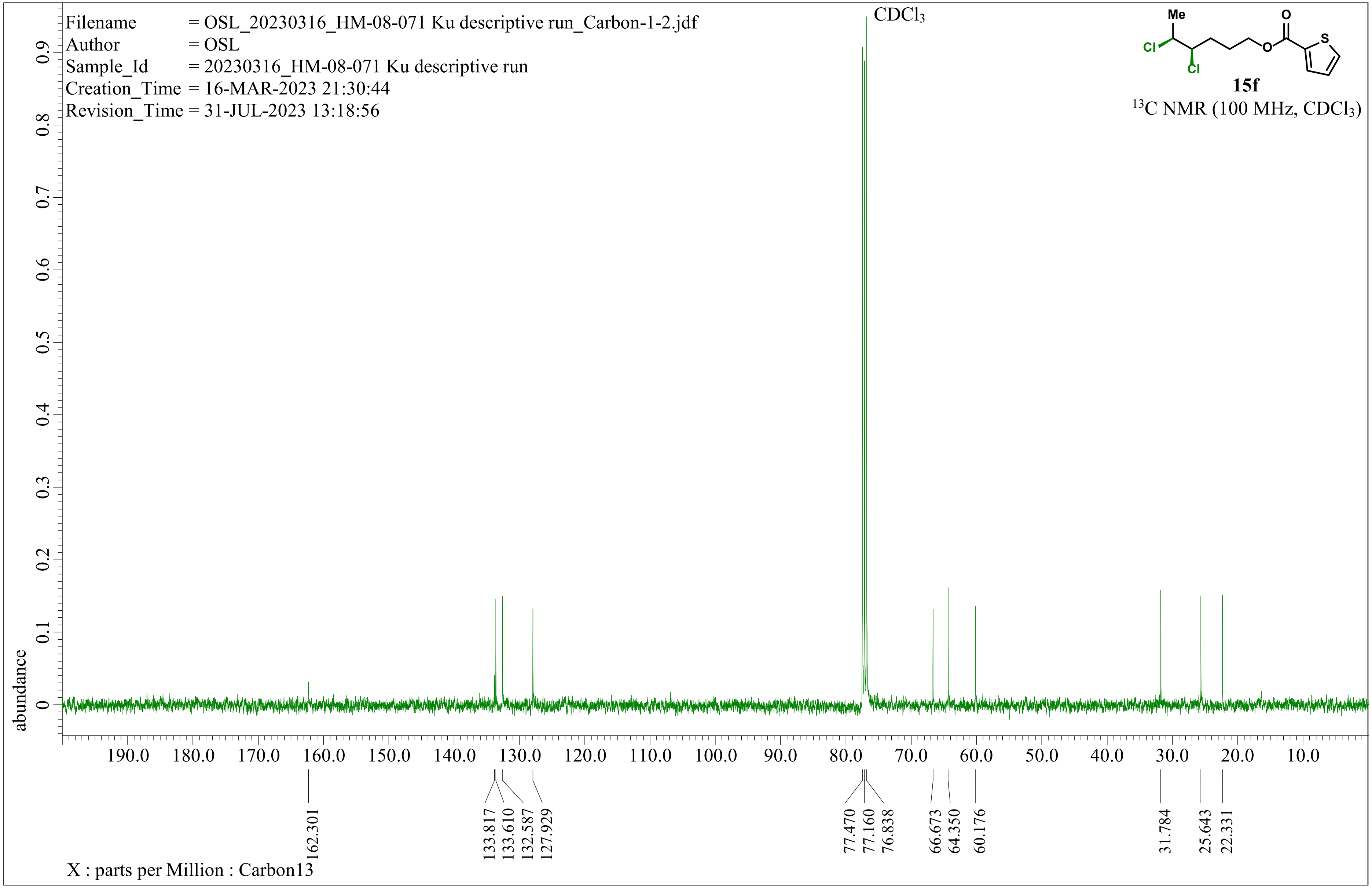

Supplementary Fig. 42. ^{13}C NMR spectrum of compound **15f**, recorded at 100 MHz and 298 K in $CDCl_3$.

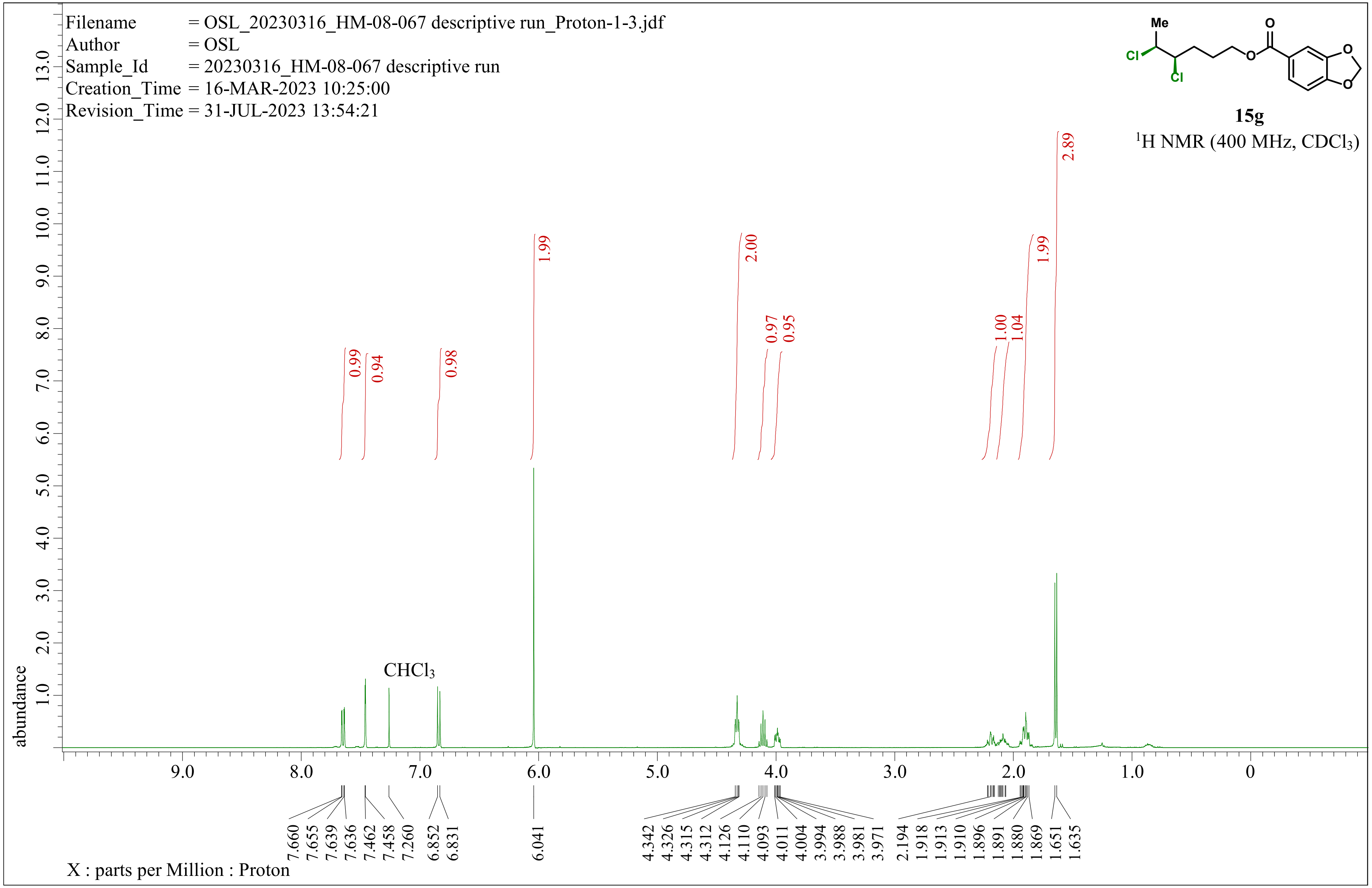

Supplementary Fig. 43. ^{1}H NMR spectrum of compound **15g**, recorded at 400 MHz and 298 K in $CDCl_3$.

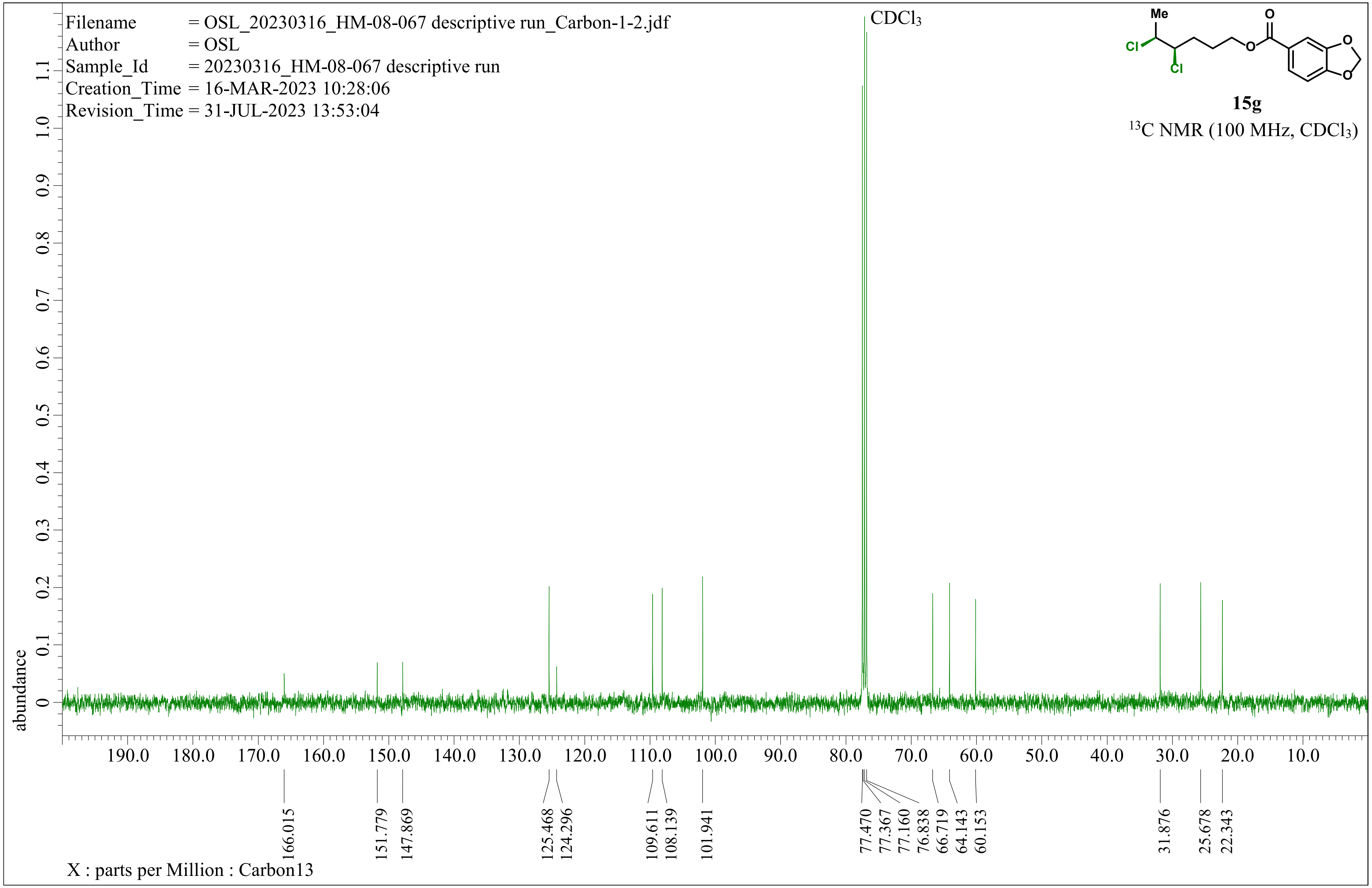

Supplementary Fig. 44. ^{13}C NMR spectrum of compound **15g**, recorded at 100 MHz and 298 K in $CDCl_3$.

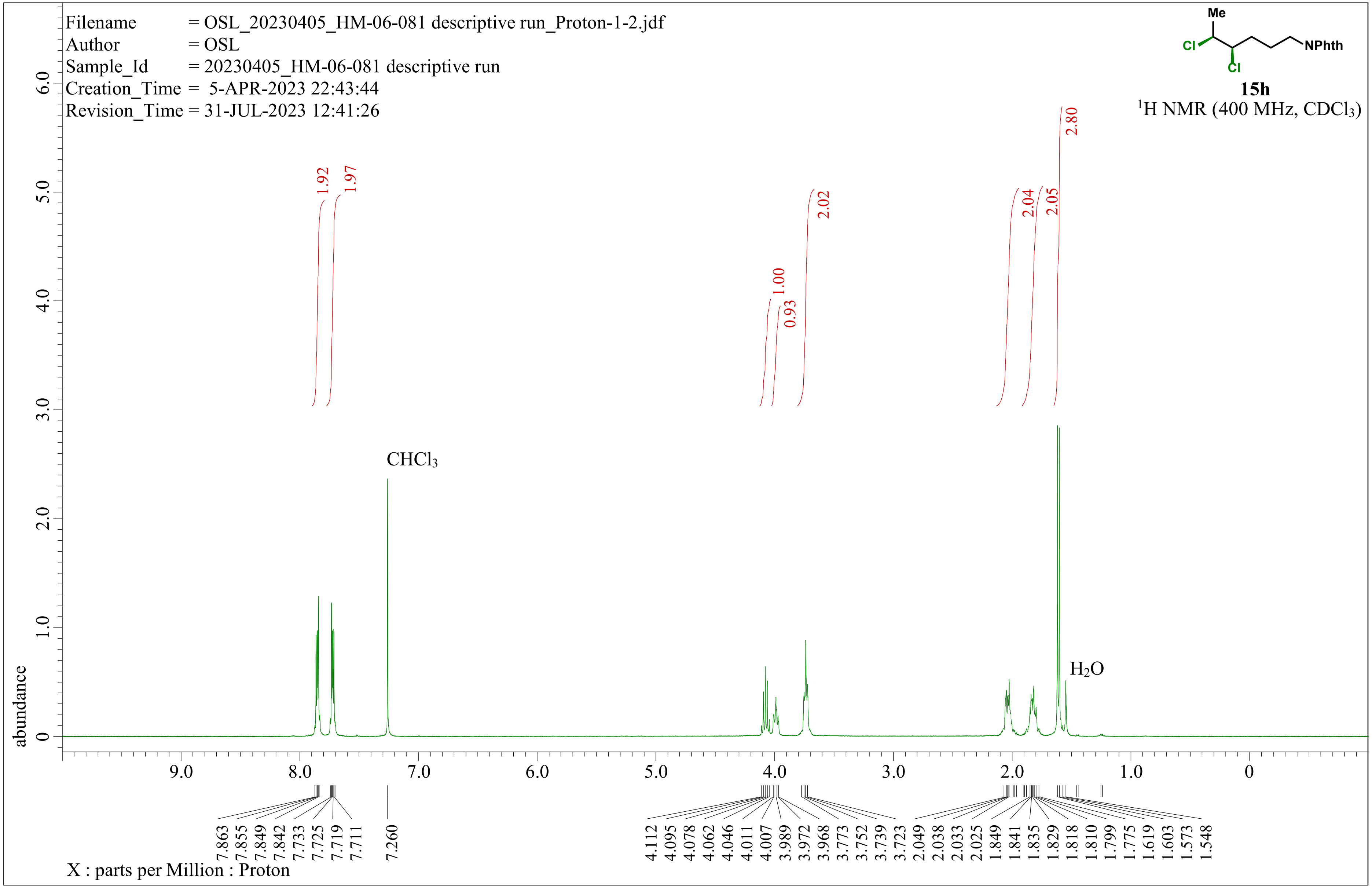

Supplementary Fig. 45. ^{1}H NMR spectrum of compound **15h**, recorded at 400 MHz and 298 K in $CDCl_3$.

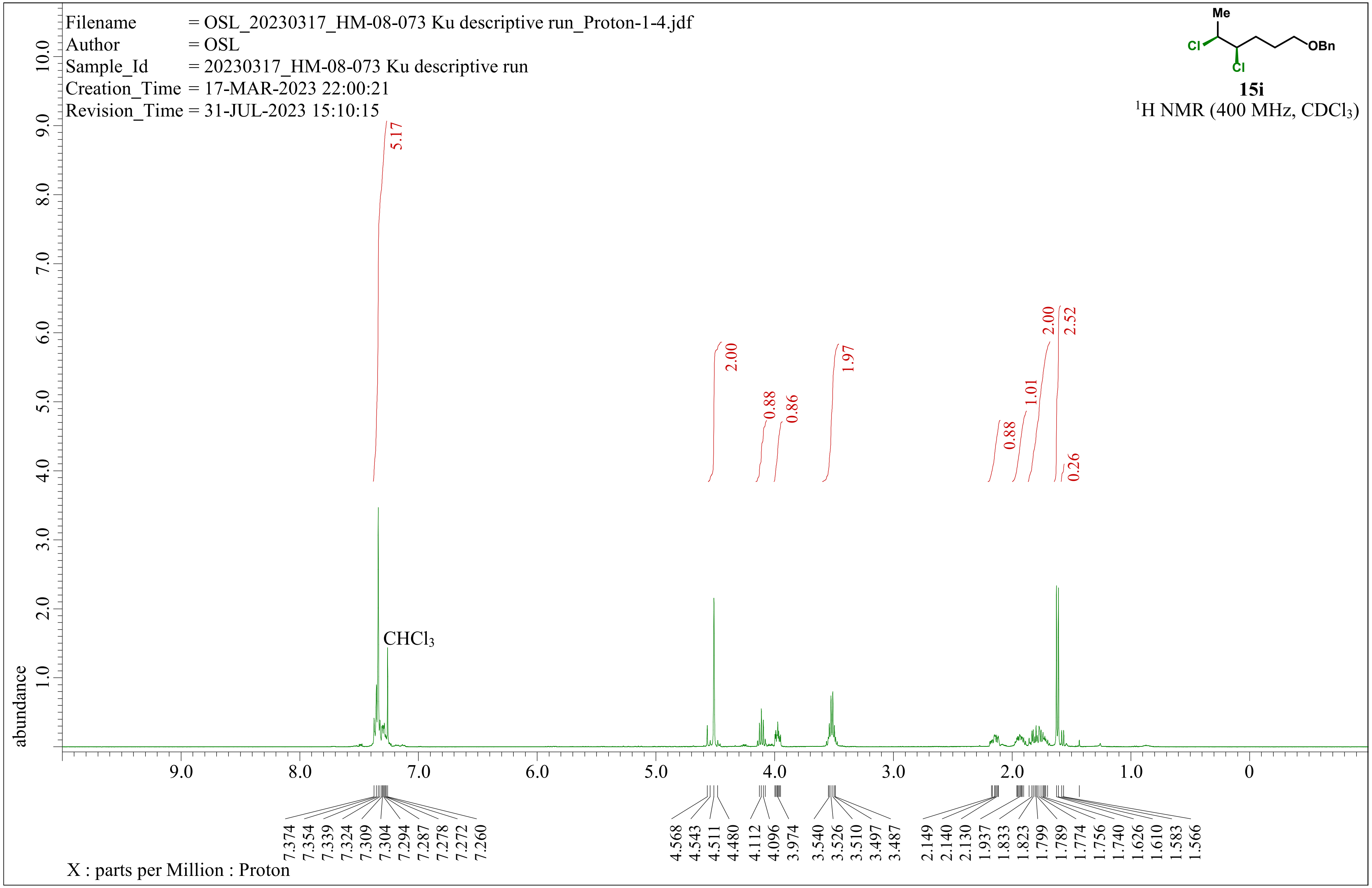

Supplementary Fig. 46. ^{1}H NMR spectrum of compound **15i**, recorded at 400 MHz and 298 K in $CDCl_3$.

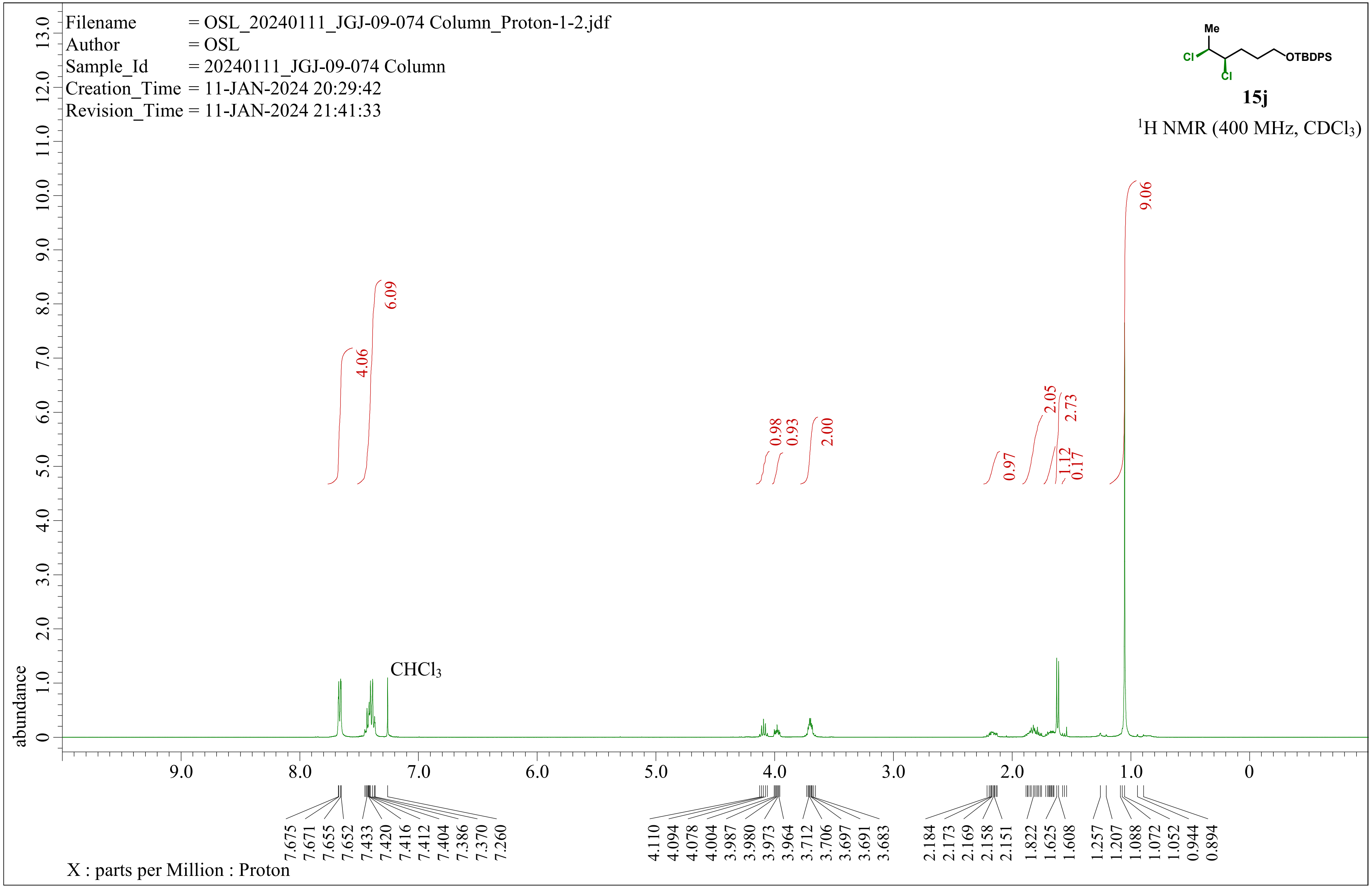

Supplementary Fig. 47. ^{1}H NMR spectrum of compound **15j**, recorded at 400 MHz and 298 K in $CDCl_3$.

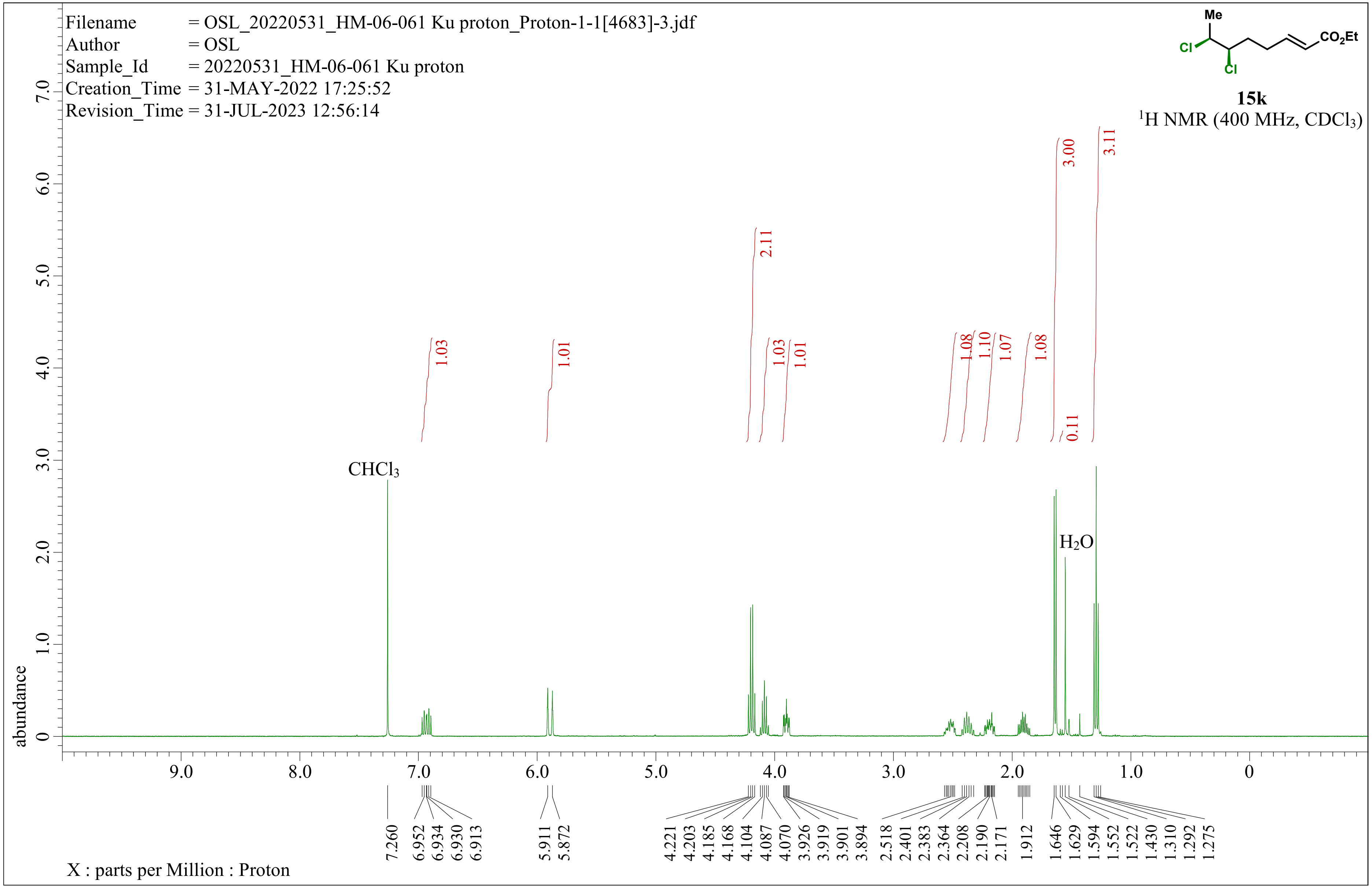

Supplementary Fig. 48. ^{1}H NMR spectrum of compound **15k**, recorded at 400 MHz and 298 K in $CDCl_3$.

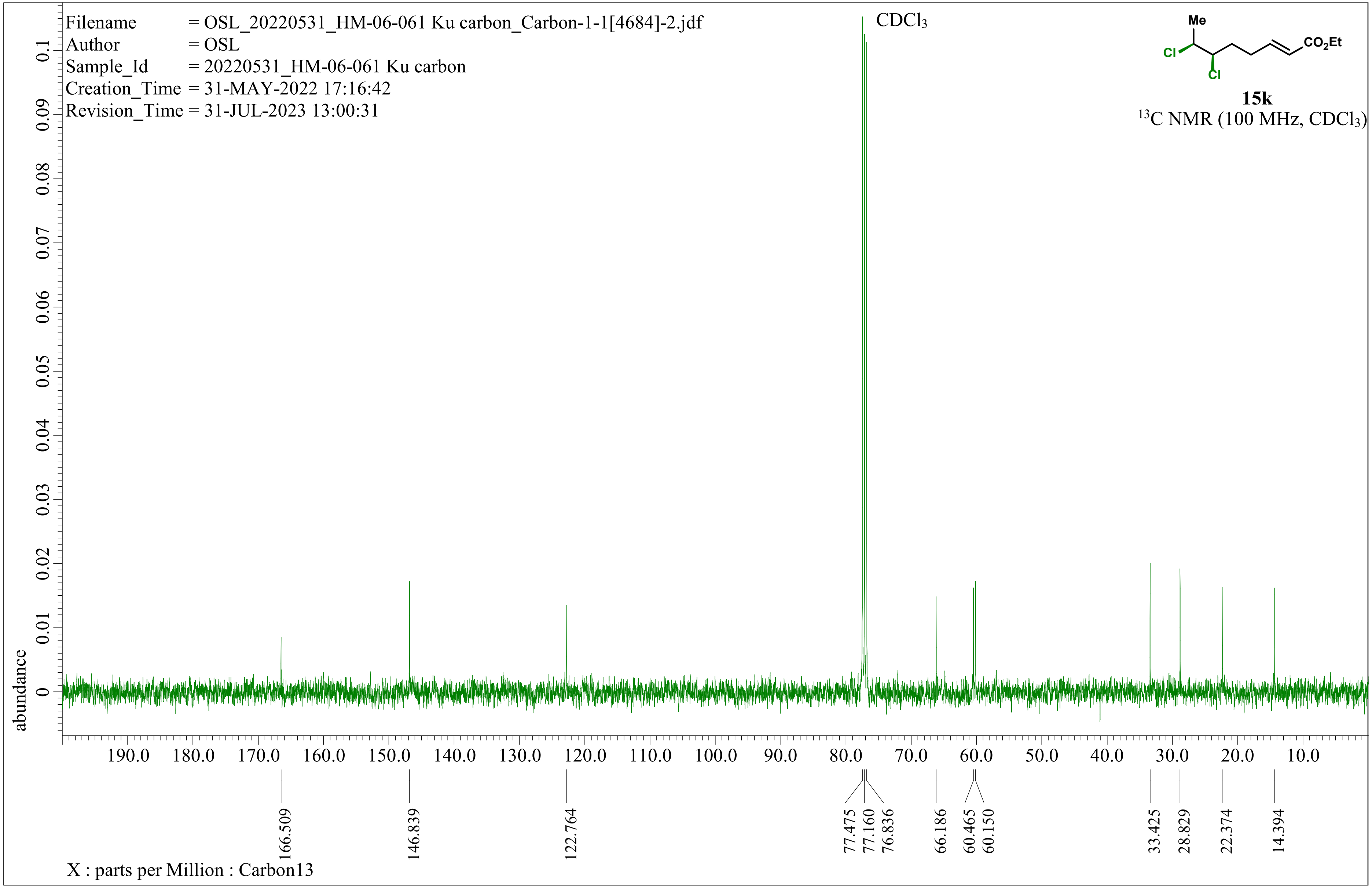

Supplementary Fig. 49. ^{13}C NMR spectrum of compound **15k**, recorded at 100 MHz and 298 K in $CDCl_3$.

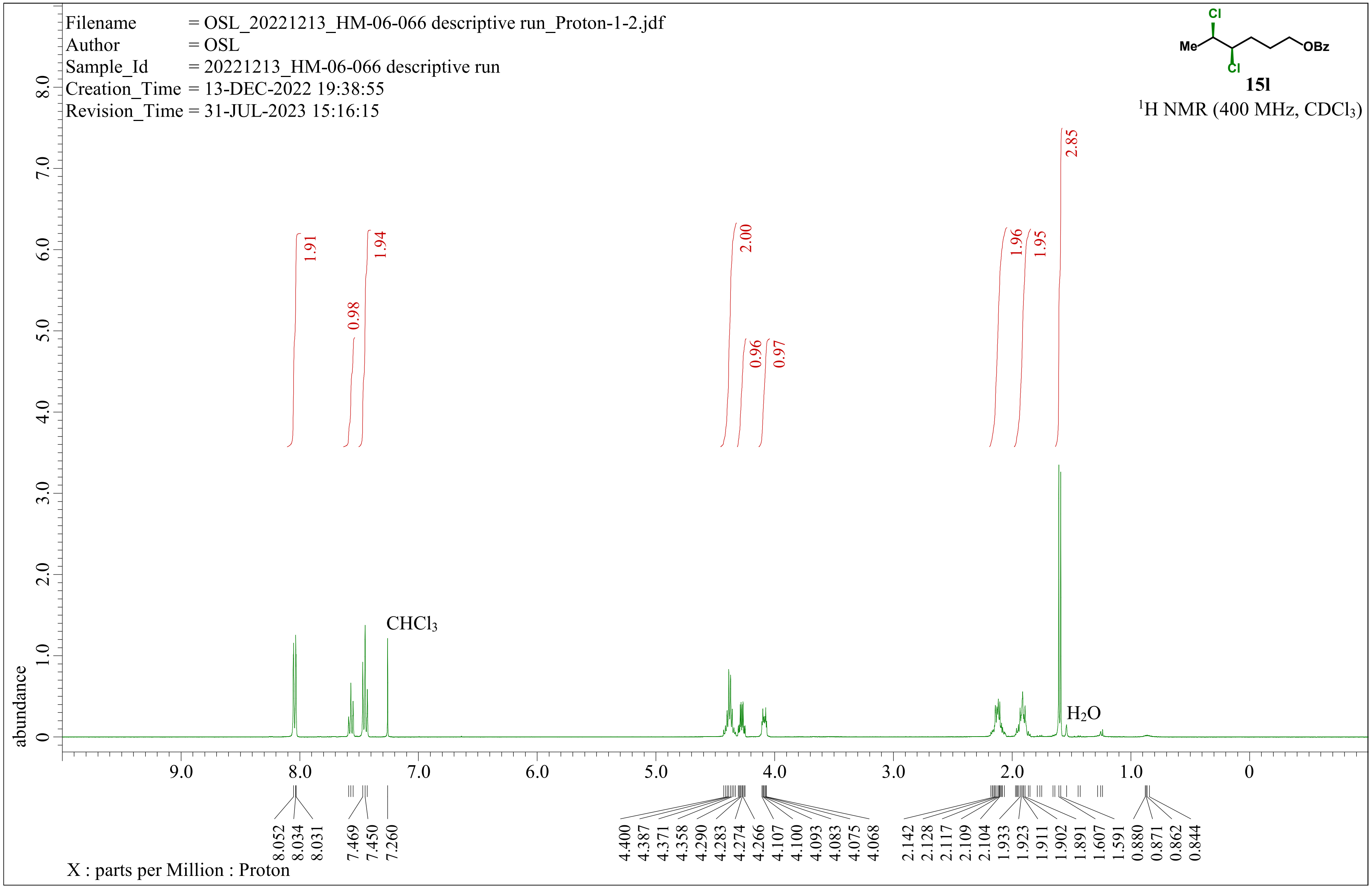

Supplementary Fig. 50. ^{1}H NMR spectrum of compound **15l**, recorded at 400 MHz and 298 K in $CDCl_3$.

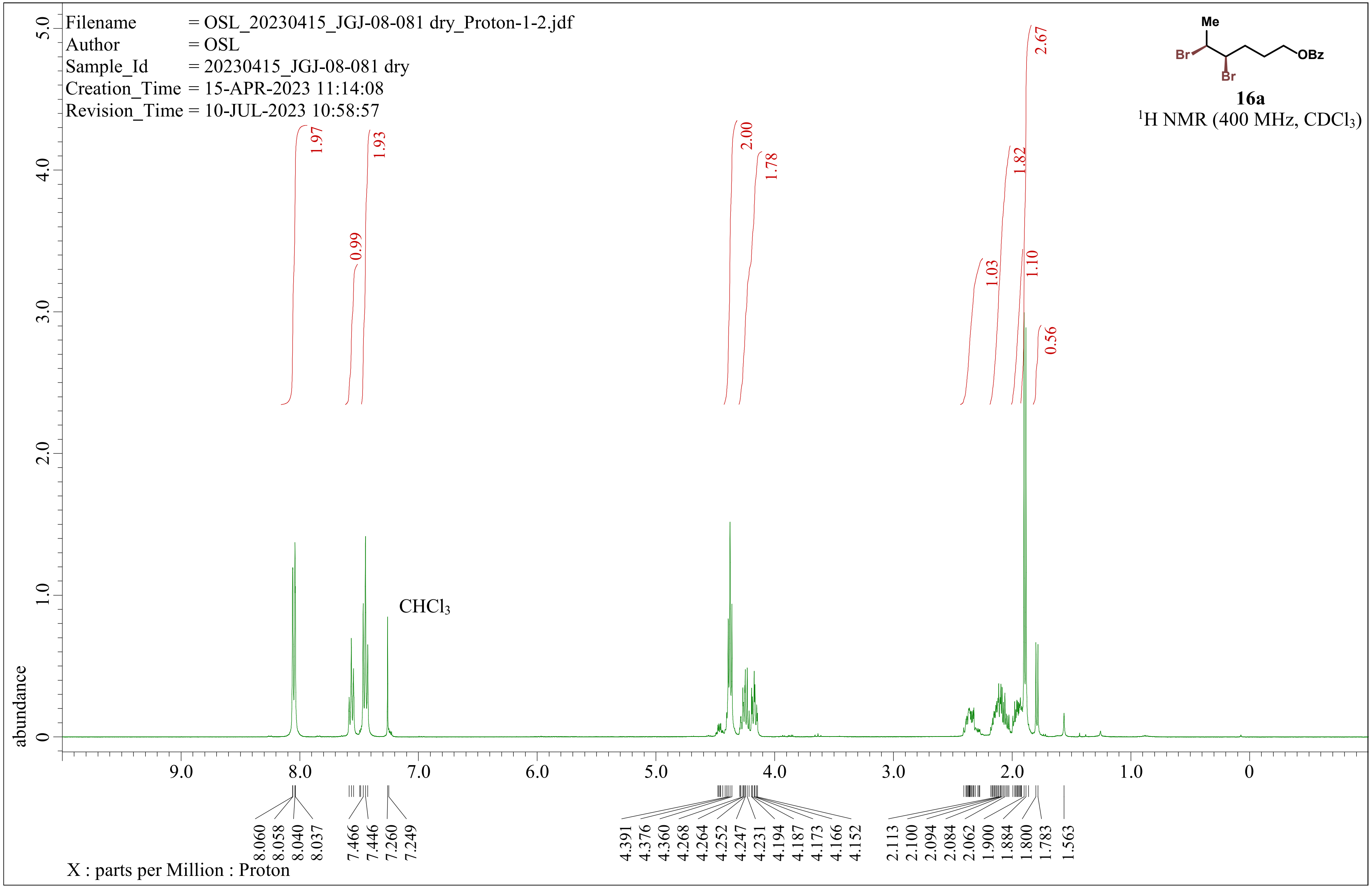

Supplementary Fig. 51. ^{1}H NMR spectrum of compound **16a**, recorded at 400 MHz and 298 K in $CDCl_3$.

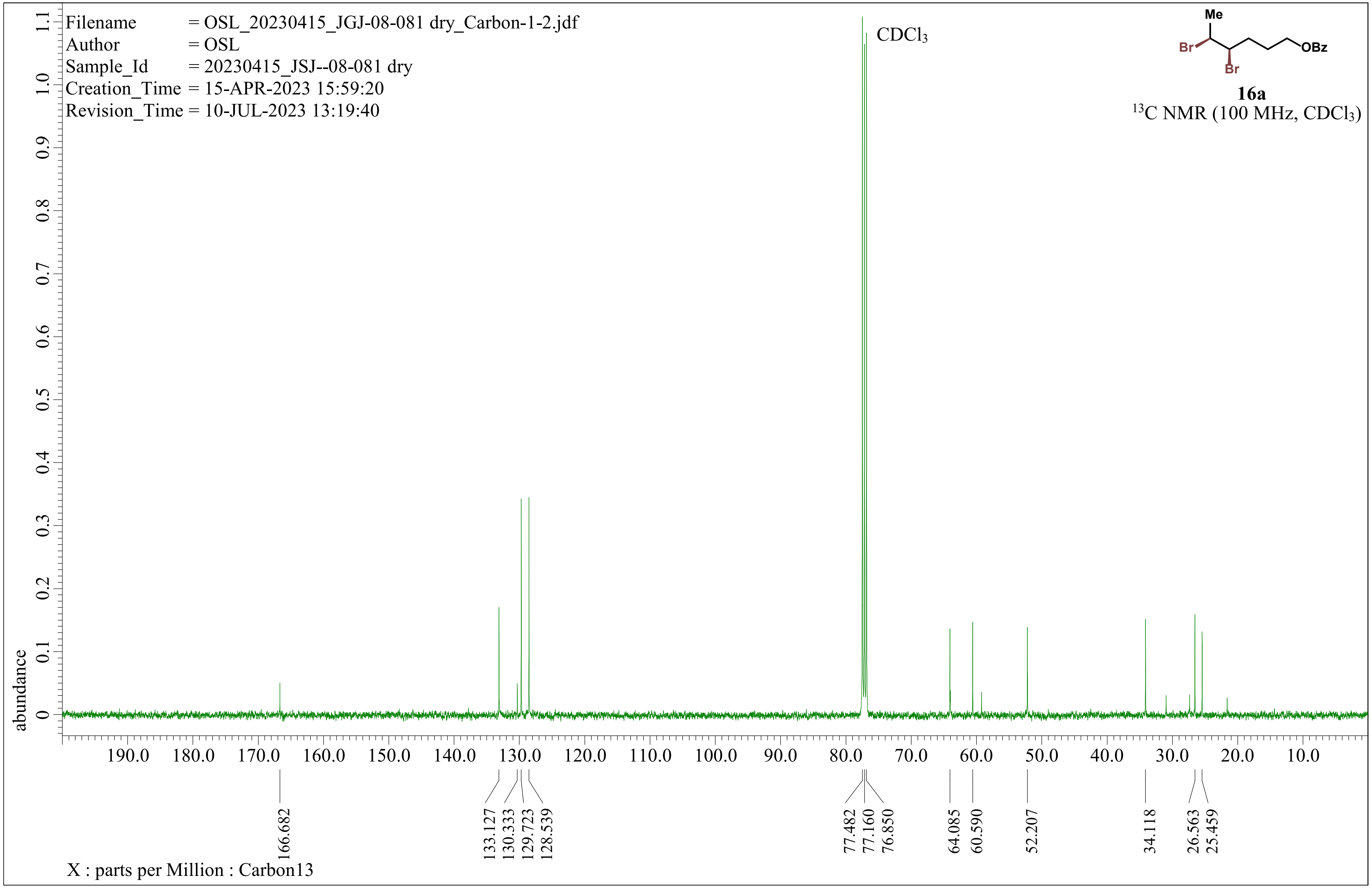

Supplementary Fig. 52. ^{13}C NMR spectrum of compound **16a**, recorded at 100 MHz and 298 K in $CDCl_3$.

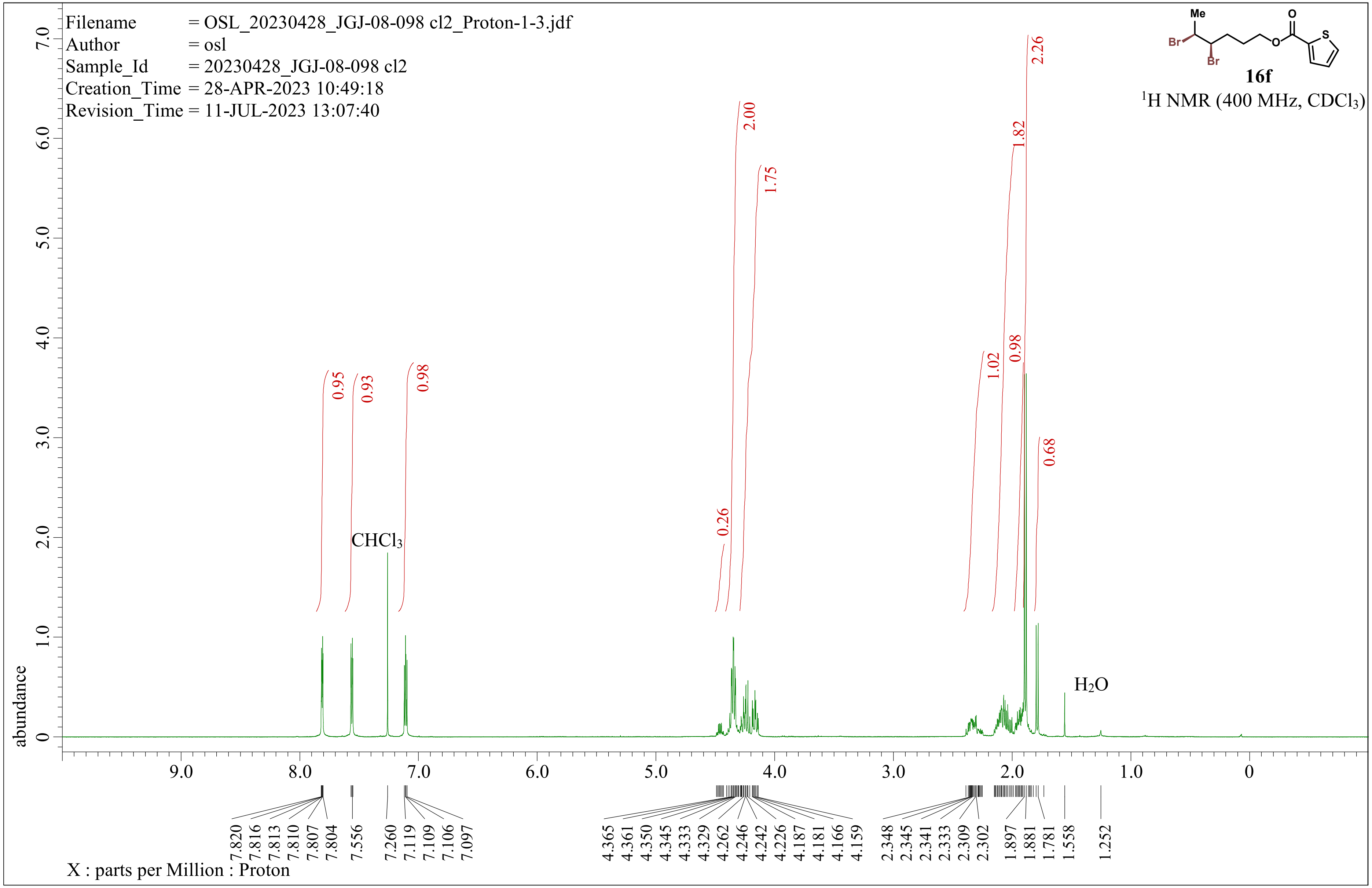

Supplementary Fig. 53. ^{1}H NMR spectrum of compound **16f**, recorded at 400 MHz and 298 K in $CDCl_3$.

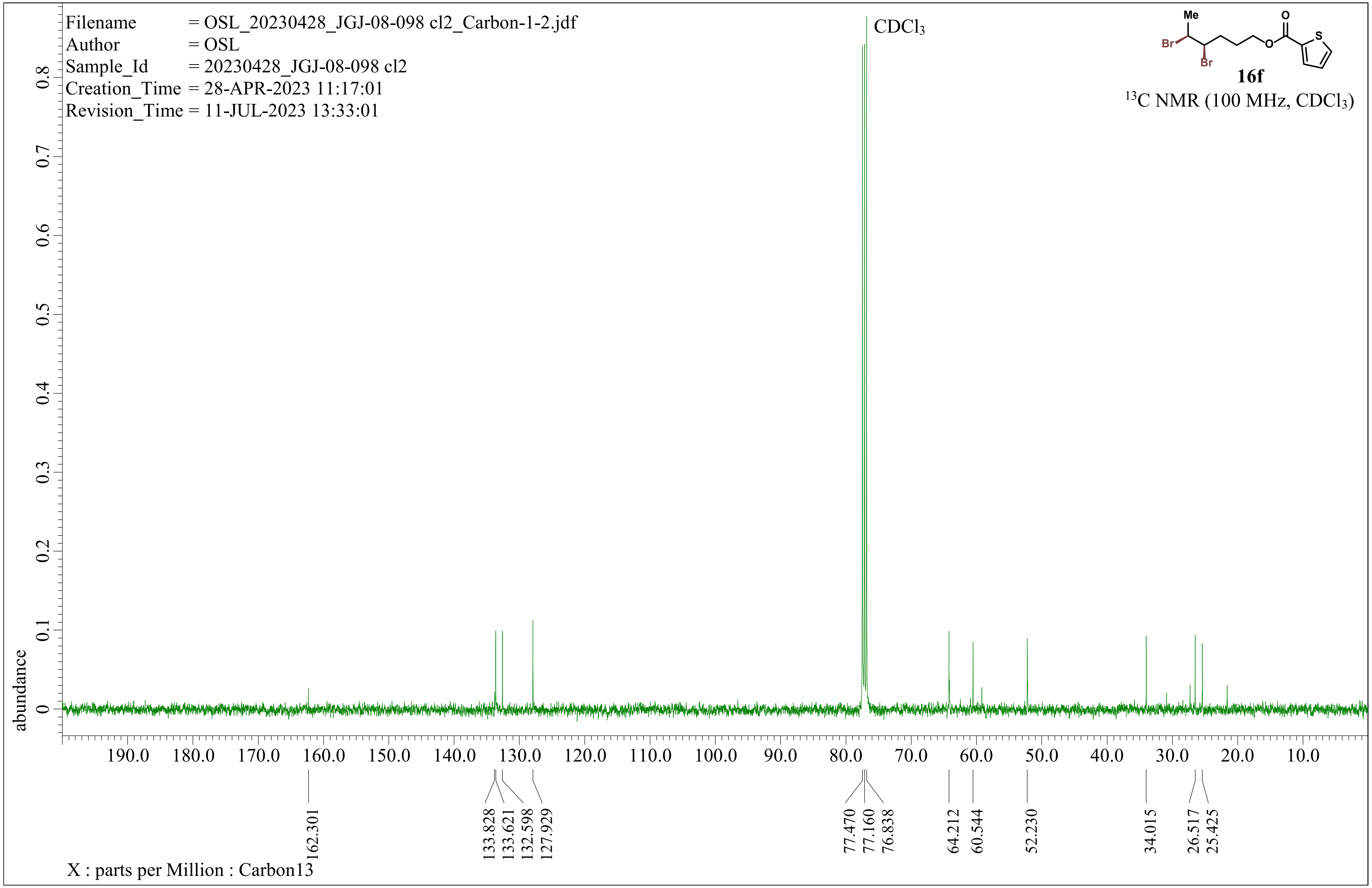

Supplementary Fig. 54. ^{13}C NMR spectrum of compound **16f**, recorded at 100 MHz and 298 K in $CDCl_3$.

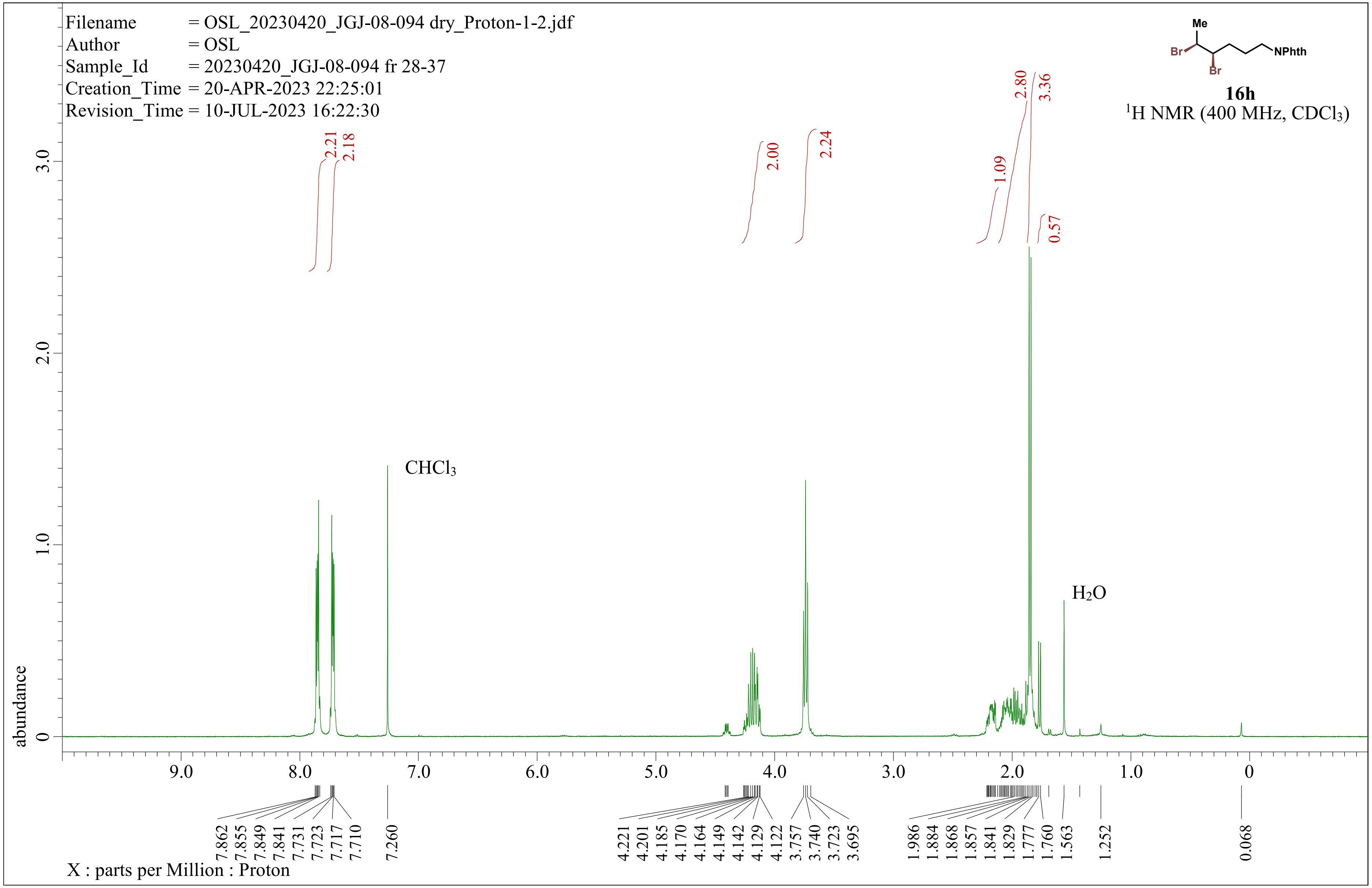

Supplementary Fig. 55. ^{1}H NMR spectrum of compound **16h**, recorded at 400 MHz and 298 K in $CDCl_3$.

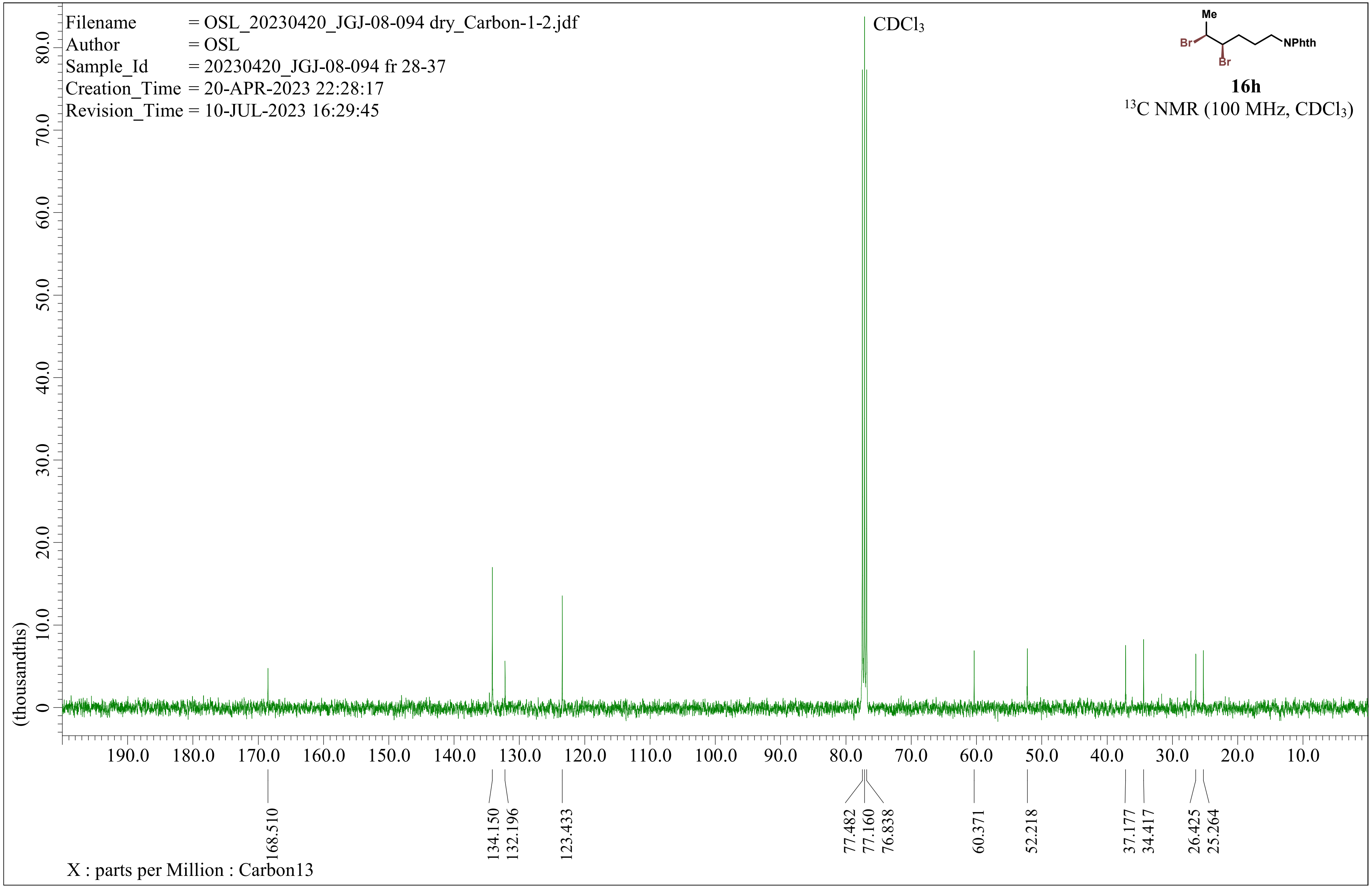

Supplementary Fig. 56. ^{13}C NMR spectrum of compound **16h**, recorded at 100 MHz and 298 K in $CDCl_3$.

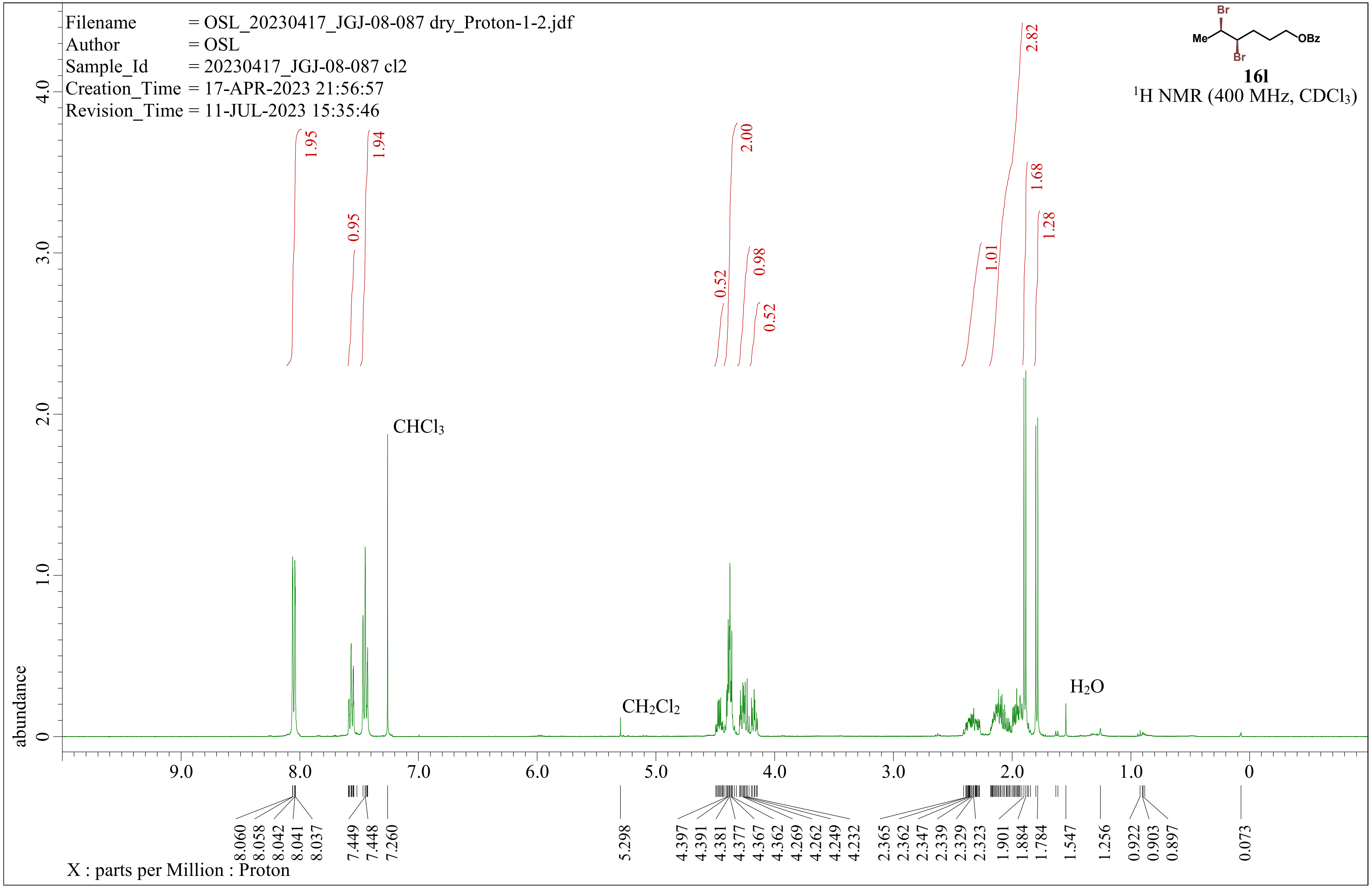

Supplementary Fig. 57. ^{1}H NMR spectrum of compound **16l**, recorded at 400 MHz and 298 K in $CDCl_3$.

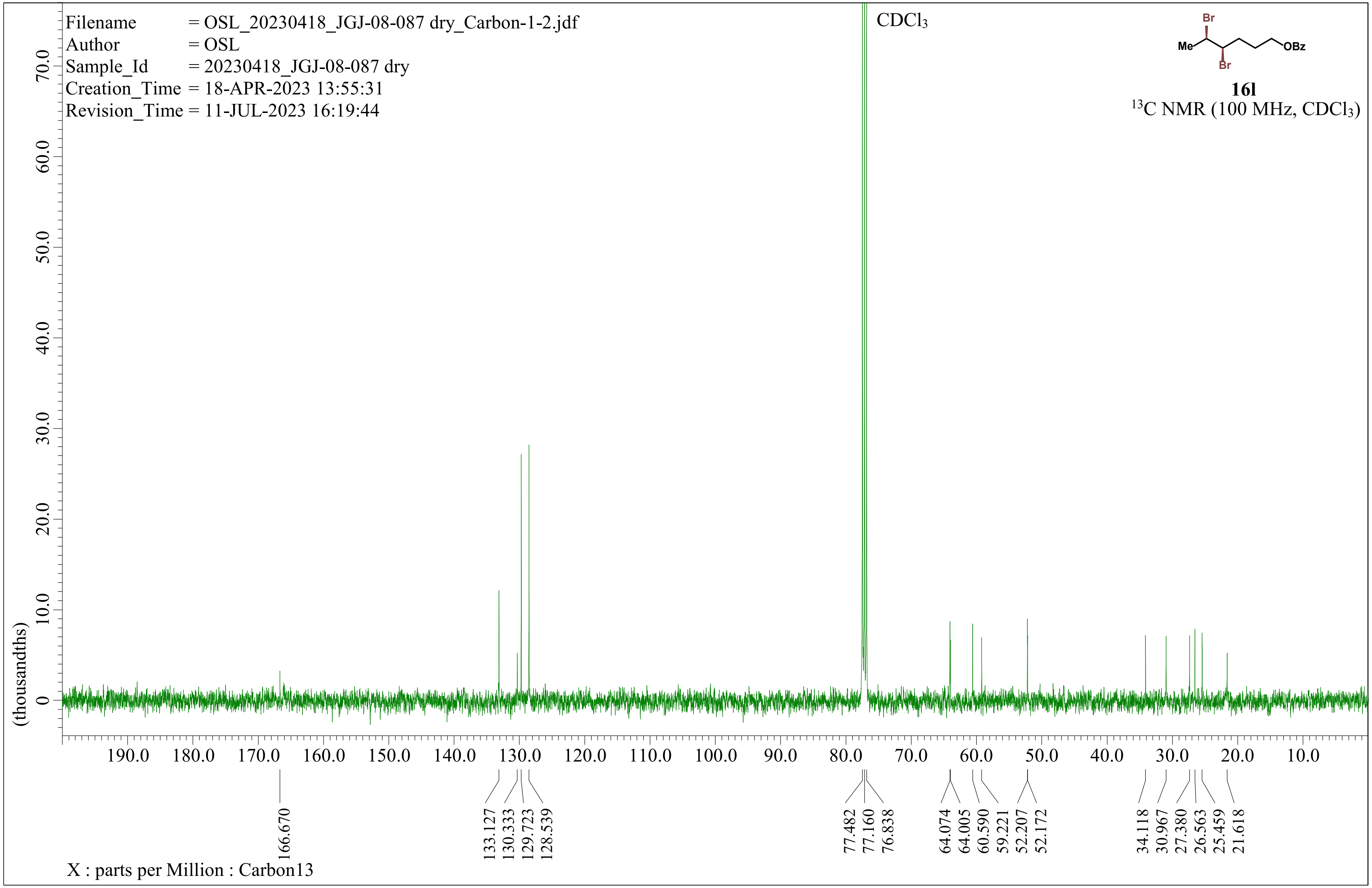

Supplementary Fig. 58. ^{13}C NMR spectrum of compound **16l**, recorded at 100 MHz and 298 K in $CDCl_3$.

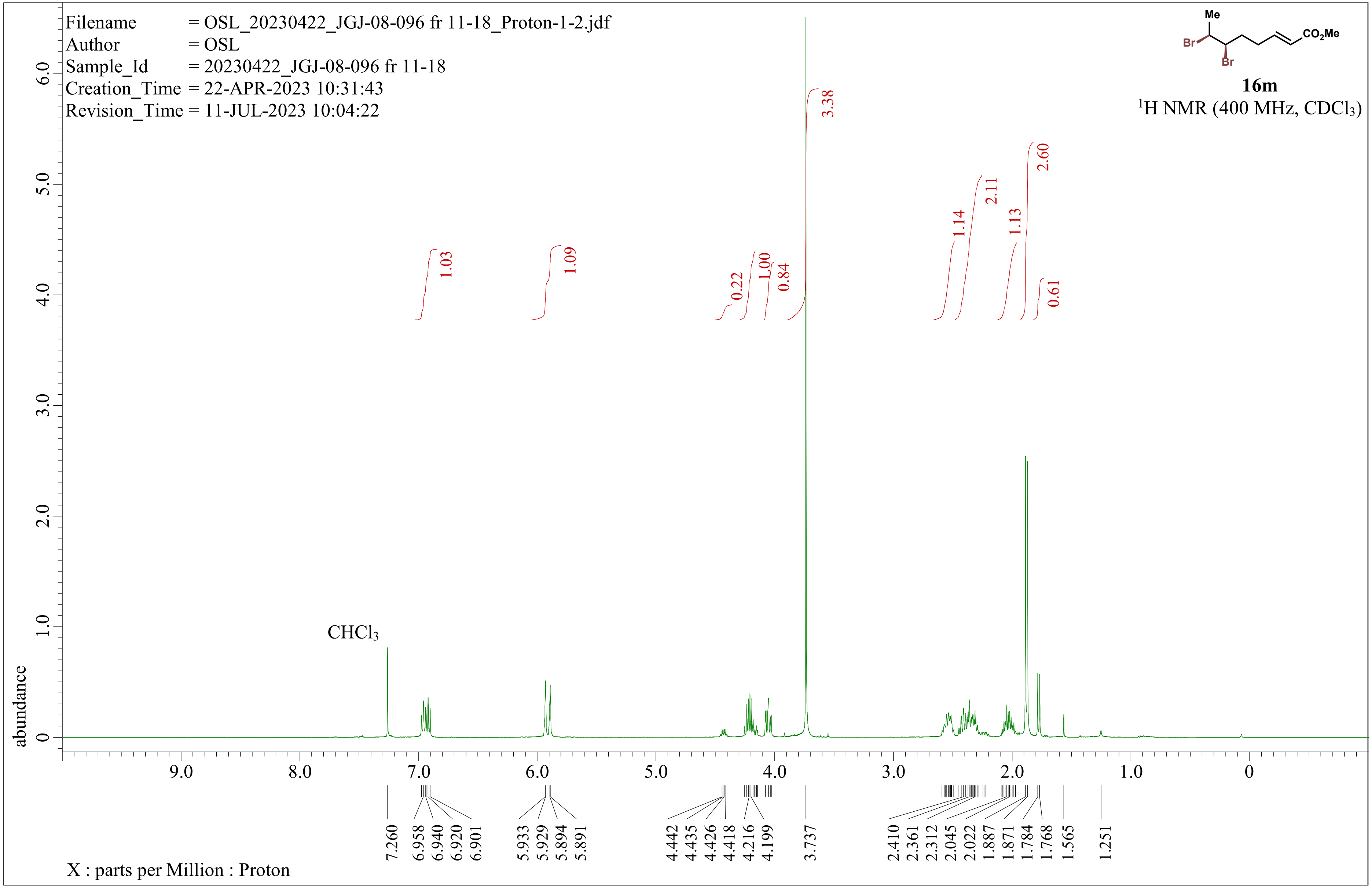

Supplementary Fig. 59. ^{1}H NMR spectrum of compound **16m**, recorded at 400 MHz and 298 K in $CDCl_3$.

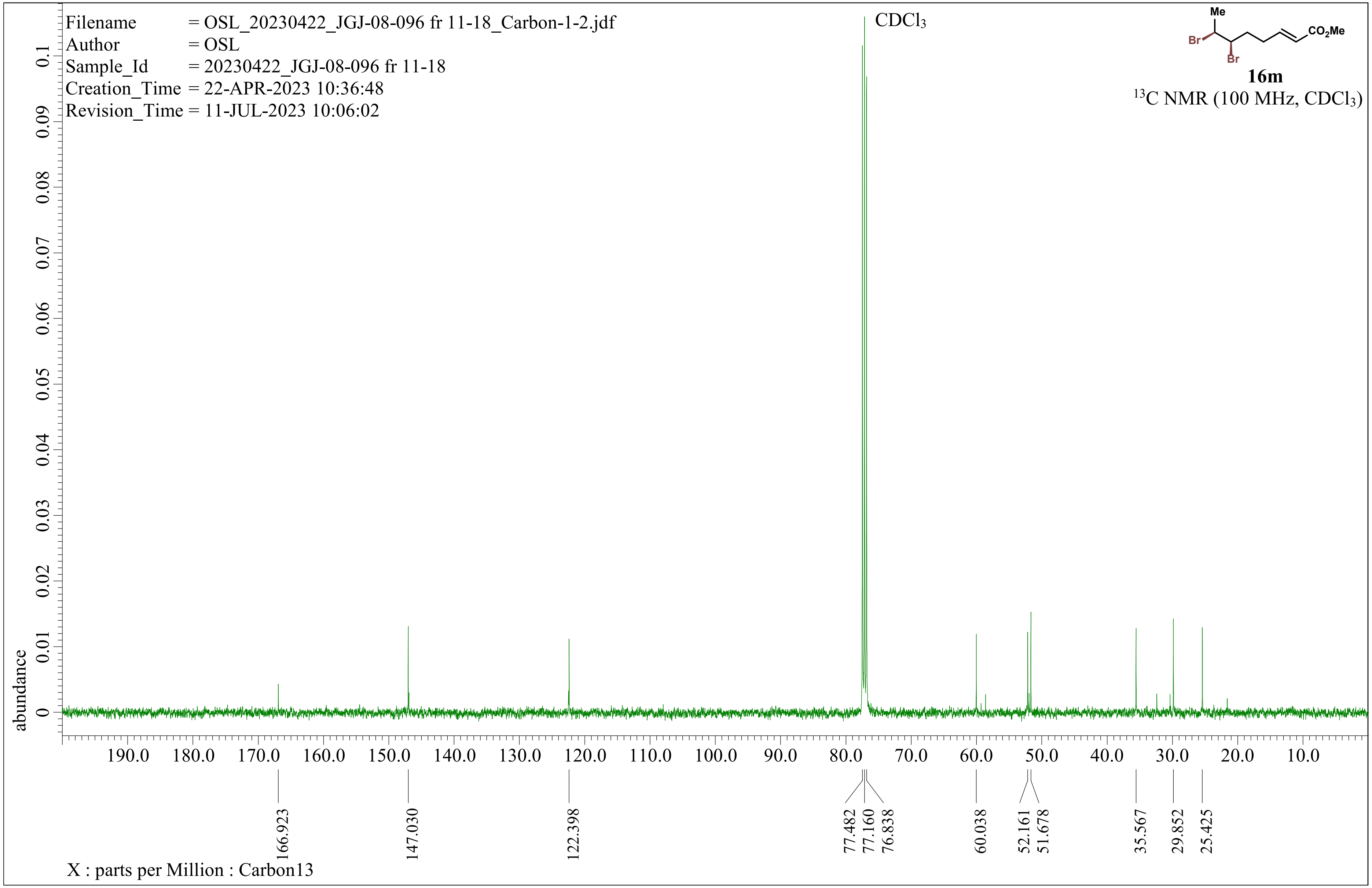

Supplementary Fig. 60. ^{13}C NMR spectrum of compound **16m**, recorded at 100 MHz and 298 K in $CDCl_3$.

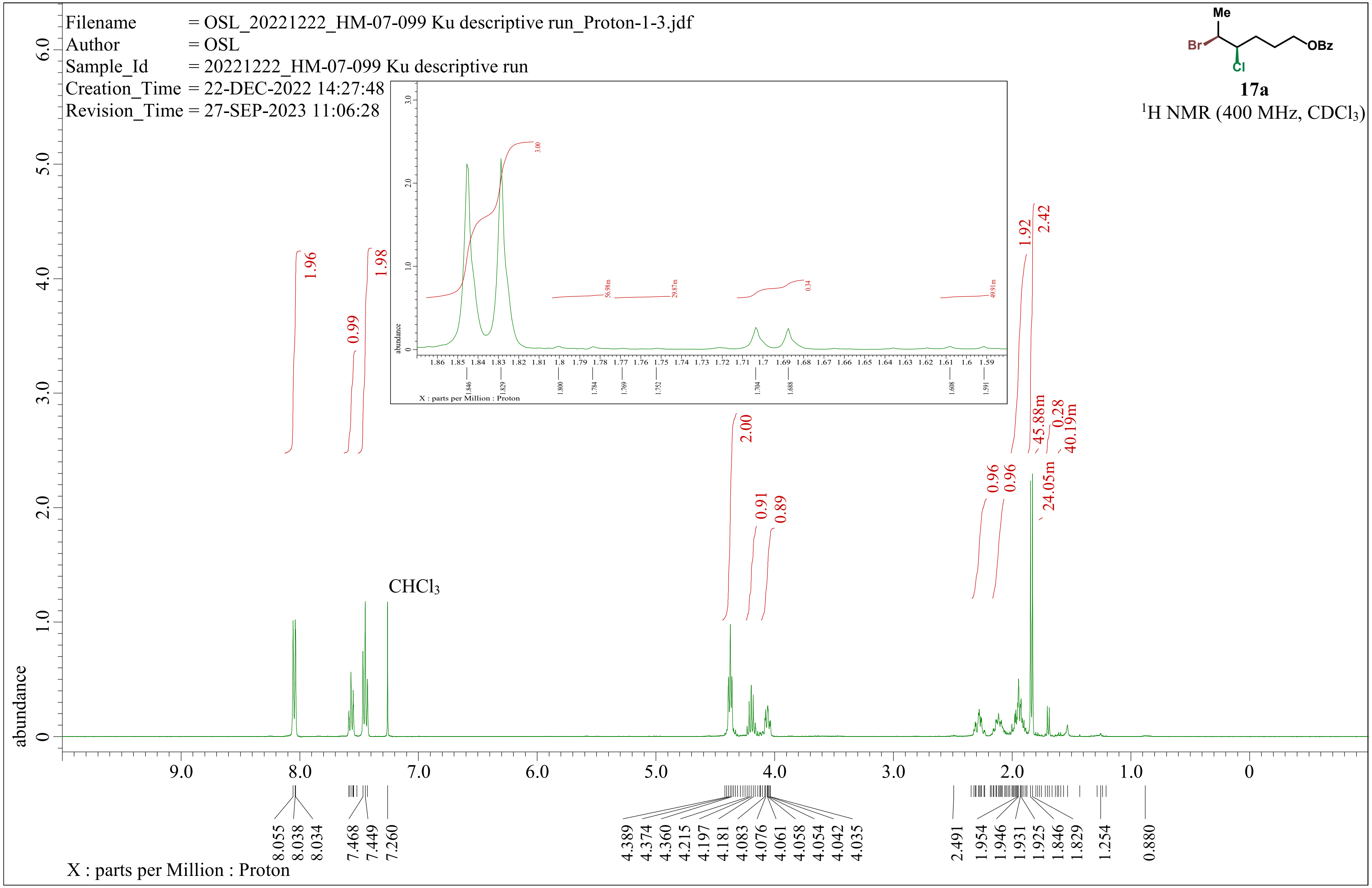

Supplementary Fig. 61. ^{1}H NMR spectrum of compound **17a**, recorded at 400 MHz and 298 K in $CDCl_3$.

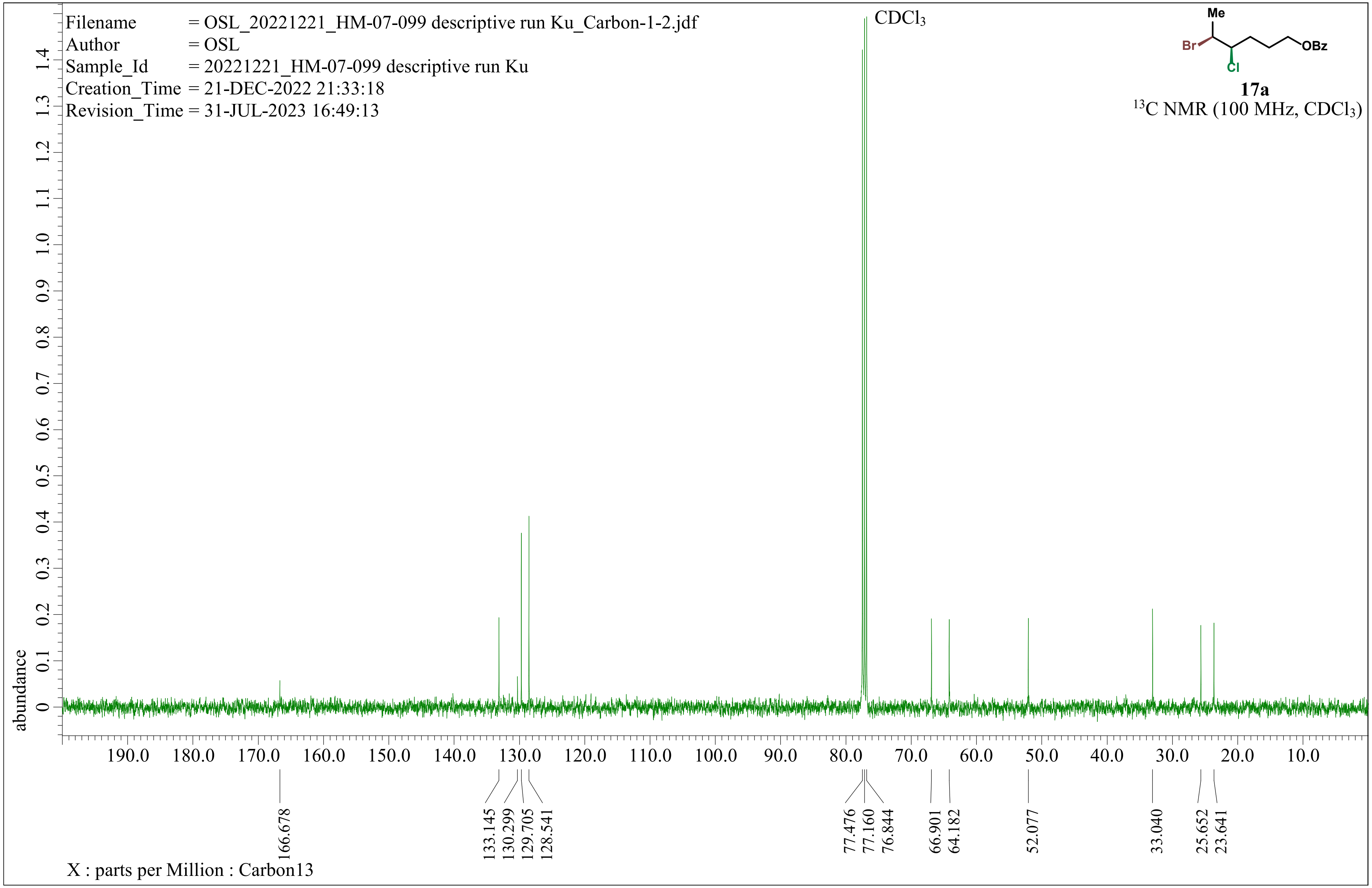

Supplementary Fig. 62. ^{13}C NMR spectrum of compound **17a**, recorded at 100 MHz and 298 K in $CDCl_3$.

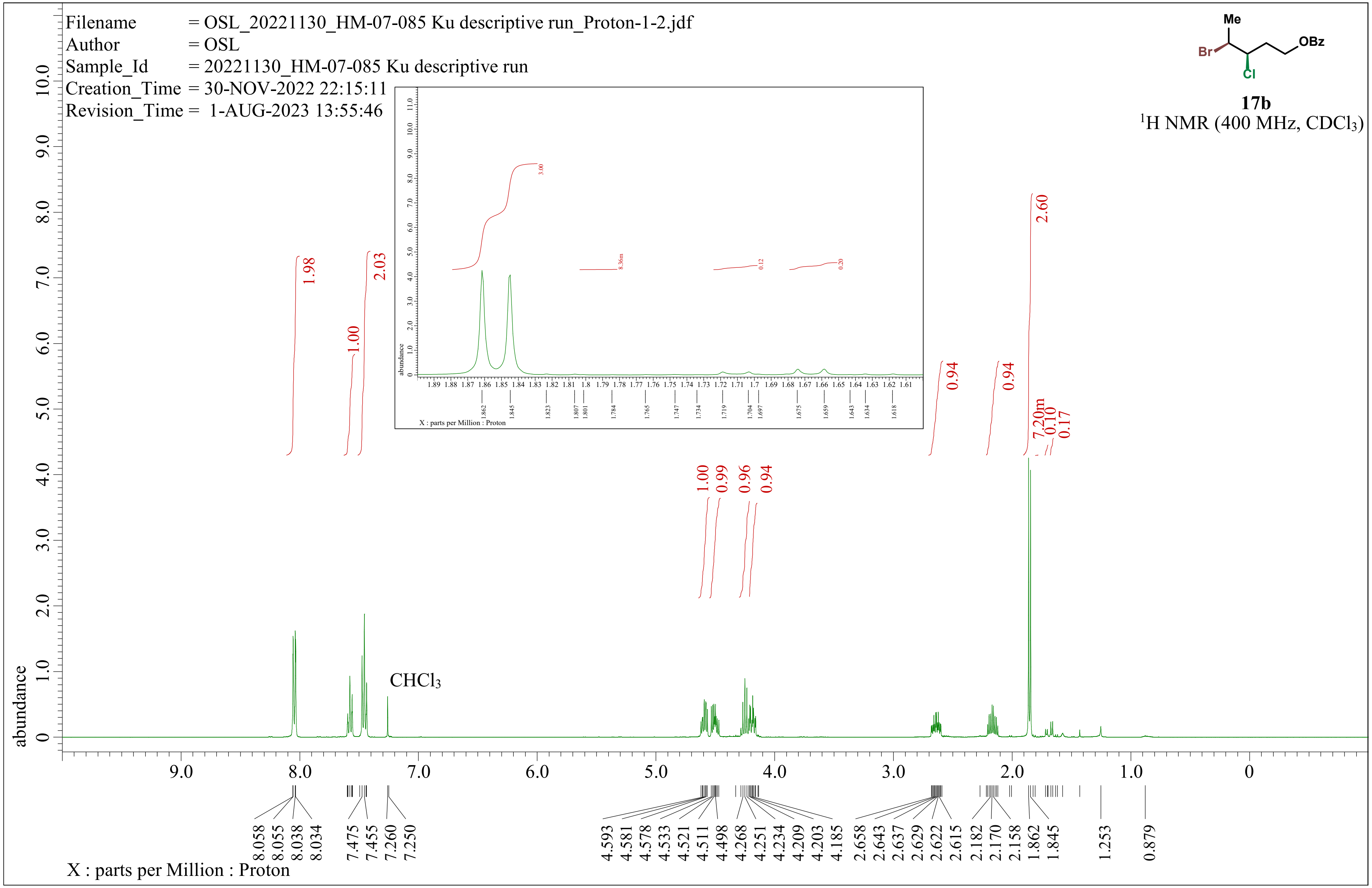

Supplementary Fig. 63. ^{1}H NMR spectrum of compound **17b**, recorded at 400 MHz and 298 K in $CDCl_3$.

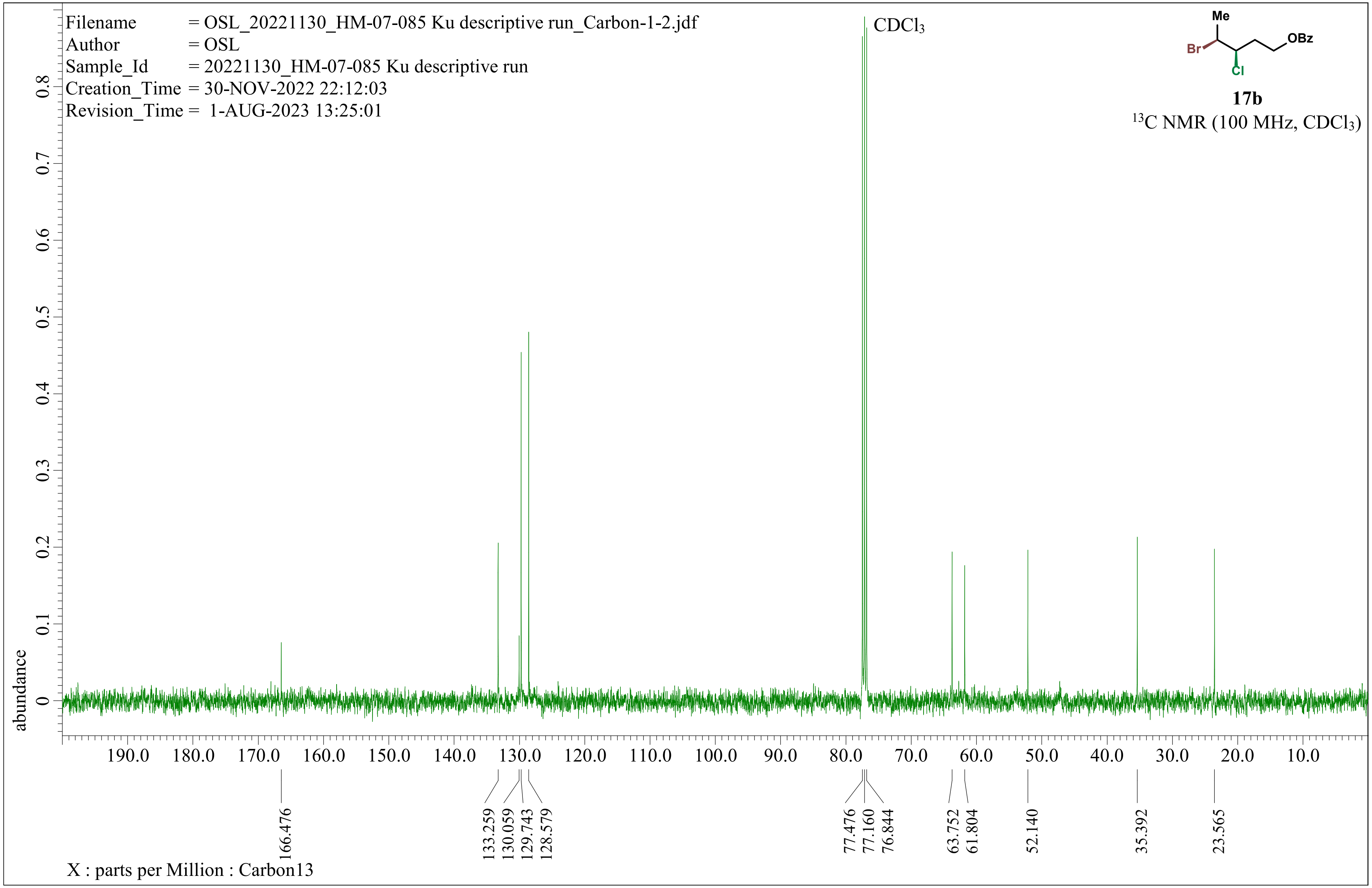

Supplementary Fig. 64. ^{13}C NMR spectrum of compound **17b**, recorded at 100 MHz and 298 K in $CDCl_3$.

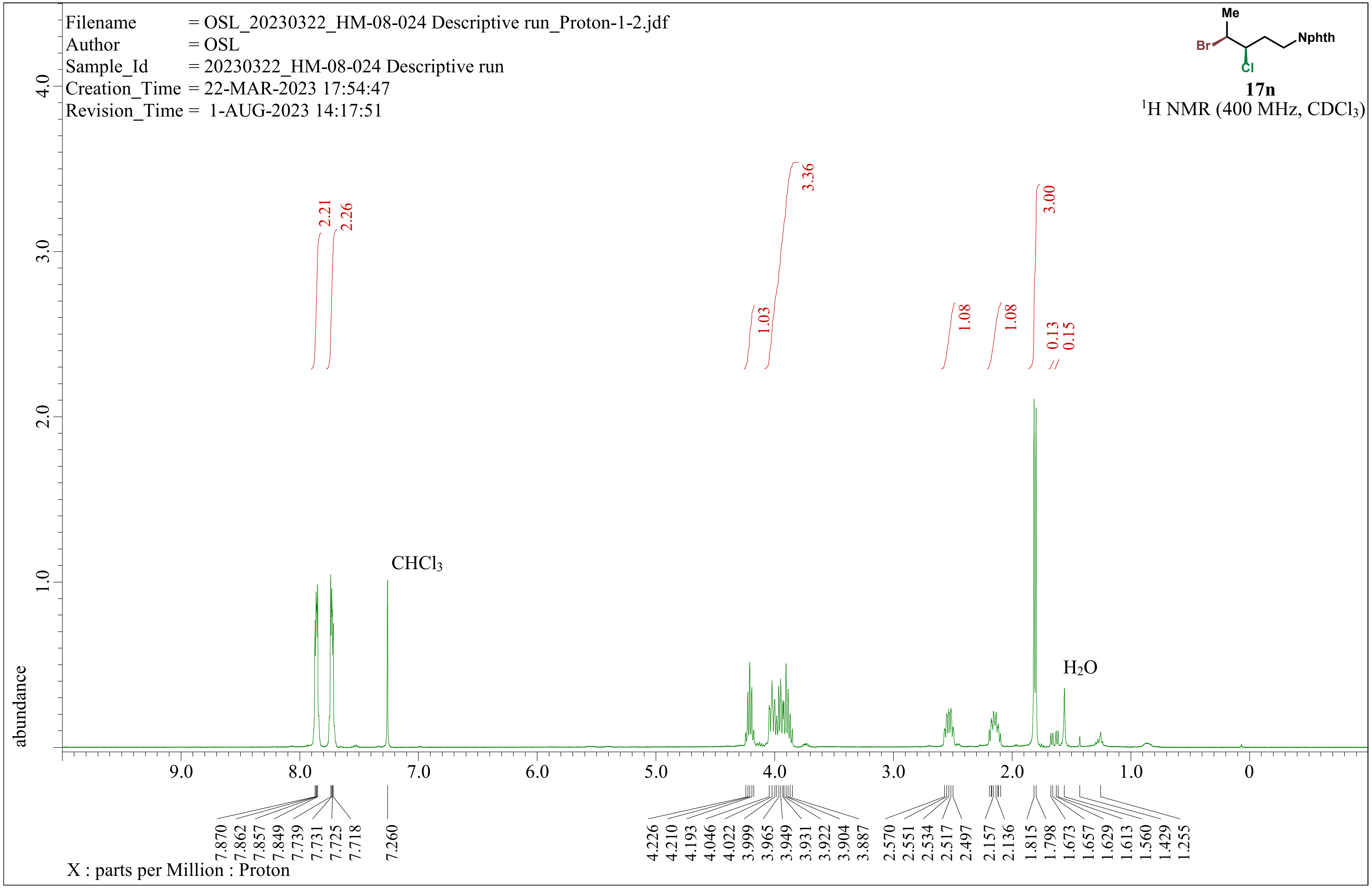

Supplementary Fig. 65. ^{1}H NMR spectrum of compound **17n**, recorded at 400 MHz and 298 K in $CDCl_3$.

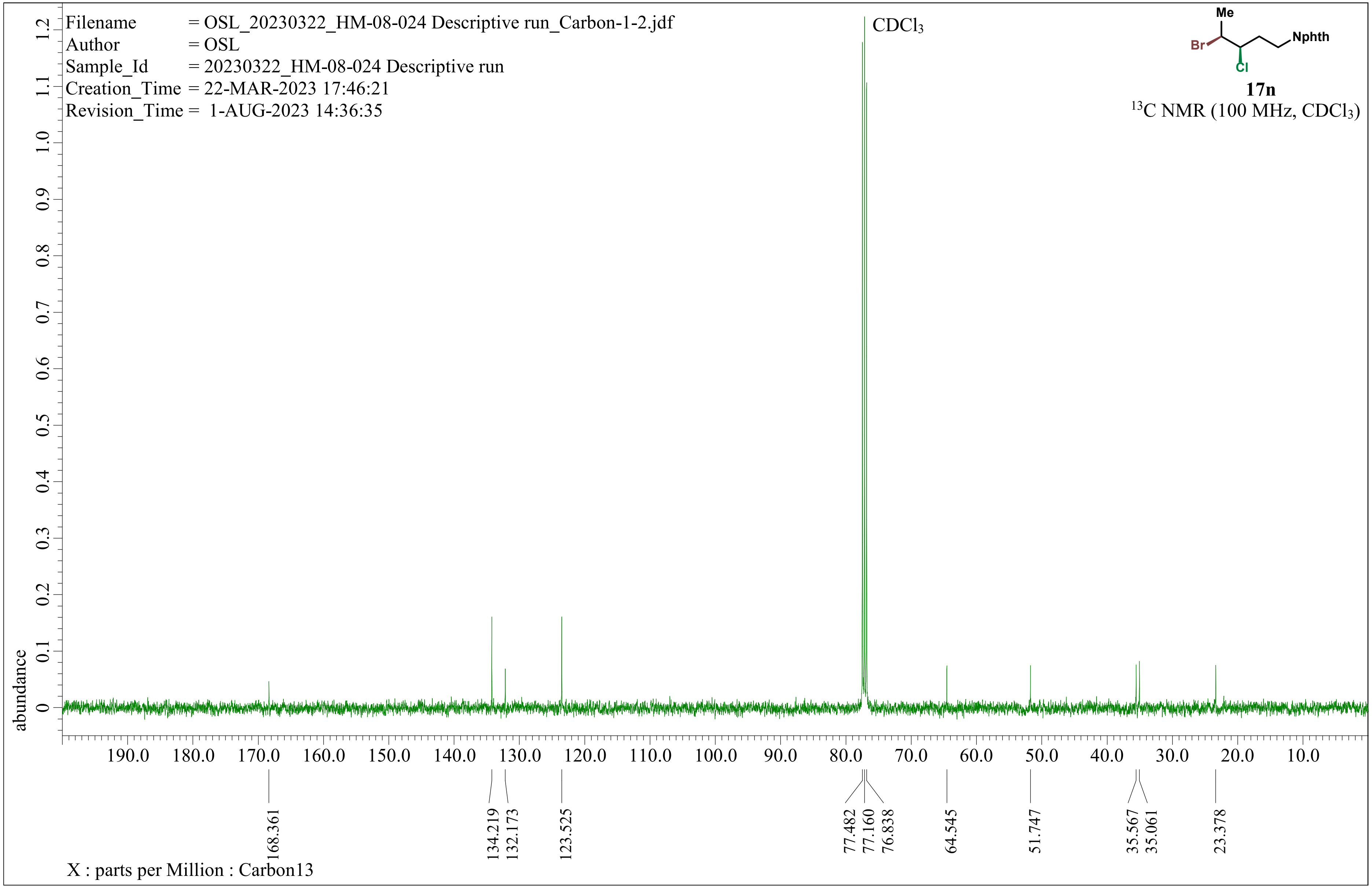

Supplementary Fig. 66. ^{13}C NMR spectrum of compound **17n**, recorded at 100 MHz and 298 K in $CDCl_3$.

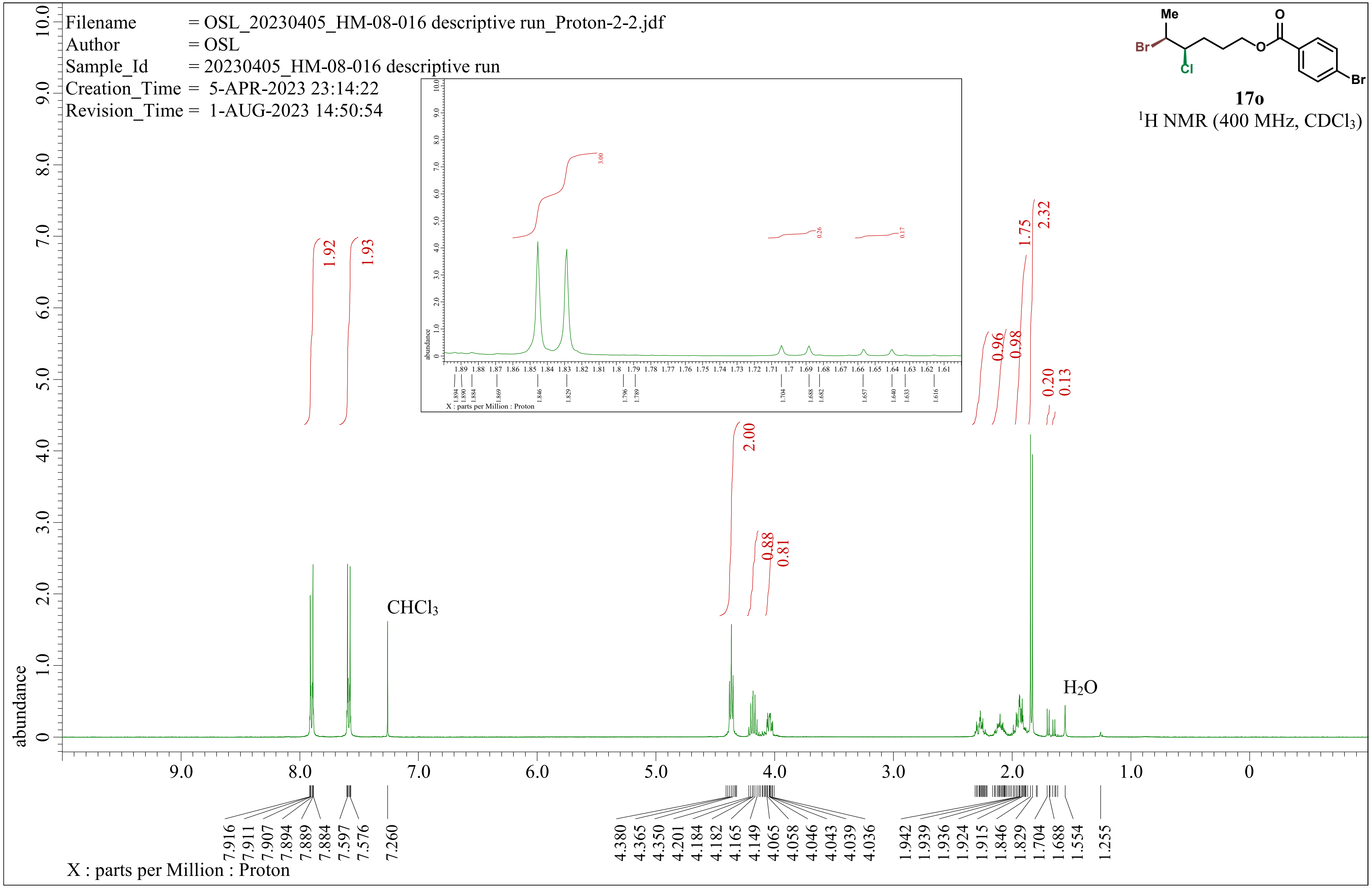

Supplementary Fig. 67. ^{1}H NMR spectrum of compound **17o**, recorded at 400 MHz and 298 K in $CDCl_3$.

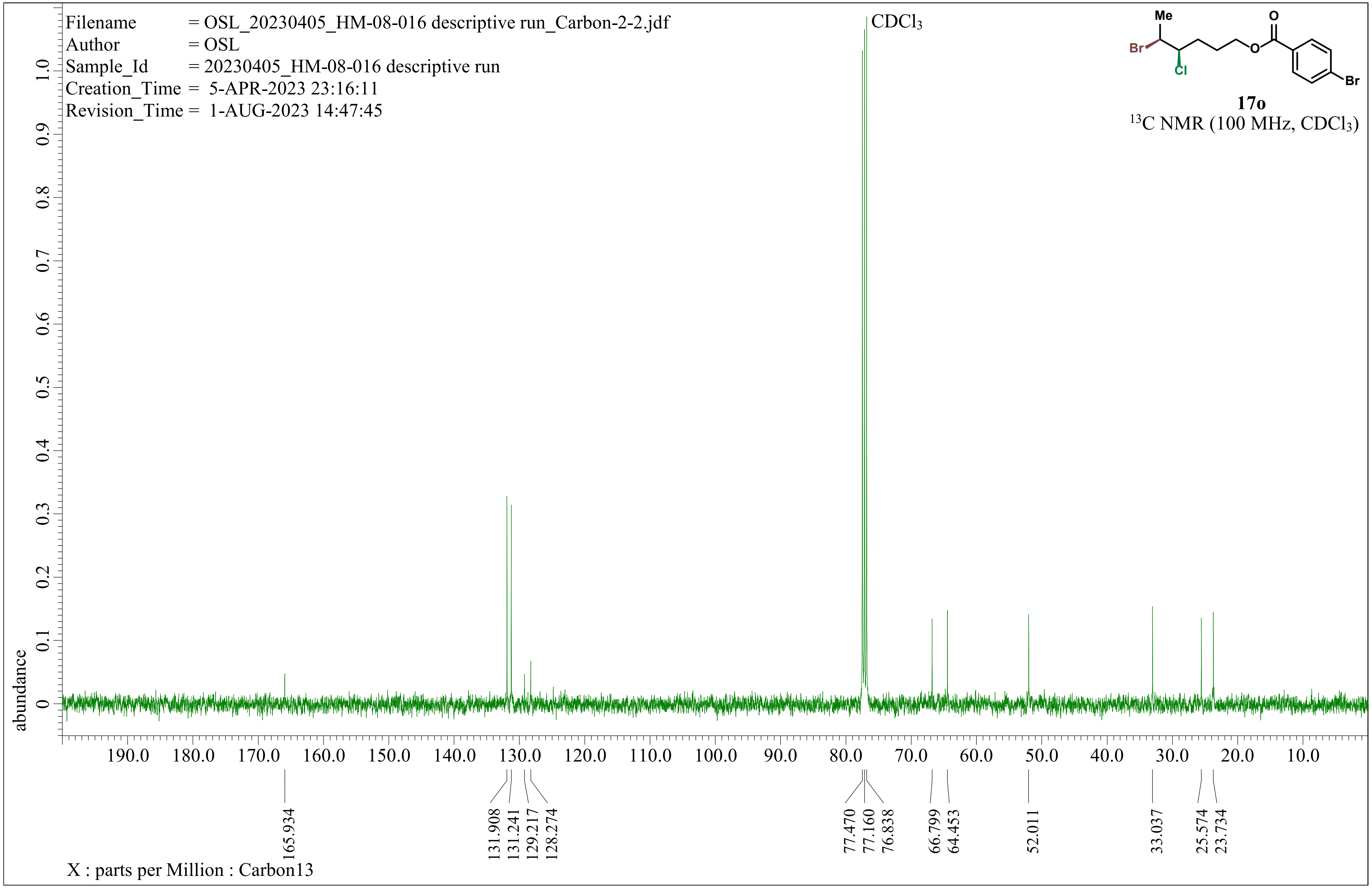

Supplementary Fig. 68. ^{13}C NMR spectrum of compound **17o**, recorded at 100 MHz and 298 K in $CDCl_3$.

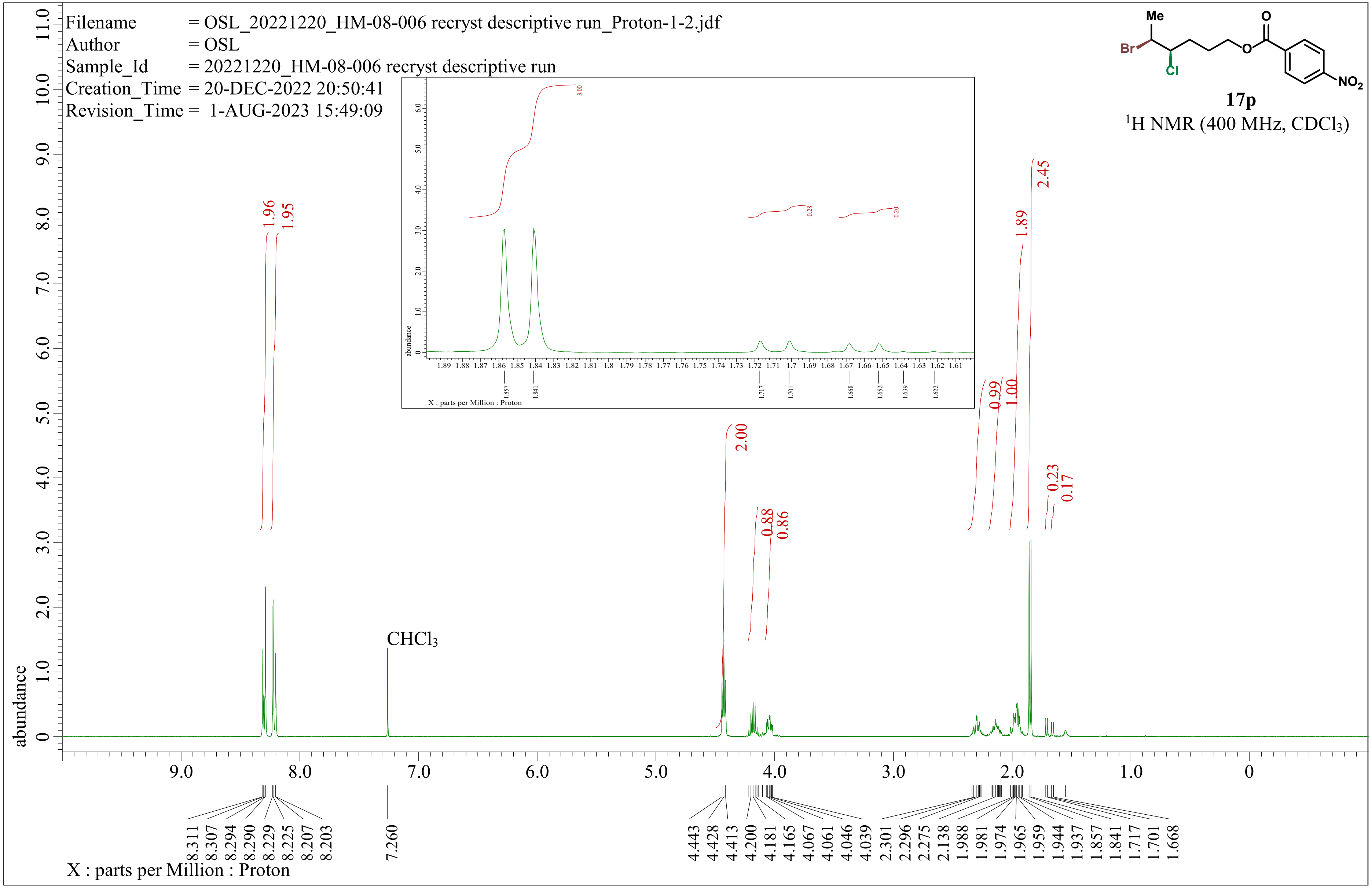

Supplementary Fig. 69. ^{1}H NMR spectrum of compound **17p**, recorded at 400 MHz and 298 K in $CDCl_3$.

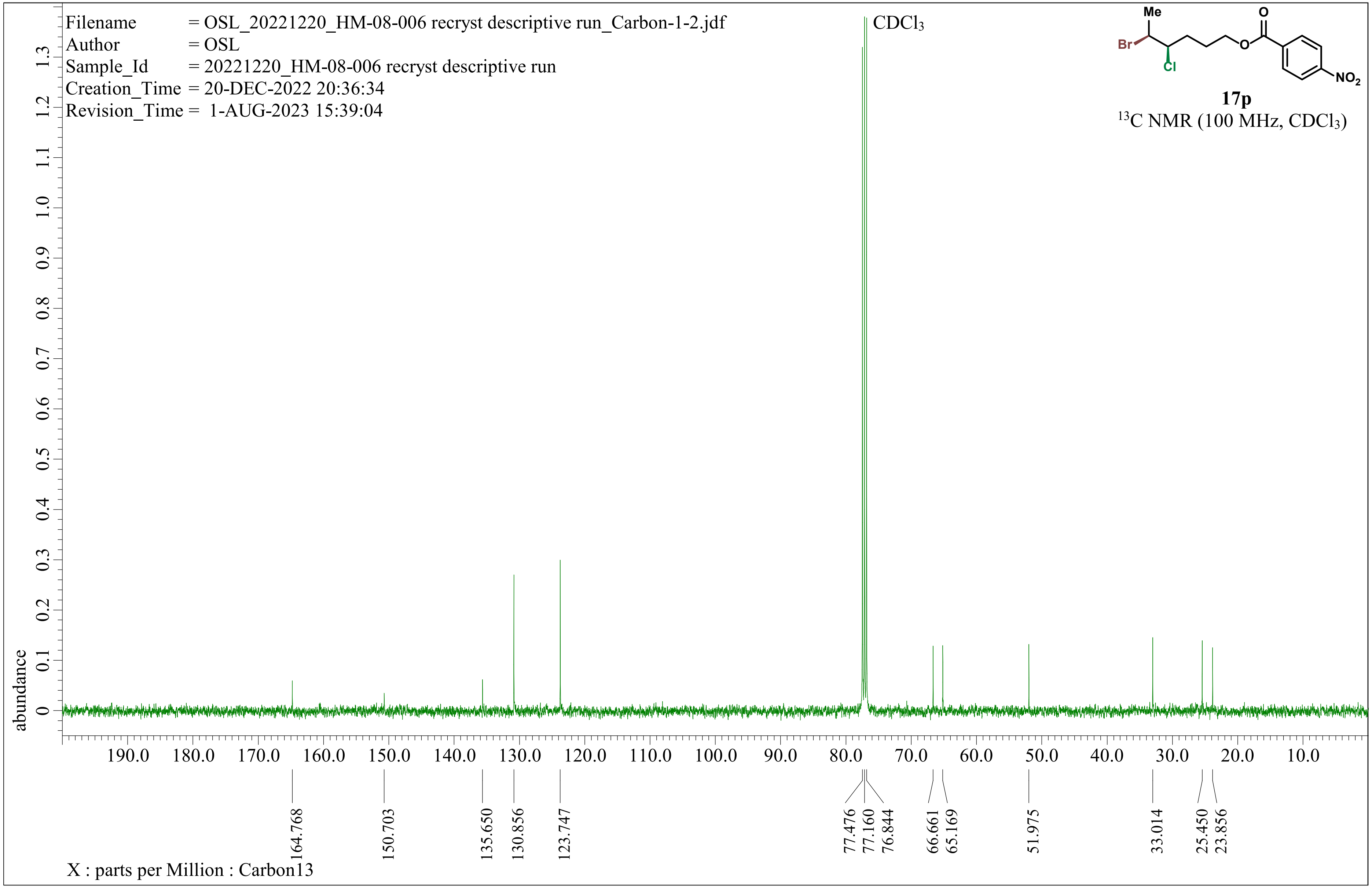

Supplementary Fig. 70. ^{13}C NMR spectrum of compound **17p**, recorded at 100 MHz and 298 K in $CDCl_3$.

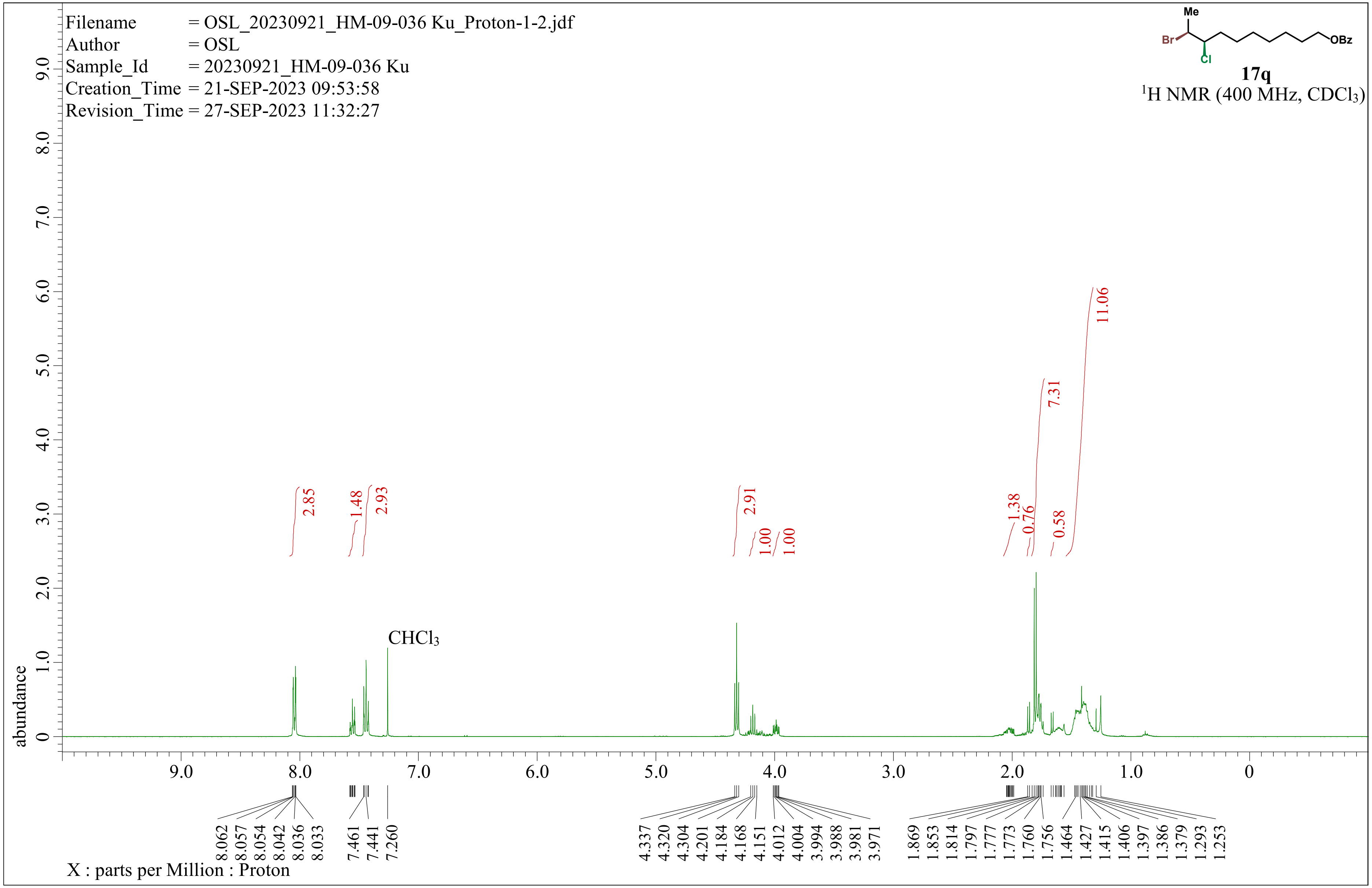

Supplementary Fig. 71. ^{1}H NMR spectrum of compound **17q**, recorded at 400 MHz and 298 K in $CDCl_3$.

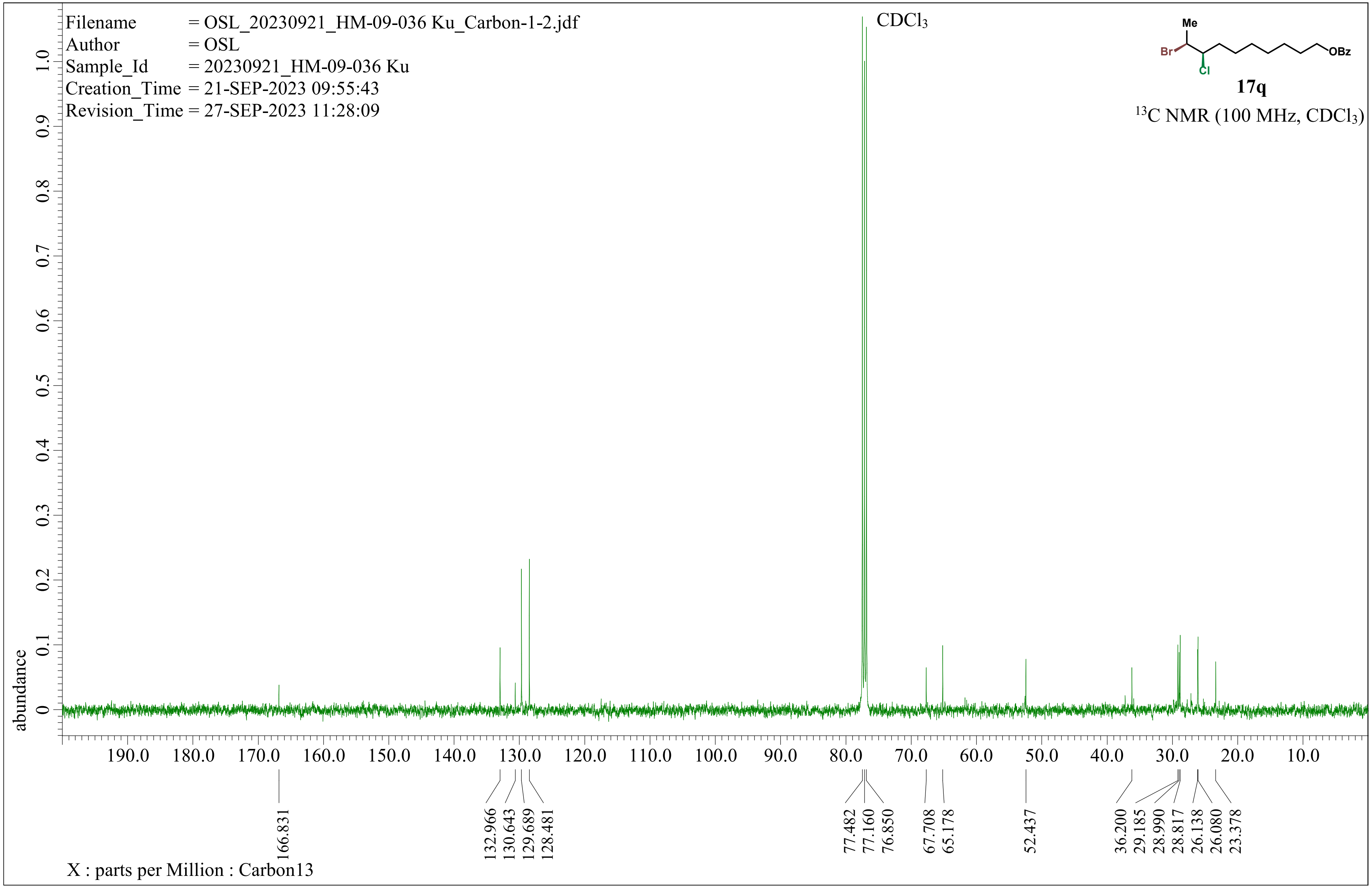

Supplementary Fig. 72. ^{13}C NMR spectrum of compound **17q**, recorded at 100 MHz and 298 K in $CDCl_3$.

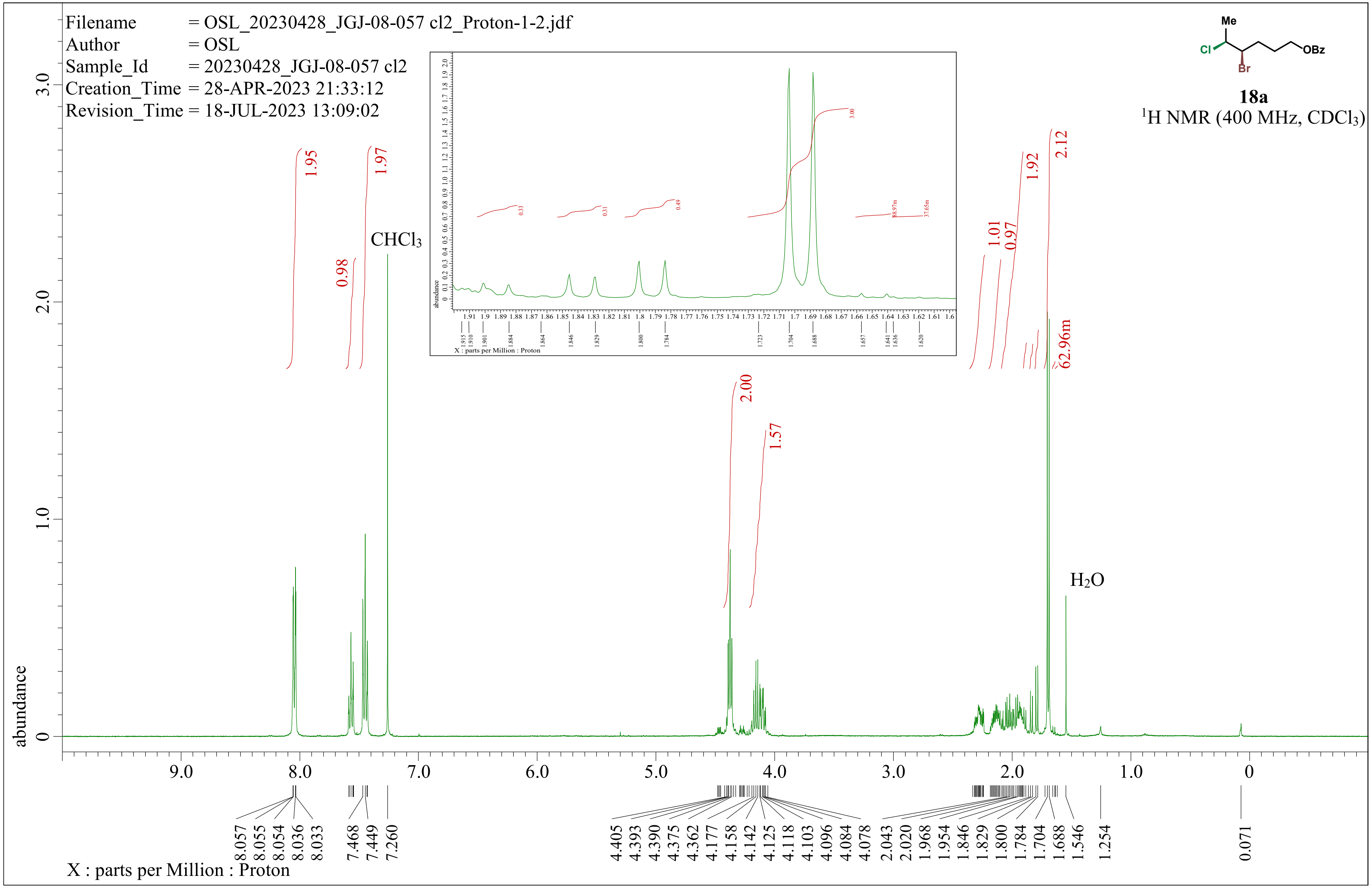

Supplementary Fig. 73. ^{1}H NMR spectrum of compound **18a**, recorded at 400 MHz and 298 K in $CDCl_3$.

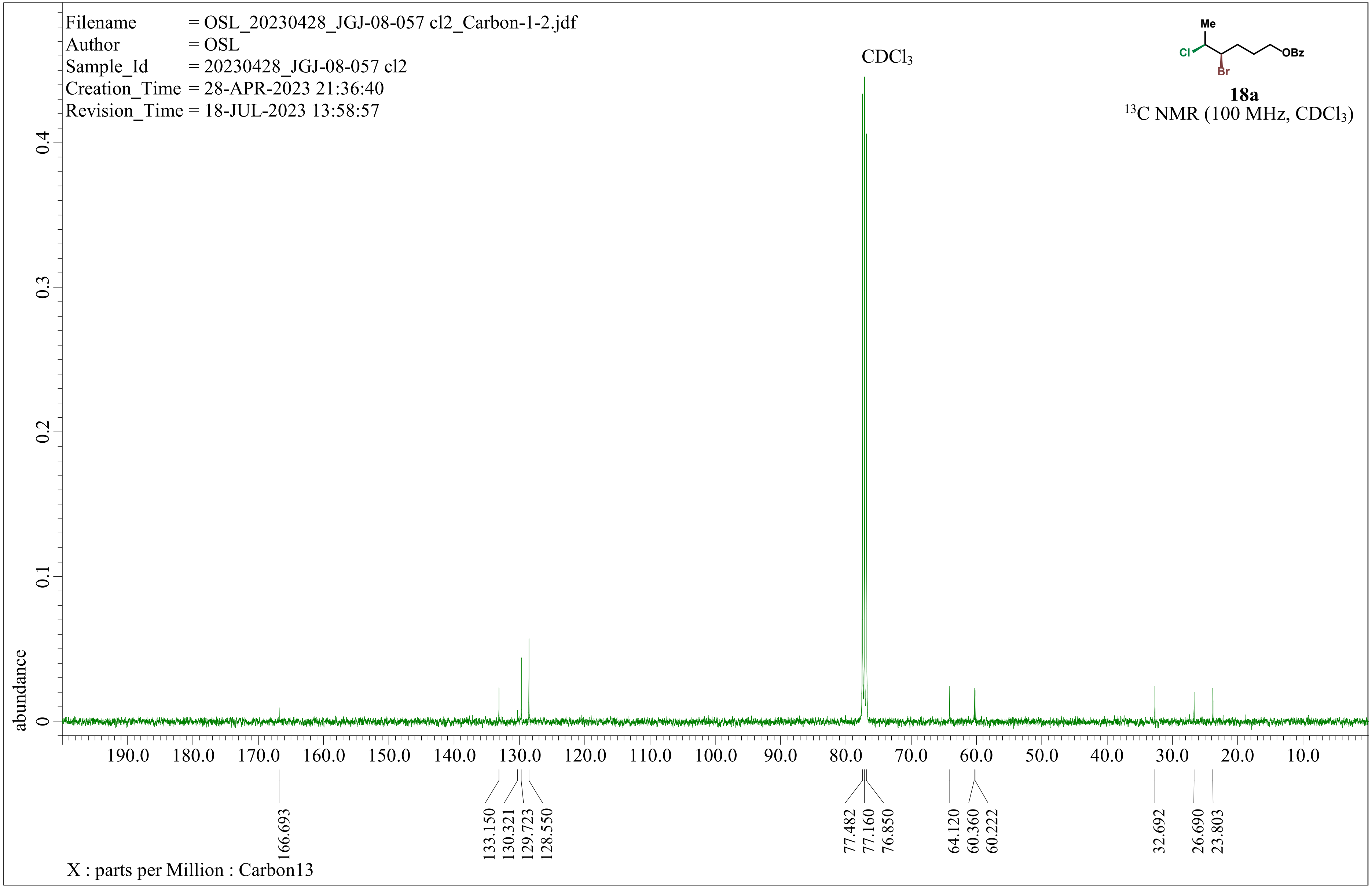

Supplementary Fig. 74. ^{13}C NMR spectrum of compound **18a**, recorded at 100 MHz and 298 K in $CDCl_3$.

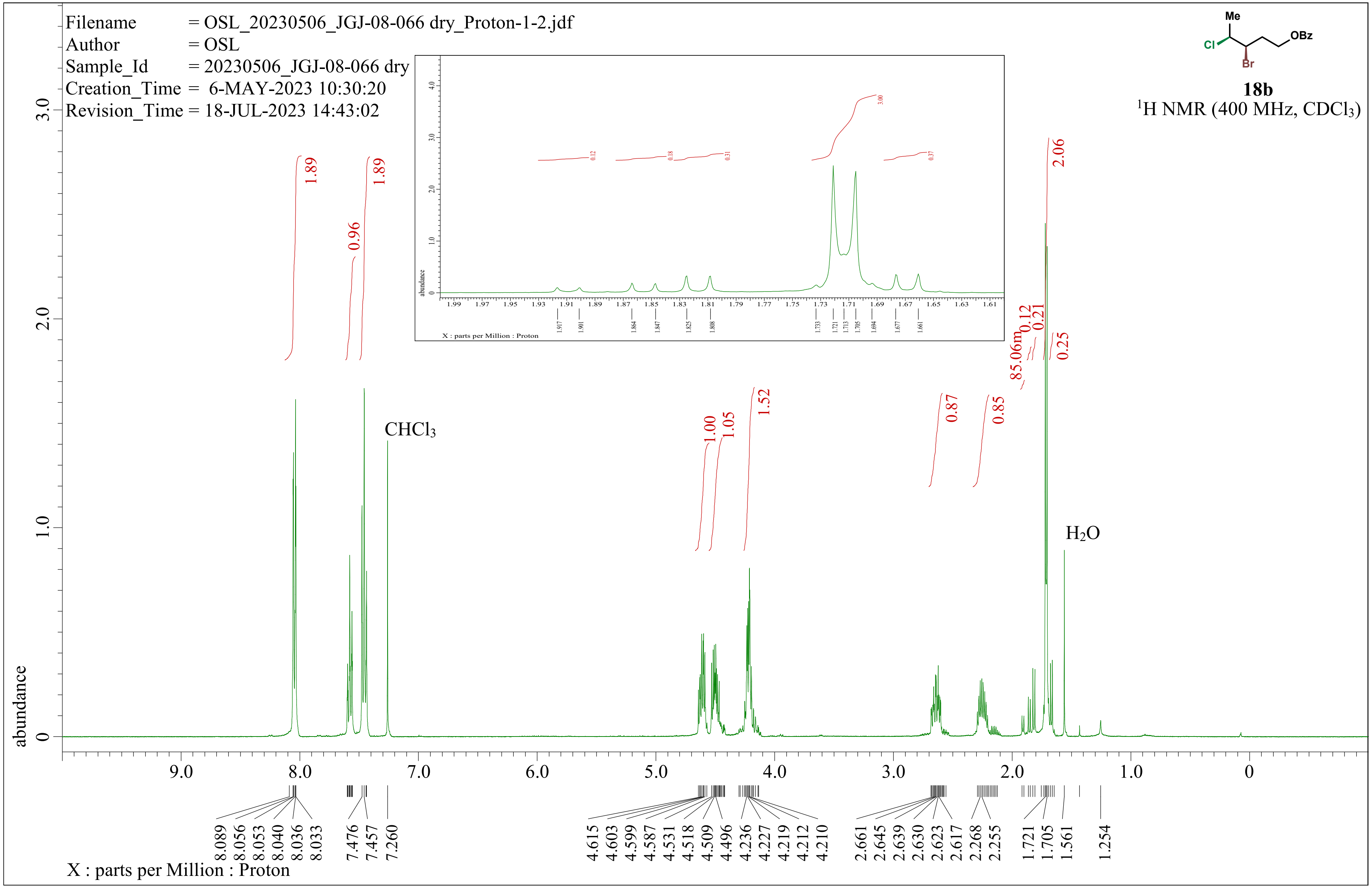

Supplementary Fig. 75. ^{1}H NMR spectrum of compound **18b**, recorded at 400 MHz and 298 K in $CDCl_3$.

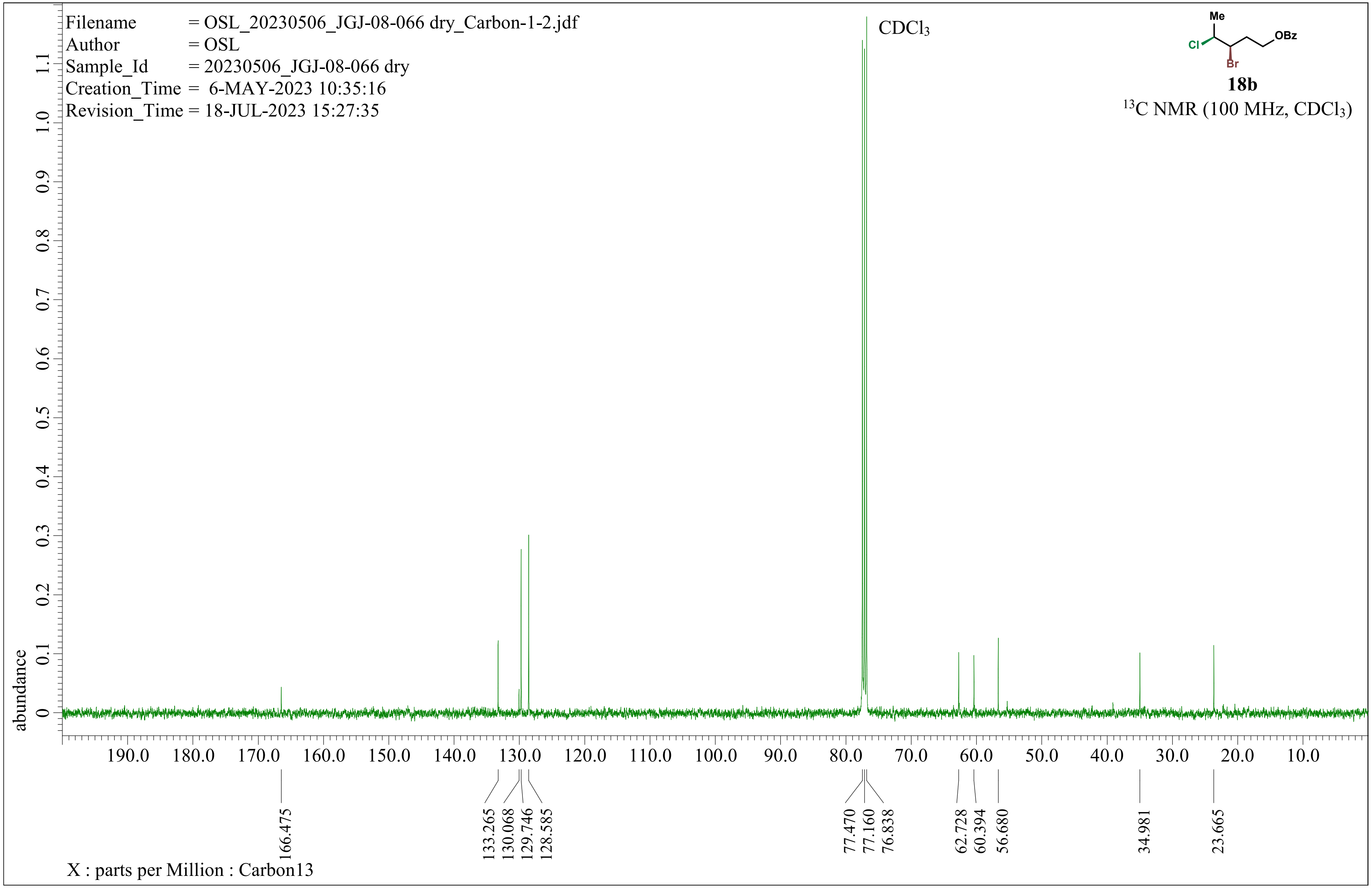

Supplementary Fig. 76. ^{13}C NMR spectrum of compound **18b**, recorded at 100 MHz and 298 K in $CDCl_3$.

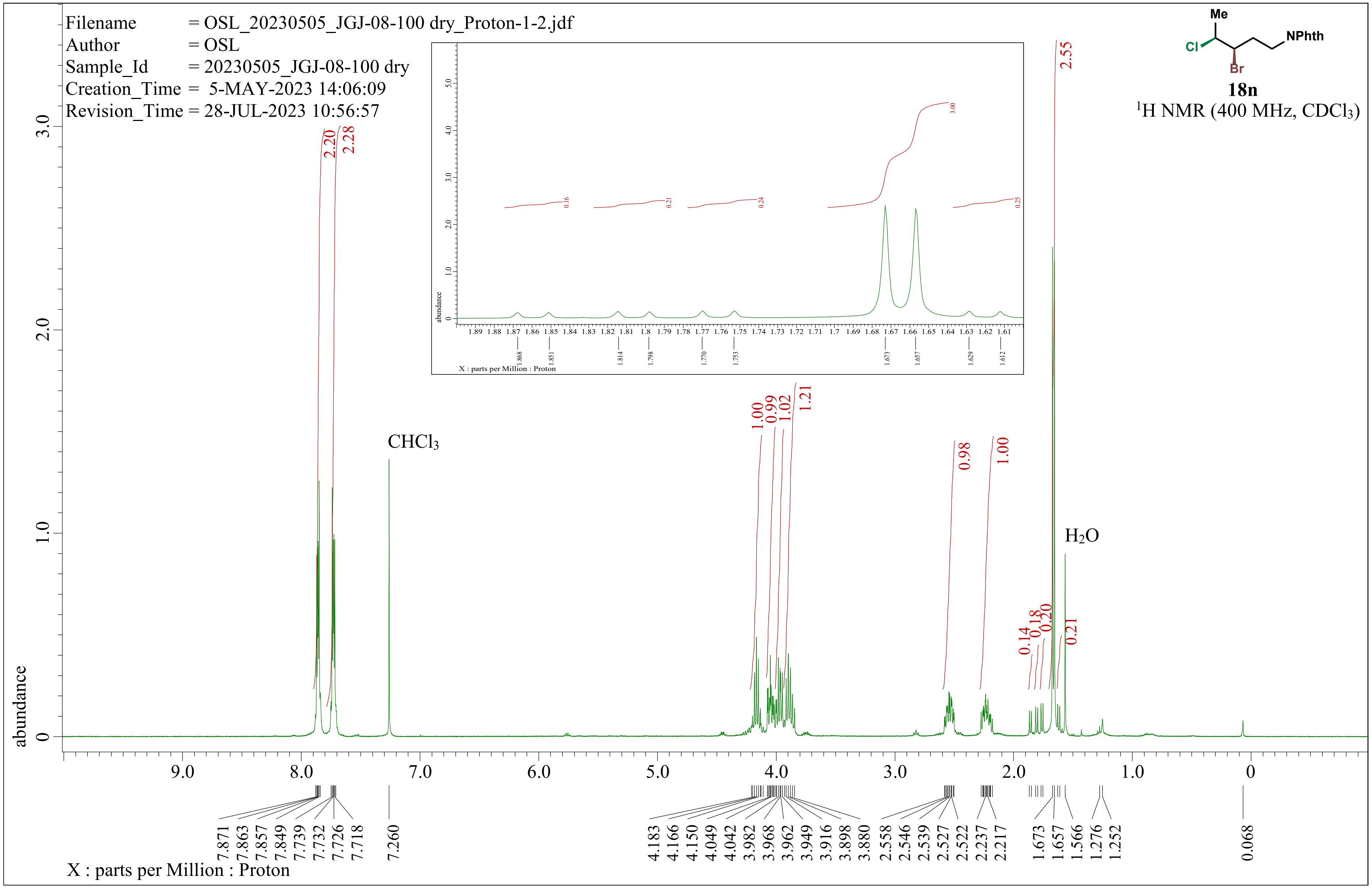

Supplementary Fig. 77. ^1H NMR spectrum of compound **18n**, recorded at 400 MHz and 298 K in $CDCl_3$.

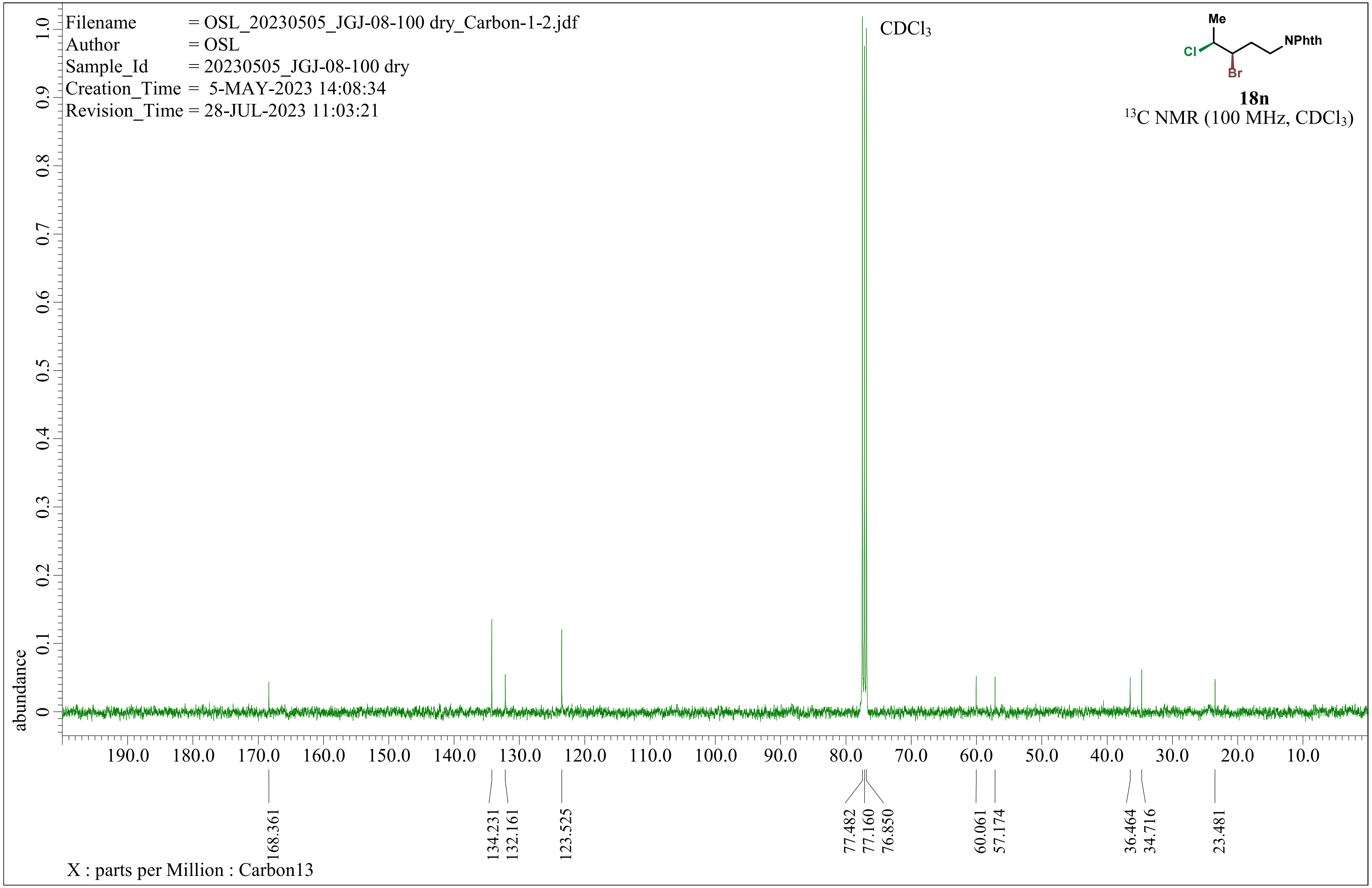

Supplementary Fig. 78. ^{13}C NMR spectrum of compound **18n**, recorded at 100 MHz and 298 K in $CDCl_3$.

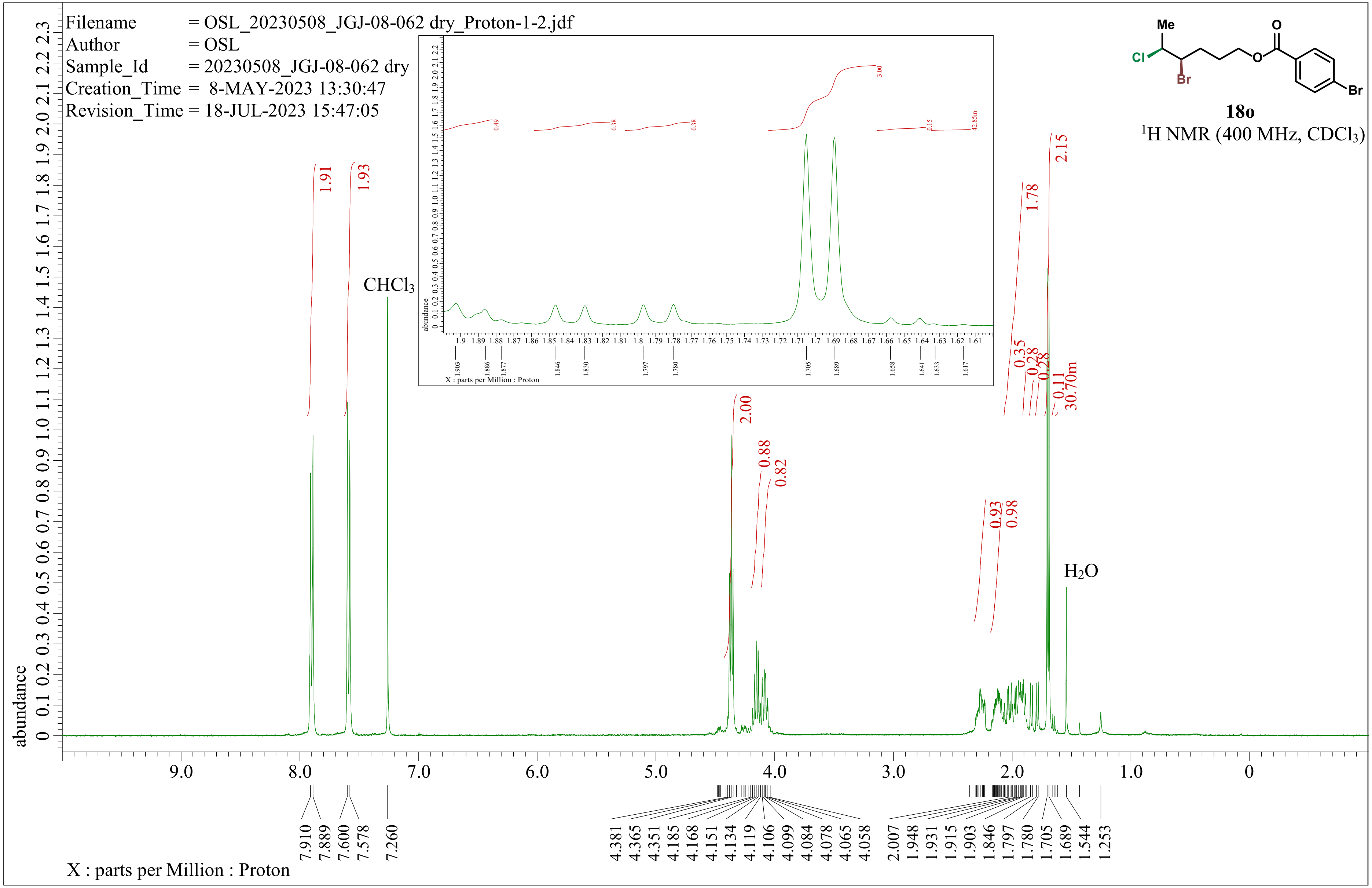

Supplementary Fig. 79. ^{1}H NMR spectrum of compound **18o**, recorded at 400 MHz and 298 K in $CDCl_3$.

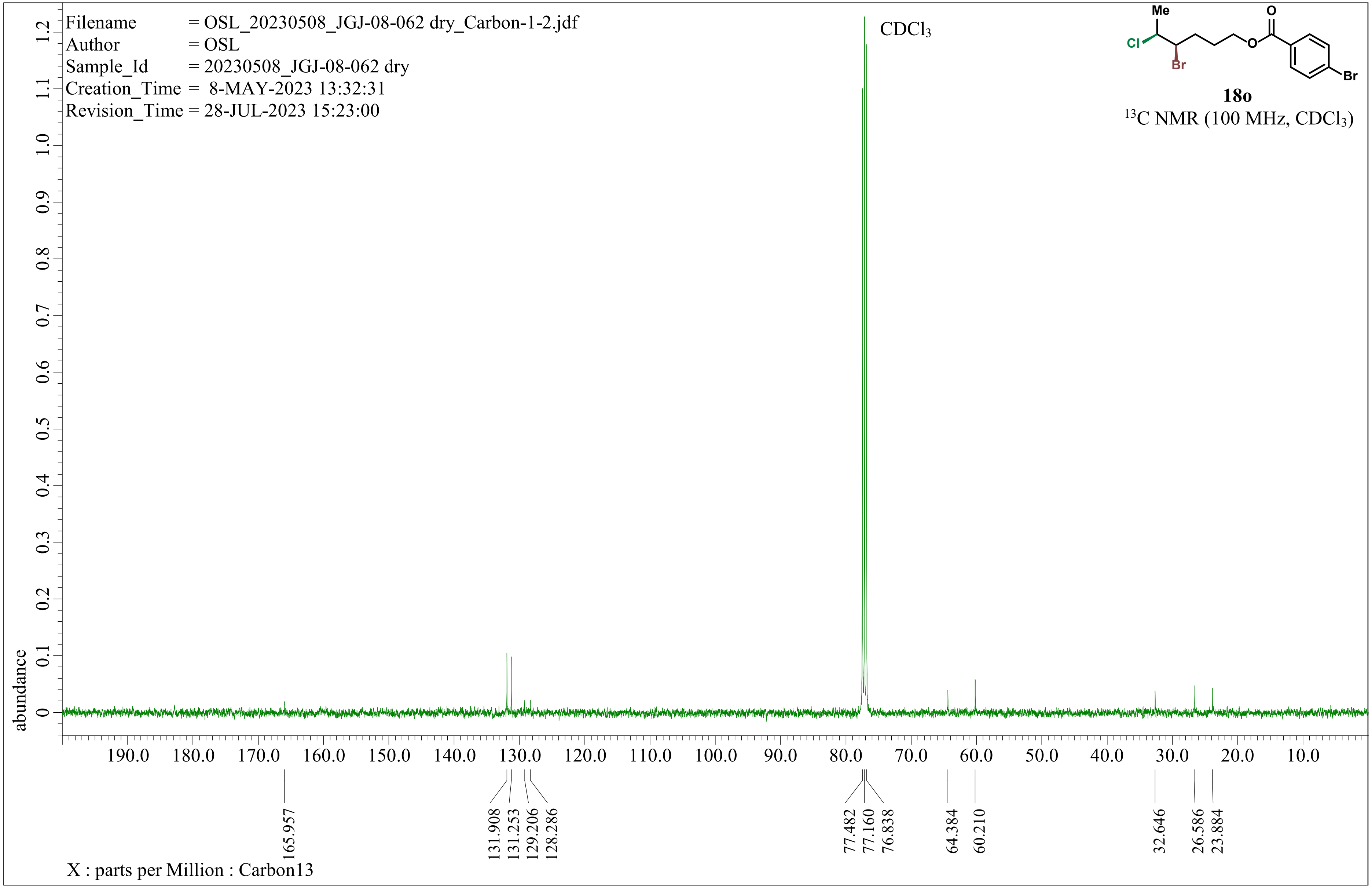

Supplementary Fig. 80. ^{13}C NMR spectrum of compound **18o**, recorded at 100 MHz and 298 K in $CDCl_3$.

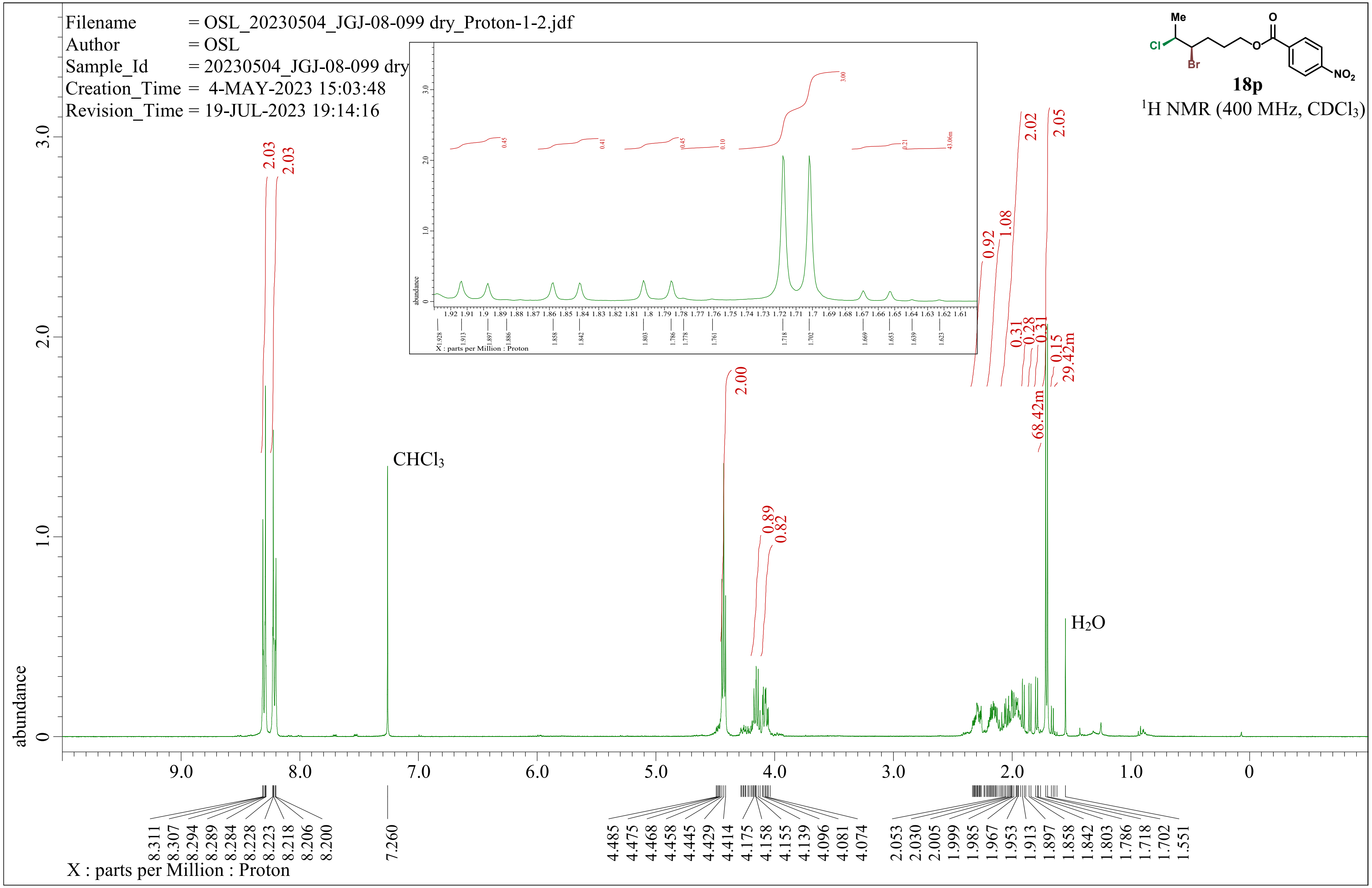

Supplementary Fig. 81. ^{1}H NMR spectrum of compound **18p**, recorded at 400 MHz and 298 K in $CDCl_3$.

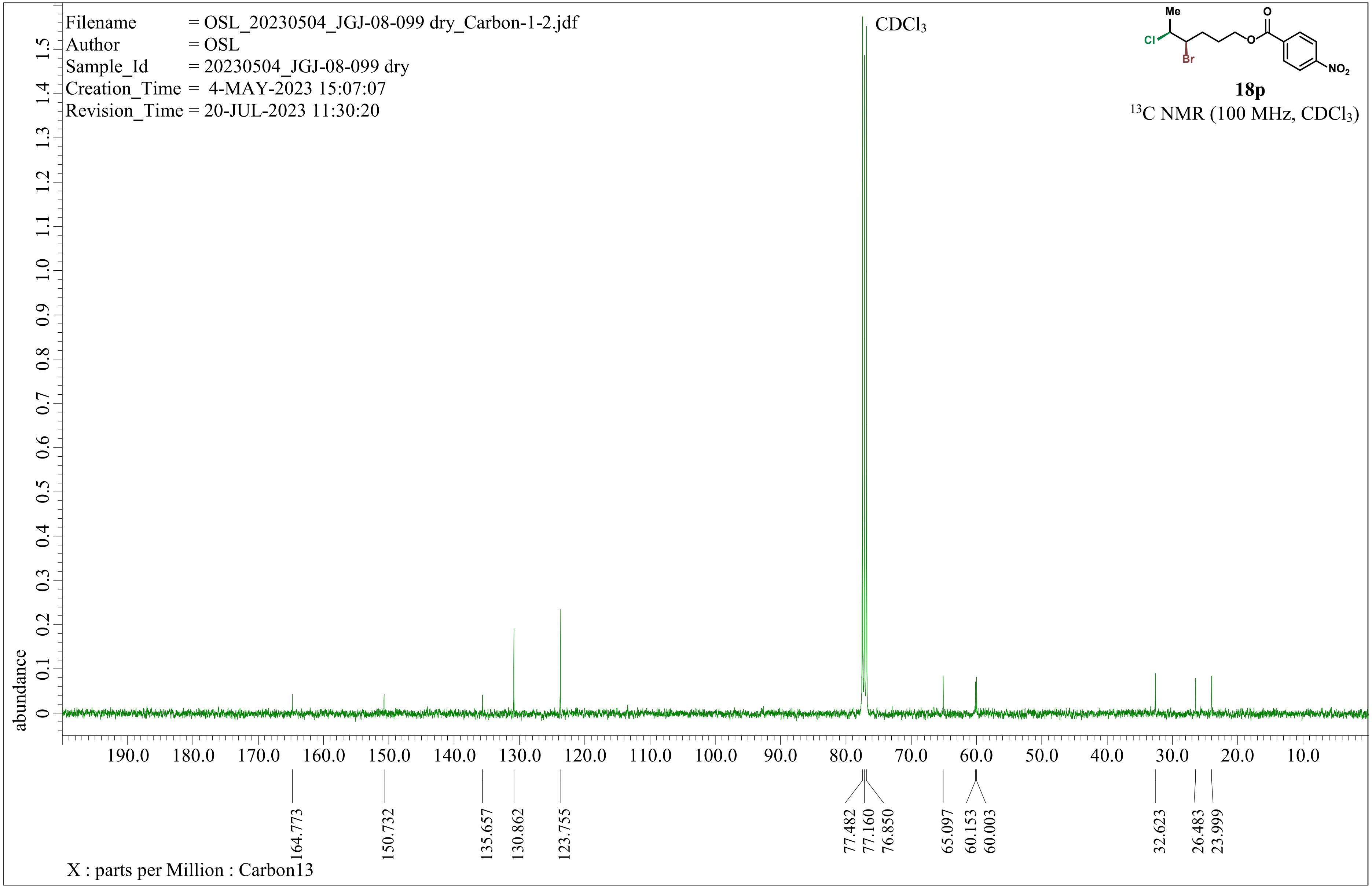

Supplementary Fig. 82. ^{13}C NMR spectrum of compound **18p**, recorded at 100 MHz and 298 K in $CDCl_3$.

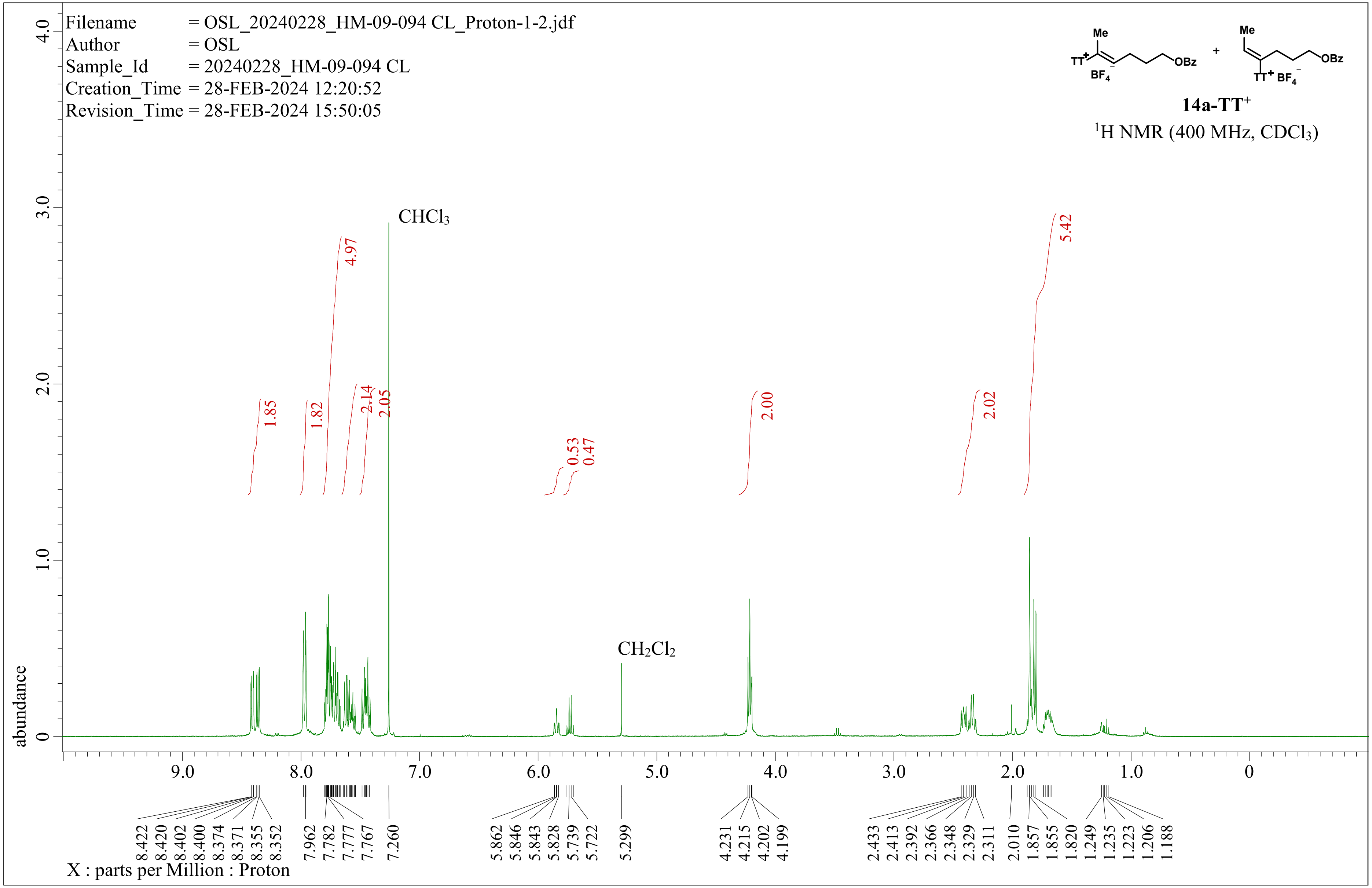

Supplementary Fig. 83.^{1}H NMR spectrum of compound **14a-TT^{+}**, recorded at 400 MHz and 298 K in $CDCl_3$.

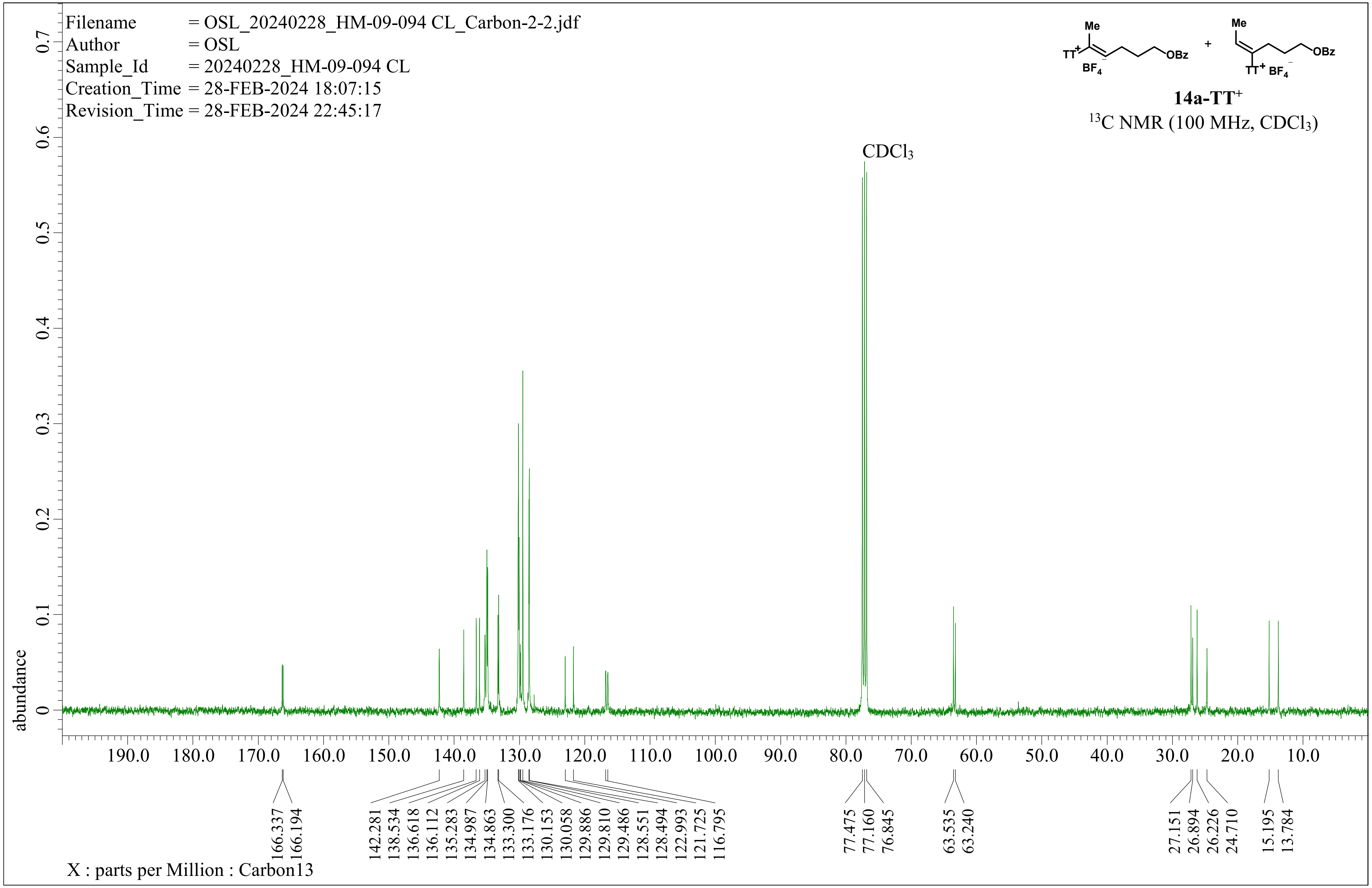

Supplementary Fig. 84. ^{13}C NMR spectrum of compound **14a-TT⁺**, recorded at 100 MHz and 298 K in $CDCl_3$.

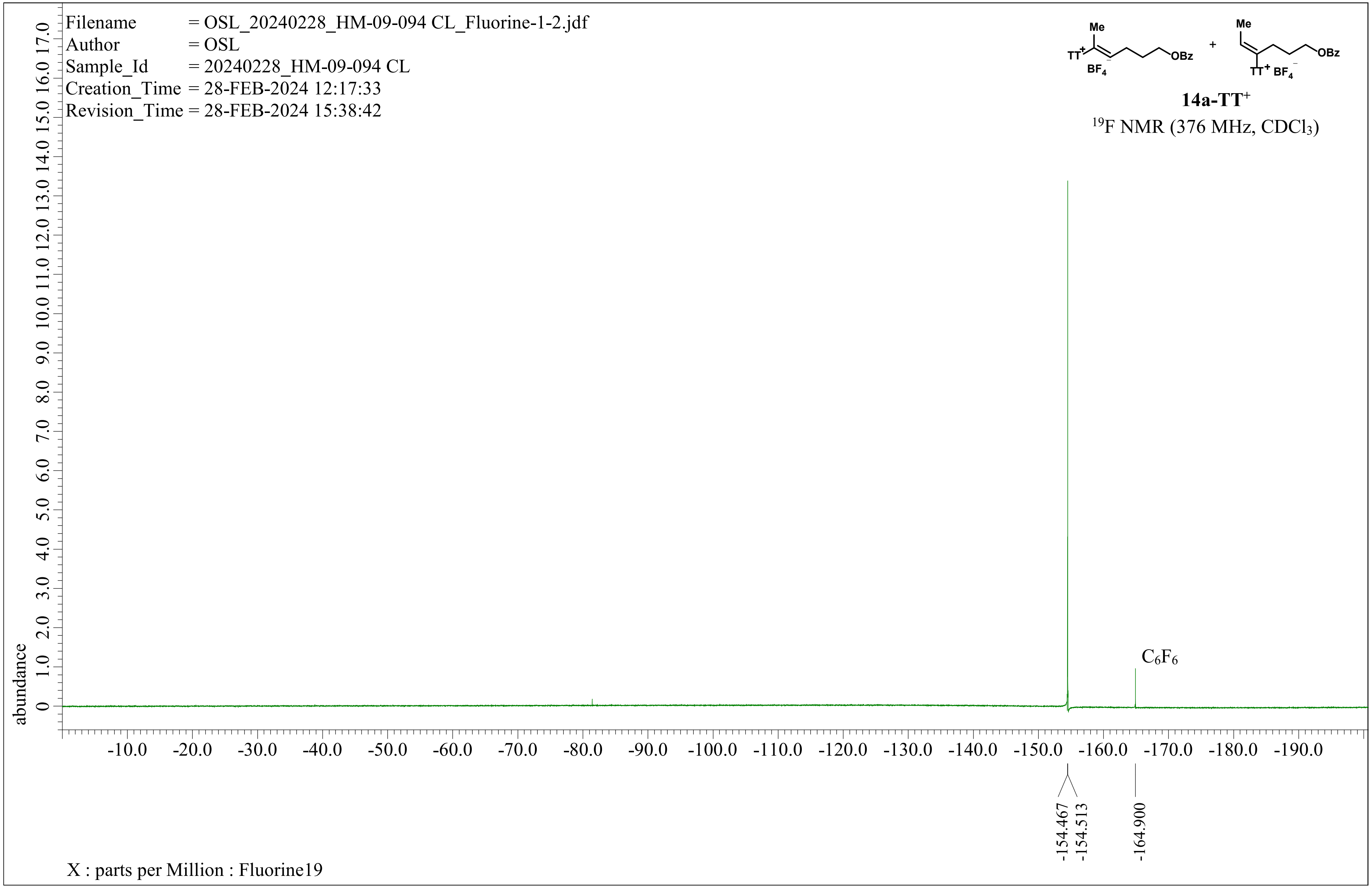

Supplementary Fig. 85. ^{19}F NMR spectrum of compound **14a-TT⁺**, recorded at 376 MHz and 298 K in $CDCl_3$.

4. Supplementary References

1. Cresswell, A. J., Eey, S. T.-C. & Denmark, S. E. Catalytic, stereospecific *syn*-dichlorination of alkenes. *Nat. Chem.* **7**, 146–152 (2015).
2. Ahmed, T. S., Montgomery, T. P. & Grubbs R. H. Using stereoretention for the synthesis of *E*-macrocycles with ruthenium-based olefin metathesis catalysts. *Chem. Sci.* **9**, 3580–3583 (2018).
3. Sarie, J. C., Neufeld, J., Daniliuc, C. G. & Gilmour, R. Catalytic vicinal dichlorination of unactivated alkenes. *ACS Catal.* **9**, 7232–7237 (2019).
4. Kunio, M., Toshiki, M. & Yoshin, T. *Cis*-configurational unsaturated ester, process for producing the same, and fragrance composition containing the same. EP 0 950 652 A1 (1999).
5. Ambroise, Y., Mioskowski, C., Djèga-Mariadassou, G. & Rousseau, B. Consequences of affinity in heterogeneous catalytic reactions: highly chemoselective hydrogenolysis of iodoarenes. *J. Org. Chem*. **65**, 7183–7186 (2000).
6. Cruz, D. A., Sinka, V., de Armas, P., Steingruber, H. S., Fernández, I., Martín, V. S., Miranda, P. O. & Padrón, J. I. Iron(II) and copper(I) control the total regioselectivity in the hydrobromination of alkenes. *Org. Lett.* **23**, 6105–6109 (2021).
7. Kilman, L. T., Mlynarski, S. N., Ferris, G. E. & Morken, J. P. Catalytic enantioselective 1,2-diboration of 1,3-dienes: versatile reagents for stereoselective allylation, *Angew. Chem., Int. Ed.* **51**, 521–524 (2012).
8. Nakagiri, T., Murai, M. & Takai, K. Stereospecific deoxygenation of aliphatic epoxides to alkenes under rhenium catalysis. *Org. Lett.* **17**, 3346–3349 (2015).
9. Bertrand, M. B., Neukom, J. D. & Wolfe, J. P. Mild conditions for Pd-catalyzed carboamination of *N*-protected hex-4-enylamines and 1-, 3-, and 4-substituted pent-4-enylamines. Scope, limitations, and mechanism of pyrrolidine formation. *J. Org. Chem.* **73**, 8851–8860 (2008).
10. Reddy, B. V. S., Borkar, P., Yadav, J. S., Sridhar, B. & Greè, R. Tandem Prins/Friedel–Crafts cyclization for stereoselective synthesis of heterotricyclic systems. *J. Org. Chem.* **76**, 7677–7690 (2011).
11. Berger, F., Plutschack, M. B., Riegger, J., Yu, W., Speicher, S., Ho, M., Frank, N. & Ritter, T. Site-selective and versatile aromatic C–H functionalization by thianthrenation, *Nature* **567***,* 223–228 (2019).
12. Agilent (2014). CrysAlis PRO. Agilent Technologies Ltd, Yarnton, Oxfordshire, England.
13. Dolomanov, O. V., Bourhis, L. J., Gildea, R. J., Howard, J. A. K. & Puschmann, H. OLEX2: a complete structure solution, refinement and analysis program. *J. Appl. Crystallogr.* **42**, 339–341 (2009).
14. Johnson, E. R., Keinan, S., Mori-Sánchez, P., Contreras-García, J., Cohen, A. J. & Yang, W. Revealing noncovalent interactions. *J. Am. Chem. Soc.* **132**, 6498–6506 (2010).
15. Contreras-García, J., Johnson, E. R., Keinan, S., Chaudret, R., Piquemal, J.-P., Beratan, D. N. & Yang, W. NCIPLOT: A program for plotting noncovalent interaction regions. *J. Chem. Theory Comput.* **7**, 625–632 (2011).
16. Lu, T. & Chen, F. Multiwfn: A multifunctional wavefunction analyser. *J. Comput. Chem.* **33**, 580–592 (2012).
17. Humphrey, W., Dalke, A. & Schulten, K. VMD: Visual molecular dynamics. *J. Molec. Graphics* **14**, 33–38 (1996).
