# Supplementary material for: Stereospecific syn-dihalogenations and regiodivergent syn-interhalogenation of alkenes via vicinal double electrophilic activation strategy
Source: Nat Commun. 2024 May 2;15:3710. doi: 10.1038/s41467-024-47942-w (PMC11066093; doi:10.1038/s41467-024-47942-w)
Supplement: Supplementary file 3 — Description of Additional Supplementary Files [file 41467_2024_47942_MOESM3_ESM.docx]

**File Name:** Supplementary Data 1
**Description:** the computation output files
